# IGF2BP3 As a Prognostic Biomarker and Regulator of Metastasis in Merkel Cell Carcinoma

**DOI:** 10.1016/j.xjidi.2025.100355

**Published:** 2025-02-12

**Authors:** Yajie Yang, Jiwei Gao, Hao Shi, Harri Sihto, Sami Kilpinen, François Vilcot, Libuse Janská, Jakob Jeschonneck, Todor Cvetanovic, Anders Höög, Jan Siarov, John Paoli, C. Christofer Juhlin, Lisa Villabona, Catharina Larsson, Weng-Onn Lui

**Affiliations:** 1 Department of Oncology-Pathology, Karolinska Institutet and BioClinicum, Karolinska University Hospital, Solna, Sweden; 2 Department of Pathology, University of Helsinki and Helsinki University Hospital, Helsinki, Finland; 3 Molecular and Integrative Biosciences Research Programme, University of Helsinki, Helsinki, Finland; 4 Department of Clinical Pathology and Cancer Diagnostics, Karolinska University Hospital, Solna, Stockholm, Sweden; 5 Department of Laboratory Medicine, Institute of Biomedicine, Sahlgrenska Academy, University of Gothenburg, Gothenburg, Sweden; 6 Department of Dermatology and Venereology, Sahlgrenska Academy, University of Gothenburg, Gothenburg, Sweden

**Keywords:** Biomarker, IGF2BP3, Merkel cell carcinoma, Metastasis

## Abstract

Merkel cell carcinoma (MCC) is an aggressive skin cancer with frequent metastasis; however, effective treatment options for advanced disease are often lacking. In this study, we investigated the clinical significance and functional impact of IGF2 mRNA-binding protein 3 (IGF2BP3) in MCC. Our results revealed elevated IGF2BP3 expression in metastases compared to that in primary tumors. High *IGF2BP3* levels in primary MCCs were associated with shorter disease-specific survival rates. In an MCC xenograft model, the lung metastases exhibited increased IGF2BP3 expression. Functional studies showed that IGF2BP3 primarily regulates MCC cell migration and invasion. We identified 281 direct RNA targets of IGF2BP3 with enriched functions linked to metastasis-related processes, and several targets overlapped with genes differentially expressed between MCC primary tumors and metastases, implying that IGF2BP3 and its targets contribute to tumor progression. Inhibition or silencing of bromodomain-containing protein 4 reduced IGF2BP3 expression, suggesting that bromodomain-containing protein 4 is a potential regulator of IGF2BP3. Our study underscores the role of IGF2BP3 in MCC metastasis and its potential as a prognostic biomarker.

## Introduction

Merkel cell carcinoma (MCC) is an aggressive skin cancer with high recurrence and mortality rates (Mistry et al, 2021). The diagnosis of MCC is often made at an advanced stage, which is related to its nonspecific symptoms (Gauci et al, 2022; Heath et al, 2008; Svensson and Paoli, 2021). Approximately 40% of MCC cases progress to regional or distant metastasis, resulting in a median survival of only 9 months after metastasis detection (Miller et al, 2013). Approximately half of the patients with metastatic disease benefit from immune checkpoint blockade; however, many eventually develop resistance (D’Angelo et al, 2020; Kaufman et al, 2016). Hence, there is an urgent need to identify early prognostic biomarkers and understand the mechanisms driving MCC progression.

IGF2 mRNA-binding protein 3 (IGF2BP3) is an oncofetal protein predominantly expressed during embryogenesis (Mori et al, 2001; Mueller-Pillasch et al, 1999). It is also frequently overexpressed and associated with adverse outcomes and metastasis in various cancer types (Mancarella and Scotlandi, 2019), such as melanoma (Sheen et al, 2015), liver (Jeng et al, 2008), bladder (Huang et al, 2023; Lee et al, 2014; Sitnikova et al, 2008), colorectal (Chen et al, 2023; Lochhead et al, 2012), and endometrial (Visser et al, 2019; Wang et al, 2022) cancers. Elevated IGF2BP3 levels are driven by transcriptional regulation involving factors such as MYC (Du et al, 2022), NANOG (Chen et al, 2013), RELA/p65 (Bhargava et al, 2017), and the MLL-AF4 fusion (Tran et al, 2022). As an RNA-binding protein, IGF2BP3 critically controls tumor invasiveness and metastasis by regulating the stability and translation of multiple oncogenic transcripts, such as *HMGA2* (Jeng et al, 2008; Sheen et al, 2015), *E2F3* (Wang et al, 2022), and *EGFR* (Chen et al, 2023).

IGF2BP3 is frequently detected in MCC (Dasgeb et al, 2019; Pryor et al, 2009), but its clinical significance and biological roles of IGF2BP3 remain unexplored. This study aimed to elucidate the clinical relevance and functional role of IGF2BP3 in MCC. We found increased levels of IGF2BP3 in metastases and a marginal association between elevated IGF2BP3 expression and survival, suggesting its potential as a biomarker. Functional studies have demonstrated that increased IGF2BP3 promotes cell migration and invasion through the regulation of key transcripts. In addition, treatment with bromodomain and extra-terminal motif (BET) inhibitors or bromodomain-containing protein (BRD) 4 silencing effectively reduced IGF2BP3 expression, implicating BRD4 as a regulator of IGF2BP3.

## Results

### Comparison of IGF2BP3 expression between primary tumors and metastases in MCC and pan-cancer

**Figure 1:**
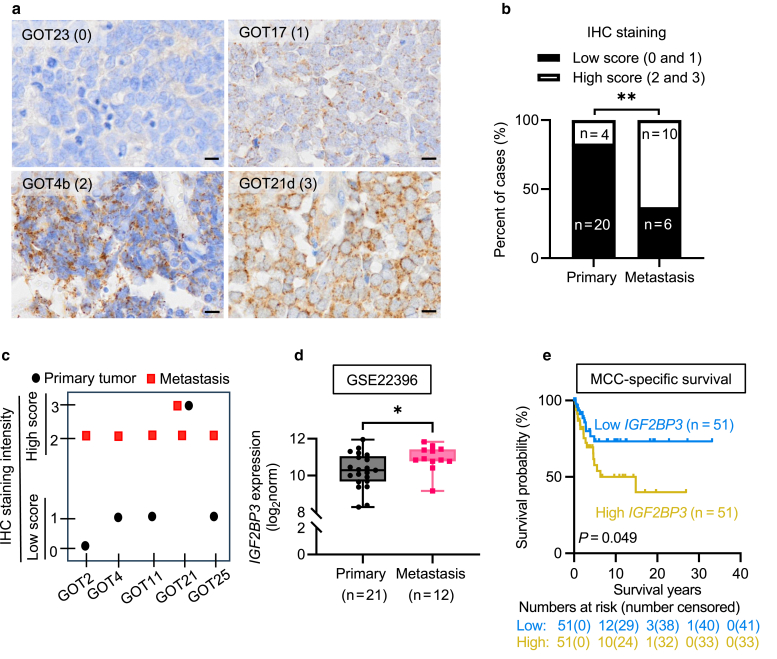
**Association of IGF2BP3 expression with metastasis and disease-specific survival in MCC.** (**a**) Representative IHC of IGF2BP3 in the Swedish MCC cohort showing negative (0), weak (1), intermediate (2), and strong (3) staining. Scale bar = 10 μm. (**b**) IHC analysis of IGF2BP3 expression between primary tumors and metastases in the Swedish cohort. ∗∗*P* < .01 by Fisher’s exact test. (**c**) IGF2BP3 expression in matched primary tumors and metastases from the same patients in the Swedish cohort. (**d**) *IGF2BP3* mRNA in primary tumors and metastases from the GSE22396 dataset. Log_2_norm, normalized expression in log_2_ scale. The box represents the median and interquartile range (25%–75%). ∗*P* < .05 by Mann-Whitney U-test. (**e**) Association of *IGF2BP3* mRNA expression with MCC-specific survival in the Finnish MCC cohort. *P* value was calculated by Log-rank (Mantel-Cox). IGF2BP3, IGF 2 mRNA-binding protein 3; IHC, immunohistochemistry; MCC, Merkel cell carcinoma.

We first performed immunohistochemistry (IHC) to evaluate IGF2BP3 expression in a Swedish MCC cohort comprising 24 primary tumors and 16 metastases. A total of 33/40 samples (83%) displayed cytoplasmic IGF2BP3 expression of varying intensities (score 1:20; score 2:8; score 3:5), whereas 7 had no detectable expression (score 0) (Figure 1a and Supplementary Table S1). We observed that primary tumors were more frequent in scores 0 and 1, whereas tumor metastases were more common in scores 2 and 3 (*P* = .0048 for scores 0 vs 1 vs 2 vs 3, Fisher’s exact test; Supplementary Figure S1). Categorizing IGF2BP3 expression into groups with low (0 and 1) and high (2 and 3) scores revealed significantly higher levels in metastases than in primary tumors (*P* = .0051, Fisher’s exact test; Figure 1b). Specifically, high score expression was observed in 10/16 metastases (63%) compared with 4/24 primary tumors (17%). Five patients in the Swedish MCC cohort had paired primary tumors and metastases (Figure 1c). All metastases exhibited a high score for IGF2BP3 expression, whereas all, except one, of the corresponding primary tumors had a low score.

Further analysis of *IGF2BP3* mRNA expression using the Gene Expression Omnibus (GEO) microarray dataset (GSE22396) revealed higher levels of *IGF2BP3* expression in MCC metastases than in primary tumors (Figure 1d). However, no significant differences were observed between the 2 groups in the GSE50451 and GSE39612 datasets (Supplementary Figure S2a). Despite the critical role of Merkel cell polyomavirus (MCPyV) in MCC, our analysis found no difference in *IGF2BP3* levels between virus-positive and -negative MCC in GEO datasets (Supplementary Figure S2b).

Extensive analyses were performed across diverse tumor types using The Cancer Genome Atlas (TCGA) pan-cancer data from University of California Santa Cruz (UCSC) Xena (https://xenabrowser.net/) and TNMplot (https://tnmplot.com/analysis/). These analyses consistently demonstrated elevated *IGF2BP3* levels in metastases versus primary tumors within the TCGA pan-cancer cohorts and the TCGA skin cancer dataset (Supplementary Figure S3a) and in skin, colon, liver, and kidney cancers from the TNMplot (Supplementary Figure S3b), highlighting its crucial role in tumor metastasis across multiple tumor types.

### Association of *IGF2BP3* expression with clinical parameters in MCC

To determine the clinical relevance of IGF2BP3 in MCC, we first analyzed *IGF2BP3* mRNA levels in the GSE235092 cohort, which comprised tumors classified as stage I–II, III, or IV. In this cohort, *IGF2BP3* expression was higher in stage IV tumors than in stages I–II, but this difference did not reach statistical significance (*P* = .0553; Supplementary Figure S4). We subsequently analyzed the Finnish MCC cohort, which comprised 102 primary tumors with extensive longitudinal clinical data spanning approximately 30 years. High *IGF2BP3* mRNA expression was marginally associated with adverse MCC-specific survival (Figure 1e), whereas overall survival did not reach statistical significance (Supplementary Figure S5). In addition, we identified associations between *IGF2BP3* mRNA levels and the clinical parameters (Table 1). Specifically, tumors with higher *IGF2BP3* expression were significantly larger, approximately double the size of those in the low-expression group (median = 22 mm vs 12 mm; *P* < .001). Patients with high *IGF2BP3* expression were also more frequently diagnosed at advanced stages (67% in stage II, 78% in stage III, and 67% in stage IV), whereas low-expression cases were more often at stage I (63%) (*P* = .018). Moreover, we observed differences in *IGF2BP3* levels based on the anatomical site; tumors located in the head and neck showed lower levels than those in the limb and trunk. Consistent with observations from MCC microarray datasets, no significant association was found between *IGF2BP3* expression and MCPyV status. Although a large proportion of primary tumors with high *IGF2BP3* expression developed metastasis during follow-up (62%) and at initial diagnosis (75%) compared with those with low expression, the difference was not statistically significant (*P* = .077 and *P* = .054, respectively).

**Table 1:** Comparison of *IGF2BP3* Expression With MCC Clinical Pathological Features of Primary Tumors in the Finnish MCC Cohort

Parameter, N	*IGF2BP3* Expression^1^		*P* Value
	Low N (%)	High N (%)	
Sex (N = 102)			.092^3^
Male	8 (33.3)	16 (66.7)	
Female	43 (55.1)	35 (44.9)	
Age (N = 102)			.285^4^
Median (Min–Max)	80 (51–93)	79 (46–100)	
Tumor diameter, mm (N = 87)			<.001^2^^,^^4^
Median (Min–Max)	12 (3–27)	22 (4–50)	
Tumor site (N = 102)			.047^2^^,^^5^
Head (N = 64)	38 (59.4)	26 (40.6)	
Limb (N = 28)	10 (35.7)	18 (64.3)	
Trunk (N = 10)	3 (30.0)	7 (70.0)	
MCPyV status^6^ (N = 94)			.092^3^
Negative (N = 31)	11 (35.5)	20 (64.5)	
Positive (N = 63)	34 (54.0)	29 (46.0)	
Stage (N = 85)			.018^2^^,^^5^
I (N = 49)	31 (63.3)	18 (36.7)	
II (N = 24)	8 (33.3)	16 (66.7)	
III (N = 9)	2 (22.2)	7 (77.8)	
IV (N = 3)	1 (33.3)	2 (66.7)	
Metastasis during follow-up (N = 99)			.077^3^
Yes (N = 34)	13 (38.2)	21 (61.8)	
No (N = 65)	37 (56.9)	28 (42.2)	
Metastasis at diagnosis (N = 96)			.054^3^
Yes (N = 12)	3 (25.0)	9 (75.0)	
No (N = 84)	46 (54.8)	38 (45.2)	

Abbreviations: IGF2BP3, IGF2 mRNA-binding protein 3; LT, large T antigen; MCPyV, Merkel cell polyomavirus; N, number of informative.

1 Expression values were extracted from previously published RNA-seq data (Sundqvist et al, 2023, 2022).

2 Significant *P* values.

3 Chi-square test.

4 Mann-Whitney U-test.

5 Fisher-Freeman-Halton exact test.

6 MCPyV status was assessed by immunohistochemical detection of MCPyV LT, as previously described (Sihto et al, 2011).

### IGF2BP3 expression in primary tumors and lung metastases of MCC xenograft mouse model

**Figure 2:**
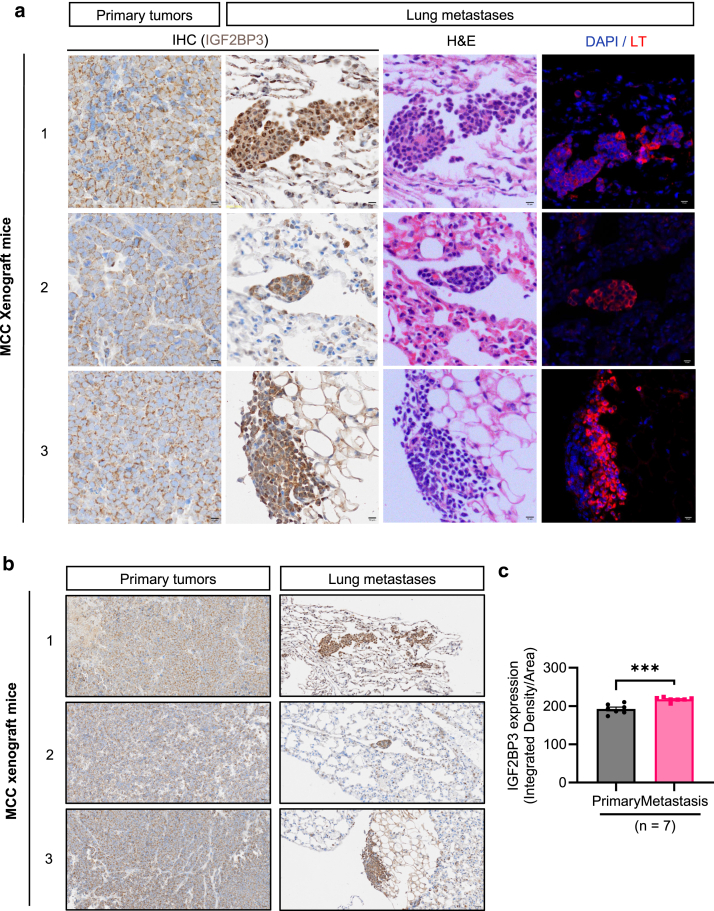
**Elevated IGF2BP3 expression in lung metastases compared to primary tumors in MCC xenograft model.** (**a**) Examples of IHC images depicting IGF2BP3 expression in primary tumors and lung metastases of the xenograft model. H&E staining and immunofluorescence detection of MCPyV LT (red) were performed in consecutive sections of the same lung metastasis. Nuclei were counterstained with DAPI (blue). Scale bar = 10 μm. The numbers represent different xenograft mice. (**b**) Lower magnification of the same tumors shown in (**a**), revealing specific IGF2BP3 immunoreactivity mostly in MCC cells. Scale bar = 20 μm. (**c**) Quantification of IGF2BP3 IHC in mice xenograft tumors and lung metastases was performed by calculating the integrated density per area (Gray value/pixel^2^) of 3 areas for each sample using ImageJ (n = 7). (**a** and **b**) 1, 2, and 3 represent tumor sections from 3 xenograft mice. IGF2BP3, IGF2 mRNA-binding protein 3; IHC, immunohistochemistry; LT, large T antigen; MCC, Merkel cell carcinoma; MCPyV, Merkel cell polyomavirus.

To further verify the importance of IGF2BP3 in tumor metastasis, we examined its levels in primary tumors and corresponding lung metastases in an MCC xenograft mouse model established from MCPyV-positive WaGa cells. IHC staining revealed higher IGF2BP3 expression in lung metastases than in primary tumors (n = 7, Figure 2a–c). H&E staining and immunofluorescence staining of MCPyV large T antigen further confirmed MCC metastasis in the lung (Figure 2a and Supplementary Figure S6).

### Regulation of cell migration and invasion by IGF2BP3 in MCC cells

**Figure 3:**
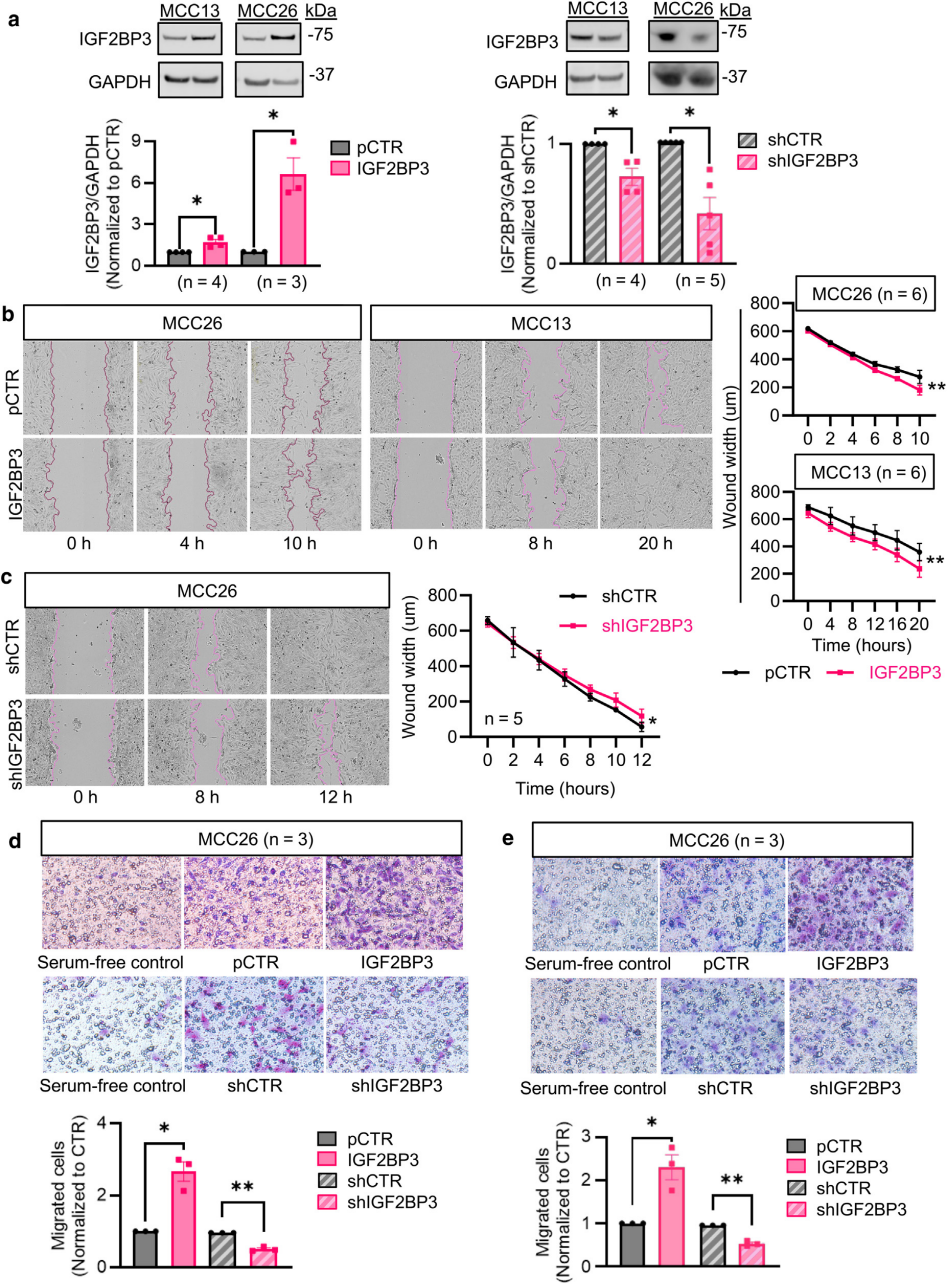
**Impact of IGF2BP3 silencing or overexpression on MCC cell migration and invasion.** (**a**) Immunoblot analysis showing the effects on IGF2BP3 expression after transfection with an IGF2BP3-expressing plasmid or shIGF2BP3 for 48 hours. Empty vectors (pCTR or shCTR), negative controls. GAPDH, loading control. (**b**) Wound healing demonstrating the effect of IGF2BP3 overexpression on cell migration. The wound width (average distance between wound boundaries) was calculated using the IncuCyte ZOOM software. (**c**) Wound healing assay by IncuCyte upon IGF2BP3 silencing. (**d**, **e**) Transwell migration assay (**d**) and invasion assay (**e**) in cells with IGF2BP3 overexpression or silencing. Serum-free medium, negative control. (**a**–**e**) Error bars represent mean ± SEM. ∗*P* < .05, ∗∗*P* < .01 were assessed by paired *t*-test (**a**, **d,** and **e**) or two-way ANOVA (**b** and **c**). GAPDH, glyceraldehyde-3-phosphate dehydrogenase; IGF2BP3, IGF2 mRNA-binding protein 3; MCC, Merkel cell carcinoma; pCTR, plasmid control; shCTR, short hairpin control.

To understand the role of IGF2BP3 in tumor progression, we studied its impact on cell migration and invasion using 2 adherent MCC cell lines, MCC26 and MCC13. Notably, alterations in IGF2BP3 expression had no effect on cell growth (Figure 3a, Supplementary Figure S7a and b) but significantly influenced wound closure rates (Figure 3b and c). Overexpression of IGF2BP3 accelerated wound closure in both cell lines (Figure 3b), whereas silencing IGF2BP3 specifically retarded closure in MCC26 cells (Figure 3c), with no observable difference in MCC13 cells (Supplementary Figure S8). Further examination via transwell assays revealed that IGF2BP3 overexpression promoted cell migration (Figure 3d) and invasion (Figure 3e), whereas its downregulation had the opposite effect (Figure 3d and e).

Given the reported impact of IGF2BP3 on cell apoptosis and viability in other tumor types (Huang et al, 2020; Yang et al, 2020), we examined these functional phenotypes in WaGa and MKL1 cells using Annexin V, WST-1, and trypan blue assays. However, our results did not reveal significant changes in MCC viability or apoptosis upon IGF2BP3 knockdown (Supplementary Figure S9). Together, our findings indicate that IGF2BP3 is not crucial for cell viability but is important for MCC tumor progression.

### Identification of IGF2BP3 RNA targets in MCC

**Figure 4:**
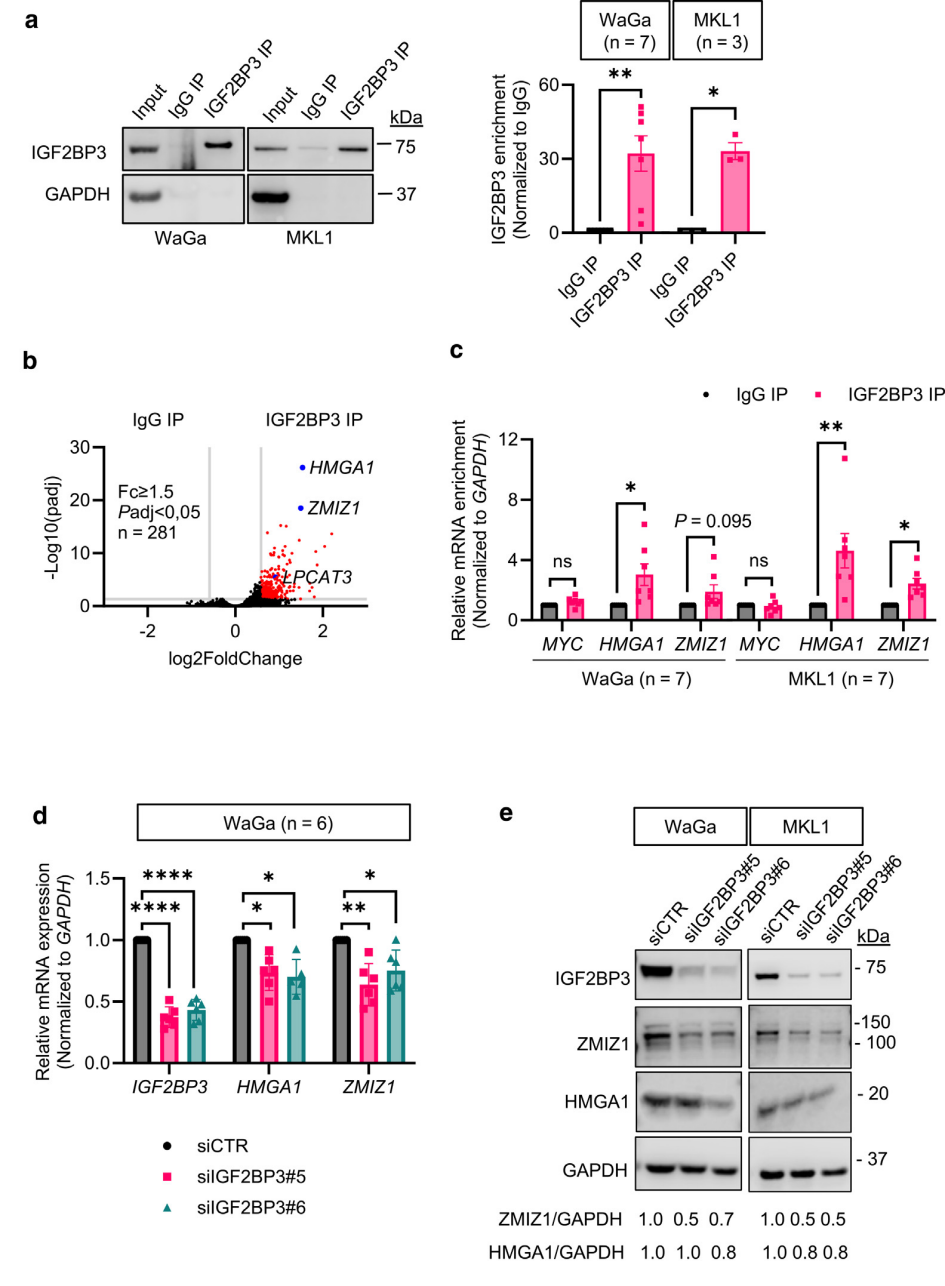
**Identification and validation of IGF2BP3 RNA targets in MCC cells.** (**a**) Immunoblotting showing the immunoprecipitation efficiency in RIP using IGF2BP3 antibody. IgG IP, negative control. GAPDH detection was used for evaluating the IGF2BP3 antibody’s binding specificity. (**b**) RIP sequencing was performed in WaGa cells (n = 3). A volcano plot displays genes with a fold change of 1.5 or higher and *P*adj lower than 0.05 (in red) between IgG IP and IGF2BP3 IP. (**c**) Validation of IGF2BP3-bound transcripts by RIP RT-qPCR. *MYC*, negative control. (**d**) RT-qPCR analysis of the IGF2BP3 target genes upon silencing of IGF2BP3. (**e**) Immunoblotting analysis of target genes upon IGF2BP3 silencing. (**a**, **c,** and **d**) Error bars represent mean ± SEM. ∗*P* < .05, ∗∗*P* < .01, ∗∗∗∗*P* < .0001; ns indicates not significant by paired *t*-test (**a** and **c**) or one-way ANOVA with Dunnett’s test (**d**). Fc, fold change; GAPDH, glyceraldehyde-3-phosphate dehydrogenase; IGF2BP3, IGF2 mRNA-binding protein 3; IP, immunoprecipitation; MCC, Merkel cell carcinoma; ns, not significant; *P*adj, *P* adjusted; RIP, RNA immunoprecipitation.

IGF2BP3 functions by binding to specific RNA targets and regulating their stability or translation (Schneider et al, 2019). To elucidate its role in MCC, we conducted RNA immunoprecipitation sequencing (RIP-seq) in WaGa cells (Figure 4a). Comparative analysis between RNAs pulled down by IGF2BP3 immunoprecipitation (IP) and IgG IP (negative control) revealed significant enrichment of 281 RNAs (fold change ≥ 1.5 and *P*adj < .05) in the IGF2BP3 IP (Figure 4b, Supplementary Table S2). Among these, *HMGA1* and *ZMIZ1* emerged as top hits and were further validated using RT-qPCR. Both *HMGA1* and *ZMIZ1* showed substantial enrichment in the IGF2BP3 IP in both WaGa and MKL1 cell lines, whereas no enrichment was found for *MYC*, a negative control that was not enriched in the IGF2BP3 IP by RIP-seq (Figure 4c). Knockdown of *IGF2BP3* resulted in reduced mRNA and protein levels of HMGA1 and ZMIZ1, confirming them as IGF2BP3 targets (Figure 4d and e).

### Functional analysis and clinical relevance of the IGF2BP3 targets

**Figure 5:**
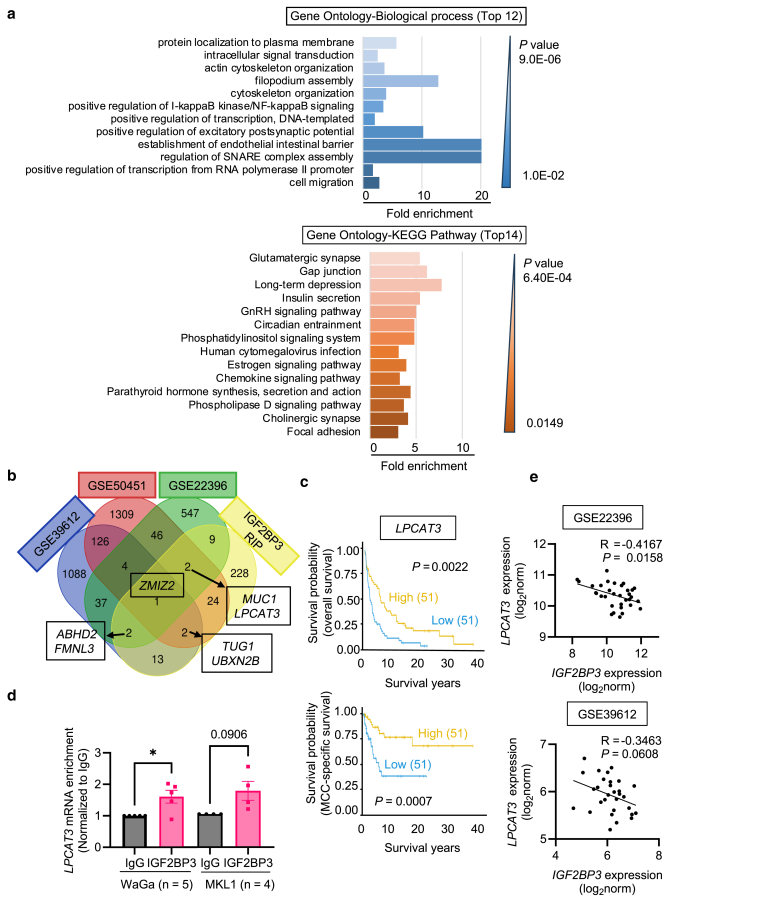
**Functional annotation of IGF2BP3 targets.** (**a**) IGF2BP3 targets identified by RIP sequencing were subjected to Gene Ontology–Biological process and KEGG pathway analyses using DAVID (https://david.ncifcrf.gov/). (**b**) Venn diagram illustrating overlapped differentially expressed genes among the 3 MCC microarray datasets and the IGF2BP3 RIP-seq data. Seven genes (in boxes) present in at least 2 microarray datasets and the RIP-seq data were chosen for further investigation. (**c**) Kaplan-Meier plots showing the survival differences between patients with low (<median) and high (>median) expression of *LPCAT3*. *P* values were calculated by log-rank test. (**d**) Validation of *LPCAT3* as a direct target of IGF2BP3 by RIP RT-qPCR. Error bars represent mean ± SEM. ∗*P* < .05 by paired t-test. (**e**) Pearson’s correlation analysis between *IGF2BP3* and *LPCAT3* expressions in GEO datasets. DAVID, Database for Annotation, Visualization and Integrated Discovery; GEO, Gene Expression Omnibus; IGF2BP3, IGF2 mRNA-binding protein 3; KEGG, Kyoto Encyclopedia of Genes and Genomes; MCC, Merkel cell carcinoma; ns, not significant; RIP, RNA immunoprecipitation.

Enrichment analysis revealed that IGF2BP3 target genes are involved in biological processes related to tumor progression, such as cell migration, cytoskeleton organization, and filopodium assembly (Figure 5a and Supplementary Table S3) and metastasis-associated pathways, such as chemokine signaling, focal adhesion, gap junction, and phospholipase D signaling (Figure 5a and Supplementary Table S4). Overlapping IGF2BP3 target genes from RIP-seq with differentially expressed genes between primary tumors and metastases in 3 MCC datasets (GSE39612, GSE22396, and GSE50451; Supplementary Tables S5–S7), we found 7 common genes, including *ZMIZ2*, *ABHD2*, *FMNL3*, *TUG1*, *UBXN2B*, *MUC1* and *LPCAT3*. Six of these genes were previously identified as IGF2BP3-bound transcripts in other cell types (Supplementary Table S8). Three genes (*FMNL3*, *LPCAT3,* and *MUC1*) were significantly differentially expressed between primary tumors and metastases in TCGA pan-cancer (Supplementary Figure S10) and were associated with overall, disease-specific, and/or progression-free survival (Supplementary Figure S11). Consistently, these 3 genes were also associated with survival in our Finnish MCC cohort (Figure 5c and Supplementary Figure S12). In particular, low *LPCAT3* levels were associated with shorter overall and MCC-specific survival (Figure 5c). This gene was also enriched in the IGF2BP3 IP by RIP RT-qPCR (Figure 5d) and was inversely correlated with *IGF2BP3* expression (Figure 5e), supporting its interaction with IGF2BP3. Together, our results suggest that IGF2BP3 and its target genes may contribute to tumor progression and serve as prognostic biomarkers for MCC.

### Reduced IGF2BP3 expression and cell viability in MCC cells treated with BET protein inhibitor JQ1 or degrader dBET1

**Figure 6:**
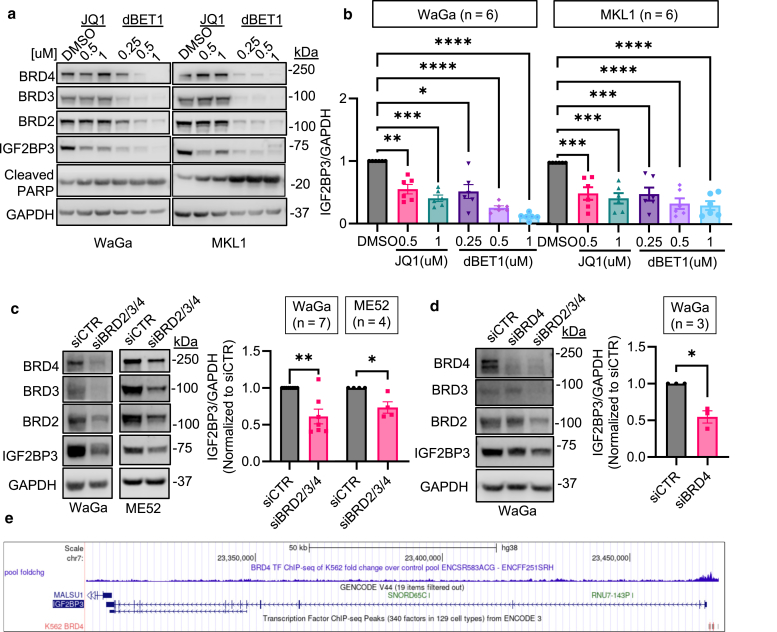
**Impact of BET inhibitor/degrader treatment and BRD4 silencing on IGF2BP3 expression in MCC cells.** (**a**) Immunoblots displaying the effects after 48 hours of treatments with BET inhibitor (JQ1) or degrader (dBET1). Cleaved PARP, apoptotic marker. GAPDH, loading control. (**b**) Quantification of IGF2BP3 expression from (**a**). (**c**) Evaluation of IGF2BP3 expression after simultaneous silencing of BRD2/3/4 by immunoblotting. (**d**) Assessment of IGF2BP3 expression after silencing BRD4. Simultaneous silencing of BRD2/3/4 was performed in parallel as a positive control. (**e**) An illustration showing the BRD4 binding sites identified by ChIP-seq from the UCSC genome browser (https://genome.ucsc.edu/). (**b, c,** and **d**) Error bars represent mean ± SEM. *P* values were assessed by one-way ANOVA with Dunnett’s test (**b**) or paired *t*-test (**c** and **d**). BET, bromodomain and extra-terminal motif; BRD, bromodomain-containing protein; ChIP-seq, chromatin immunoprecipitation sequencing; GAPDH, glyceraldehyde-3-phosphate dehydrogenase; IGF2BP3, IGF2 mRNA-binding protein 3; MCC, Merkel cell carcinoma; PARP, poly(ADP-ribose) polymerase; UCSC, University of California Santa Cruz.

The BET bromodomain inhibitor JQ1 has been shown to reduce IGF2BP3 expression in neonatal megakaryocytes (Elagib et al, 2017) and Ewing sarcoma cells (Mancarella et al, 2018). In addition, BET protein degraders provide more effective and sustained inhibition by promoting their degradation, a mechanism distinct from that of classical BET inhibitors, such as JQ1 (Choi et al, 2019). Given these characteristics, we chose JQ1 and degrader dBET1 to evaluate their impact on IGF2BP3 expression in MCC cells. Consistent with the findings in neonatal megakaryocytes (Elagib et al, 2017) and Ewing sarcoma (Mancarella et al, 2018), both JQ1 and dBET1 dose-dependently reduced IGF2BP3 protein levels in WaGa and MKL1 cells (Figure 6a and b). Notably, dBET1 displayed a stronger suppressive effect on IGF2BP3 than JQ1 at equivalent concentrations.

Consistent with the observations by Choi et al (2019), both JQ1 and dBET1 triggered apoptosis and reduced viability in both WaGa and MKL1 cells (Supplementary Figure S13). Cell viability was significantly decreased in both cell lines after treatment with JQ1 or dBET1, as assessed using trypan blue and WST-1 assays. Using the Annexin V assay, we observed an increase in apoptosis in WaGa cells treated with JQ1 or dBET1, whereas this effect was only observed in MKL1 cells treated with dBET1. Nonetheless, the apoptotic effect was evident in both cell lines treated with JQ1 or dBET1, as indicated by the increased expression of cleaved PARP, an apoptosis marker (Figure 6a). Collectively, our results demonstrate that both JQ1 and dBET1 effectively reduced IGF2BP3 expression and affected cell viability in MCC cells.

### Reduced expression of IGF2BP3 upon silencing of BRD4

Since JQ1 is an inhibitor of BET proteins, including BRD2, BRD3, BRD4, and the testis-specific BRDT (Filippakopoulos et al, 2010), we investigated their regulatory roles in IGF2BP3 expression. Simultaneous silencing of BRD2, BRD3, and BRD4 (siBRD2/3/4) in both WaGa and ME52 cells led to a significant downregulation of IGF2BP3 expression (Figure 6c). To identify the specific BRD protein regulating IGF2BP3 expression, we individually silenced BRD2, BRD3, and BRD4 in WaGa cells and found that only BRD4 silencing resulted in reduced IGF2BP3 expression (Figure 6d and Supplementary Figure S14). Consistent with BRD4 being a transcription regulator, analysis of chromatin immunoprecipitation sequencing data revealed putative BRD4 binding sites upstream of the transcription start site of *IGF2BP3* (Figure 6e), indicating a potential interaction between BRD4 and the promoter/enhancer of *IGF2BP3*. *BRD4* expression was also positively correlated with *IGF2BP3* expression in MCC (Supplementary Figure S15a).

*BRD4* expression, like IGF2BP3, was higher in tumor metastases than in primary tumors or normal tissues in both the TCGA pan-cancer and TNM skin cancer cohorts (Supplementary Figure S15b). Moreover, Kaplan-Meier survival analysis revealed that elevated *BRD4* levels were associated with poorer overall survival, disease-specific survival, progression-free interval, and disease-free interval (Supplementary Figure S15c). These findings suggest that BRD4 and IGF2BP3 are functionally related and contribute to tumor progression.

## Discussion

Timely detection of metastatic disease is crucial for improving clinical outcomes in MCC, as advanced stages of the disease present significant challenges in terms of treatment and management. Although reliable early detection biomarkers are still lacking, our study contributes by identifying IGF2BP3 as a potential prognostic biomarker and a key player in tumor progression.

We demonstrated an association between elevated IGF2BP3 expression and metastasis, as well as a marginal association with adverse disease-specific survival in MCC, which aligns with similar observations in diverse cancer types, including melanoma (Hanniford et al, 2020; Sheen et al, 2015), breast cancer (Jiang et al, 2022), nasopharyngeal carcinoma (Du et al, 2022; Xu et al, 2022), pancreatic cancer (Taniuchi et al, 2014), prostate cancer (Yu et al, 2022), Ewing sarcoma (Mancarella et al, 2020), and bladder cancer (Liu et al, 2022). These findings suggest that IGF2BP3 may have broader implications beyond MCC, indicating its potential as a universal biomarker across various malignancies.

Furthermore, our study revealed that IGF2BP3 primarily regulates cell migration and invasion in MCC. We identified several targets of IGF2BP3 linked to cell motility and invasion processes, including *HMGA1*, *ZMIZ1,* and *LPCAT3*. These targets have been implicated in tumor recurrence, metastasis promotion, regulation of cell migration, and patient survival (Mathios et al, 2019; Ouyang et al, 2022; Rogers et al, 2013; Wang et al, 2019; Zhu et al, 2022). Although we did not observe significant associations between *HMGA1* or *ZMIZ1* and clinical outcomes in our MCC cohort, *LPCAT3* emerged as a promising candidate associated with both overall and MCC-specific survival. These findings underscore the potential prognostic value of LPCAT3 in MCC cases. Nevertheless, further studies with independent cohorts are warranted to validate the prognostic impact of LPCAT3 and gain deeper insights into its biological roles in MCC.

Despite the previously recognized impact of IGF2BP3 on cell proliferation and apoptosis in cancer cells (Huang et al, 2020), we did not observe significant effects on these processes upon modulating IGF2BP3 expression in MCC cells. This suggests that IGF2BP3 is not essential for cell growth and viability in MCC but primarily contributes to tumor progression. The detailed mechanisms by which IGF2BP3 drives MCC tumor progression warrant further investigation.

In addition, we demonstrated that both JQ1 and dBET1 effectively reduced IGF2BP3 expression in MCC cells, implicating the involvement of BRD family proteins in the regulation of IGF2BP3 levels. Gene silencing experiments revealed that specifically reducing BRD4, but not BRD2 or BRD3, affected IGF2BP3 levels. Although direct evidence of the interaction between BRD4 and the IGF2BP3 promoter/enhancer was not provided in our study, analysis of BRD4 chromatin immunoprecipitation sequencing in the K562 leukemia cell line identified potential binding sites upstream of the transcription start site of *IGF2BP3* expression, supporting the role of BRD4 in controlling *IGF2BP3* transcription.

Furthermore, our pan-cancer analysis revealed a significant association between *BRD4* expression and metastasis and survival, underscoring the involvement of BRD4 and IGF2BP3 in tumor progression across various cancer types. Our results suggest that targeting BRD family proteins may be a promising strategy for inhibiting IGF2BP3 expression and potentially affecting tumor progression in MCC and other cancers.

In conclusion, our study highlights the potential of IGF2BP3 as a prognostic biomarker for MCC and provides insights into the mechanisms underlying tumor progression. These findings may contribute to the development of reliable prognostic tools and targeted therapies for patients with advanced MCC.

## Materials and Methods

### Clinical samples

Two MCC cohorts were included in this study. The Swedish MCC cohort comprised 40 formalin-fixed paraffin-embedded tumors from 33 patients, of which 24 were primary tumors from 15 men and 9 women with a median age at diagnosis of 80 years (range: 58–98), whereas 16 were metastases from 9 men and 7 women with a median age at diagnosis of 78 years (range: 51–98) (Supplementary Table S1).

The Finnish nationwide MCC cohort included 102 primary tumors from 24 male and 78 female patients with a median age at diagnosis of 79 years (range: 46–100) (Sihto et al, 2011). Sixty-four tumors were found in the head, 28 in the limbs, and 10 in the trunk. The tumor samples were classified as stage I (49 cases), II (24 cases), III (9 cases), and IV (3 cases), according to the American Joint Committee on Cancer eighth edition staging system (Amin et al, 2017); 17 cases could not be evaluated because of missing information. Twelve patients had metastasis at diagnosis, whereas 34 developed metastasis during follow-up. Sixty-three tumors were MCPyV-positive, and 31 were negative. The clinicopathological information of the Finnish cohort is presented in Table 1.

The MCPyV status of 37 samples from the Swedish cohort was analyzed using PCR in this study (Supplementary Table S1), whereas the remaining samples were evaluated using PCR and/or IHC in previous studies (Sihto et al, 2011; Xie et al, 2014).

### MCC xenograft model

An MCC xenograft mouse model was previously established by subcutaneously injecting 2 × 10^7^ MCPyV-positive WaGa cells in 100 μl PBS into the right flank of 6-week-old Naval Medical Research Institute (NMRI)-nu/nu immunodeficient nude mice (n = 7) (Shi et al, unpublished data), in which multiple lung metastases were observed. IHC was performed on tissue sections obtained from 7 xenograft tumors and lung metastases. The entire lung was dissected at 150 μm intervals, and each section was subjected to H&E staining to enumerate lung metastases. Lung metastases were further verified by immunofluorescence detection of MCPyV large T antigen.

### Cell culture

Four established human MCC cell lines were used, including 2 MCPyV-positive (WaGa and MKL1) and 2 MCPyV-negative (MCC13 and MCC26) cell lines. Their sources, culture conditions, and authentication have been described in our previous publications (Kumar et al, 2020). In addition, a primary MCC culture, ME52, was established from a fresh surgical specimen after mechanical disaggregation and filtration through a 100-μm mesh filter. The cells were cultured at 37 °C and 5% CO_2_ in RPMI 1640 medium supplemented with 15% fetal bovine serum, 1% L-glutamine, and 1% penicillin and streptomycin. The establishment of the primary culture and confirmation of MCPyV-negative MCC were described by Shi et al (unpublished data). WaGa, MKL1, and ME52 cells grow in suspension, whereas MCC13 and MCC26 cells grow adherently.

### Data analysis of published MCC datasets

Gene expression profiles of MCC cohorts were retrieved from the GEO database (GSE22396, GSE39612, GSE50451, and GSE235092). To identify differentially expressed genes between primary tumors and metastases, we utilized GEO2R (https://www.ncbi.nlm.nih.gov/geo/geo2r/) for the GSE22396 and GSE50451 datasets and limma (Ritchie et al, 2015) for the GSE39612 dataset. To analyze *IGF2BP3* expression, the expression values and sample information from GSE22396, GSE39612, and GSE50451, were extracted, normalized, and transformed into a log2 scale using Limma for microarray data. The GSE235092 dataset, which consists of bulk RIP-seq data, was normalized and log2-transformed using DESeq2 (Love et al, 2014). All datasets were analyzed using GraphPad Prism 10 (GraphPad Software, Boston, MA).

The RIP-seq data (BioProject accession number PRJNA775071) and clinicopathological information of the Finnish MCC cohort have been described previously (Sundqvist et al, 2023, 2022). The expression values of the selected genes were normalized and transformed as log2 counts per million, in which their median values were used as thresholds to define high and low expression groups for each patient. The median was chosen to ensure balanced group sizes for statistical robustness, not because of pre-existing biological significance.

### UCSC Xena and TNMplot analysis

Gene expression data for *IGF2BP3*, *ZMIZ2*, *FMNL3*, *ABHD2*, *LPCAT3*, *MUC1*, *TUG1*, *UBXN2B,* and *BRD4* in the TCGA pan-cancer cohort were extracted using UCSC Xena (https://xenabrowser.net/), whereas *IGF2BP3* levels in specific cancer types were retrieved from the gene chip data of TNMplot (https://tnmplot.com/analysis/). The gene expression data from UCSC Xena were normalized using Transcripts Per Million to ensure comparability across samples. Batch effects were corrected to minimize technical variability, and the data were log2-transformed using the formula log2 (norm_value + 1). In the TNMplot database, microarray data were normalized using the MAS5 algorithm through the Bioconductor Affy package (Pepper et al, 2007), followed by a second scaling normalization to set the mean expression of each array to 1000. The processed gene expression values were plotted and analyzed using GraphPad Prism 10 software.

### Small interfering RNAs and plasmids

Small interfering RNA targeting *IGF2BP3* (siIGF2BP3#5, SI02655394; siIGF2BP3#6, SI04344256) and *BRD3* (siBRD3, SI05131147) were acquired from Qiagen, whereas *BRD2* (siBRD2, AM16708), *BRD4* (siBRD4, 4390824) and small interfering RNA control (siCTR, AM4635) were purchased from Thermo Fisher Scientific.

A short hairpin RNA targeting *IGF2BP3* (target sequence: CACAUUUAAUUCCUGGAUUAA; shIGF2BP3) was cloned into the BglII and KpnI sites of the pcDNA3-U6M2 plasmid. For the IGF2BP3-expressing plasmid, the *IGF2BP3* coding sequence was excised from pDESTmycIGF2BP3 (Addgene, #19879) and inserted into the pCMV6entry vector using NotI and SalI. The integrity of all plasmids was confirmed via sequencing.

### Transfection

For suspension cells (WaGa, MKL1, and ME52), 3 million cells were suspended in 100 μL of Ingenio electroporation solution (MIR50111; Mirus Bio LLC) and 20–30 pmol of small interfering RNA, and transfected using the D-24 (WaGa), A-23 (MKL1), or X-01 (ME52) program of the Nucleofector 2b Device (Lonza Bioscience). Adherent cells (MCC13 and MCC26) were transfected using Lipofectamine LTX with PLUS reagent (#15338100; Thermo Fisher Scientific) with 1 μg of plasmid DNA.

### RNA immunoprecipitation and sequencing

RNA immunoprecipitation (RIP) followed by sequencing (RIP-seq) was performed to identify the direct RNA targets of IGF2BP3. RIP was performed using 5 μg IGF2BP3 (# 57145; Cell Signaling Technology) or IgG (#3900; Cell Signaling Technology) antibodies and 100 μL of Protein G Sepharose 4 Fast Flow beads (GE17-0618-01; Sigma-Aldrich), following the procedures described previously. To digest the proteins, 1 mg/ml proteinase K was added and incubated for 30 minutes at 55 °C, followed by RNA extraction using Trizol Reagent (Thermo Fisher Scientific).

Library preparation and sequencing were performed by Novogene Company Limited on an Illumina HiSeq platform with 150 bp paired-end reads. The reads were aligned to the human genome (GRCh38) using STAR (version 2.7.9a). Quantification of transcripts was performed using rsem (https://github.com/deweylab/RSEM) with human gene annotation from gencode version 37. Differential gene expression analysis between IGF2BP3 IP and IgG IP was performed using DESeq2 (Love et al, 2014). RIP-seq data are available under the GEO accession number GSE280116.

### RT-qPCR

RNA was extracted from cells or beads using TRIzol reagent (Thermo Fisher Scientific). The concentration was determined using NanoDrop ND-2000 (Thermo Fisher Scientific). cDNA synthesis was performed using the High-Capacity cDNA Reverse Transcription Kit with RNase Inhibitor (#4374966, Applied Biosystems/Thermo Fisher Scientific). Gene expression was performed using pre-designed TaqMan assays (*GAPDH*, Hs99999905_m1; *IGF2BP3*, Hs00559907_g1; *MYC*, Hs00153408_m1; *ZMIZ1*, Hs01119362_m1, and *HMGA1*, Hs00852949_g1) and TaqMan Universal PCR Master Mix (#4304437; Thermo). The qPCR reaction was performed using the QuantStudio 1 Real-Time PCR System and analyzed using the QuantStudio Real-Time PCR Software version 1.3 (Thermo Fisher Scientific). *GAPDH* was used for normalization.

### Immunoblotting

Cell lysates were prepared using RIPA lysis and extraction buffer (#89900; Thermo Fisher Scientific) supplemented with protease inhibitor (#P2714; Sigma-Aldrich) and 1% PMSF (#93482; Sigma-Aldrich), and the protein concentration was quantified using the Pierce BCA Protein Assay Kit (#23225; Thermo Fisher Scientific). Protein lysates (30–60 ug) were separated via electrophoresis and transferred to nitrocellulose membranes (#LC2000; Thermo Fisher Scientific). The membrane was incubated with primary antibodies at 4 °C overnight, followed by incubation with fluorescence-conjugated secondary antibodies. Images were detected using the Odyssey Fc Image system (LI-COR Biosciences) and analyzed using Image Studio Lite (LI-COR Biosciences). The primary and secondary antibodies used and their dilutions are listed in Supplementary Table S9.

### Immunohistochemistry

Paraffin-embedded tissue sections (5-μm) were first deparaffinized with xylene and rehydrated with a descending ethanol series (100%, 95%, 70%, and 50%), followed by incubation with a peroxidase blocking reagent (3% H_2_O_2_ in water) to quench endogenous peroxidase activity. After blocking, the tissue sections were incubated with IGF2BP3 antibody (sc365640; 1:500; Santa Cruz Biotechnology) diluted in Renior Red diluent (#PD9004M; Biocare Medical), followed by detection using the Biocare Medical Mach-1 Universal HRP-Polymer Kit (MIU539L10). IGF2BP3 expression in human samples was evaluated based on the staining intensity and classified as follows: 0 (negative), 1 (weak), 2 (intermediate), and 3 (strong) by 2 independent observers (JG and CCJ), blinded to clinical information. Cases with low scores (0 and 1) and with high scores (2 and 3) were grouped together for further analysis.

To quantify IGF2BP3 expression in mouse xenograft tumors and lung metastases, 3 random areas (600 × 600 pixel^2^) were selected from each primary tumor or 3 different metastases, and the integrated density per area (integrated density/pixel^2^) was calculated using ImageJ software. We noted that some cells in the lung metastases had both cytoplasmic and nuclear staining for IGF2BP3, which were also stained positive for MCPyV large T antigen; therefore, both cytoplasmic- and nuclear-stained cells were included for quantification.

### Immunofluorescence

The slides underwent deparaffinization and rehydration, followed by antigen retrieval using 100 mM sodium citrate buffer (pH = 6.0, Sigma-Aldrich, #C9999) at 95 ºC for 15 minutes. The slides were permeabilized with 0.5% Triton X-100/1% bovine serum albumin for 10 minutes, quenched for autofluorescence using the Vector TrueVIEW Autofluorescence Quenching Kit (Vector Laboratories; Catalog #SP-8400), and blocked with 0.3% Triton X-100/2% bovine serum albumin for 20 min at room temperature. After washing, the slides were incubated with MCPyV large T antigen–antibody (sc-136172; Santa Cruz Biotechnology; 1:50) overnight at 4 °C, followed by Alexa Fluor 633 anti-mouse secondary antibody (A21050; Invitrogen; 1:400) at room temperature for 1 hour. Nuclei were counterstained with DAPI-containing Vector Shield mounting medium (#H-1200; Vector Laboratories). Images were acquired using a Zeiss Observer 7 microscope and analyzed using the ZEN software (Carl Zeiss Microscopy GmbH).

### Annexin V apoptosis and trypan blue assays

Cell apoptosis was analyzed using the FITC Annexin V Apoptosis Detection Kit (556547; BD Biosciences) according to the manufacturer’s instructions. Stained cells were analyzed using the NovoCyte 3000 (Agilent).

Trypan blue assay was used to assess cell viability, and 10 μl of cell suspension was thoroughly mixed with an equal volume of 0.4% trypan blue solution (15250-061; Gibco/Thermo Fisher Scientific). The number of viable cells was counted using an automated cell counter (TC10; Bio-Rad).

### WST-1 assay

The treated or transfected cells were transferred into 96-well flat-bottom plates, with 5 × 10^4^ cells in a final volume of 100 μl per well. Subsequently, 10 μl of WST-1 reagent (Sigma-Aldrich) was added to each well, and the plates were incubated for 2 hours in a humidified incubator (37 °C, 5% CO_2_). The absorbance was measured using a Versamax microplate reader (Molecular Devices) at 450 nm and 650 nm (reference wavelength).

### Wound healing assay

Transfected cells were cultivated in 96-well flat-bottom plates until they reached 90% confluence. The IncuCyte WoundMaker tool was then used to create a standardized wound across the cell monolayer. The plate was transferred to an IncuCyte S3, which was programmed to capture images every 2 hours for MCC26 or every 4 hours for MCC13. Wound width was calculated using IncuCyte ZOOM software, which represents the average distance between the wound boundaries.

### Transwell migration and invasion assay

Cells were transfected with plasmids (IGF2BP3, pCTR, shIGF2BP3, or shCTR) for 48 h, followed by overnight starvation in serum-free medium. For the migration assay, starved cells (3 × 10^4^) were suspended in 500 μl of RPMI 1640 medium without fetal bovine serum and seeded into culture inserts with an 8 μm pore size membrane (PTEP24H48; Millipore/Merck). The inserts were placed in a 24-well plate with the outer compartment containing 750 μl of RPMI 1640 medium supplemented with 10% fetal bovine serum as chemotactic agents. The plate was incubated in a humidified incubator at 37 °C with 5% CO_2_ for 18 hours. For the invasion assay, the procedure was similar, except that culture inserts were coated with 100 μl of diluted Cultrex UltiMatrix RGF BME solution (BME001-05; R&D Systems; 1:10), and the plate was cultured for 24 hours before analysis. After removing non-migrated cells, the migrated cells were fixed using cold methanol and stained with 1% crystal violet solution (HT90132; Sigma-Aldrich) in ethanol. The migrated cells were counted in 3 random fields of equal size in each insert using an inverted microscope.

### Statistical analysis

All statistical analyses were performed using GraphPad Prism 10 (GraphPad Software). Fisher’s exact test determined the association of IGF2BP3 levels with primary tumors or metastases. A paired *t*-test compared the means of 2 conditions, whereas one-way ANOVA with post-hoc Dunnett’s-test analyzed 3 or more groups. The Mann-Whitney U-test compared the medians of 2 independent groups, whereas the Kruskal-Wallis with post-hoc Dunn’s-test assessed multiple groups. Two-way ANOVA estimated mean wound width changes between the 2 groups. Pearson’s correlation test measured linear correlation. MCC-specific survival and overall survival were assessed using the Finnish MCC cohort. MCC-specific survival refers to the time until death directly attributed to MCC, whereas overall survival accounts for deaths from any cause. Survival analysis was performed using the Kaplan Meier method and significance was assessed using the Log-rank (Mantel-Cox) test. Statistical significance: ∗*P* < .05, ∗∗*P* < .01, ∗∗∗*P* < .001, ∗∗∗*P* < .0001.

## Ethics Statement

This study was approved by the Swedish Ethical Review Authority (Dnr 2010/1092-31/3) and the Ethics Committee of the Helsinki University Central Hospital (HUS1455/2017). The collection of Finnish Merkel cell carcinoma samples and clinical data was approved by the Ministry of Health and Social Affairs (STM/398/2005) and the National Authority for Medicolegal Affairs (4942/05.01.00.0672009). The use of archival materials was authorized by the Stockholm Medical Biobank and Biobank West. All samples were coded to ensure that no personal identifiers were linked to the data. The need for informed consent was waived by the respective ethical committees and governmental agencies, as the study involved the use of pre-existing, de-identified materials, posing minimal risk to participants.

## Data Availability Statement

Datasets related to IGF 2 mRNA-binding protein 3 RNA immunoprecipitation sequencing in this article can be found at https://www.ncbi.nlm.nih.gov/geo/query/acc.cgi?acc=GSE280116, hosted at Gene Expression Omnibus (GSE280116). All other study data are available in the article and/or its supplementary materials or can be requested from the author.

## ORCIDs

Yajie Yang: http://orcid.org/0000-0002-4200-1015

Jiwei Gao: https://orcid.org/0000-0002-9786-853X

Hao Shi: https://orcid.org/0000-0003-0839-6516

Harri Sihto: https://orcid.org/0000-0001-5265-5509

Sami Kilpinen: https://orcid.org/0000-0003-3444-2539

François Vilcot: https://orcid.org/0009-0000-3009-9601

Libuše Janská: https://orcid.org/0000-0002-7695-9686

Jakob Jeschonneck: https://orcid.org/0009-0002-0250-5451

Todor Cvetanovic: https://orcid.org/0000-0003-4728-5536

Anders Höög: https://orcid.org/0000-0001-7603-3421

Jan Siarov: https://orcid.org/0000-0002-2013-3113

John Paoli: https://orcid.org/0000-0003-1326-8535

C. Christofer Juhlin: https://orcid.org/0000-0002-5945-9081

Lisa Villabona: https://orcid.org/0000-0001-7702-4754

Catharina Larsson: https://orcid.org/0000-0003-4813-3657

Weng-Onn Lui: http://orcid.org/0000-0003-4717-4473

## Conflict of Interest

The authors state no conflict of interest.
