# Supplementary material for: IGF2BP3 As a Prognostic Biomarker and Regulator of Metastasis in Merkel Cell Carcinoma
Source: JID Innov. 2025 Feb 12;5(3):100355. doi: 10.1016/j.xjidi.2025.100355 (PMC11951866; doi:10.1016/j.xjidi.2025.100355)
Supplement: Supplementary Tables 1-9 [file mmc1.pdf]

Supplementary Table S1. Immunohistochemistry of IGF2BP3 expression in 40 tumor samples in the Swedish MCC cohort

Tumor ID [1]	Sex	Age atdiagnosis	MCPyV status [2]	Tumor sample	Immunohistochemistry Score [3]	Expression group [4]
GOT1	M	84	-	Primary	0	Low score
GOT2a	M	79	-	Primary	0	Low score
GOT2b	M	79	-	Metastasis	2	High score
GOT4a	M	98	-	Primary	1	Low score
GOT4b	M	98	-	Metastasis	2	Low score
GOT5	M	78	+	Primary	1	Low score
GOT6	F	91	+	Primary	1	Low score
GOT7	F	97	+	Primary	1	Low score
GOT9	F	88	+	Primary	1	Low score
GOT10	M	70	+	Primary	2	High score
GOT11a	M	58	-	Primary	1	Low score
GOT11c	M	58	-	Metastasis	2	High score
GOT12a	M	81	+	Primary	1	Low score
GOT13	M	74	+	Primary	0	Low score
GOT14a	M	78	-	Metastasis	0	Low score
GOT14b	M	78	-	Metastasis	1	Low score
GOT15	F	73	+	Primary	3	High score
GOT16	F	84	-	Primary	1	Low score
GOT17	M	87	+	Primary	1	Low score
GOT19	F	56	+	Metastasis	2	High score
GOT20a	M	79	-	Primary	1	Low score
GOT21a	M	69	+	Primary	3	High score
GOT21b	M	69	+	Metastasis	2	High score
GOT21d	M	69	+	Metastasis	3	High score
GOT22	F	92	-	Primary	1	Low score
GOT23	F	72	-	Primary	0	Low score
GOT24	M	83	+	Primary	1	Low score
GOT25a	F	81	-	Primary	1	Low score
GOT25b	F	81	-	Metastasis	2	High score
GOT26	M	63	+	Primary	1	Low score
GOT27a	M	51	-	Metastasis	2	High score
MCCT_1b*	F	91	-	Metastasis	1	Low score
MCCT_22*	F	63	+	Metastasis	3	High score
MCCT_24*	F	72	-	Metastasis	3	High score
MCCT_25*	M	84	+	Metastasis	1	Low score
MCCT_26*	F	85	-	Metastasis	1	Low score
MCCT_2a*	F	91	-	Primary	1	Low score
MCCT_4a*	M	69	+	Primary	1	Low score
MCCT_6a*	M	74	+	Primary	0	Low score
MCCT_7b*	F	87	+	Metastasis	0	Low score

[1] a, b, c, d or e = different tumors from the same patient

[2] MCPyV status (positive, + and negative, -) was determined by PCR of tumor genomic DNA, as described in Xie. et al. 2014. Samples analyzed in previous study are marked by *

[3] According to staining intensity: negative (score 0), weak (1), intermediate (2) or strong (3)

[4] Based on the IHC score: low score group (0 and 1), high score group (2 and 3)

F = female; M = male; MCPyV = Merkel cell polyomavirus

Supplementary Table S2. Identification of IGF2BP3 target genes by RIP seq

gene id	gene name	baseMean[1]	log2FC	lfcSE[2]	stat[3]	*P*- value	*P* adj
ENSG00000137309.20	*HMGA1*	3604.16	1.53	0.13	11.586	4.8E-31	6.2E-27
ENSG00000108175.18	*ZMIZ1*	2151.65	1.49	0.15	9.890	4.6E-23	2.9E-19
ENSG00000120685.20	*PROSER1*	1101.28	1.03	0.11	9.053	1.4E-19	5.9E-16
ENSG00000149679.11	*CABLES2*	1605.20	1.16	0.13	8.988	2.5E-19	8.1E-16
ENSG00000128050.9	*PAICS*	10653.43	0.73	0.08	8.648	5.3E-18	1.3E-14
ENSG00000151176.8	*PLBD2*	158.32	2.20	0.26	8.564	1.1E-17	2.3E-14
ENSG00000090924.15	*PLEKHG2*	203.96	1.46	0.18	8.222	2E-16	3.6E-13
ENSG00000235437.8	*LINC01278*	85.83	2.04	0.26	7.861	3.8E-15	5.6E-12
ENSG00000157851.17	*DPYSL5*	1066.43	1.14	0.14	7.856	4E-15	5.6E-12
ENSG00000105426.17	*PTPRS*	748.56	0.90	0.12	7.764	8.2E-15	1.1E-11
ENSG00000164054.15	*SHISA5*	305.09	1.08	0.14	7.606	2.8E-14	3.3E-11
ENSG00000076201.15	*PTPN23*	127.51	1.84	0.24	7.545	4.5E-14	4.8E-11
ENSG00000106785.15	*TRIM14*	612.86	0.89	0.12	7.464	8.4E-14	8.2E-11
ENSG00000003249.15	*DBNDD1*	224.90	1.29	0.17	7.426	1.1E-13	1E-10
ENSG00000278259.5	*MYO19*	1154.59	0.80	0.11	7.387	1.5E-13	1.3E-10
ENSG00000113763.12	*UNC5A*	271.69	1.08	0.15	7.332	2.3E-13	1.8E-10
ENSG00000167461.12	*RAB8A*	828.52	0.81	0.11	7.305	2.8E-13	2.1E-10
ENSG00000048991.16	*R3HDM1*	1014.63	0.87	0.12	7.289	3.1E-13	2.2E-10
ENSG00000103353.16	*UBFD1*	2033.22	0.83	0.11	7.275	3.5E-13	2.3E-10
ENSG00000162512.16	*SDC3*	709.65	1.12	0.16	7.232	4.8E-13	3.1E-10
ENSG00000088256.9	*GNA11*	1037.27	0.99	0.14	7.163	7.9E-13	4.8E-10
ENSG00000164742.16	*ADCY1*	1495.40	1.00	0.14	7.133	9.8E-13	5.7E-10
ENSG00000177732.9	*SOX12*	427.89	1.24	0.18	6.942	3.9E-12	2.1E-09
ENSG00000161847.14	*RAVER1*	176.52	1.25	0.18	6.785	1.2E-11	6.2E-09
ENSG00000176087.16	*SLC35A4*	273.68	1.18	0.18	6.692	2.2E-11	1.1E-08
ENSG00000166340.17	*TPP1*	82.21	1.87	0.28	6.631	3.3E-11	1.6E-08
ENSG00000175220.12	*ARHGAP1*	345.14	0.99	0.15	6.530	6.6E-11	3.1E-08
ENSG00000168488.18	*ATXN2L*	788.58	0.83	0.13	6.378	1.8E-10	8.2E-08
ENSG00000143126.8	*CELSR2*	263.76	0.99	0.16	6.346	2.2E-10	9.7E-08
ENSG00000161202.20	*DVL3*	471.81	1.10	0.17	6.306	2.9E-10	1.2E-07
ENSG00000123416.15	*TUBA1B*	8463.02	0.92	0.15	6.296	3.1E-10	1.3E-07
ENSG00000168268.11	*NT5DC2*	530.04	0.80	0.13	6.278	3.4E-10	1.4E-07
ENSG00000134369.15	*NAV1*	625.42	1.11	0.18	6.237	4.4E-10	1.7E-07
ENSG00000105568.18	*PPP2R1A*	1073.99	0.68	0.11	6.192	5.9E-10	2.2E-07
ENSG00000094916.16	*CBX5*	20019.68	0.61	0.10	6.132	8.7E-10	3.2E-07
ENSG00000144677.15	*CTDSPL*	574.17	0.73	0.12	6.118	9.5E-10	3.4E-07
ENSG00000215114.10	*UBXN2B*	1841.84	0.74	0.12	6.083	1.2E-09	4.1E-07
ENSG00000109062.12	*SLC9A3R1*	216.69	0.97	0.16	6.011	1.8E-09	6.2E-07
ENSG00000198646.14	*NCOA6*	475.76	0.87	0.15	5.954	2.6E-09	8.6E-07
ENSG00000160691.19	*SHC1*	744.67	0.78	0.13	5.884	4E-09	1.3E-06
ENSG00000102225.16	*CDK16*	347.19	0.88	0.15	5.868	4.4E-09	1.4E-06
ENSG00000162104.10	*ADCY9*	201.54	1.24	0.21	5.858	4.7E-09	1.4E-06
ENSG00000126883.17	*NUP214*	498.83	1.00	0.17	5.849	5E-09	1.5E-06
ENSG00000100167.20	*SEPTIN3*	817.42	0.79	0.13	5.839	5.3E-09	1.5E-06
ENSG00000129595.14	*EPB41L4A*	281.70	0.85	0.15	5.755	8.7E-09	2.4E-06
ENSG00000134802.18	*SLC43A3*	787.80	0.67	0.12	5.758	8.5E-09	2.4E-06
ENSG00000151468.11	*CCDC3*	486.66	0.73	0.13	5.713	1.1E-08	3E-06

ENSG00000188807.13	*TMEM201*	161.41	1.07	0.19	5.706	1.2E-08	3.1E-06
ENSG00000185499.16	*MUC1*	176.39	1.18	0.21	5.689	1.3E-08	3.3E-06
ENSG00000103197.18	*TSC2*	287.56	0.83	0.15	5.691	1.3E-08	3.3E-06
ENSG00000175334.8	*BANF1*	316.10	0.88	0.16	5.681	1.3E-08	3.4E-06
ENSG00000140577.16	*CRTC3*	392.23	0.79	0.14	5.671	1.4E-08	3.5E-06
ENSG00000111684.11	*LPCAT3*	611.19	0.92	0.16	5.668	1.4E-08	3.5E-06
ENSG00000253352.10	*TUG1*	4101.36	0.59	0.10	5.657	1.5E-08	3.6E-06
ENSG00000122515.15	*ZMIZ2*	329.12	0.96	0.17	5.634	1.8E-08	4.1E-06
ENSG00000141576.16	*RNF157*	383.54	0.91	0.16	5.525	3.3E-08	7.5E-06
ENSG00000166257.10	*SCN3B*	195.58	0.96	0.18	5.409	6.3E-08	1.4E-05
ENSG00000149639.15	*SOGA1*	821.15	1.02	0.19	5.401	6.6E-08	1.5E-05
ENSG00000173258.13	*ZNF483*	1145.07	0.61	0.11	5.397	6.8E-08	1.5E-05
ENSG00000108306.13	*FBXL20*	787.54	0.62	0.11	5.388	7.1E-08	1.5E-05
ENSG00000116237.16	*ICMT*	1469.30	0.60	0.11	5.320	1E-07	2.2E-05
ENSG00000163814.8	*CDCP1*	601.07	0.72	0.14	5.316	1.1E-07	2.2E-05
ENSG00000173402.12	*DAG1*	464.04	0.82	0.15	5.305	1.1E-07	2.3E-05
ENSG00000204569.10	*PPP1R10*	910.35	0.59	0.11	5.263	1.4E-07	2.8E-05
ENSG00000162065.14	*TBC1D24*	741.04	0.76	0.15	5.255	1.5E-07	2.9E-05
ENSG00000143569.19	*UBAP2L*	1142.33	0.82	0.16	5.237	1.6E-07	3.2E-05
ENSG00000100147.14	*CCDC134*	260.76	0.82	0.16	5.220	1.8E-07	3.4E-05
ENSG00000126464.14	*PRR12*	200.86	1.05	0.20	5.202	2E-07	3.6E-05
ENSG00000172936.16	*MYD88*	396.24	0.69	0.13	5.204	1.9E-07	3.6E-05
ENSG00000183856.11	*IQGAP3*	173.27	1.07	0.21	5.178	2.2E-07	4E-05
ENSG00000161642.18	*ZNF385A*	1410.58	0.93	0.18	5.160	2.5E-07	4.4E-05
ENSG00000172992.12	*DCAKD*	296.02	0.77	0.15	5.147	2.7E-07	4.5E-05
ENSG00000140848.17	*CPNE2*	470.12	0.71	0.14	5.147	2.6E-07	4.5E-05
ENSG00000182446.14	*NPLOC4*	578.66	0.69	0.13	5.152	2.6E-07	4.5E-05
ENSG00000099864.18	*PALM*	281.50	0.75	0.15	5.140	2.7E-07	4.6E-05
ENSG00000277287.1	*AL109976.1*	91.82	1.70	0.33	5.133	2.9E-07	4.7E-05
ENSG00000132466.19	*ANKRD17*	1518.02	0.65	0.13	5.132	2.9E-07	4.7E-05
ENSG00000136828.19	*RALGPS1*	358.96	0.80	0.16	5.096	3.5E-07	5.6E-05
ENSG00000111676.15	*ATN1*	118.96	1.35	0.27	5.081	3.8E-07	6E-05
ENSG00000266028.7	*SRGAP2*	337.40	0.72	0.14	5.076	3.8E-07	6.1E-05
ENSG00000082397.18	*EPB41L3*	1560.81	0.78	0.16	5.035	4.8E-07	7.5E-05
ENSG00000280798.1	*LINC00294*	496.02	0.83	0.16	5.026	5E-07	7.7E-05
ENSG00000166987.15	*MBD6*	140.03	1.09	0.22	5.022	5.1E-07	7.8E-05
ENSG00000241973.11	*PI4KA*	313.35	0.75	0.15	5.019	5.2E-07	7.8E-05
ENSG00000110925.7	*CSRNP2*	588.56	0.61	0.12	4.957	7.2E-07	0.00011
ENSG00000117713.20	*ARID1A*	947.67	0.82	0.17	4.926	8.4E-07	0.00012
ENSG00000171786.6	*NHLH1*	927.22	0.70	0.14	4.909	9.2E-07	0.00013
ENSG00000105738.11	*SIPA1L3*	298.12	1.01	0.21	4.902	9.5E-07	0.00013
ENSG00000133065.11	*SLC41A1*	177.16	1.11	0.23	4.857	1.2E-06	0.00016
ENSG00000013288.9	*MAN2B2*	159.53	1.25	0.26	4.808	1.5E-06	0.0002
ENSG00000204138.13	*PHACTR4*	453.28	0.64	0.13	4.808	1.5E-06	0.0002
ENSG00000125648.15	*SLC25A23*	106.20	1.35	0.28	4.775	1.8E-06	0.00024
ENSG00000078900.15	*TP73*	488.06	0.68	0.14	4.764	1.9E-06	0.00025
ENSG00000213020.10	*ZNF611*	928.91	0.63	0.13	4.756	2E-06	0.00025
ENSG00000072071.16	*ADGRL1*	545.28	0.77	0.16	4.739	2.1E-06	0.00027
ENSG00000185900.11	*POMK*	933.94	0.73	0.16	4.733	2.2E-06	0.00028

ENSG00000164916.11	*FOXK1*	525.16	0.97	0.21	4.725	2.3E-06	0.00028
ENSG00000172081.14	*MOB3A*	133.72	1.45	0.31	4.715	2.4E-06	0.00029
ENSG00000182318.6	*ZSCAN22*	82.84	1.23	0.26	4.715	2.4E-06	0.00029
ENSG00000145113.22	*MUC4*	69.66	1.75	0.37	4.709	2.5E-06	0.00029
ENSG00000144043.12	*TEX261*	261.91	0.71	0.15	4.695	2.7E-06	0.00031
ENSG00000137166.17	*FOXP4*	309.70	1.00	0.21	4.693	2.7E-06	0.00031
ENSG00000205978.6	*NYNRIN*	260.06	0.87	0.19	4.683	2.8E-06	0.00033
ENSG00000196313.11	*POM121*	416.44	0.78	0.17	4.677	2.9E-06	0.00033
ENSG00000141905.19	*NFIC*	917.84	0.97	0.21	4.670	3E-06	0.00034
ENSG00000179134.15	*SAMD4B*	507.15	0.73	0.16	4.643	3.4E-06	0.00038
ENSG00000171435.14	*KSR2*	133.49	1.05	0.23	4.637	3.5E-06	0.00038
ENSG00000166887.16	*VPS39*	344.41	0.68	0.15	4.637	3.5E-06	0.00038
ENSG00000186472.20	*PCLO*	513.96	0.72	0.16	4.615	3.9E-06	0.00042
ENSG00000104812.15	*GYS1*	105.83	1.03	0.22	4.596	4.3E-06	0.00045
ENSG00000178385.15	*PLEKHM3*	293.77	0.73	0.16	4.595	4.3E-06	0.00045
ENSG00000229847.9	*EMX2OS*	253.04	0.70	0.15	4.596	4.3E-06	0.00045
ENSG00000204310.13	*AGPAT1*	478.64	0.61	0.13	4.596	4.3E-06	0.00045
ENSG00000135966.13	*TGFBRAP1*	385.88	0.72	0.16	4.585	4.5E-06	0.00047
ENSG00000196924.19	*FLNA*	287.60	0.78	0.17	4.573	4.8E-06	0.00049
ENSG00000116221.15	*MRPL37*	331.15	0.65	0.14	4.571	4.9E-06	0.00049
ENSG00000087258.16	*GNAO1*	474.83	0.81	0.18	4.564	5E-06	0.0005
ENSG00000173456.5	*RNF26*	213.71	0.73	0.16	4.561	5.1E-06	0.00051
ENSG00000175567.11	*UCP2*	299.46	0.68	0.15	4.452	8.5E-06	0.00083
ENSG00000155052.14	*CNTNAP5*	131.18	0.99	0.22	4.447	8.7E-06	0.00084
ENSG00000069424.15	*KCNAB2*	108.22	0.92	0.21	4.421	9.8E-06	0.00094
ENSG00000127481.15	*UBR4*	480.56	0.69	0.16	4.405	1.1E-05	0.001
ENSG00000116127.19	*ALMS1*	644.66	0.62	0.14	4.399	1.1E-05	0.00102
ENSG00000204463.13	*BAG6*	833.82	0.62	0.14	4.394	1.1E-05	0.00104
ENSG00000056277.16	*ZNF280C*	344.65	0.62	0.14	4.393	1.1E-05	0.00104
ENSG00000197122.12	*SRC*	178.01	1.03	0.23	4.388	1.1E-05	0.00105
ENSG00000174903.16	*RAB1B*	407.03	0.59	0.14	4.385	1.2E-05	0.00106
ENSG00000010803.16	*SCMH1*	263.90	0.70	0.16	4.382	1.2E-05	0.00107
ENSG00000188493.15	*C19orf54*	89.56	1.06	0.24	4.360	1.3E-05	0.00117
ENSG00000101464.11	*PIGU*	134.41	0.85	0.20	4.350	1.4E-05	0.00122
ENSG00000084731.15	*KIF3C*	158.97	1.32	0.30	4.343	1.4E-05	0.00124
ENSG00000132846.6	*ZBED3*	148.42	0.91	0.21	4.342	1.4E-05	0.00124
ENSG00000136854.24	*STXBP1*	1011.65	0.79	0.18	4.334	1.5E-05	0.00127
ENSG00000173801.17	*JUP*	571.35	0.59	0.14	4.333	1.5E-05	0.00127
ENSG00000141867.19	*BRD4*	252.21	0.76	0.18	4.318	1.6E-05	0.00134
ENSG00000251322.9	*SHANK3*	48.47	1.70	0.39	4.314	1.6E-05	0.00136
ENSG00000232098.3	*AC012313.1*	91.46	1.25	0.29	4.299	1.7E-05	0.00144
ENSG00000198911.12	*SREBF2*	1614.67	0.79	0.18	4.295	1.8E-05	0.00146
ENSG00000142319.18	*SLC6A3*	72.71	1.18	0.27	4.289	1.8E-05	0.00146
ENSG00000109066.14	*TMEM104*	116.94	0.98	0.23	4.289	1.8E-05	0.00146
ENSG00000204469.13	*PRRC2A*	1427.01	0.73	0.17	4.289	1.8E-05	0.00146
ENSG00000136504.13	*KAT7*	792.47	0.67	0.16	4.291	1.8E-05	0.00146
ENSG00000076513.17	*ANKRD13A*	589.87	0.60	0.14	4.274	1.9E-05	0.00154
ENSG00000065717.15	*TLE2*	151.87	0.85	0.20	4.246	2.2E-05	0.0017
ENSG00000086758.16	*HUWE1*	1032.94	0.75	0.18	4.243	2.2E-05	0.00172

ENSG00000149294.17	*NCAM1*	1120.73	0.65	0.15	4.235	2.3E-05	0.00176
ENSG00000115306.16	*SPTBN1*	408.82	0.62	0.15	4.236	2.3E-05	0.00176
ENSG00000169213.7	*RAB3B*	714.44	0.70	0.17	4.230	2.3E-05	0.00178
ENSG00000025800.14	*KPNA6*	1064.86	0.70	0.17	4.223	2.4E-05	0.00183
ENSG00000161082.13	*CELF5*	198.30	0.74	0.17	4.208	2.6E-05	0.00193
ENSG00000175662.18	*TOM1L2*	292.22	0.59	0.14	4.201	2.7E-05	0.00197
ENSG00000167861.16	*HID1*	112.59	0.97	0.23	4.188	2.8E-05	0.00207
ENSG00000227500.10	*SCAMP4*	106.57	0.87	0.21	4.176	3E-05	0.00217
ENSG00000161791.14	*FMNL3*	380.14	0.60	0.15	4.165	3.1E-05	0.00226
ENSG00000100350.15	*FOXRED2*	586.66	0.59	0.14	4.156	3.2E-05	0.00233
ENSG00000100605.17	*ITPK1*	368.01	0.68	0.16	4.143	3.4E-05	0.00245
ENSG00000166341.9	*DCHS1*	184.22	0.95	0.23	4.141	3.5E-05	0.00245
ENSG00000170653.19	*ATF7*	327.12	0.59	0.14	4.129	3.6E-05	0.00257
ENSG00000165238.16	*WNK2*	99.02	0.91	0.22	4.126	3.7E-05	0.00259
ENSG00000167972.14	*ABCA3*	71.27	1.07	0.26	4.124	3.7E-05	0.0026
ENSG00000124939.6	*SCGB2A1*	120.04	0.88	0.21	4.122	3.8E-05	0.00261
ENSG00000105245.9	*NUMBL*	287.95	0.61	0.15	4.118	3.8E-05	0.00263
ENSG00000118160.14	*SLC8A2*	88.25	1.18	0.29	4.115	3.9E-05	0.00266
ENSG00000105323.17	*HNRNPUL1*	1609.55	0.81	0.20	4.111	3.9E-05	0.00268
ENSG00000177728.17	*TMEM94*	121.45	0.81	0.20	4.111	3.9E-05	0.00268
ENSG00000178233.18	*TMEM151B*	257.08	0.79	0.19	4.079	4.5E-05	0.00304
ENSG00000087086.15	*FTL*	1170.17	0.61	0.15	4.067	4.8E-05	0.00316
ENSG00000072958.9	*AP1M1*	291.28	0.59	0.14	4.064	4.8E-05	0.00319
ENSG00000112511.18	*PHF1*	115.83	0.84	0.21	4.059	4.9E-05	0.00325
ENSG00000149091.15	*DGKZ*	227.07	0.66	0.16	4.029	5.6E-05	0.00364
ENSG00000136715.19	*SAP130*	310.16	0.86	0.22	4.015	5.9E-05	0.00383
ENSG00000089693.11	*MLF2*	324.36	0.62	0.15	4.013	6E-05	0.00386
ENSG00000203950.7	*RTL8A*	170.53	0.94	0.24	3.976	7E-05	0.00447
ENSG00000119242.9	*CCDC92*	128.80	0.81	0.20	3.973	7.1E-05	0.00449
ENSG00000088808.18	*PPP1R13B*	159.07	0.73	0.18	3.963	7.4E-05	0.00463
ENSG00000168763.16	*CNNM3*	147.29	0.76	0.19	3.919	8.9E-05	0.00546
ENSG00000167978.17	*SRRM2*	913.11	0.66	0.17	3.911	9.2E-05	0.0056
ENSG00000265763.4	*ZNF488*	52.99	1.13	0.29	3.908	9.3E-05	0.00563
ENSG00000114650.20	*SCAP*	157.52	0.72	0.18	3.905	9.4E-05	0.00567
ENSG00000258017.2	*AC011603.2*	134.96	0.94	0.24	3.901	9.6E-05	0.00572
ENSG00000139668.9	*WDFY2*	464.53	0.59	0.15	3.901	9.6E-05	0.00572
ENSG00000214078.13	*CPNE1*	188.35	0.69	0.18	3.894	9.9E-05	0.00584
ENSG00000147100.11	*SLC16A2*	193.34	0.79	0.20	3.889	0.0001	0.00594
ENSG00000123933.17	*MXD4*	200.03	0.63	0.16	3.870	0.00011	0.00634
ENSG00000163531.16	*NFASC*	452.60	0.77	0.20	3.865	0.00011	0.00646
ENSG00000055118.16	*KCNH2*	105.26	0.81	0.21	3.856	0.00012	0.00668
ENSG00000183580.10	*FBXL7*	86.88	1.13	0.29	3.850	0.00012	0.00676
ENSG00000144711.16	*IQSEC1*	156.86	0.71	0.18	3.851	0.00012	0.00676
ENSG00000105732.13	*ZNF574*	174.90	0.75	0.20	3.836	0.00013	0.00715
ENSG00000011347.10	*SYT7*	405.06	0.84	0.22	3.828	0.00013	0.00726
ENSG00000170145.5	*SIK2*	503.04	0.61	0.16	3.829	0.00013	0.00726
ENSG00000174943.11	*KCTD13*	123.47	0.92	0.24	3.818	0.00013	0.00747
ENSG00000132361.18	*CLUH*	133.61	0.73	0.19	3.820	0.00013	0.00747
ENSG00000099308.10	*MAST3*	126.01	0.77	0.20	3.813	0.00014	0.0076

ENSG00000175727.14	*MLXIP*	179.62	0.71	0.19	3.807	0.00014	0.00767
ENSG00000148337.21	*CIZ1*	185.47	0.63	0.17	3.809	0.00014	0.00767
ENSG00000140526.18	*ABHD2*	455.24	0.73	0.19	3.786	0.00015	0.00819
ENSG00000166482.12	*MFAP4*	216.95	0.69	0.18	3.782	0.00016	0.00827
ENSG00000140443.15	*IGF1R*	315.42	0.88	0.23	3.778	0.00016	0.00839
ENSG00000250986.1	*LINC02600*	292.62	0.71	0.19	3.776	0.00016	0.00842
ENSG00000197136.4	*PCNX3*	160.58	0.82	0.22	3.768	0.00016	0.00865
ENSG00000183778.17	*B3GALT5*	1222.02	0.67	0.18	3.765	0.00017	0.00872
ENSG00000165802.23	*NSMF*	202.26	0.64	0.17	3.759	0.00017	0.00885
ENSG00000110400.11	*NECTIN1*	192.27	0.78	0.21	3.745	0.00018	0.00918
ENSG00000131043.13	*AAR2*	215.60	0.64	0.17	3.747	0.00018	0.00918
ENSG00000134590.14	*RTL8C*	165.35	0.88	0.24	3.733	0.00019	0.00957
ENSG00000157216.16	*SSBP3*	500.83	0.63	0.17	3.731	0.00019	0.00958
ENSG00000197386.13	*HTT*	275.09	0.61	0.16	3.720	0.0002	0.00997
ENSG00000085644.14	*ZNF213*	80.02	1.05	0.28	3.713	0.0002	0.01017
ENSG00000103021.9	*CCDC113*	90.95	0.84	0.23	3.710	0.00021	0.01021
ENSG00000139641.13	*ESYT1*	293.37	0.67	0.18	3.710	0.00021	0.01021
ENSG00000164880.16	*INTS1*	132.89	0.91	0.25	3.686	0.00023	0.01113
ENSG00000116604.18	*MEF2D*	205.81	0.75	0.20	3.673	0.00024	0.01161
ENSG00000164061.5	*BSN*	243.76	0.72	0.20	3.673	0.00024	0.01161
ENSG00000105464.4	*GRIN2D*	182.89	0.82	0.22	3.672	0.00024	0.01162
ENSG00000160445.11	*ZER1*	169.43	0.71	0.19	3.667	0.00025	0.01175
ENSG00000178921.14	*PFAS*	172.35	0.72	0.20	3.649	0.00026	0.01257
ENSG00000132182.12	*NUP210*	574.56	0.61	0.17	3.632	0.00028	0.01329
ENSG00000166501.14	*PRKCB*	101.20	0.89	0.24	3.628	0.00029	0.01336
ENSG00000137216.19	*TMEM63B*	126.39	0.77	0.21	3.627	0.00029	0.01336
ENSG00000054523.18	*KIF1B*	839.98	0.61	0.17	3.625	0.00029	0.01342
ENSG00000178719.17	*GRINA*	79.37	0.89	0.25	3.609	0.00031	0.01418
ENSG00000183307.4	*TMEM121B*	255.32	0.70	0.19	3.604	0.00031	0.01438
ENSG00000167182.15	*SP2*	135.25	0.74	0.21	3.591	0.00033	0.01487
ENSG00000156299.13	*TIAM1*	461.65	0.68	0.19	3.569	0.00036	0.01596
ENSG00000140564.13	*FURIN*	182.70	0.74	0.21	3.567	0.00036	0.01599
ENSG00000137266.15	*SLC22A23*	79.00	1.04	0.29	3.554	0.00038	0.0168
ENSG00000164068.16	*RNF123*	93.43	0.82	0.23	3.544	0.00039	0.01734
ENSG00000173020.11	*GRK2*	180.22	0.71	0.20	3.542	0.0004	0.01745
ENSG00000169710.9	*FASN*	604.07	0.97	0.28	3.531	0.00041	0.01802
ENSG00000185504.17	*FAAP100*	141.04	0.68	0.19	3.523	0.00043	0.01854
ENSG00000169783.13	*LINGO1*	166.68	0.97	0.28	3.517	0.00044	0.01891
ENSG00000166833.22	*NAV2*	727.65	0.77	0.22	3.513	0.00044	0.01909
ENSG00000105427.10	*CNFN*	22.83	1.80	0.51	3.510	0.00045	0.01927
ENSG00000189114.8	*BLOC1S3*	99.35	0.94	0.27	3.508	0.00045	0.01934
ENSG00000186111.11	*PIP5K1C*	207.95	0.66	0.19	3.506	0.00046	0.01946
ENSG00000205336.14	*ADGRG1*	84.03	1.07	0.30	3.503	0.00046	0.01957
ENSG00000102302.8	*FGD1*	97.67	0.77	0.22	3.499	0.00047	0.01981
ENSG00000175115.13	*PACS1*	136.66	0.77	0.22	3.485	0.00049	0.02058
ENSG00000131094.4	*C1QL1*	108.58	0.77	0.22	3.482	0.0005	0.02073
ENSG00000183682.8	*BMP8A*	104.52	0.79	0.23	3.478	0.00051	0.02095
ENSG00000019485.13	*PRDM11*	115.87	0.71	0.20	3.459	0.00054	0.02224
ENSG00000186185.14	*KIF18B*	173.65	0.67	0.19	3.458	0.00054	0.02226

ENSG00000286176.2	*AC116317.1*	106.37	0.92	0.27	3.449	0.00056	0.02298
ENSG00000053702.15	*NRIP2*	147.54	0.67	0.19	3.443	0.00058	0.02336
ENSG00000007047.15	*MARK4*	97.28	0.81	0.24	3.440	0.00058	0.02346
ENSG00000084774.14	*CAD*	156.13	0.68	0.20	3.440	0.00058	0.02346
ENSG00000172053.18	*QARS1*	230.11	0.59	0.17	3.438	0.00058	0.0235
ENSG00000101255.11	*TRIB3*	287.51	0.84	0.24	3.437	0.00059	0.02356
ENSG00000172269.19	*DPAGT1*	60.08	0.92	0.27	3.431	0.0006	0.02384
ENSG00000157933.10	*SKI*	267.16	0.85	0.25	3.429	0.00061	0.02391
ENSG00000066322.15	*ELOVL1*	106.52	0.72	0.21	3.427	0.00061	0.02403
ENSG00000061337.15	*LZTS1*	326.75	0.73	0.21	3.420	0.00063	0.02437
ENSG00000111249.14	*CUX2*	188.75	0.64	0.19	3.420	0.00063	0.02437
ENSG00000129993.15	*CBFA2T3*	426.08	0.77	0.22	3.407	0.00066	0.02527
ENSG00000131759.18	*RARA*	95.24	0.95	0.28	3.405	0.00066	0.02532
ENSG00000084628.10	*NKAIN1*	38.42	1.15	0.34	3.401	0.00067	0.02559
ENSG00000105613.10	*MAST1*	119.90	0.68	0.20	3.384	0.00071	0.0269
ENSG00000096433.11	*ITPR3*	163.37	0.70	0.21	3.375	0.00074	0.0277
ENSG00000107331.17	*ABCA2*	53.19	1.05	0.31	3.370	0.00075	0.02805
ENSG00000125505.17	*MBOAT7*	168.97	0.61	0.18	3.361	0.00078	0.02871
ENSG00000116128.11	*BCL9*	128.32	0.85	0.25	3.357	0.00079	0.02905
ENSG00000223501.9	*VPS52*	357.01	0.59	0.17	3.351	0.00081	0.0296
ENSG00000228594.4	*FNDC10*	66.00	0.91	0.27	3.324	0.00089	0.03206
ENSG00000100228.13	*RAB36*	176.46	0.64	0.19	3.320	0.0009	0.03232
ENSG00000149328.15	*GLB1L2*	117.96	0.75	0.22	3.318	0.00091	0.0324
ENSG00000185189.18	*NRBP2*	105.98	0.84	0.25	3.308	0.00094	0.0333
ENSG00000023191.17	*RNH1*	204.09	0.65	0.20	3.302	0.00096	0.03401
ENSG00000159363.19	*ATP13A2*	103.77	0.71	0.22	3.290	0.001	0.03528
ENSG00000267534.4	*S1PR2*	71.55	0.89	0.27	3.277	0.00105	0.03679
ENSG00000185621.11	*LMLN*	81.10	0.85	0.26	3.274	0.00106	0.03697
ENSG00000158467.16	*AHCYL2*	98.60	0.80	0.24	3.265	0.0011	0.03802
ENSG00000111640.15	*GAPDH*	5390.18	0.65	0.20	3.236	0.00121	0.04142
ENSG00000141574.8	*SECTM1*	44.38	1.19	0.37	3.229	0.00124	0.04238
ENSG00000136944.19	*LMX1B*	27.16	1.49	0.46	3.208	0.00134	0.04481
ENSG00000162706.13	*CADM3*	252.78	0.73	0.23	3.206	0.00135	0.04501
ENSG00000183751.15	*TBL3*	65.15	0.93	0.29	3.203	0.00136	0.04535
ENSG00000099797.15	*TECR*	301.81	0.59	0.18	3.185	0.00145	0.04728
ENSG00000175221.15	*MED16*	110.81	0.73	0.23	3.184	0.00145	0.04728
ENSG00000072110.15	*ACTN1*	270.22	0.63	0.20	3.168	0.00154	0.04896
ENSG00000089159.17	*PXN*	35.20	1.16	0.37	3.165	0.00155	0.04911

[1]baseMean—The average of the normalized count values, dividing by size factors, taken over all samples.
[2]lfcSE–The standard error estimate for the log2 fold change estimate.
[3]stat–The value of the test statistic for the gene or transcript.

Supplementary Table S3. Gene Ontology analysis of IGF2BP3 targets based on biological

Term	Genes	Count	%	List Total[1]	Pop Hits[2]	Pop Total[3]	Fold Enrichment[3]	*P-v* alue
GO:0072659~protein localization to plasma membrane	*RAB3B, NFASC, JUP, PACS1, STXBP1, EPB41L3, PALM, FLNA, DCHS1, SCN3B, SPTBN1, RAB8A*	12	4.36	253	162	19414	5.68	9.0E-06
GO:0035556~intracellular signal transduction	*SHC1, PRKCB, MAST3, SRC, MAST1, ADCY1, KSR2, DGKZ, NRBP2, ADCY9, WNK2, DVL3, SIK2, MARK4, RALGPS1*	15	5.45	253	459	19414	2.51	2.8E-03
GO:0030036~actin cytoskeleton organization	*FMNL3, SHC1, IQSEC1, ACTN1, FLNA, PIP5K1C, FGD1, SPTBN1, PHACTR4*	9	3.27	253	189	19414	3.65	3.4E-03
GO:0046847~filopodium assembly	*FMNL3, S1PR2, SRGAP2, FGD1*	4	1.45	253	24	19414	12.79	3.6E-03
GO:0007010~cytoskeleton organization	*FMNL3, PCLO, MAST3, EPB41L3, MAST1, PALM, SIPA1L3, FGD1*	8	2.91	253	156	19414	3.94	4.3E-03
GO:0043123~positive regulation of I-kappaB kinase/NF-kappaB signaling	*ANKRD17, PRKCB, SHISA5, TRIM14, FLNA, SECTM1, GAPDH, MYD88, BRD4*	9	3.27	253	199	19414	3.47	4.6E-03
GO:0045893~positive regulation of transcription, DNA-templated	*KCNH2, SHC1, PRKCB, NCOA6, SRC, ACTN1, HMGA1, ATN1, FOXK1, ARID1A, MED16, PRDM11, NFIC, RARA, DVL3, TRIM14, KAT7, BRD4, TP73*	19	6.91	253	724	19414	2.01	6.7E-03

GO:2000463~positive regulation of excitatory postsynaptic potential	*CUX2, TBC1D24, SHANK3, GRIN2D*	4	1.45	253	30	19414	10.23	6.8E-03
GO:0090557~establishment of endothelial intestinal barrier	*PTPRS, FASN, MYD88*	3	1.09	253	11	19414	20.93	8.5E-03
GO:0035542~regulation of SNARE complex assembly	*STXBP1, TGFBRAP1, VPS39*	3	1.09	253	11	19414	20.93	8.5E-03
GO:0045944~positive regulation of transcription from RNA polymerase II promoter	*CSRNP2, CRTC3, ZMIZ1, NHLH1, ZMIZ2, KPNA6, DVL3, KAT7, MEF2D, ZBED3, BRD4, MLXIP, JUP, NCOA6, HMGA1, SOX12, SREBF2, SKI, BCL9, NFIC, RARA, ATF7, LMX1B, SSBP3, MYD88, TP73, ZNF574*	27	9.82	253	1222	19414	1.70	9.1E-03
GO:0016477~cell migration	*FMNL3, TIAM1, ADGRG1, RNH1, JUP, PXN, SDC3, ATN1, KCTD13, DGKZ*	10	3.64	253	275	19414	2.79	1.0E-02
GO:0007179~transforming growth factor beta receptor signaling pathway	*SKI, SRC, ZMIZ1, TGFBRAP1, PXN, FURIN*	6	2.18	253	101	19414	4.56	1.0E-02
GO:0045815~positive regulation of gene expression, epigenetic	*ZMIZ1, ZMIZ2, KAT7, ARID1A*	4	1.45	253	41	19414	7.49	1.6E-02

GO:0051056~regulation of small GTPase mediated signal transduction	*TIAM1, ARHGAP1, TSC2, SRGAP2, SIPA1L3, FGD1*	6	2.18	253	119	19414	3.87	2.0E-02
GO:0007420~brain development	*BAG6, ADGRG1, NFASC, QARS1, NCOA6, MAST1, POMK, ADCY1, GRIN2D*	9	3.27	253	261	19414	2.65	2.1E-02
GO:0006468~protein phosphorylation	*PRKCB, SRC, MAST1, KSR2, IQGAP3, NRBP2, GRK2, WNK2, POMK, RARA, TRIB3, SIK2, MARK4, CDK16*	14	5.09	253	536	19414	2.00	2.3E-02
GO:0007626~locomotory behavior	*ABCA2, NAV2, SLC6A3, ZNF385A, C1QL1*	5	1.82	253	86	19414	4.46	2.6E-02
GO:0099526~presynapse to nucleus signaling pathway	*PCLO, BSN*	2	0.73	253	2	19414	76.74	2.6E-02
GO:0021591~ventricular system development	*CCDC134, MBOAT7, CELSR2*	3	1.09	253	20	19414	11.51	2.7E-02
GO:0071277~cellular response to calcium ion	*CPNE1, CPNE2, ADCY1, SLC25A23, SYT7*	5	1.82	253	90	19414	4.26	3.0E-02
GO:0007030~Golgi organization	*NPLOC4, UBXN2B, RAB1B, HUWE1, HTT, RAB8A*	6	2.18	253	133	19414	3.46	3.0E-02
GO:0007157~heterophilic cell-cell adhesion via plasma membrane cell adhesion molecules	*CADM3, ADGRL1, DCHS1, NECTIN1*	4	1.45	253	54	19414	5.68	3.3E-02

GO:0016192~vesicle-mediated transport	*RAB36, STXBP1, CPNE1, TSC2, KIF1B, SYT7, VPS39, AP1M1*	8	2.91	253	235	19414	2.61	3.4E-02
GO:0030521~androgen receptor signaling pathway	*ZMIZ1, SCGB2A1, ARID1A*	3	1.09	253	23	19414	10.01	3.5E-02
GO:0006541~glutamine metabolic process	*CAD, UCP2, PFAS*	3	1.09	253	24	19414	9.59	3.8E-02
GO:1904071~presynaptic active zone assembly	*PCLO, BSN*	2	0.73	253	3	19414	51.16	3.8E-02
GO:0098609~cell-cell adhesion	*NFASC, JUP, SHC1, SRC, PIP5K1C, CELSR2, NECTIN1*	7	2.55	253	195	19414	2.75	4.2E-02
GO:0050808~synapse organization	*NFASC, PTPRS, FLNA, SLC8A2*	4	1.45	253	60	19414	5.12	4.3E-02
GO:0045892~negative regulation of transcription, DNA-templated	*TLE2, CBX5, SHC1, SRC, HMGA1, FOXK1, PRDM11, CBFA2T3, QARS1, SCMH1, RARA, TRIB3, KAT7, ZNF488*	14	5.09	253	588	19414	1.83	4.4E-02
GO:0048714~positive regulation of oligodendrocyte differentiation	*DAG1, ZNF488, TP73*	3	1.09	253	26	19414	8.85	4.4E-02
GO:0007399~nervous system development	*NUMBL, ADGRG1, DPYSL5, TPP1, NAV1, NAV2, ARID1A, SRGAP2, SCN3B, MARK4, MEF2D*	11	4.00	253	418	19414	2.02	4.7E-02
GO:0009410~response to xenobiotic stimulus	*ABCA2, SRC, ABCA3, BLOC1S3, ADCY1, PFAS, SLC6A3, TP73*	8	2.91	253	253	19414	2.43	4.8E-02

GO:0006606~protein import into nucleus	*NUP214, POM121, PPP1R10, KPNA6, TSC2*	5	1.82	253	105	19414	3.65	4.8E-02
GO:0060291~long-term synaptic potentiation	*ITPR3, SHANK3, GRIN2D, SLC8A2*	4	1.45	253	63	19414	4.87	4.9E-02

[1]List tota refers to the total number of genes analyzed.

[2]Pop hits are the genes in the list that are annotated with a specific GO term.

[3]Pop total is the total number of genes in the reference population annotated with that GO term.

[4]Fold enrichment indicates how much more (or less) a specific GO term is represented in the list of genes compared to the reference population

Supplementary Table S4. KEGG analysis of IGF2BP3 target genes

Term	Genes	Count	%	List Total[1]	Pop Hits[2]	Pop Total[3]	Fold Enrichment[4]	*P* -value
hsa04724:Glutamatergic synapse	*GNAO1, ADCY9, GRK2, PRKCB, ITPR3, ADCY1, SHANK3, GRIN2D*	8	2.91	109	115	8465	5.40	6.4E-04
hsa04540:Gap junction	*TUBA1B, ADCY9, SRC, PRKCB, GNA11, ITPR3, ADCY1*	7	2.55	109	88	8465	6.18	8.7E-04
hsa04730:Long-term depression	*GNAO1, PRKCB, PPP2R1A, GNA11, ITPR3, IGF1R*	6	2.18	109	60	8465	7.77	9.6E-04
hsa04911:Insulin secretion	*ADCY9, PCLO, PRKCB, GNA11, ITPR3, ADCY1*	6	2.18	109	86	8465	5.42	4.7E-03
hsa04912:GnRH signaling pathway	*ADCY9, SRC, PRKCB, GNA11, ITPR3, ADCY1*	6	2.18	109	93	8465	5.01	6.6E-03
hsa04713:Circadian entrainment	*GNAO1, ADCY9, PRKCB, ITPR3, ADCY1, GRIN2D*	6	2.18	109	97	8465	4.80	7.8E-03
hsa04070:Phosphatidylinositol signaling system	*PRKCB, ITPK1, PI4KA, ITPR3, PIP5K1C, DGKZ*	6	2.18	109	97	8465	4.80	7.8E-03
hsa05163:Human cytomegalovirus infection	*GNAO1, ADCY9, SRC, PRKCB, GNA11, PXN, TSC2, ITPR3, ADCY1*	9	3.27	109	225	8465	3.11	7.9E-03
hsa04915:Estrogen signaling pathway	*GNAO1, ADCY9, SHC1, SRC, RARA, ITPR3, ADCY1*	7	2.55	109	138	8465	3.94	8.3E-03
hsa04062:Chemokine signaling pathway	*TIAM1, ADCY9, GRK2, SHC1, SRC, PRKCB, PXN, ADCY1*	8	2.91	109	192	8465	3.24	1.1E-02
hsa04928:Parathyroid hormone synthesis, secretion and action	*ADCY9, PRKCB, GNA11, ITPR3, ADCY1, MEF2D*	6	2.18	109	106	8465	4.40	1.1E-02

hsa04072:Phospholipase D signaling pathway	*ADCY9, SHC1, TSC2, PIP5K1C, ADCY1, AGPAT1, DGKZ*	7	2.55	109	148	8465	3.67	1.1E-02
hsa04725:Cholinergic synapse	*GNAO1, ADCY9, PRKCB, GNA11, ITPR3, ADCY1*	6	2.18	109	113	8465	4.12	1.5E-02
hsa04510:Focal adhesion	*SHC1, SRC, PRKCB, ACTN1, PXN, FLNA, PIP5K1C, IGF1R*	8	2.91	109	203	8465	3.06	1.5E-02
hsa04015:Rap1 signaling pathway	*GNAO1, TIAM1, ADCY9, SRC, PRKCB, ADCY1, SIPA1L3, IGF1R*	8	2.91	109	210	8465	2.96	1.8E-02
hsa04935:Growth hormone synthesis, secretion and action	*ADCY9, SHC1, PRKCB, GNA11, ITPR3, ADCY1*	6	2.18	109	120	8465	3.88	1.8E-02
hsa04152:AMPK signaling pathway	*GYS1, PPP2R1A, FASN, TSC2, RAB8A, IGF1R*	6	2.18	109	121	8465	3.85	1.9E-02
hsa04727:GABAergic synapse	*GNAO1, ADCY9, SRC, PRKCB, ADCY1*	5	1.82	109	89	8465	4.36	2.7E-02
hsa05032:Morphine addiction	*GNAO1, ADCY9, GRK2, PRKCB, ADCY1*	5	1.82	109	91	8465	4.27	2.9E-02
hsa01522:Endocrine resistance	*ADCY9, SHC1, SRC, ADCY1, IGF1R*	5	1.82	109	98	8465	3.96	3.6E-02
hsa04925:Aldosterone synthesis and secretion	*ADCY9, PRKCB, GNA11, ITPR3, ADCY1*	5	1.82	109	98	8465	3.96	3.6E-02
hsa04750:Inflammatory mediator regulation of TRP channels	*ADCY9, SRC, PRKCB, ITPR3, ADCY1*	5	1.82	109	98	8465	3.96	3.6E-02
hsa04916:Melanogenesis	*GNAO1, ADCY9, PRKCB, DVL3, ADCY1*	5	1.82	109	101	8465	3.84	4.0E-02
hsa05142:Chagas disease	*GNAO1, PPP2R1A, GNA11, ADCY1, MYD88*	5	1.82	109	102	8465	3.81	4.1E-02

hsa04972:Pancreatic secretion	*ADCY9, PRKCB, ITPR3, ADCY1, RAB8A*	5	1.82	109	102	8465	3.81	4.1E-02
hsa04921:Oxytocin signaling pathway	*GNAO1, ADCY9, SRC, PRKCB, ITPR3, ADCY1*	6	2.18	109	154	8465	3.03	4.7E-02
hsa05205:Proteoglycans in cancer	*TIAM1, SRC, PRKCB, PXN, FLNA, ITPR3, IGF1R*	7	2.55	109	205	8465	2.65	4.7E-02
hsa04927:Cortisol synthesis and secretion	*ADCY9, GNA11, ITPR3, ADCY1*	4	1.45	109	65	8465	4.78	5.0E-02

[1]List tota refers to the total number of genes analyzed.
[2]Pop hits are the genes in the list that are annotated with a specific GO term.
[3]Pop total is the total number of genes in the reference population annotated with that GO term.
[4]Fold enrichment indicates how much more (or less) a specific GO term is represented in the list of genes compared to the reference population

Supplementary Table S5. Differentially expressed genes between MCC primay tumors and metastases in the MCC GEO cohort GSE22396 (cut off p<0.05 & fold change >=1.5)

ID	Gene symbol	Gene title	logFC	*P*-Value
Over-expressed genes in Metastases vs. Primary MCC tumors				
100142686_TGI_at	*LZTS1-AS1*	LZTS1 antisense RNA 1	0.852	0.0006
100144165_TGI_at	*NECTIN2*	nectin cell adhesion molecule 2	0.713	0.0009
100134334_TGI_at	*NAV1*	SH3 and cysteine rich domain 3	0.898	0.0020
100303253_TGI_at	*CDC42BPB*	nectin cell adhesion molecule 2	0.569	0.0021
100122396_TGI_at	*HBQ1*	neuron navigator 1	0.629	0.0023
100134264_TGI_at	*GRIA4*	neuron navigator 1	0.599	0.0023
100126999_TGI_at	*PPIH*	CDC42 binding protein kinase beta	0.258	0.0025
100145614_TGI_at	*CHRM2*	cullin 2	0.530	0.0034
100304072_TGI_at	*TMEM51*	5'-nucleotidase, cytosolic II	0.364	0.0038
100133785_TGI_at	*SEL1L2*	WW domain containing adaptor with coiled-coil	0.616	0.0043
100147927_TGI_at	*MME*	transmembrane protein 51	0.472	0.0045
100126424_TGI_at	*CRB2*	solute carrier family 30 member 7	0.306	0.0049
100146052_TGI_at	*ANKH*	membrane metallo-endopeptidase	1.256	0.0050
100128942_TGI_at	*STX1B*	signal transducer and activator of transcription 3	0.425	0.0050
100308987_TGI_at	*FLCN*	ANKH inorganic pyrophosphate transport regulator	0.746	0.0051
100124310_TGI_at	*GRIP1*	potassium channel tetramerization domain containing 14	1.588	0.0052
100135396_TGI_at	*MEF2D*	folliculin	0.364	0.0054
100311515_TGI_at	*SP8*	transmembrane protein 51	0.517	0.0055
100127769_TGI_at	*TCL6*	PHD finger protein 12	0.428	0.0056
100143443_TGI_at	*KIAA1644*	peptidyl arginine deiminase 2	1.244	0.0057
100137745_TGI_at	*LRRC6*	myocyte enhancer factor 2D	0.411	0.0057
100149836_TGI_at	*CACFD1*	neuron navigator 1	0.602	0.0065
100147451_TGI_at	*LOC100130744*	leucine zipper tumor suppressor 1	0.752	0.0071
100307628_TGI_at	*SFTA1P*	family with sequence similarity 222 member B	0.243	0.0076
100138941_TGI_at	*CBLN1*	calcium channel flower domain containing 1	0.539	0.0076
100308083_TGI_at	*ZNF488*	ANKH inorganic pyrophosphate transport regulator	0.377	0.0079
100152317_TGI_at	*TBR1*	fibrosin	0.223	0.0080
100313252_TGI_at	*NAPA*	neuron navigator 1	0.599	0.0085
100154926_TGI_at	*LINC01372*	surfactant associated 1, pseudogene	0.528	0.0086
100301133_TGI_at	*GPRC5B*	zinc finger protein 488	0.667	0.0089
100133620_TGI_at	*FCER2*	BTB domain containing 9	0.364	0.0091
100133885_TGI_at	*SOCS2-AS1*	NSF attachment protein alpha	0.356	0.0094
100135004_TGI_at	*RPL35*	long intergenic non-protein coding RNA 1372	1.040	0.0097
100150788_TGI_at	*PCDH15*	tetraspanin 14	0.436	0.0099
100144335_TGI_at	*STK24*	uncharacterized LOC338620	0.283	0.0100

100308839_TGI_at	*AZIN1*	G protein-coupled receptor class C group 5 member B	0.579	0.0101
100153314_TGI_at	*CD19*	SOCS2 antisense RNA 1	0.755	0.0107
100306086_TGI_at	*NOM1*	fibroblast growth factor receptor substrate 2	0.381	0.0111
100304251_TGI_at	*TTPAL*	tetraspanin 14	0.413	0.0114
100160157_TGI_at	*UBXN10*	serine/threonine kinase 24	0.456	0.0114
100158767_TGI_at	*MLF1*	CTAGE family member 5, ER export factor	0.620	0.0116
100121741_TGI_at	*RXRG*	G protein-coupled receptor class C group 5 member B	0.491	0.0117
100134461_TGI_at	*GIT2*	RALBP1 associated Eps domain containing 2	0.237	0.0118
100300387_TGI_at	*C3orf35*	membrane metallo-endopeptidase	1.465	0.0119
100129222_TGI_at	*USP9X*	pleckstrin homology, MyTH4 and FERM domain containing H1	0.673	0.0120
100129202_TGI_at	*COBL*	NRDE-2, necessary for RNA interference, domain containing	0.365	0.0123
100124155_TGI_at	*WDR20*	zinc finger and BTB domain containing 3	0.413	0.0131
100137162_TGI_at	*RAB6A*	GIT ArfGAP 2	0.342	0.0133
100307607_TGI_at	*ERO1B*	golgi membrane protein 1	0.467	0.0135
100307279_TGI_at	*GLRA3*	ubiquitin specific peptidase 9, X-linked	0.362	0.0137
100149516_TGI_at	*SLC4A10*	quiescin sulfhydryl oxidase 1	0.844	0.0137
100142677_TGI_at	*MYPOP*	LIM domain only 4	0.298	0.0140
100159824_TGI_at	*ZNF827*	neuron navigator 1	0.574	0.0142
100130832_TGI_at	*NAA60*	meiosis 1 associated protein	0.622	0.0147
100311288_TGI_at	*PDZD9*	WD repeat domain 20	0.244	0.0148
100152462_TGI_at	*CLTC*	solute carrier family 6 member 6	0.656	0.0149
100309263_TGI_at	*DOCK9-AS2*	RAB6A, member RAS oncogene family	1.004	0.0149
100312200_TGI_at	*BTN1A1*	endoplasmic reticulum oxidoreductase 1 beta	0.842	0.0152
100130944_TGI_at	*DNAJB12*	trans-golgi network protein 2	0.217	0.0153
100125706_TGI_at	*COA1*	thrombospondin 4	1.191	0.0156
100140256_TGI_at	*NPHP3*	Myb related transcription factor, partner of profilin	0.356	0.0157
100152369_TGI_at	*ST8SIA2*	zinc finger protein 827	0.572	0.0160
100301738_TGI_at	*GCG*	potassium channel tetramerization domain containing 14	1.329	0.0163
100143511_TGI_at	*STARD3NL*	tetratricopeptide repeat domain 8	0.423	0.0163
100124252_TGI_at	*DAZAP2*	nuclear factor I C	0.334	0.0164
100124903_TGI_at	*ARIH2*	N(alpha)-acetyltransferase 60, NatF catalytic subunit	0.280	0.0167
100309810_TGI_at	*BPHL*	PDZ domain containing 9	0.325	0.0169
100309361_TGI_at	*TAOK2*	clathrin heavy chain	0.267	0.0170
100127140_TGI_at	*OSBPL10*	DOCK9 antisense RNA 2 (head to head)	0.554	0.0170
100159196_TGI_at	*RHOBTB1*	DnaJ heat shock protein family (Hsp40) member B12	0.350	0.0173
100311546_TGI_at	*HOXA2*	chromosome X open reading frame 23	0.289	0.0178

100142706_TGI_at	*HIST1H3E*	matrix metallopeptidase 15	0.533	0.0178
100137846_TGI_at	*CELSR2*	double PHD fingers 2	0.329	0.0180
100148052_TGI_at	*RADIL*	ST3 beta-galactoside alpha-2,3-sialyltransferase 4	0.537	0.0183
100134927_TGI_at	*SERPINE1*	DAZ associated protein 2	0.348	0.0184
100163165_TGI_at	*IFT43*	solute carrier family 4 member 4	1.366	0.0184
100145653_TGI_at	*C11orf70*	TAO kinase 2	0.366	0.0190
100144779_TGI_at	*NR1I3*	sulfite oxidase	0.362	0.0191
100138674_TGI_at	*TRIM8*	ANKH inorganic pyrophosphate transport regulator	0.607	0.0191
100149999_TGI_at	*LOC200830*	Rho related BTB domain containing 1	0.643	0.0191
100308545_TGI_at	*MIGA1*	mitochondrial pyruvate carrier 2	0.490	0.0196
100121798_TGI_at	*PDE7B*	histone cluster 1, H3e	0.303	0.0197
100141788_TGI_at	*PABPC1L2B*	family with sequence similarity 222 member B	0.247	0.0199
100146487_TGI_at	*PTHLH*	cadherin EGF LAG seven-pass G-type receptor 2	0.409	0.0199
100127351_TGI_at	*PAPOLA*	long intergenic non-protein coding RNA 1048	0.847	0.0199
100301959_TGI_at	*SGCB*	WW domain containing adaptor with coiled-coil	0.279	0.0200
100309692_TGI_at	*TNKS2*	UDP-GlcNAc:betaGal beta-1,3-N-acetylglucosaminyltransferase 2	0.303	0.0202
100305951_TGI_at	*IPO11*	MIR4458 host gene	1.430	0.0202
100127490_TGI_at	*DCUN1D1*	potassium channel tetramerization domain containing 5	0.273	0.0204
100159705_TGI_at	*CLP1*	solute carrier family 4 member 4	1.172	0.0206
100307316_TGI_at	*PCDHGC3*	gastric cancer associated transcript 2 (non-protein coding)	0.313	0.0208
100303863_TGI_at	*POU4F2*	tripartite motif containing 8	0.290	0.0213
100302990_TGI_at	*FAM102A*	ATPase H+ transporting V0 subunit c	0.286	0.0214
100125220_TGI_at	*SSPO*	uncharacterized LOC200830	0.371	0.0214
100131355_TGI_at	*MAX*	family with sequence similarity 13 member C	0.517	0.0218
100141869_TGI_at	*ARID1A*	Wnt family member 2	0.700	0.0218
100129780_TGI_at	*NRAV*	mitoguardin 1	0.372	0.0224
100136062_TGI_at	*ELMSAN1*	phosphodiesterase 7B	0.714	0.0225
100162265_TGI_at	*EPYC*	tripartite motif containing 8	0.274	0.0226
100300809_TGI_at	*SEMA3A*	parathyroid hormone like hormone	1.129	0.0227
100304256_TGI_at	*LOC642852*	syntaxin 6	0.268	0.0228
100156030_TGI_at	*RCAN2*	chromosome 6 open reading frame 89	0.244	0.0232
100153594_TGI_at	*PNMA2*	tankyrase 2	0.394	0.0233
100148210_TGI_at	*ACP2*	interleukin 1 receptor like 1	0.279	0.0233
100128789_TGI_at	*AATBC*	Sp2 transcription factor	0.392	0.0234
100121882_TGI_at	*FAM20A*	src-related kinase lacking C-terminal regulatory tyrosine and N-terminal myristylation sites	0.248	0.0235
100162894_TGI_at	*MCCC2*	MIR4458 host gene	1.029	0.0237
100140576_TGI_at	*FCRL6*	WAS/WASL interacting protein family member 2	0.335	0.0238

100301513_TGI_at	*ADH5*	protocadherin gamma subfamily C, 3	0.304	0.0238
100159642_TGI_at	*SLC27A5*	family with sequence similarity 102 member A	0.512	0.0242
100311314_TGI_at	*FTH1*	transmembrane protein 127	0.362	0.0243
100152982_TGI_at	*ZMIZ2*	human immunodeficiency virus type I enhancer binding protein 1	0.325	0.0245
100313904_TGI_at	*METTL6*	spectrin beta, erythrocytic	0.465	0.0245
100310160_TGI_at	*NPY5R*	MYC associated factor X	0.302	0.0246
100310977_TGI_at	*CDH6*	uncharacterized LOC441178	0.409	0.0246
100134931_TGI_at	*ZNF568*	AT-rich interaction domain 1A	0.337	0.0246
100311268_TGI_at	*TOMM5*	elongation factor for RNA polymerase II	0.199	0.0246
100150067_TGI_at	*LOC285500*	endoplasmic reticulum oxidoreductase 1 beta	0.763	0.0246
100133834_TGI_at	*MED15*	early B-cell factor 2	0.658	0.0247
100130510_TGI_at	*PRR34*	RNA binding motif protein 26	0.451	0.0248
100150877_TGI_at	*ARHGAP31*	ELM2 and Myb/SANT domain containing 1	0.369	0.0249
100147267_TGI_at	*COL4A6*	PCF11 cleavage and polyadenylation factor subunit	0.222	0.0249
100128694_TGI_at	*FOXP1*	epiphycan	1.359	0.0254
100122379_TGI_at	*MANF*	uncharacterized LOC642852	0.199	0.0255
100155585_TGI_at	*LARP4B*	zinc finger AN1-type containing 3	0.247	0.0255
100157034_TGI_at	*OR7G3*	long intergenic non-protein coding RNA 473	0.507	0.0255
100132313_TGI_at	*SLC39A7*	kelch like family member 41	1.379	0.0257
100135207_TGI_at	*CRHBP*	acid phosphatase 2, lysosomal	0.421	0.0258
100312306_TGI_at	*MAU2*	membrane metallo-endopeptidase	1.405	0.0258
100130924_TGI_at	*BBS7*	apoptosis associated transcript in bladder cancer	0.839	0.0261
100138713_TGI_at	*GOLGA5*	family with sequence similarity 20 member A	0.456	0.0262
100160925_TGI_at	*LSM1*	potassium voltage-gated channel subfamily E regulatory subunit 1	0.526	0.0263
100136914_TGI_at	*MED18*	zinc finger FYVE-type containing 1	0.229	0.0264
100158484_TGI_at	*NME7*	Fc receptor like 6	0.253	0.0265
100146503_TGI_at	*LOC100131510*	DMRT like family A2	1.297	0.0265
100311443_TGI_at	*POLR3D*	capping protein regulator and myosin 1 linker 1	0.469	0.0266
100161355_TGI_at	*AGRN*	UDP-GlcNAc:betaGal beta-1,3-N-acetylglucosaminyltransferase 2	0.288	0.0267
100124855_TGI_at	*DAO*	cytohesin 1	0.338	0.0267
100149346_TGI_at	*CBX3P2*	suppressor of cytokine signaling 2	0.591	0.0268
100309912_TGI_at	*ZBTB43*	ferritin heavy chain 1	0.267	0.0272
100132714_TGI_at	*HAPLN4*	CTAGE family member 5, ER export factor	0.586	0.0273
100309089_TGI_at	*KIAA1143*	zinc finger MIZ-type containing 2	0.328	0.0273
100156242_TGI_at	*MMP8*	cadherin 6	0.759	0.0275
100124061_TGI_at	*MUC1*	zinc finger protein 568	0.650	0.0275
100142488_TGI_at	*IGSF11*	mediator complex subunit 15	0.229	0.0277
100139620_TGI_at	*FAM234B*	proline rich 34	0.278	0.0279

100158035_TGI_at	*NFIX*	Rho GTPase activating protein 31	0.500	0.0280
100136497_TGI_at	*ERCC8*	polypeptide N-acetylgalactosaminyltransferase 5	0.717	0.0282
100309027_TGI_at	*ZNF791*	forkhead box P1	0.369	0.0282
100143713_TGI_at	*PTCHD4*	La ribonucleoprotein domain family member 4B	0.340	0.0286
100303120_TGI_at	*PTMAP3*	AKT1 substrate 1	0.313	0.0287
100146268_TGI_at	*TBCA*	chromosome X open reading frame 23	0.271	0.0287
100135653_TGI_at	*TNNI3K*	uncharacterized LOC727916	0.779	0.0288
100127735_TGI_at	*CHCHD6*	solute carrier family 39 member 7	0.243	0.0289
100130647_TGI_at	*WDR60*	corticotropin releasing hormone binding protein	1.606	0.0290
100121990_TGI_at	*C5orf17*	MAU2 sister chromatid cohesion factor	0.297	0.0291
100152345_TGI_at	*SMIM24*	kelch repeat and BTB domain containing 12	1.616	0.0292
100307025_TGI_at	*SIAH2*	NSF attachment protein alpha	0.370	0.0292
100149520_TGI_at	*PCBP2*	microtubule associated serine/threonine kinase family member 4	0.577	0.0293
100136747_TGI_at	*C16orf46*	golgin A5	0.199	0.0294
100144313_TGI_at	*RPS3A*	chromosome 6 open reading frame 89	0.368	0.0296
100134829_TGI_at	*ORAI2*	leucine rich repeat containing 8 family member A	0.265	0.0297
100132416_TGI_at	*PDZD11*	collagen type V alpha 3 chain	0.684	0.0298
100133804_TGI_at	*KCNE2*	uncharacterized LOC100131510	0.289	0.0298
100128021_TGI_at	*ITGB3BP*	agrin	0.474	0.0300
100130105_TGI_at	*TKTL1*	D-amino acid oxidase	0.221	0.0300
100147225_TGI_at	*RHBDL3*	chromobox 3 pseudogene 2	0.363	0.0301
100121621_TGI_at	*DOCK9*	aldehyde dehydrogenase 8 family member A1	0.731	0.0301
100139023_TGI_at	*GCSAML-AS1*	Rho related BTB domain containing 1	0.588	0.0301
100125331_TGI_at	*PEAR1*	zinc finger and BTB domain containing 43	0.191	0.0303
100313182_TGI_at	*CSN3*	tetraspanin 1	1.186	0.0307
100144725_TGI_at	*IL17RA*	matrix metallopeptidase 8	0.251	0.0309
100132898_TGI_at	*GEMIN6*	mucin 1, cell surface associated	1.157	0.0309
100126597_TGI_at	*NODAL*	growth factor receptor bound protein 2	0.269	0.0311
100131082_TGI_at	*PRICKLE1*	membrane associated guanylate kinase, WW and PDZ domain containing 3	0.521	0.0311
100151408_TGI_at	*TNFRSF13B*	nuclear factor I X	0.637	0.0315
100142950_TGI_at	*IGHD*	GRAM domain containing 1B	0.392	0.0316
100152940_TGI_at	*HSDL1*	mitochondrial pyruvate carrier 2	0.389	0.0316
100301119_TGI_at	*VSTM2L*	zinc finger protein 791	0.275	0.0316
100128601_TGI_at	*DNAAF1*	Rho guanine nucleotide exchange factor 10 like	0.299	0.0317
100125461_TGI_at	*TRAF6*	zinc finger homeobox 4	0.458	0.0323
100141473_TGI_at	*RAB33B*	tetraspanin 1	1.188	0.0326

100157235_TGI_at	*LOC644686*	chromosome 5 open reading frame 17	0.532	0.0326
100309806_TGI_at	*EFHD2*	tripartite motif containing 8	0.265	0.0326
100309904_TGI_at	*SCOC*	Sp2 transcription factor	0.353	0.0327
100137529_TGI_at	*HOXC10*	taste 2 receptor member 3	0.200	0.0327
100127869_TGI_at	*ATP1B2*	tropomyosin 3	0.714	0.0327
100153687_TGI_at	*CSMD1*	membrane associated guanylate kinase, WW and PDZ domain containing 3	0.493	0.0330
100130481_TGI_at	*ARHGEF11*	G protein-coupled receptor 155	0.457	0.0333
100312951_TGI_at	*ESYT3*	ST3 beta-galactoside alpha-2,3-sialyltransferase 4	0.496	0.0336
100150497_TGI_at	*AIRE*	chromosome X open reading frame 23	0.301	0.0337
100154602_TGI_at	*C12orf60*	rhomboid like 3	0.930	0.0338
100143673_TGI_at	*HIST1H4B*	dedicator of cytokinesis 9	0.396	0.0339
100140499_TGI_at	*CRIPT*	ANKH inorganic pyrophosphate transport regulator	0.502	0.0340
100313012_TGI_at	*MED31*	CTAGE family member 5, ER export factor	0.334	0.0340
100154930_TGI_at	*SLF2*	platelet endothelial aggregation receptor 1	0.652	0.0341
100302285_TGI_at	*SMIM7*	sushi domain containing 6	0.335	0.0341
100132236_TGI_at	*ANKRD55*	casein kappa	0.244	0.0343
100302321_TGI_at	*ZNF578*	interleukin 17 receptor A	0.479	0.0345
100152737_TGI_at	*ACSM3*	trio Rho guanine nucleotide exchange factor	0.316	0.0348
100309507_TGI_at	*BCL2L11*	nodal growth differentiation factor	0.177	0.0349
100312184_TGI_at	*PODNL1*	prickle planar cell polarity protein 1	0.639	0.0349
100302881_TGI_at	*GPT2*	parathyroid hormone like hormone	1.034	0.0354
100302385_TGI_at	*DNAJB1*	pre-mRNA processing factor 6	0.317	0.0357
100139928_TGI_at	*FAM212B*	long intergenic non-protein coding RNA 1393	0.370	0.0357
100151734_TGI_at	*MRO*	SRY-box 13	0.628	0.0358
100160982_TGI_at	*SREK1IP1*	SEC14 like lipid binding 2	0.473	0.0360
100141337_TGI_at	*FAM102B*	cancer susceptibility candidate 15 (non-protein coding)	0.564	0.0361
100302715_TGI_at	*ZNF354B*	TNF receptor associated factor 6	0.296	0.0361
100148826_TGI_at	*SMARCA2*	mitogen-activated protein kinase-activated protein kinase 2	0.225	0.0361
100147714_TGI_at	*CANT1*	chromosome 20 open reading frame 202	0.243	0.0362
100304151_TGI_at	*ORC5*	mucin 1, cell surface associated	0.895	0.0362
100305819_TGI_at	*HOXA9*	family with sequence similarity 168 member A	0.309	0.0362
100313257_TGI_at	*RASEF*	neuropilin 1	0.511	0.0363
100160455_TGI_at	*ITGA8*	sonic hedgehog	0.207	0.0365
100143980_TGI_at	*LOC105374366*	EF-hand domain family member D2	0.383	0.0366
100158526_TGI_at	*DPP3*	golgi brefeldin A resistant guanine nucleotide exchange factor 1	0.280	0.0366

100312061_TGI_at	*ZNF462*	membrane associated guanylate kinase, WW and PDZ domain containing 3	0.563	0.0367
100125739_TGI_at	*KIAA0196*	transmembrane protein 184B	0.226	0.0369
100303719_TGI_at	*TBC1D16*	ATPase Na+/K+ transporting subunit beta 2	0.596	0.0370
100163012_TGI_at	*EBF3*	lysophosphatidylcholine acyltransferase 3	0.276	0.0370
100145649_TGI_at	*SRPK2*	phosphodiesterase 1B	0.717	0.0371
100129008_TGI_at	*SLC22A18AS*	parathyroid hormone like hormone	1.213	0.0372
100159456_TGI_at	*FUT8*	zinc finger protein 516	0.542	0.0373
100134094_TGI_at	*MMD2*	MYC associated factor X	0.184	0.0375
100137049_TGI_at	*ARHGEF2*	Rho guanine nucleotide exchange factor 11	0.283	0.0375
100312132_TGI_at	*NKX2-5*	protein kinase N2	0.239	0.0375
100302758_TGI_at	*DNASE2*	solute carrier family 1 member 1	1.054	0.0376
100148386_TGI_at	*RTF1*	insulin like 4	0.187	0.0376
100146748_TGI_at	*TRMT10C*	methyl-CpG binding protein 2	0.277	0.0377
100153282_TGI_at	*DPY19L3*	CREB3 regulatory factor	0.322	0.0381
100124891_TGI_at	*GUCY1A2*	SMC5-SMC6 complex localization factor 2	0.370	0.0382
100309244_TGI_at	*ZSCAN32*	small integral membrane protein 7	0.231	0.0382
100149751_TGI_at	*IFT22*	zinc finger protein 827	0.542	0.0384
100139731_TGI_at	*PRKX*	zinc finger protein 430	0.266	0.0386
100126347_TGI_at	*GPR35*	zinc finger protein 827	0.553	0.0386
100129408_TGI_at	*TAS2R40*	ERCC excision repair 5, endonuclease	0.237	0.0387
100135737_TGI_at	*SGK2*	acyl-CoA synthetase medium-chain family member 3	0.752	0.0387
100302051_TGI_at	*CCDC65*	transmembrane protein 127	0.268	0.0388
100150811_TGI_at	*BET1L*	long intergenic non-protein coding RNA 849	0.353	0.0390
100128921_TGI_at	*FER1L4*	BCL2 like 11	0.448	0.0391
100127195_TGI_at	*SRGAP3*	podocan like 1	0.235	0.0391
100124961_TGI_at	*CPNE4*	lysine demethylase 2A	0.254	0.0392
100131935_TGI_at	*ABHD13*	leucine rich repeat containing 4	0.625	0.0397
100133914_TGI_at	*IGLV3-25*	capicua transcriptional repressor	0.417	0.0398
100144237_TGI_at	*LOC100133985*	folliculin interacting protein 1	0.240	0.0399
100303233_TGI_at	*B3GALNT1*	family with sequence similarity 222 member B	0.269	0.0400
100128270_TGI_at	*BBX*	uncharacterized LOC101927354	0.182	0.0401
100311760_TGI_at	*TFCP2L1*	family with sequence similarity 212 member B	0.441	0.0402
100138221_TGI_at	*TMEM231*	maestro	0.676	0.0403
100158127_TGI_at	*PSMA5*	SREK1 interacting protein 1	0.515	0.0403
100151039_TGI_at	*DIP2A*	family with sequence similarity 102 member B	0.426	0.0404
100124937_TGI_at	*SOX18*	5'-nucleotidase, cytosolic II	0.230	0.0404
100309503_TGI_at	*PPP1R1A*	SWI/SNF related, matrix associated, actin dependent regulator of chromatin, subfamily a, member 2	0.311	0.0405

100161851_TGI_at	*LOC101927571*	calcium activated nucleotidase 1	0.299	0.0406
100148295_TGI_at	*GEMIN5*	RAS and EF-hand domain containing	0.730	0.0408
100125197_TGI_at	*MIOS*	serine incorporator 2	0.692	0.0409
100125374_TGI_at	*MIR181A2HG*	dipeptidyl peptidase 3	0.318	0.0411
100308653_TGI_at	*GPX6*	zinc finger protein 462	0.437	0.0411
100134164_TGI_at	*FAM162A*	KIAA0196	0.190	0.0412
100130123_TGI_at	*KDM6A*	solute carrier family 6 member 6	0.504	0.0413
100162003_TGI_at	*PRKRIP1*	KIAA0513	0.528	0.0413
100140272_TGI_at	*SLC39A10*	TBC1 domain family member 16	0.386	0.0414
100150271_TGI_at	*ABHD10*	early B-cell factor 3	0.838	0.0414
100310934_TGI_at	*BPNT1*	sushi, nidogen and EGF like domains 1	0.440	0.0415
100142439_TGI_at	*AREL1*	solute carrier family 22 member 18 antisense	0.424	0.0418
100157932_TGI_at	*RCHY1*	platelet derived growth factor subunit B	0.385	0.0418
100132933_TGI_at	*BAZ2A*	fucosyltransferase 8	0.360	0.0418
100132618_TGI_at	*ACHE*	zinc finger protein 75D	0.278	0.0419
100145955_TGI_at	*CEP104*	Rho/Rac guanine nucleotide exchange factor 2	0.244	0.0423
100132785_TGI_at	*ZNF618*	deoxyribonuclease 2, lysosomal	0.446	0.0424
100302247_TGI_at	*GNG8*	pyridoxal dependent decarboxylase domain containing 1	0.315	0.0425
100309599_TGI_at	*SNRPE*	IQ motif containing K	0.325	0.0427
100139653_TGI_at	*BARHL2*	phospholipase A2 group IIA	1.594	0.0428
100134356_TGI_at	*DNAJC27-AS1*	nuclear factor I C	0.384	0.0431
100157383_TGI_at	*ARMC10*	zinc finger and SCAN domain containing 32	0.255	0.0436
100140865_TGI_at	*NHSL2*	alpha-methylacyl-CoA racemase	0.450	0.0436
100135583_TGI_at	*ZFP42*	protein kinase, X-linked	0.413	0.0439
100125997_TGI_at	*SEC61G*	G protein-coupled receptor 35	0.262	0.0440
100133973_TGI_at	*PVALB*	SGK2, serine/threonine kinase 2	0.865	0.0441
100132475_TGI_at	*B3GNT3*	nuclear factor I C	0.326	0.0443
100136783_TGI_at	*LOC285629*	Bet1 golgi vesicular membrane trafficking protein like	0.226	0.0443
100122206_TGI_at	*CCL21*	membrane metallo-endopeptidase	1.221	0.0444
100150024_TGI_at	*PCDHB17P*	SLIT-ROBO Rho GTPase activating protein 3	0.554	0.0445

Under-expressed genes in Metastases vs. Primary MCC tumors

100148385_TGI_at	*ICAM5*	intercellular adhesion molecule 5	-0.340	0.0005
100310903_TGI_at	*C1orf167*	chromosome 1 open reading frame 167	-0.755	0.0011
100155284_TGI_at	*ZNF860*	zinc finger protein 860	-0.411	0.0012
100308040_TGI_at	*DNAJC12*	DnaJ heat shock protein family (Hsp40) member C12	-0.796	0.0012
100129908_TGI_at	*ART4*	ADP-ribosyltransferase 4 (Dombrock blood group)	-0.906	0.0014

100143642_TGI_at	*INSIG2*	insulin induced gene 2	-0.541	0.0014
100313696_TGI_at	*WDR27*	WD repeat domain 27	-0.474	0.0014
100151614_TGI_at	*KCTD15*	DnaJ heat shock protein family (Hsp40) member C12	-1.199	0.0015
100123994_TGI_at	*MPP3*	potassium channel tetramerization domain containing 15	-0.365	0.0016
100302960_TGI_at	*STAC3*	membrane palmitoylated protein 3	-1.137	0.0018
100313808_TGI_at	*NDUFA5*	DnaJ heat shock protein family (Hsp40) member C12	-1.309	0.0024
100140484_TGI_at	*RHBDD1*	hemoglobin subunit theta 1	-0.842	0.0025
100128055_TGI_at	*TMEM266*	glutamate ionotropic receptor AMPA type subunit 4	-1.551	0.0026
100140974_TGI_at	*CUL2*	NADH:ubiquinone oxidoreductase subunit A5	-0.496	0.0027
100150815_TGI_at	*NT5C2*	peptidylprolyl isomerase H	-0.411	0.0029
100159115_TGI_at	*RMND5A*	rhomboid domain containing 1	-0.556	0.0030
100159957_TGI_at	*WAC*	transmembrane protein 266	-0.796	0.0033
100125161_TGI_at	*VMA21*	required for meiotic nuclear division 5 homolog A	-0.445	0.0040
100136455_TGI_at	*SLC30A7*	cholinergic receptor muscarinic 2	-0.304	0.0044
100127092_TGI_at	*TTC9B*	VMA21 vacuolar H+-ATPase homolog (S. cerevisiae)	-0.236	0.0047
100303979_TGI_at	*STAT3*	SEL1L2 ERAD E3 ligase adaptor subunit	-0.472	0.0048
100122141_TGI_at	*KCTD14*	tetratricopeptide repeat domain 9B	-0.274	0.0050
100139005_TGI_at	*LOC101929305*	crumbs 2, cell polarity complex component	-0.277	0.0051
100306052_TGI_at	*IGHM*	insulin induced gene 2	-0.478	0.0052
100143483_TGI_at	*PHF12*	syntaxin 1B	-0.282	0.0053
100140955_TGI_at	*PADI2*	uncharacterized LOC101929305	-0.448	0.0053
100310050_TGI_at	*LINC00944*	immunoglobulin heavy constant mu	-2.515	0.0055
100301826_TGI_at	*BOLL*	glutamate receptor interacting protein 1	-0.411	0.0056
100151710_TGI_at	*QDPR*	long intergenic non-protein coding RNA 944	-0.579	0.0060
100141975_TGI_at	*LZTS1*	Sp8 transcription factor	-1.492	0.0062
100154638_TGI_at	*ANKRD37*	boule homolog, RNA binding protein	-0.957	0.0063
100150535_TGI_at	*FAM222B*	insulin induced gene 2	-0.367	0.0063
100139272_TGI_at	*FBRS*	T-cell leukemia/lymphoma 6 (non-protein coding)	-0.269	0.0066
100122190_TGI_at	*ADGRA1*	KIAA1644	-0.458	0.0066
100137109_TGI_at	*LOC642862///RHEB*	leucine rich repeat containing 6	-0.660	0.0069
100311459_TGI_at	*LINC01254*	quinoid dihydropteridine reductase	-0.452	0.0070
100152778_TGI_at	*EFCAB6*	ankyrin repeat domain 37	-0.676	0.0071
100308176_TGI_at	*CR2*	insulin induced gene 2	-0.514	0.0079
100122054_TGI_at	*CLEC4M*	adhesion G protein-coupled receptor A1	-0.672	0.0081
100308575_TGI_at	*BTBD9*	Ras homolog enriched in brain pseudogene///Ras homolog enriched in brain	-0.227	0.0084
100138973_TGI_at	*KIAA1456*	long intergenic non-protein coding RNA 1254	-0.511	0.0084

100313045_TGI_at	*TDRD9*	uncharacterized LOC100130744	-0.251	0.0085
100300058_TGI_at	*TXNDC9*	EF-hand calcium binding domain 6	-0.384	0.0085
100304517_TGI_at	*TSPAN14*	peptidylprolyl isomerase H	-0.363	0.0086
100131242_TGI_at	*RPL23AP53*	cerebellin 1 precursor	-1.136	0.0087
100128636_TGI_at	*LOC338620*	complement component 3d receptor 2	-1.354	0.0087
100312466_TGI_at	*IQCF3*	T-box, brain 1	-1.040	0.0089
100125437_TGI_at	*GRIN2A*	C-type lectin domain family 4 member M	-0.434	0.0090
100137626_TGI_at	*GLP2R*	KIAA1456	-1.023	0.0093
100310905_TGI_at	*AFDN*	tudor domain containing 9	-0.794	0.0094
100301810_TGI_at	*CCDC167*	DnaJ heat shock protein family (Hsp40) member C12	-1.079	0.0095
100309772_TGI_at	*FRS2*	thioredoxin domain containing 9	-0.428	0.0096
100304214_TGI_at	*SLC35B4*	ribosomal protein L23a pseudogene 53	-0.559	0.0100
100135730_TGI_at	*CTAGE5*	IQ motif containing F3	-0.413	0.0101
100129434_TGI_at	*HSDL2*	glutamate ionotropic receptor NMDA type subunit 2A	-1.450	0.0102
100313477_TGI_at	*CRLS1*	adhesion G protein-coupled receptor A1	-0.445	0.0102
100153603_TGI_at	*REPS2*	Fc fragment of IgE receptor II	-0.468	0.0103
100154049_TGI_at	*RDH13*	glucagon like peptide 2 receptor	-0.545	0.0104
100159351_TGI_at	*PLEKHH1*	T-box, brain 1	-0.927	0.0107
100130029_TGI_at	*JAM3*	afadin, adherens junction formation factor	-0.664	0.0108
100126946_TGI_at	*PPP1R17*	coiled-coil domain containing 167	-0.349	0.0110
100304504_TGI_at	*NRDE2*	ribosomal protein L35	-0.194	0.0113
100134529_TGI_at	*LEPR*	protocadherin related 15	-0.952	0.0113
100154729_TGI_at	*SNX5*	solute carrier family 35 member B4	-0.424	0.0114
100313285_TGI_at	*WDR59*	antizyme inhibitor 1	-0.357	0.0115
100312123_TGI_at	*ZBTB3*	hydroxysteroid dehydrogenase like 2	-0.475	0.0117
100304650_TGI_at	*CDH4*	cardiolipin synthase 1	-0.446	0.0117
100147403_TGI_at	*GOLM1*	retinol dehydrogenase 13	-0.291	0.0119
100163079_TGI_at	*TMED10*	CD19 molecule	-0.560	0.0119
100139973_TGI_at	*QSOX1*	junctional adhesion molecule 3	-0.535	0.0121
100148063_TGI_at	*CCT4*	protein phosphatase 1 regulatory subunit 17	-1.143	0.0122
100132343_TGI_at	*LMO4*	nucleolar protein with MIF4G domain 1	-0.522	0.0123
100134018_TGI_at	*USP45*	leptin receptor	-0.669	0.0124
100138585_TGI_at	*LINC00493*	alpha tocopherol transfer protein like	-0.307	0.0125
100134770_TGI_at	*HOXC-AS3*	sorting nexin 5	-0.760	0.0127
100305943_TGI_at	*LINC01114*	UBX domain protein 10	-0.498	0.0127
100130052_TGI_at	*WDR33*	WD repeat domain 59	-0.536	0.0128
100301782_TGI_at	*DUXAP10*	myeloid leukemia factor 1	-0.726	0.0130
100139915_TGI_at	*C12orf29*	WD repeat domain 59	-0.327	0.0131
100131432_TGI_at	*M1AP*	retinoid X receptor gamma	-0.528	0.0131
100301678_TGI_at	*SLC6A6*	cadherin 4	-1.231	0.0132
100129396_TGI_at	*TTC29*	chromosome 3 open reading frame 35	-0.207	0.0134

100313809_TGI_at	*TGOLN2*	transmembrane p24 trafficking protein 10	-0.309	0.0136
100313359_TGI_at	*THBS4*	chaperonin containing TCP1 subunit 4	-0.238	0.0139
100152463_TGI_at	*DCUN1D4*	cadherin 4	-0.864	0.0139
100311062_TGI_at	*LINC01296///DUXAP10///DUXAP8*	cordon-bleu WH2 repeat protein	-0.757	0.0141
100149952_TGI_at	*LGI3*	ubiquitin specific peptidase 45	-0.369	0.0142
100129752_TGI_at	*PARD6G-AS1*	long intergenic non-protein coding RNA 493	-0.286	0.0143
100158813_TGI_at	*TTC8*	HOXC cluster antisense RNA 3	-0.387	0.0143
100127355_TGI_at	*NTS*	long intergenic non-protein coding RNA 1114	-0.280	0.0143
100155473_TGI_at	*NFIC*	WD repeat domain 33	-0.433	0.0143
100122368_TGI_at	*EPPIN*	double homeobox A pseudogene 10	-1.246	0.0145
100305764_TGI_at	*SLC25A37*	leucine rich repeat containing 6	-0.775	0.0145
100129688_TGI_at	*HYDIN*	chromosome 12 open reading frame 29	-0.354	0.0146
100139888_TGI_at	*ZBED9*	tetratricopeptide repeat domain 29	-0.750	0.0150
100130921_TGI_at	*LGALS14*	glycine receptor alpha 3	-0.598	0.0154
100136308_TGI_at	*CALCR*	solute carrier family 4 member 10	-0.521	0.0154
100155494_TGI_at	*CXorf23*	defective in cullin neddylation 1 domain containing 4	-0.334	0.0156
100142750_TGI_at	*MMP15*	long intergenic non-protein coding RNA 1296///double homeobox A pseudogene 10///double homeobox A pseudogene 8	-1.398	0.0160
100125145_TGI_at	*GSG1///TRAPPC8*	leucine rich repeat LGI family member 3	-0.418	0.0160
100307669_TGI_at	*DPF2*	PARD6G antisense RNA 1	-0.482	0.0161
100132943_TGI_at	*ST3GAL4*	neurotensin	-1.277	0.0163
100153414_TGI_at	*CTB-178M22.2*	epididymal peptidase inhibitor	-0.219	0.0165
100304840_TGI_at	*SLC4A4*	solute carrier family 25 member 37	-0.400	0.0165
100311584_TGI_at	*LOC101927967*	HYDIN, axonemal central pair apparatus protein	-0.431	0.0166
100155347_TGI_at	*DNAJC19*	UBX domain protein 10	-0.688	0.0170
100140536_TGI_at	*SUOX*	zinc finger BED-type containing 9	-1.647	0.0170
100160722_TGI_at	*DNALI1*	butyrophilin subfamily 1 member A1	-0.770	0.0173
100301528_TGI_at	*EFCAB1*	hydroxysteroid dehydrogenase like 2	-0.373	0.0174
100141901_TGI_at	*TIGD4*	galectin 14	-0.246	0.0174
100134863_TGI_at	*BHMT*	calcitonin receptor	-0.242	0.0174
100129145_TGI_at	*COMMD2*	cytochrome c oxidase assembly factor 1 homolog	-0.387	0.0177
100301167_TGI_at	*MPC2*	nephronophthisis 3 (adolescent)	-0.271	0.0178
100137388_TGI_at	*GATB*	germ cell associated 1///trafficking protein particle complex 8	-0.777	0.0179
100153515_TGI_at	*NTSR2*	ST8 alpha-N-acetyl-neuraminide alpha-2,8-sialyltransferase 2	-0.266	0.0180
100302952_TGI_at	*LINC01048*	glucagon	-0.453	0.0183
100306495_TGI_at	*MRPL52*	STARD3 N-terminal like	-0.341	0.0183

100154064_TGI_at	*B3GNT2*	uncharacterized LOC101927862	-0.754	0.0184
100132463_TGI_at	*MIR4458HG*	uncharacterized LOC101927967	-0.538	0.0188
100313629_TGI_at	*DCDC5*	ariadne RBR E3 ubiquitin protein ligase 2	-0.333	0.0189
100162828_TGI_at	*KCTD5*	biphenyl hydrolase like	-0.420	0.0190
100134462_TGI_at	*SLC20A2*	oxysterol binding protein like 10	-0.545	0.0190
100310056_TGI_at	*GACAT2*	DnaJ heat shock protein family (Hsp40) member C19	-0.271	0.0191
100127796_TGI_at	*ATP6V0C*	dynein axonemal light intermediate chain 1	-0.850	0.0191
100304858_TGI_at	*EXOC4*	myeloid leukemia factor 1	-0.689	0.0192
100123606_TGI_at	*GABRG1*	myeloid leukemia factor 1	-0.766	0.0193
100133510_TGI_at	*THADA*	EF-hand calcium binding domain 1	-0.521	0.0193
100151991_TGI_at	*FAM13C*	membrane palmitoylated protein 3	-0.577	0.0194
100141779_TGI_at	*PYDC2*	tigger transposable element derived 4	-0.260	0.0194
100128876_TGI_at	*WNT2*	betaine--homocysteine S-methyltransferase	-0.629	0.0194
100158134_TGI_at	*BTBD17*	KIAA1456	-1.066	0.0194
100156188_TGI_at	*MUC12*	COMM domain containing 2	-0.373	0.0194
100136695_TGI_at	*SLC16A10*	homeobox A2	-1.189	0.0195
100308883_TGI_at	*SNHG15*	solute carrier family 35 member B4	-0.482	0.0197
100313544_TGI_at	*TRPV6*	glutamyl-tRNA amidotransferase subunit B	-0.453	0.0198
100133746_TGI_at	*RGSL1*	neurotensin receptor 2	-0.494	0.0198
100300939_TGI_at	*STX6*	mitochondrial ribosomal protein L52	-0.386	0.0200
100154946_TGI_at	*DCAF13*	Rap associating with DIL domain	-0.719	0.0200
100125779_TGI_at	*SSC4D*	hydroxysteroid dehydrogenase like 2	-0.499	0.0200
100136641_TGI_at	*C6orf89*	glutamate ionotropic receptor AMPA type subunit 4	-1.052	0.0201
100122624_TGI_at	*HOTAIRM1///HOXA2*	serpin family E member 1	-0.907	0.0201
100303072_TGI_at	*IL1RL1*	intraflagellar transport 43	-0.247	0.0202
100143514_TGI_at	*SP2*	doublecortin domain containing 5	-1.736	0.0204
100147461_TGI_at	*SRMS*	chromosome 11 open reading frame 70	-1.038	0.0205
100303236_TGI_at	*IL1RAPL2*	glutamate ionotropic receptor AMPA type subunit 4	-0.878	0.0206
100304864_TGI_at	*WIPF2*	solute carrier family 20 member 2	-0.341	0.0206
100129103_TGI_at	*LINC00839*	nuclear receptor subfamily 1 group I member 3	-0.434	0.0211
100147965_TGI_at	*FUNDC2*	glutamate ionotropic receptor NMDA type subunit 2A	-0.564	0.0212
100142376_TGI_at	*TMEM127*	exocyst complex component 4	-0.324	0.0215
100159569_TGI_at	*HIVEP1*	gamma-aminobutyric acid type A receptor gamma1 subunit	-0.556	0.0215
100311032_TGI_at	*SPTB*	THADA, armadillo repeat containing	-0.301	0.0215
100123444_TGI_at	*LOC441178*	pyrin domain containing 2	-0.223	0.0218
100130955_TGI_at	*ELL*	BTB domain containing 17	-0.473	0.0219
100129808_TGI_at	*EBF2*	mucin 12, cell surface associated	-0.323	0.0224
100143058_TGI_at	*MYRF*	solute carrier family 16 member 10	-0.732	0.0224
100127254_TGI_at	*RBM26*	small nucleolar RNA host gene 15	-0.460	0.0225

100146977_TGI_at	*PCF11*	transient receptor potential cation channel subfamily V member 6	-0.189	0.0225
100153795_TGI_at	*RABGEF1*	regulator of G-protein signaling like 1	-0.283	0.0226
100145119_TGI_at	*LOC101927876*	poly(A) binding protein cytoplasmic 1 like 2B	-0.229	0.0226
100159600_TGI_at	*CPA2*	poly(A) polymerase alpha	-0.244	0.0228
100142856_TGI_at	*ZFAND3*	DDB1 and CUL4 associated factor 13	-0.309	0.0229
100136814_TGI_at	*LINC00473*	cadherin 4	-1.362	0.0229
100157888_TGI_at	*SOX5*	scavenger receptor cysteine rich family member with 4 domains	-0.464	0.0229
100144358_TGI_at	*ATP11B*	sarcoglycan beta	-0.410	0.0231
100160131_TGI_at	*KLHL41*	HOXA transcript antisense RNA, myeloid-specific 1///homeobox A2	-0.487	0.0232
100153549_TGI_at	*LL0XNC01-116E7.2*	importin 11	-0.307	0.0234
100130545_TGI_at	*PDPR*	defective in cullin neddylation 1 domain containing 1	-0.357	0.0235
100136570_TGI_at	*KCNE1*	interleukin 1 receptor accessory protein like 2	-0.305	0.0236
100304604_TGI_at	*ZFYVE1*	cleavage and polyadenylation factor I subunit 1	-0.292	0.0236
100123769_TGI_at	*DMRTA2*	long intergenic non-protein coding RNA 839	-0.420	0.0238
100147241_TGI_at	*CARMIL1*	FUN14 domain containing 2	-0.238	0.0238
100131380_TGI_at	*CYTH1*	POU class 4 homeobox 2	-2.154	0.0238
100301408_TGI_at	*SOCS2*	zinc finger BED-type containing 9	-1.390	0.0241
100300844_TGI_at	*CNIH1*	SCO-spondin	-0.521	0.0242
100151438_TGI_at	*LOC102546294*	myelin regulatory factor	-0.586	0.0247
100156584_TGI_at	*IGLV3-21*	negative regulator of antiviral response (non-protein coding)	-0.538	0.0247
100308892_TGI_at	*TRMU///TRNT1*	RAB guanine nucleotide exchange factor 1	-0.524	0.0249
100145202_TGI_at	*GALNT5*	uncharacterized LOC101927876	-0.245	0.0252
100158468_TGI_at	*GLG1*	semaphorin 3A	-1.079	0.0254
100146928_TGI_at	*ACER2*	carboxypeptidase A2	-0.397	0.0254
100137614_TGI_at	*GABRA2*	SRY-box 5	-0.663	0.0256
100135724_TGI_at	*AKT1S1*	ATPase phospholipid transporting 11B (putative)	-0.477	0.0256
100158079_TGI_at	*C8orf34-AS1*	regulator of calcineurin 2	-0.640	0.0257
100305082_TGI_at	*LOC727916*	hydroxysteroid dehydrogenase like 2	-0.503	0.0257
100148002_TGI_at	*ABCC5-AS1*	paraneoplastic Ma antigen 2	-1.497	0.0257
100159379_TGI_at	*KBTBD12*	uncharacterized LOC100128594	-0.353	0.0259
100308581_TGI_at	*MAST4*	HOXA transcript antisense RNA, myeloid-specific 1///homeobox A2	-0.912	0.0259
100138214_TGI_at	*SLC44A5*	cordon-bleu WH2 repeat protein	-1.075	0.0262
100159685_TGI_at	*MYNN*	pyruvate dehydrogenase phosphatase regulatory subunit	-0.671	0.0262
100163176_TGI_at	*OR2C3*	cardiolipin synthase 1	-0.287	0.0263
100309938_TGI_at	*LRRC8A*	methylcrotonoyl-CoA carboxylase 2	-0.380	0.0265

100309942_TGI_at	*COL5A3*	alcohol dehydrogenase 5 (class III), chi polypeptide	-0.300	0.0265
100150898_TGI_at	*ALDH8A1*	solute carrier family 27 member 5	-0.562	0.0270
100124449_TGI_at	*UBE2G2*	cornichon family AMPA receptor auxiliary protein 1	-0.275	0.0271
100309797_TGI_at	*TSPAN1*	methyltransferase like 6	-0.321	0.0273
100123400_TGI_at	*IGF2BP3*	neuropeptide Y receptor Y5	-0.637	0.0274
100307928_TGI_at	*PRMT2*	translocase of outer mitochondrial membrane 5	-0.278	0.0276
100152248_TGI_at	*LINC01224*	uncharacterized LOC285500	-0.439	0.0277
100139443_TGI_at	*GRB2*	uncharacterized LOC102546294	-0.271	0.0277
100135842_TGI_at	*MAGI3*	immunoglobulin lambda variable 3-21	-1.895	0.0278
100154573_TGI_at	*GPR135*	collagen type IV alpha 6 chain	-0.377	0.0280
100158839_TGI_at	*GRAMD1B*	hydroxysteroid dehydrogenase like 2	-0.495	0.0281
100153391_TGI_at	*FGF14*	tRNA 5-methylaminomethyl-2-thiouridylate methyltransferase///tRNA nucleotidyl transferase 1	-0.354	0.0282
100129226_TGI_at	*ARHGEF10L*	golgi glycoprotein 1	-0.391	0.0283
100138426_TGI_at	*BARX1*	alkaline ceramidase 2	-0.406	0.0283
100157186_TGI_at	*NR0B1*	mesencephalic astrocyte derived neurotrophic factor	-0.273	0.0284
100148048_TGI_at	*NPTX1*	olfactory receptor family 7 subfamily G member 3	-0.212	0.0286
100308388_TGI_at	*SGCE*	gamma-aminobutyric acid type A receptor alpha2 subunit	-0.490	0.0286
100158406_TGI_at	*NDUFA13*	glutamate ionotropic receptor NMDA type subunit 2A	-0.468	0.0287
100133026_TGI_at	*ZFHX4*	C8orf34 antisense RNA 1	-0.880	0.0287
100134567_TGI_at	*SPR*	ABCC5 antisense RNA 1	-0.224	0.0290
100160065_TGI_at	*TAS2R3*	Bardet-Biedl syndrome 7	-0.400	0.0293
100309603_TGI_at	*TPM3*	solute carrier family 44 member 5	-0.824	0.0293
100159199_TGI_at	*DCTD*	myoneurin	-0.330	0.0293
100157020_TGI_at	*LOC497256*	LSM1 homolog, mRNA degradation associated	-0.248	0.0294
100143983_TGI_at	*SUSD1*	olfactory receptor family 2 subfamily C member 3	-0.322	0.0295
100145961_TGI_at	*SSFA2*	mediator complex subunit 18	-0.328	0.0296
100142250_TGI_at	*GPR155*	NME/NM23 family member 7	-0.472	0.0298
100123177_TGI_at	*LOC101927905*	RNA polymerase III subunit D	-0.419	0.0299
100130629_TGI_at	*SNORA4*	ubiquitin conjugating enzyme E2 G2	-0.645	0.0301
100124801_TGI_at	*SUSD6*	hyaluronan and proteoglycan link protein 4	-0.295	0.0304
100133562_TGI_at	*DEFB123*	KIAA1143	-0.273	0.0304
100134038_TGI_at	*FCAMR*	insulin like growth factor 2 mRNA binding protein 3	-0.687	0.0309
100145642_TGI_at	*LOC100507412 ///RNA5-8S5*	protein arginine methyltransferase 2	-0.329	0.0309
100148247_TGI_at	*TRIO*	long intergenic non-protein coding RNA 1224	-0.860	0.0310

100125606_TGI_at	*RGS2*	immunoglobulin superfamily member 11	-0.539	0.0310
100132854_TGI_at	*GABRA5*	family with sequence similarity 234 member B	-0.729	0.0314
100123074_TGI_at	*MRPL47*	G protein-coupled receptor 135	-0.350	0.0315
100140725_TGI_at	*THUMPD3*	fibroblast growth factor 14	-0.766	0.0316
100303475_TGI_at	*PPIL6*	ERCC excision repair 8, CSA ubiquitin ligase complex subunit	-0.494	0.0316
100158068_TGI_at	*LINC01158*	BARX homeobox 1	-0.370	0.0318
100141385_TGI_at	*PRPF6*	nuclear receptor subfamily 0 group B member 1	-0.599	0.0318
100155847_TGI_at	*LINC01393*	patched domain containing 4	-0.446	0.0319
100131417_TGI_at	*SOX13*	neuronal pentraxin 1	-1.582	0.0319
100126039_TGI_at	*TARID*	sarcoglycan epsilon	-0.631	0.0321
100141785_TGI_at	*SEC14L2*	prothymosin, alpha pseudogene 3	-0.256	0.0321
100144355_TGI_at	*DCDC1*	NADH:ubiquinone oxidoreductase subunit A13	-0.189	0.0323
100304373_TGI_at	*CASC15*	tubulin folding cofactor A	-0.306	0.0323
100155734_TGI_at	*MAPKAPK2*	TNNI3 interacting kinase	-0.536	0.0324
100143725_TGI_at	*C20orf202*	coiled-coil-helix-coiled-coil-helix domain containing 6	-0.326	0.0325
100149157_TGI_at	*FAM168A*	sepiapterin reductase (7,8-dihydrobiopterin:NADP+ oxidoreductase)	-0.432	0.0325
100304809_TGI_at	*NRP1*	WD repeat domain 60	-0.336	0.0325
100141500_TGI_at	*SHH*	glutamyl-tRNA amidotransferase subunit B	-0.443	0.0326
100300217_TGI_at	*GBF1*	small integral membrane protein 24	-0.601	0.0326
100145882_TGI_at	*TMEM184B*	siah E3 ubiquitin protein ligase 2	-0.272	0.0326
100129528_TGI_at	*CHL1*	poly(rC) binding protein 2	-0.196	0.0327
100140780_TGI_at	*LPCAT3*	dCMP deaminase	-0.452	0.0328
100123765_TGI_at	*MRS2*	quinoid dihydropteridine reductase	-0.317	0.0329
100126194_TGI_at	*TTC26*	chromosome 16 open reading frame 46	-0.287	0.0329
100137844_TGI_at	*PDE1B*	uncharacterized LOC497256	-0.194	0.0330
100309336_TGI_at	*FLT4*	sushi domain containing 1	-0.357	0.0332
100127103_TGI_at	*ZNF516*	sperm specific antigen 2	-0.608	0.0332
100160613_TGI_at	*FAM71A*	ribosomal protein S3A	-0.364	0.0333
100154838_TGI_at	*SOX30*	ORAI calcium release-activated calcium modulator 2	-0.213	0.0333
100313161_TGI_at	*PKN2*	PDZ domain containing 11	-0.274	0.0333
100155146_TGI_at	*SLC1A1*	potassium voltage-gated channel subfamily E regulatory subunit 2	-0.560	0.0334
100311344_TGI_at	*INSL4*	uncharacterized LOC101927905	-0.360	0.0335
100162321_TGI_at	*MECP2*	integrin subunit beta 3 binding protein	-0.417	0.0336
100307395_TGI_at	*CCDC58*	transketolase like 1	-0.361	0.0336
100128489_TGI_at	*SYT11*	GCSAML antisense RNA 1	-1.215	0.0339
100139053_TGI_at	*CREBRF*	small nucleolar RNA, H/ACA box 4	-0.440	0.0341
100162413_TGI_at	*BNIP3*	defensin beta 123	-0.253	0.0342
100313000_TGI_at	*ZNF430*	Fc fragment of IgA and IgM receptor	-0.341	0.0344
100162978_TGI_at	*B3GAT1*	gem nuclear organelle associated protein 6	-0.489	0.0345

100313688_TGI_at	*ERCC5*	uncharacterized LOC100507412///RNA, 5.8S ribosomal 5	-0.482	0.0347
100158261_TGI_at	*LINC00849*	regulator of G-protein signaling 2	-0.696	0.0349
100310021_TGI_at	*KDM2A*	gamma-aminobutyric acid type A receptor alpha5 subunit	-0.863	0.0349
100126658_TGI_at	*SNORA71D*	TNF receptor superfamily member 13B	-0.551	0.0350
100309811_TGI_at	*CEP83*	mitochondrial ribosomal protein L47	-0.237	0.0350
100142954_TGI_at	*SRPRB*	immunoglobulin heavy constant delta	-1.205	0.0353
100152092_TGI_at	*HTR6*	THUMP domain containing 3	-0.277	0.0354
100146439_TGI_at	*LRRC4*	peptidylprolyl isomerase like 6	-0.741	0.0354
100148530_TGI_at	*CIC*	cardiolipin synthase 1	-0.368	0.0355
100312796_TGI_at	*OLIG2*	hydroxysteroid dehydrogenase like 1	-0.383	0.0356
100136678_TGI_at	*FNIP1*	V-set and transmembrane domain containing 2 like	-0.953	0.0356
100129608_TGI_at	*LOC100507291*	dynein axonemal assembly factor 1	-0.410	0.0356
100305798_TGI_at	*LOC101927354*	long intergenic non-protein coding RNA 1158	-1.404	0.0356
100140380_TGI_at	*TFF3*	TCF21 antisense RNA inducing promoter demethylation	-0.282	0.0358
100144558_TGI_at	*CETN3*	doublecortin domain containing 1	-0.460	0.0361
100305484_TGI_at	*ANKRD46*	fibroblast growth factor 14	-0.352	0.0361
100157964_TGI_at	*SERINC2*	RAB33B, member RAS oncogene family	-0.307	0.0364
100128011_TGI_at	*SLCO4A1*	uncharacterized LOC644686	-0.228	0.0364
100157934_TGI_at	*GCGR*	cell adhesion molecule L1 like	-0.520	0.0369
100149184_TGI_at	*FNDC9*	short coiled-coil protein	-0.303	0.0369
100158652_TGI_at	*KIAA0513*	homeobox C10	-1.437	0.0369
100309250_TGI_at	*SNED1*	MRS2, magnesium transporter	-0.478	0.0370
100159640_TGI_at	*HOTAIRM1*	tetratricopeptide repeat domain 26	-0.533	0.0371
100308165_TGI_at	*CCDC70*	quinoid dihydropteridine reductase	-0.331	0.0372
100151204_TGI_at	*TRIM51*	CUB and Sushi multiple domains 1	-0.574	0.0373
100126425_TGI_at	*PDGFB*	fms related tyrosine kinase 4	-0.231	0.0373
100137286_TGI_at	*ZNF75D*	family with sequence similarity 71 member A	-0.239	0.0373
100129603_TGI_at	*FAM114A2*	SRY-box 30	-0.519	0.0374
100133403_TGI_at	*GSG1*	coiled-coil domain containing 58	-0.325	0.0377
100134627_TGI_at	*PDXDC1*	extended synaptotagmin 3	-0.949	0.0378
100160635_TGI_at	*DLGAP5*	autoimmune regulator	-0.181	0.0378
100127917_TGI_at	*IQCK*	chromosome 12 open reading frame 60	-0.416	0.0379
100130560_TGI_at	*PLA2G2A*	histone cluster 1, H4b	-0.365	0.0379
100149415_TGI_at	*UTS2*	synaptotagmin 11	-0.406	0.0380
100302337_TGI_at	*LINC01004*	CXXC repeat containing interactor of PDZ3 domain	-0.335	0.0380
100122425_TGI_at	*DYX1C1*	mediator complex subunit 31	-0.353	0.0380
100139816_TGI_at	*SNORA80A*	T-cell leukemia/lymphoma 6 (non-protein coding)	-0.329	0.0382
100309805_TGI_at	*AMACR*	BCL2 interacting protein 3	-0.581	0.0383

100128484_TGI_at	*BHLHB9*	ankyrin repeat domain 55	-0.447	0.0385
100155695_TGI_at	*DDX52*	zinc finger protein 578	-0.352	0.0386
100143064_TGI_at	*TMED4*	Fc fragment of IgA and IgM receptor	-0.473	0.0386
100142633_TGI_at	*SUN1*	beta-1,3-glucuronyltransferase 1	-0.882	0.0386
100137157_TGI_at	*DDX18*	small nucleolar RNA, H/ACA box 71D	-0.291	0.0393
100155755_TGI_at	*CCDC173*	centrosomal protein 83	-0.426	0.0393
100305459_TGI_at	*SIX6*	defective in cullin neddylation 1 domain containing 4	-0.396	0.0394
100147680_TGI_at	*OAF*	SRP receptor beta subunit	-0.349	0.0395
100158683_TGI_at	*LRRC34*	glutamic--pyruvic transaminase 2	-0.586	0.0395
100122621_TGI_at	*FOXI1*	5-hydroxytryptamine receptor 6	-0.773	0.0397
100305001_TGI_at	*EMID1*	KIAA1456	-0.497	0.0398
100131660_TGI_at	*TNRC6A*	oligodendrocyte lineage transcription factor 2	-0.637	0.0399
100306595_TGI_at	*LCT*	uncharacterized LOC100507291	-0.415	0.0400
100126544_TGI_at	*APOB*	DnaJ heat shock protein family (Hsp40) member B1	-0.458	0.0401
100123633_TGI_at	*LINC01091*	trefoil factor 3	-0.869	0.0403
100306839_TGI_at	*SLC22A15*	centrin 3	-0.322	0.0403
100150127_TGI_at	*CST9*	zinc finger protein 354B	-0.292	0.0404
100151717_TGI_at	*ZNF385B*	ankyrin repeat domain 46	-0.367	0.0406
100130341_TGI_at	*MMD*	origin recognition complex subunit 5	-0.328	0.0407
100146932_TGI_at	*UFSP2*	hydroxysteroid dehydrogenase like 2	-0.480	0.0407
100159162_TGI_at	*RNF6*	homeobox A9	-1.618	0.0407
100303605_TGI_at	*SLC30A4*	serpin family E member 1	-0.969	0.0409
100311732_TGI_at	*DDHD2*	solute carrier organic anion transporter family member 4A1	-0.497	0.0409
100154353_TGI_at	*TCOF1*	integrin subunit alpha 8	-0.927	0.0409
100147719_TGI_at	*MYBPC1*	uncharacterized LOC105374366	-0.251	0.0409
100152539_TGI_at	*SEC22C*	sarcoglycan epsilon	-0.694	0.0410
100309846_TGI_at	*FMNL3*	DnaJ heat shock protein family (Hsp40) member B1	-0.464	0.0411
100162441_TGI_at	*PTCH1*	glucagon receptor	-0.211	0.0413
100161963_TGI_at	*ABHD2*	fibronectin type III domain containing 9	-0.347	0.0413
100301962_TGI_at	*PEG10*	solute carrier family 16 member 10	-0.652	0.0415
100148392_TGI_at	*PHF5A*	rhomboid domain containing 1	-0.438	0.0416
100155445_TGI_at	*SPCS3*	HOXA transcript antisense RNA, myeloid-specific 1	-0.751	0.0417
100148169_TGI_at	*SORCS2*	SRSF protein kinase 2	-0.380	0.0418
100148837_TGI_at	*IGF2R*	coiled-coil domain containing 70	-0.176	0.0418
100301486_TGI_at	*PIRT*	tripartite motif-containing 51	-0.311	0.0418
100139178_TGI_at	*FAM78A*	family with sequence similarity 114 member A2	-0.466	0.0421
100123897_TGI_at	*SAG*	monocyte to macrophage differentiation associated 2	-0.567	0.0423
100132096_TGI_at	*PM20D2*	NK2 homeobox 5	-0.549	0.0423
100313909_TGI_at	*THEG*	RTF1 homolog, Paf1/RNA polymerase II complex component	-0.403	0.0424
100124169_TGI_at	*VGF*	tRNA methyltransferase 10C, mitochondrial RNase P subunit	-0.203	0.0425

100311935_TGI_at	*AASDH*	germ cell associated 1	-0.383	0.0425
100304664_TGI_at	*UFM1*	DLG associated protein 5	-0.212	0.0427
100123989_TGI_at	*ETFBKMT*	urotensin 2	-0.294	0.0428
100149219_TGI_at	*ITPRIPL1*	long intergenic non-protein coding RNA 1004	-0.515	0.0431
100313862_TGI_at	*COBLL1*	semaphorin 3A	-1.040	0.0432
100154070_TGI_at	*VPS11*	dyslexia susceptibility 1 candidate 1	-0.792	0.0433
100127811_TGI_at	*HSPA1A*	dpy-19 like 3 (C. elegans)	-0.439	0.0435
100138220_TGI_at	*C8orf48*	small nucleolar RNA, H/ACA box 80A	-0.322	0.0435
100309030_TGI_at	*GREB1*	guanylate cyclase 1 soluble subunit alpha 2	-0.742	0.0435
100145303_TGI_at	*STC1*	solute carrier organic anion transporter family member 4A1	-0.479	0.0438
100146548_TGI_at	*IKZF4*	intraflagellar transport 22	-0.433	0.0438
100138323_TGI_at	*LRRIQ1*	basic helix-loop-helix domain containing, class B, 9	-0.369	0.0439
100313434_TGI_at	*PCNX3*	DEAD-box helicase 52	-0.218	0.0439
100143128_TGI_at	*CACNA2D4*	transmembrane p24 trafficking protein 4	-0.401	0.0439
100136119_TGI_at	*SKAP2*	Sad1 and UNC84 domain containing 1	-0.479	0.0440
100144936_TGI_at	*CLCN2*	taste 2 receptor member 40	-0.187	0.0441
100127981_TGI_at	*CLSTN3*	coiled-coil domain containing 65	-0.337	0.0441
100147020_TGI_at	*SLC25A34*	fer-1 like family member 4, pseudogene	-0.364	0.0444
100128667_TGI_at	*SCOC-AS1*	copine 4	-0.781	0.0446
100307902_TGI_at	*TMEM53*	DEAD-box helicase 18	-0.256	0.0446
100162988_TGI_at	*LOC105369431*	coiled-coil domain containing 173	-0.748	0.0446
100125248_TGI_at	*IZUMO2*	SIX homeobox 6	-0.686	0.0447

Supplementary Table S6. Differentially expressed genes between MCC primay tumors and metastases in the MCC GEO cohort GSE50451 (cut off p<0.05 & fold change >=1.5)

ID	Gene symbol	Gene title	logFC	*P*-Value
Over-expressed genes in Metastases vs. Primary MCC tumors				
217226_s_at	*SFXN3*	sideroflexin 3	0.614	9E-06
218725_at	*SLC25A22*	solute carrier family 25 member 22	0.532	1.4E-05
220974_x_at	*CNTRL*	sideroflexin 3	0.575	4.1E-05
215349_at	*ALDH3B1*	BTB domain containing 18	0.772	5.8E-05
211004_s_at	*CACNB2*	aldehyde dehydrogenase 3 family member B1	0.514	5.9E-05
215365_at	*KRAS*	calcium voltage-gated channel auxiliary subunit beta 2	0.726	6.4E-05
204010_s_at	*CENPF*	KRAS proto-oncogene, GTPase	0.323	7.2E-05
208342_x_at	*USP1*	chorionic somatomammotropin hormone 2///chorionic somatomammotropin hormone 1	0.910	8.6E-05
205357_s_at	*TRA2B*	angiotensin II receptor type 1	0.240	0.0002
212906_at	*ASMTL*	GRAM domain containing 1B	0.982	0.0002
209394_at	*STAG1*	acetylserotonin O-methyltransferase-like	0.504	0.0003
217912_at	*CRHBP*	dihydrouridine synthase 1 like	0.490	0.0003
205640_at	*SMPDL3B*	aldehyde dehydrogenase 3 family member B1	0.616	0.0003
205984_at	*SEC24D*	corticotropin releasing hormone binding protein	2.066	0.0003
205309_at	*ACO1*	sphingomyelin phosphodiesterase acid like 3B	0.618	0.0004
211508_s_at	*GH1*	growth hormone 2	2.594	0.0004
215209_at	*NPFF*	SEC24 homolog D, COPII coat complex component	0.790	0.0004
36553_at	*HNRNPU*	acetylserotonin O-methyltransferase-like	0.549	0.0004
206886_x_at	*OXLD1*	growth hormone 1	0.983	0.0004
206402_s_at	*ACAT2*	neuropeptide FF-amide peptide precursor	0.584	0.0004
202590_s_at	*PSMD11*	pyruvate dehydrogenase kinase 2	0.362	0.0004
50374_at	*TJP3*	oxidoreductase like domain containing 1	0.475	0.0005
215813_s_at	*BUB1*	prostaglandin-endoperoxide synthase 1	1.637	0.0005
35148_at	*NDC80*	tight junction protein 3	0.727	0.0005
208069_x_at	*SIGIRR*	growth hormone 2///growth hormone 1///chorionic somatomammotropin hormone like 1///chorionic somatomammotropin hormone 1	0.983	0.0005
221797_at	*SRGAP2C///SRGAP2B///SRGAP2*	oxidoreductase like domain containing 1	0.463	0.0006
52940_at	*NCKAP1L*	single Ig and TIR domain containing	1.582	0.0006
205128_x_at	*CSHL1*	prostaglandin-endoperoxide synthase 1	1.517	0.0006
218921_at	*DUSP5*	single Ig and TIR domain containing	1.486	0.0006
217549_at	*CD151*	NCK associated protein 1 like	0.627	0.0007
208294_x_at	*SMPD1*	chorionic somatomammotropin hormone like 1	1.066	0.0007
219550_at	*NEK2*	roundabout guidance receptor 3	1.929	0.0007
211151_x_at	*NR2F1*	growth hormone 1	1.335	0.0008
206885_x_at	*POU2F3*	growth hormone 1	0.900	0.0008

208068_x_at	*NRAS*	growth hormone 2///growth hormone 1///chorionic somatomammotropin hormone like 1///chorionic somatomammotropin hormone 1	0.731	0.0008
49878_at	*VAV1*	peroxisomal biogenesis factor 16	0.337	0.0008
204306_s_at	*MIR631///NEIL1*	CD151 molecule (Raph blood group)	0.654	0.0008
209420_s_at	*METTL10*	sphingomyelin phosphodiesterase 1	0.732	0.0008
218436_at	*BACE1*	SIL1 nucleotide exchange factor	0.393	0.0009
207109_at	*LCT*	POU class 2 homeobox 3	1.190	0.0009
206219_s_at	*RBKS*	vav guanine nucleotide exchange factor 1	1.055	0.0010
216698_x_at	*G3BP1*	olfactory receptor family 7 subfamily E member 12 pseudogene	0.733	0.0010
219396_s_at	*BMP3*	microRNA 631///nei like DNA glycosylase 1	1.043	0.0010
215061_at	*NFIL3*	methyltransferase like 10	0.229	0.0010
218922_s_at	*ELL3*	ceramide synthase 4	0.674	0.0010
217904_s_at	*RGS4*	beta-secretase 1	0.796	0.0011
206945_at	*CSDC2*	lactase	0.806	0.0011
57540_at	*CTNNBIP1*	ribokinase	0.608	0.0011
205840_x_at	*BTBD3*	growth hormone 1	1.024	0.0012
40472_at	*KIAA0101*	lysophosphatidylcholine acyltransferase 4	0.397	0.0012
208244_at	*BARD1*	bone morphogenetic protein 3	0.388	0.0012
203657_s_at	*KIF15*	cathepsin F	0.577	0.0013
219517_at	*LPGAT1*	elongation factor for RNA polymerase II 3	0.979	0.0013
211739_x_at	*SH2D3A*	growth hormone 1///chorionic somatomammotropin hormone like 1///chorionic somatomammotropin hormone 2///chorionic somatomammotropin hormone 1	0.404	0.0013
204337_at	*ZNF329*	regulator of G-protein signaling 4	1.912	0.0013
206195_x_at	*TSC22D4*	growth hormone 2	1.034	0.0013
209981_at	*IL1A*	cold shock domain containing C2	1.055	0.0013
206475_x_at	*ST3GAL4*	growth hormone 1///chorionic somatomammotropin hormone like 1///chorionic somatomammotropin hormone 1	0.434	0.0014
208341_x_at	*BRCA1*	chorionic somatomammotropin hormone 2///chorionic somatomammotropin hormone 1	1.085	0.0014
203081_at	*CHTOP*	catenin beta interacting protein 1	0.638	0.0014
203700_s_at	*RFC4*	deiodinase, iodothyronine, type II	2.089	0.0014
219783_at	*POLA1*	solute carrier family 35 member F6	0.285	0.0015
219513_s_at	*POFUT2*	SH2 domain containing 3A	0.883	0.0016
208104_s_at	*HDHD3*	TSC22 domain family member 4	0.285	0.0016
212465_at	*MMP15*	SET domain containing 3	0.399	0.0016
203759_at	*FXR1*	ST3 beta-galactoside alpha-2,3-sialyltransferase 4	0.666	0.0016
200086_s_at	*ACAA1*	cytochrome c oxidase subunit 4I1	0.361	0.0017
205426_s_at	*EZH2*	huntingtin interacting protein 1	0.381	0.0019
203028_s_at	*DNAJB1*	cytochrome b-245 alpha chain	1.068	0.0019
203150_at	*MPPED1*	Rab9 effector protein with kelch motifs	0.799	0.0019
207449_s_at	*COBLL1*	protein O-fucosyltransferase 2	0.369	0.0019

219709_x_at	*ATG2A*	family with sequence similarity 173 member A	0.672	0.0019
221256_s_at	*NLRP1*	haloacid dehalogenase like hydrolase domain containing 3	0.581	0.0019
203292_s_at	*DESI2*	VPS11, CORVET/HOPS core subunit	0.382	0.0019
203365_s_at	*CASQ2*	matrix metallopeptidase 15	0.506	0.0020
202451_at	*COIL*	general transcription factor IIH subunit 1	0.546	0.0020
214274_s_at	*ARFIP2*	acetyl-CoA acyltransferase 1	0.389	0.0021
203699_s_at	*CDK5R2*	deiodinase, iodothyronine, type II	2.034	0.0021
208505_s_at	*AQP3*	fucosyltransferase 2	0.359	0.0021
218959_at	*TOP2A*	homeobox C10	1.551	0.0021
206436_at	*ZNF467*	metallophosphoesterase domain containing 1	1.222	0.0021
215393_s_at	*PLCE1*	cordon-bleu WH2 repeat protein like 1	0.624	0.0021
213300_at	*ABCB9*	autophagy related 2A	0.483	0.0021
211824_x_at	*EXO1*	NLR family pyrin domain containing 1	0.920	0.0021
207317_s_at	*LOC101927086*	calsequestrin 2	1.231	0.0022
208573_s_at	*EIF1AX*	olfactory receptor family 2 subfamily H member 2	0.302	0.0022
205127_at	*UCP2*	prostaglandin-endoperoxide synthase 1	0.713	0.0022
214944_at	*OR7E37P*	PH domain and leucine rich repeat protein phosphatase 2	0.434	0.0022
213078_x_at	*PPFIA3*	lysophosphatidylcholine acyltransferase 4	0.451	0.0022
202109_at	*GPC4*	ADP ribosylation factor interacting protein 2	0.539	0.0022
204338_s_at	*MFSD5*	regulator of G-protein signaling 4	0.970	0.0023
222169_x_at	*PUS3*	SH2 domain containing 3A	0.313	0.0023
205852_at	*TNFRSF1A*	cyclin dependent kinase 5 regulatory subunit 2	0.607	0.0023
39248_at	*PTBP3*	aquaporin 3 (Gill blood group)	1.183	0.0023
200928_s_at	*OSBPL11*	RAB14, member RAS oncogene family	0.391	0.0023
215641_at	*SNX24*	SEC24 homolog D, COPII coat complex component	0.462	0.0024
213758_at	*ZCCHC14*	cytochrome c oxidase subunit 4I1	0.430	0.0024
214746_s_at	*TNPO3*	zinc finger protein 467	0.949	0.0024
214209_s_at	*FER*	ATP binding cassette subfamily B member 9	0.776	0.0024
202865_at	*AIDA*	DnaJ heat shock protein family (Hsp40) member B12	0.245	0.0025
216489_at	*SPTBN2*	uncharacterized LOC101927086	0.218	0.0025
201017_at	*LOC101930405*	eukaryotic translation initiation factor 1A, X-linked	0.542	0.0025
208998_at	*FLT1*	uncoupling protein 2	0.724	0.0025
59697_at	*MOB1A*	RAB15, member RAS oncogene family	1.185	0.0025
217499_x_at	*LRP5L*	olfactory receptor family 7 subfamily E member 37 pseudogene	0.825	0.0025
213368_x_at	*LOC101929726*	PTPRF interacting protein alpha 3	0.465	0.0025
207205_at	*IGLC1*	carcinoembryonic antigen related cell adhesion molecule 4	0.612	0.0025
39249_at	*ASAP2*	aquaporin 3 (Gill blood group)	0.470	0.0026
211822_s_at	*NMU*	NLR family pyrin domain containing 1	0.733	0.0026

212861_at	*LMNB2*	major facilitator superfamily domain containing 5	0.328	0.0026
221277_s_at	*NDUFV1*	pseudouridylate synthase 3	0.560	0.0027
209368_at	*ATAD2*	epoxide hydrolase 2	0.615	0.0027
207643_s_at	*C1orf112*	TNF receptor superfamily member 1A	1.093	0.0027
212360_at	*NEO1*	adenosine monophosphate deaminase 2	0.779	0.0028
215427_s_at	*POLQ*	zinc finger CCHC-type containing 14	0.341	0.0030
209873_s_at	*TBCE*	plakophilin 3	1.075	0.0030
208806_at	*GABPA*	chromodomain helicase DNA binding protein 3	0.671	0.0030
215112_x_at	*SUZ12*	MCF.2 cell line derived transforming sequence-like 2	0.248	0.0031
205155_s_at	*PHF20*	spectrin beta, non-erythrocytic 2	0.769	0.0031
209872_s_at	*GNAI3*	plakophilin 3	0.424	0.0032
214873_at	*ME3*	LDL receptor related protein 5 like	0.428	0.0032
222278_at	*MYBL1*	uncharacterized LOC101929726	1.240	0.0032
204489_s_at	*C21orf33*	CD44 molecule (Indian blood group)	1.913	0.0033
55872_at	*KIF11*	zinc finger protein 512B	0.412	0.0033
203807_x_at	*LRIG2*	growth hormone 1///chorionic somatomammotropin hormone like 1///chorionic somatomammotropin hormone 2///chorionic somatomammotropin hormone 1	0.321	0.0033
208714_at	*ATP1B3*	NADH:ubiquinone oxidoreductase core subunit V1	0.447	0.0034
206869_at	*CCDC85B*	chondroadherin	0.470	0.0034
211743_s_at	*MTMR2*	proteoglycan 2, pro eosinophil major basic protein	0.842	0.0035
221621_at	*PPARA*	microRNA 6516///small Cajal body-specific RNA 16///small nucleolar RNA host gene 20	0.524	0.0035
219874_at	*KIF23*	solute carrier family 12 member 8	0.668	0.0037
206859_s_at	*PPP2CB*	progestagen associated endometrial protein	0.224	0.0037
217312_s_at	*DLGAP5*	collagen type VII alpha 1 chain	1.475	0.0038
220192_x_at	*SPRY2*	SAM pointed domain containing ETS transcription factor	0.568	0.0039
204663_at	*KRT85*	malic enzyme 3	0.758	0.0040
209456_s_at	*IGLV1-44*	F-box and WD repeat domain containing 11	0.246	0.0040
219222_at	*RRM2*	ribokinase	0.525	0.0041
202217_at	*ZBTB18*	chromosome 21 open reading frame 33	0.376	0.0041
203735_x_at	*PSRC1*	PPFIA binding protein 1	0.636	0.0041
216396_s_at	*RBMS1*	EI24, autophagy associated transmembrane protein	0.459	0.0041
217149_x_at	*EML3*	tyrosine kinase non receptor 1	0.648	0.0041
221810_at	*MCM2*	RAB15, member RAS oncogene family	1.120	0.0042
210955_at	*CUEDC1*	caspase 10	0.197	0.0043
204610_s_at	*CEP55*	coiled-coil domain containing 85B	0.543	0.0043
221991_at	*MFN1*	neurexophilin 3	0.307	0.0043
202084_s_at	*PPFIA2*	SEC14 like lipid binding 1	0.509	0.0044
200085_s_at	*HNRNPA0*	transcription elongation factor B subunit 2	0.284	0.0044
210771_at	*PTRH2*	peroxisome proliferator activated receptor alpha	0.261	0.0044

210819_x_at	*XPO1*	deiodinase, iodothyronine, type II	0.648	0.0045
219518_s_at	*SAFB*	elongation factor for RNA polymerase II 3	1.038	0.0045
221864_at	*SH3YL1*	ORAI calcium release-activated calcium modulator 3	0.571	0.0048
210047_at	*TLE3*	solute carrier family 11 member 2	0.313	0.0048
207321_s_at	*ERCC6L*	ATP binding cassette subfamily B member 9	0.483	0.0048
207670_at	*MCM4*	keratin 85	1.302	0.0049
217928_s_at	*PCDH17*	protein phosphatase 6 regulatory subunit 3	0.305	0.0049
219916_s_at	*CBS*	ring finger protein 39	0.581	0.0049
212063_at	*GRIK1-AS2///BACH1*	CD44 molecule (Indian blood group)	1.616	0.0050
203857_s_at	*NEDD4L*	microRNA 7110///protein disulfide isomerase family A member 5	0.578	0.0050
215127_s_at	*MAP3K12*	RNA binding motif single stranded interacting protein 1	0.481	0.0050
212969_x_at	*ADARB1*	echinoderm microtubule associated protein like 3	0.332	0.0051
202987_at	*SFPQ*	TRAF3 interacting protein 2	0.186	0.0051
219468_s_at	*ASXL3*	CUE domain containing 1	0.530	0.0051
212417_at	*PELI1*	secretory carrier membrane protein 1	0.536	0.0051
221157_s_at	*AKAP13*	F-box protein 24	0.224	0.0053
218072_at	*KCNH2*	COMM domain containing 9	0.441	0.0053
204136_at	*APRT*	collagen type VII alpha 1 chain	1.553	0.0053
218732_at	*LSM14A*	peptidyl-tRNA hydrolase 2	0.357	0.0054
205509_at	*MEX3C*	carboxypeptidase B1	0.233	0.0055
204021_s_at	*CLCN7*	purine rich element binding protein A	0.378	0.0056
205142_x_at	*VDR*	ATP binding cassette subfamily D member 1	0.237	0.0056
204019_s_at	*ZNF536*	SH3 and SYLF domain containing 1	0.768	0.0057
36554_at	*PGM5*	acetylserotonin O-methyltransferase-like	0.432	0.0057
218026_at	*PCDH11Y///PCDH11X*	cytochrome c oxidase assembly factor 3	0.375	0.0059
204697_s_at	*ASB13*	chromogranin A	1.058	0.0060
208357_x_at	*SLC28A3*	chorionic somatomammotropin hormone 1	0.473	0.0060
210818_s_at	*NUSAP1*	GRIK1 antisense RNA 2///BTB domain and CNC homolog 1	0.192	0.0060
212528_at	*RAD23B*	desumoylating isopeptidase 1	0.561	0.0061
205448_s_at	*SERPINE1*	mitogen-activated protein kinase kinase kinase 12	0.564	0.0061
203865_s_at	*KIAA0513*	adenosine deaminase, RNA specific B1	0.807	0.0061
210600_s_at	*NSUN5*	G protein-coupled receptor kinase 4	0.831	0.0062
211975_at	*DDN*	ADP ribosylation factor GTPase activating protein 2	0.404	0.0062
207895_at	*MSRB2*	N-acetylated alpha-linked acidic dipeptidase-like 1	0.702	0.0063
220590_at	*TOLLIP*	integrin alpha FG-GAP repeat containing 2	0.283	0.0064
208324_at	*ARHGAP24*	A-kinase anchoring protein 13	0.217	0.0064
202082_s_at	*THRB*	SEC14 like lipid binding 1	0.392	0.0064
209734_at	*PSKH1*	NCK associated protein 1 like	0.765	0.0065

210036_s_at	*RREB1*	potassium voltage-gated channel subfamily H member 2	0.417	0.0065
213892_s_at	*ANP32E*	adenine phosphoribosyltransferase	0.573	0.0065
213149_at	*RARG*	dihydrolipoamide S-acetyltransferase	0.531	0.0065
221609_s_at	*PARD6A*	Wnt family member 6	0.738	0.0065
205923_at	*RPS6KB2*	reelin	0.941	0.0066
210271_at	*KIF4A*	neuronal differentiation 2	0.906	0.0067
213925_at	*FNDC11*	stum, mechanosensory transduction mediator homolog	1.072	0.0067
205793_x_at	*SDCBP*	tyrosine kinase non receptor 1	0.336	0.0067
212014_x_at	*MIS18BP1*	CD44 molecule (Indian blood group)	1.847	0.0067
220504_at	*HIF1A*	keratocan	0.186	0.0067
38069_at	*PFDN2*	chloride voltage-gated channel 7	0.383	0.0068
204254_s_at	*CXCR4*	vitamin D (1,25- dihydroxyvitamin D3) receptor	0.751	0.0068
210113_s_at	*ST14*	NLR family pyrin domain containing 1	0.722	0.0068
202074_s_at	*CDYL*	optineurin	0.430	0.0069
204589_at	*INTS1*	NUAK family kinase 1	0.947	0.0069
208491_s_at	*ZNF211*	phosphoglucomutase 5	0.353	0.0070
221350_at	*TNFRSF25*	homeobox C8	0.396	0.0070
202942_at	*RWDD2B*	electron transfer flavoprotein beta subunit	0.715	0.0071
206194_at	*SSTR4*	homeobox C4	0.639	0.0072
202793_at	*PAAF1*	lysophosphatidylcholine acyltransferase 3	0.478	0.0072
222279_at	*PTBP1*	HLA-F antisense RNA 1	0.467	0.0072
210619_s_at	*LTBP3*	hyaluronoglucosaminidase 1	0.245	0.0073
204985_s_at	*VEGFA*	trafficking protein particle complex 6A	0.508	0.0073
218862_at	*DCP2*	ankyrin repeat and SOCS box containing 13	0.714	0.0073
220475_at	*SELENBP1*	solute carrier family 28 member 3	0.297	0.0073
205273_s_at	*C1QTNF1*	pitrilysin metallopeptidase 1	0.705	0.0073
203054_s_at	*RNGTT*	T-cell leukemia translocation altered	0.508	0.0073
210469_at	*TADA2A*	discs large MAGUK scaffold protein 5	0.835	0.0074
216860_s_at	*COL9A1*	growth differentiation factor 11	0.674	0.0074
203642_s_at	*MAPK14*	cordon-bleu WH2 repeat protein like 1	0.800	0.0074
202698_x_at	*IGHA2///IGHA1///IGH*	cytochrome c oxidase subunit 4I1	0.311	0.0074
204546_at	*DMD*	KIAA0513	0.489	0.0075
213773_x_at	*CSNK2A1*	NOP2/Sun RNA methyltransferase family member 5	0.357	0.0075
205906_at	*FBXO34*	forkhead box J1	0.697	0.0075
214788_x_at	*SLC13A3*	dendrin	0.620	0.0075
211892_s_at	*WNT10B*	prostaglandin I2 (prostacyclin) synthase	0.213	0.0076
218773_s_at	*SRSF1*	methionine sulfoxide reductase B2	0.826	0.0076
204081_at	*DSCC1*	neurogranin	1.235	0.0077
217930_s_at	*NOX1*	toll interacting protein	0.502	0.0077
208356_x_at	*EIF5*	growth hormone 1///chorionic somatomammotropin hormone like 1///chorionic somatomammotropin hormone 1	0.647	0.0077
207044_at	*EFCAB2*	thyroid hormone receptor beta	0.196	0.0078
209835_x_at	*CCNA2*	CD44 molecule (Indian blood group)	1.780	0.0078
213141_at	*TCEB3*	protein serine kinase H1	0.284	0.0078

204490_s_at	*CX3CL1*	CD44 molecule (Indian blood group)	1.701	0.0078
215620_at	*FBXO5*	ras responsive element binding protein 1	0.333	0.0078
211215_x_at	*TUBB*	deiodinase, iodothyronine, type II	0.920	0.0078
204188_s_at	*SPDL1*	retinoic acid receptor gamma	0.196	0.0079
205245_at	*NBL1*	par-6 family cell polarity regulator alpha	0.360	0.0079
201404_x_at	*AASDHPPT*	proteasome subunit beta 2	0.264	0.0080
208289_s_at	*APBB1*	EI24, autophagy associated transmembrane protein	0.444	0.0080
203777_s_at	*ZNF710*	ribosomal protein S6 kinase B2	0.496	0.0081
64899_at	*KIAA1551*	phospholipid phosphatase related 2	0.344	0.0081
203031_s_at	*PRKDC*	uroporphyrinogen III synthase	0.471	0.0081
215911_x_at	*TP53AIP1*	ATPase plasma membrane Ca2+ transporting 3	0.346	0.0081
220426_at	*RAB40B*	fibronectin type III domain containing 11	0.577	0.0083
202005_at	*CYFIP2*	suppression of tumorigenicity 14	0.934	0.0086
222304_x_at	*ACADSB*	olfactory receptor family 7 subfamily E member 47 pseudogene	1.471	0.0086
209263_x_at	*TBX5*	tetraspanin 4	0.464	0.0086
205470_s_at	*MIR3656///TRAPPC4*	kallikrein related peptidase 11	2.012	0.0086
212212_s_at	*DYRK4*	integrator complex subunit 1	0.644	0.0087
211841_s_at	*ANKRD27*	TNF receptor superfamily member 25	0.763	0.0087
207763_at	*CYP3A43*	S100 calcium binding protein A5	0.222	0.0087
218377_s_at	*INO80B-WBP1///INO80B*	RWD domain containing 2B	0.488	0.0088
214556_at	*MFSD13A*	somatostatin receptor 4	0.229	0.0088
218957_s_at	*DHRS12*	proteasomal ATPase associated factor 1	0.501	0.0088
219922_s_at	*PDPK1*	latent transforming growth factor beta binding protein 3	0.565	0.0089
205447_s_at	*CDCA8*	mitogen-activated protein kinase kinase kinase 12	0.559	0.0089
210130_s_at	*SLC35F6///CENPA*	transmembrane 7 superfamily member 2	0.759	0.0089
214433_s_at	*LRCH4*	selenium binding protein 1	0.998	0.0090
220975_s_at	*ATP5C1*	C1q and tumor necrosis factor related protein 1	0.366	0.0090
222008_at	*ALOX12*	collagen type IX alpha 1 chain	1.738	0.0092
205244_s_at	*CCT6B*	solute carrier family 13 member 3	0.773	0.0093
219165_at	*SMARCD2*	PDZ and LIM domain 2	0.969	0.0093
204339_s_at	*ZNF274*	regulator of G-protein signaling 4	0.931	0.0094
206213_at	*HBA2///HBA1*	Wnt family member 10B	0.292	0.0094
217427_s_at	*KIAA0485*	histone cell cycle regulator	0.606	0.0096
215409_at	*MXRA8*	lysophosphatidylcholine acyltransferase 4	0.277	0.0097
207217_s_at	*APOLD1///DDX47*	NADPH oxidase 1	0.191	0.0097
219246_s_at	*CELF2*	2-oxoglutarate and iron dependent oxygenase domain containing 2	0.370	0.0098
202025_x_at	*FUS*	acetyl-CoA acyltransferase 1	0.265	0.0098

218722_s_at	*HIST2H4B///HIST4H4///HIST2H4A///HIST1H4L///HIST1H4E///HIST1H4B///HIST1H4H///HIST1H4C///HIST1H4J///HIST1H4K///HIST1H4F///HIST1H4D///HIST1H4A///HIST1H4I*	coiled-coil domain containing 51	0.423	0.0098
213604_at	*RELA*	transcription elongation factor B subunit 3	0.356	0.0099
201505_at	*CCNL1*	laminin subunit beta 1	0.878	0.0100
203710_at	*PRKAR1B*	inositol 1,4,5-trisphosphate receptor type 1	0.878	0.0100
217523_at	*MUC1*	CD44 molecule (Indian blood group)	2.138	0.0100
201621_at	*RABEP1*	neuroblastoma 1, DAN family BMP antagonist	0.572	0.0100
202170_s_at	*TNFRSF1B*	aminoadipate-semialdehyde dehydrogenase-phosphopantetheinyl transferase	0.453	0.0101
202652_at	*SOX15*	amyloid beta precursor protein binding family B member 1	0.296	0.0101
39891_at	*ZNF682*	zinc finger protein 710	0.408	0.0101
201681_s_at	*INTS5*	discs large MAGUK scaffold protein 5	0.590	0.0101
212416_at	*ADCY9*	secretory carrier membrane protein 1	0.502	0.0102
220402_at	*PRKCH*	tumor protein p53 regulated apoptosis inducing protein 1	0.445	0.0102
217597_x_at	*IDO1*	RAB40B, member RAS oncogene family	0.365	0.0103
46142_at	*CKS1B*	lipase maturation factor 1	0.584	0.0103
202489_s_at	*KPNA2*	FXYD domain containing ion transport regulator 3	1.215	0.0103
217977_at	*NAA40*	methionine sulfoxide reductase B1	0.606	0.0104
203339_at	*ATP6V0D1*	solute carrier family 25 member 12	0.378	0.0104
201557_at	*WHRN*	vesicle associated membrane protein 2	0.383	0.0105
211615_s_at	*CHPF*	leucine rich pentatricopeptide repeat containing	0.324	0.0105
208888_s_at	*DNAJC13*	nuclear receptor corepressor 2	0.250	0.0105
216407_at	*LOC101060275///NPIPA5///NPIPB5///NPIPB11///LOC613037///NPIPB4///NPIPB3*	Vac14, PIKFYVE complex component	0.216	0.0106
215785_s_at	*ZBTB44*	cytoplasmic FMR1 interacting protein 2	0.391	0.0106
205355_at	*GTF3C1*	acyl-CoA dehydrogenase, short/branched chain	0.599	0.0106
219395_at	*RNF115*	microRNA 6773///epithelial splicing regulatory protein 2	1.250	0.0106
211886_s_at	*RNF123*	T-box 5	0.265	0.0106

217958_at	*SLC7A9*	microRNA 3656///trafficking protein particle complex 4	0.443	0.0106
204547_at	*MAP2K5*	RAB40B, member RAS oncogene family	0.589	0.0108
212954_at	*UPF3A*	dual specificity tyrosine phosphorylation regulated kinase 4	0.403	0.0108
203119_at	*SH3GLB1*	coiled-coil domain containing 86	0.435	0.0108
206405_x_at	*ARF3*	ubiquitin specific peptidase 32///ubiquitin specific peptidase 6	0.832	0.0109
211441_x_at	*TMEM80*	cytochrome P450 family 3 subfamily A member 43	0.231	0.0110
221251_x_at	*GRK6*	INO80B-WBP1 readthrough (NMD candidate)///INO80 complex subunit B	0.352	0.0110
219745_at	*CEMP1///AMDHD2*	major facilitator superfamily domain containing 13A	0.835	0.0110
204800_s_at	*SLC29A3*	dehydrogenase/reductase 12	0.379	0.0110
221244_s_at	*MRPL28*	3-phosphoinositide dependent protein kinase 1	0.245	0.0111
210608_s_at	*NAGPA*	fucosyltransferase 2	0.414	0.0111
203631_s_at	*PPP2R1B*	G protein-coupled receptor class C group 5 member B	0.246	0.0111
210847_x_at	*UBE2G2*	TNF receptor superfamily member 25	0.951	0.0112
201358_s_at	*LMO1*	coatomer protein complex subunit beta 1	0.223	0.0112
90610_at	*SART1*	leucine rich repeats and calponin homology domain containing 4	0.397	0.0113
205243_at	*DHPS*	solute carrier family 13 member 3	0.510	0.0113
204877_s_at	*SMS*	TAO kinase 2	0.365	0.0113
213366_x_at	*MRPL23*	ATP synthase, H+ transporting, mitochondrial F1 complex, gamma polypeptide 1	0.289	0.0113
209816_at	*TAF5L*	patched 1	0.386	0.0113
220383_at	*MAST2*	ATP binding cassette subfamily G member 5	0.946	0.0113
207206_s_at	*RSL1D1*	arachidonate 12-lipoxygenase, 12S type	1.151	0.0113
218685_s_at	*PPP5C*	single-strand-selective monofunctional uracil-DNA glycosylase 1	0.473	0.0113
40665_at	*CENPE*	flavin containing monooxygenase 3	0.433	0.0114
56197_at	*RNF38*	TMEM256-PLSCR3 readthrough (NMD candidate)///phospholipid scramblase 3	0.448	0.0115
206587_at	*C21orf91*	chaperonin containing TCP1 subunit 6B	0.596	0.0115
201827_at	*MIR6743///RIC8A*	SWI/SNF related, matrix associated, actin dependent regulator of chromatin, subfamily d, member 2	0.462	0.0115
215749_s_at	*UBXN2B*	golgi reassembly stacking protein 1	0.643	0.0115
206940_s_at	*GRK3*	POU class 4 homeobox 1	1.502	0.0115
203778_at	*EIF2AK3*	mannosidase beta	0.316	0.0115
206691_s_at	*SLC37A4*	protein disulfide isomerase family A member 2	1.111	0.0116
221701_s_at	*SCML1*	stimulated by retinoic acid 6	1.066	0.0116
204266_s_at	*IL23A*	choline kinase alpha	0.443	0.0116
209950_s_at	*EXT2*	villin like	0.649	0.0116
213422_s_at	*GLUD1*	matrix remodeling associated 8	0.816	0.0117
220173_at	*SIGMAR1*	basal body orientation factor 1	0.506	0.0117
200752_s_at	*KLHL12*	calpain 1	0.474	0.0120

204470_at	*ADGRL1*	C-X-C motif chemokine ligand 1	0.791	0.0120
201783_s_at	*TMEM134*	RELA proto-oncogene, NF-kB subunit	0.341	0.0121
200923_at	*GPS1*	galectin 3 binding protein	0.460	0.0121
219245_s_at	*PARP2*	2-oxoglutarate and iron dependent oxygenase domain containing 2	0.318	0.0122
212555_at	*NOTCH2*	protein kinase cAMP-dependent type I regulatory subunit beta	0.218	0.0123
202493_x_at	*HMGN4*	growth hormone 1///chorionic somatomammotropin hormone like 1///chorionic somatomammotropin hormone 1	0.255	0.0123
202831_at	*GOT2*	glutathione peroxidase 2	1.848	0.0124
211695_x_at	*PLPP1*	mucin 1, cell surface associated	1.254	0.0124
203223_at	*HOXC6*	rabaptin, RAB GTPase binding effector protein 1	0.545	0.0124
203508_at	*TUBGCP3*	TNF receptor superfamily member 1B	0.833	0.0125
206122_at	*GPR137*	SRY-box 15	0.590	0.0125
53968_at	*FARP1*	integrator complex subunit 5	0.357	0.0125
204497_at	*KCNA5*	adenylate cyclase 9	0.599	0.0127
214404_x_at	*ADAMTS1*	SAM pointed domain containing ETS transcription factor	0.353	0.0128
217860_at	*NFX1*	NADH:ubiquinone oxidoreductase subunit A10	0.643	0.0128
213946_s_at	*CCNB1*	obscurin like 1	0.548	0.0129
222369_at	*TFAP2A*	N(alpha)-acetyltransferase 40, NatD catalytic subunit	0.392	0.0130
221604_s_at	*CBR4*	peroxisomal biogenesis factor 16	0.375	0.0130
212041_at	*FA2H*	ATPase H+ transporting V0 subunit d1	0.380	0.0130
205750_at	*PLK1*	biphenyl hydrolase like	0.304	0.0131
221887_s_at	*DNAJC4*	whirlin	0.569	0.0132
202175_at	*PTGER3*	chondroitin polymerizing factor	0.288	0.0132
211996_s_at	*ABI2*	putative NPIP-like protein LOC613037///nuclear pore complex interacting protein family member A5///nuclear pore complex interacting protein family member B5///nuclear pore complex interacting protein family member B11///nuclear pore complex interacting protein member///nuclear pore complex interacting protein family member B4///nuclear pore complex interacting protein family member B3	0.281	0.0133
220243_at	*NPTX1*	zinc finger and BTB domain containing 44	0.496	0.0133
202320_at	*UBXN7*	general transcription factor IIIC subunit 1	0.305	0.0133
201019_s_at	*KPNA4*	eukaryotic translation initiation factor 1A, X-linked	0.317	0.0134
221063_x_at	*MIR6716///PHLDB1*	ring finger protein 123	0.316	0.0134
221869_at	*C21orf2*	zinc finger protein 512B	0.360	0.0134
216765_at	*FKBP15*	mitogen-activated protein kinase kinase 5	0.535	0.0134
217596_at	*RANBP9*	UPF3 regulator of nonsense transcripts homolog A (yeast)	0.485	0.0134
207155_at	*SNRPB*	T-box 5	0.524	0.0136

204990_s_at	*MFN2*	integrin subunit beta 4	0.589	0.0137
206185_at	*N4BP3*	crystallin beta B1	0.263	0.0137
200011_s_at	*RAP1GAP*	ADP ribosylation factor 3	0.413	0.0137
209364_at	*RXRA*	BCL2 associated agonist of cell death	0.404	0.0137
221951_at	*TGFA*	transmembrane protein 80	0.569	0.0137
211543_s_at	*TESK1*	G protein-coupled receptor kinase 6	0.335	0.0138
219082_at	*GINS1*	cementum protein 1///amidohydrolase domain containing 2	0.320	0.0138
219344_at	*BASP1*	solute carrier family 29 member 3	0.330	0.0138
203641_s_at	*TNF*	cordon-bleu WH2 repeat protein like 1	0.603	0.0138
204599_s_at	*FES*	mitochondrial ribosomal protein L28	0.329	0.0138
205090_s_at	*ACAN*	N-acetylglucosamine-1-phosphodiester alpha-N-acetylglucosaminidase	0.404	0.0139
202883_s_at	*COMT*	protein phosphatase 2 scaffold subunit Abeta	0.432	0.0140
219423_x_at	*TMEM41B*	TNF receptor superfamily member 25	0.955	0.0141
219610_at	*CEP72*	Rho guanine nucleotide exchange factor 28	0.957	0.0141
218146_at	*MGC40069///TRAC///TRAJ17///TRAV20///TRDV2///YME1L1*	glycosyltransferase 8 domain containing 1	0.294	0.0141
203747_at	*TCF3*	aquaporin 3 (Gill blood group)	0.369	0.0141
209042_s_at	*DLG1*	ubiquitin conjugating enzyme E2 G2	0.498	0.0141
206718_at	*LARS*	LIM domain only 1	0.989	0.0143
200051_at	*PPP3CA*	squamous cell carcinoma antigen recognized by T-cells 1	0.375	0.0143
213897_s_at	*NDUFS3*	mitochondrial ribosomal protein L23	0.452	0.0145
202545_at	*CEP164*	protein kinase C delta	0.450	0.0146
212019_at	*LOC101929087///SUMO2///SUMO3*	ribosomal L1 domain containing 1	0.484	0.0146
211323_s_at	*MGEA5*	inositol 1,4,5-trisphosphate receptor type 1	0.597	0.0146
214674_at	*SLC35A2*	ubiquitin specific peptidase 19	0.279	0.0147
210916_s_at	*CRLF1*	CD44 molecule (Indian blood group)	1.531	0.0148
221637_s_at	*MAP3K11*	chromosome 11 open reading frame 98///LBH domain containing 1	0.330	0.0149
221647_s_at	*SAP30*	microRNA 6743///RIC8 guanine nucleotide exchange factor A	0.397	0.0150
214045_at	*PSMD13*	lipoic acid synthetase	0.454	0.0150
204773_at	*ERP44*	interleukin 11 receptor subunit alpha	0.607	0.0150
204184_s_at	*NR2F2///NR2F1*	G protein-coupled receptor kinase 3	0.542	0.0150
207131_x_at	*B3GALT2*	gamma-glutamyltransferase 2///gamma-glutamyltransferase light chain 1///gamma-glutamyltransferase light chain 2///gamma-glutamyltransferase 1	0.265	0.0151
202830_s_at	*EPS8L2*	solute carrier family 37 member 4	0.340	0.0151
216857_at	*GMPPA*	interleukin 23 subunit alpha	0.219	0.0151
202013_s_at	*NDST1*	exostosin glycosyltransferase 2	0.331	0.0151

209235_at	*ZNF235*	chloride voltage-gated channel 7	0.320	0.0151
200947_s_at	*AKR1C1*	glutamate dehydrogenase 1	0.428	0.0152
219945_at	*TRIM66*	DEAD-box helicase 25	1.042	0.0152
39854_r_at	*DNMT1*	patatin like phospholipase domain containing 2	0.242	0.0152
201692_at	*SMAGP*	sigma non-opioid intracellular receptor 1	0.328	0.0153
221596_s_at	*DDX3X*	RNA binding motif protein 48	0.371	0.0153
214744_s_at	*CCDC40*	small nucleolar RNA, H/ACA box 21///ribosomal protein L23	0.480	0.0153
218828_at	*RACGAP1*	TMEM256-PLSCR3 readthrough (NMD candidate)///phospholipid scramblase 3	0.449	0.0154
47560_at	*PRSS53*	adhesion G protein-coupled receptor L1	0.462	0.0154
204534_at	*TMEM53*	SEBOX homeobox///vitronectin	0.224	0.0155
218531_at	*TERF2IP*	transmembrane protein 134	0.566	0.0155
202476_s_at	*SOX18*	tubulin gamma complex associated protein 2	0.289	0.0155
217782_s_at	*LSM6*	G protein pathway suppressor 1	0.334	0.0155
208997_s_at	*RHBDD3*	uncoupling protein 2	0.696	0.0155
202104_s_at	*CDV3*	uncharacterized LOC101930112///SPG7, paraplegin matrix AAA peptidase subunit	0.517	0.0156
219263_at	*PPP4R1*	ring finger protein 128, E3 ubiquitin protein ligase	1.553	0.0156
221867_at	*DGKZ*	NEDD4 binding protein 1	0.407	0.0156
208365_s_at	*PKP1*	G protein-coupled receptor kinase 4	0.711	0.0157
218734_at	*RASA4B///RASA4CP///RASA4*	N(alpha)-acetyltransferase 40, NatD catalytic subunit	0.460	0.0157
220465_at	*TMEM47*	CEBPA antisense RNA 1 (head to head)	0.201	0.0158
200708_at	*ZFR2*	glutamic-oxaloacetic transaminase 2	0.392	0.0159
206858_s_at	*PTDSS2*	homeobox C6	1.286	0.0159
43934_at	*LCMT1*	G protein-coupled receptor 137	0.221	0.0160
206762_at	*NISCH*	potassium voltage-gated channel subfamily A member 5	0.642	0.0161
217625_x_at	*TMEM8A*	long intergenic non-protein coding RNA 963	0.323	0.0161
210300_at	*HSPA1B///HSPA1A*	RRAD and GEM like GTPase 1	0.295	0.0161
210268_at	*SLC23A2*	nuclear transcription factor, X-box binding 1	0.558	0.0162
215355_at	*WWOX*	POU class 2 homeobox 3	0.624	0.0164
204654_s_at	*TBC1D31*	transcription factor AP-2 alpha	1.023	0.0165
213626_at	*FBXO2*	carbonyl reductase 4	0.586	0.0165
219429_at	*CNKSR1*	fatty acid 2-hydroxylase	1.146	0.0165
209365_s_at	*DR1*	extracellular matrix protein 1	0.654	0.0166
201600_at	*HRAS*	prohibitin 2	0.316	0.0166
206782_s_at	*HNRNPM*	DnaJ heat shock protein family (Hsp40) member C4	0.540	0.0168
208169_s_at	*SLC29A1*	prostaglandin E receptor 3	0.200	0.0168
212134_at	*HMGN1*	microRNA 6716///pleckstrin homology like domain family B member 1	0.510	0.0171
203994_s_at	*TFF2*	chromosome 21 open reading frame 2	0.343	0.0171
219451_at	*HSPBAP1*	methionine sulfoxide reductase B2	0.719	0.0175
208837_at	*ENPP2*	transmembrane p24 trafficking protein 3	0.407	0.0175

201155_s_at	*DOPEY1*	mitofusin 2	0.399	0.0177
214775_at	*COL21A1*	NEDD4 binding protein 3	0.194	0.0177
203911_at	*MYO1C*	RAP1 GTPase activating protein	1.198	0.0177
205016_at	*TAF7*	transforming growth factor alpha	0.397	0.0177
204106_at	*CISD1*	testis-specific kinase 1	0.316	0.0178
207113_s_at	*APEH*	tumor necrosis factor	0.605	0.0179
216794_at	*SLC29A2*	immunoglobin superfamily member 21 pseudogene	0.233	0.0179
205418_at	*ANKFY1*	FES proto-oncogene, tyrosine kinase	0.867	0.0179
207692_s_at	*KLRA1P*	aggrecan	0.300	0.0179
209124_at	*CWC25*	myeloid differentiation primary response 88	0.386	0.0179
208818_s_at	*CASK*	catechol-O-methyltransferase	0.336	0.0180
212622_at	*GATA2*	transmembrane protein 41B	0.377	0.0180
211282_x_at	*FEM1C*	TNF receptor superfamily member 25	0.736	0.0181
217810_x_at	*OGFOD3*	leucyl-tRNA synthetase	0.403	0.0181
202425_x_at	*ACSF2*	protein phosphatase 3 catalytic subunit alpha	0.318	0.0182
207111_at	*CHEK2*	adhesion G protein-coupled receptor E1	0.272	0.0182
203234_at	*ABO*	uridine phosphorylase 1	0.927	0.0182
203337_x_at	*MTMR14*	integrin subunit beta 1 binding protein 1	0.664	0.0182
201740_at	*NF2*	NADH:ubiquinone oxidoreductase core subunit S3	0.351	0.0182
201253_s_at	*GLS2*	CDP-diacylglycerol--inositol 3-phosphatidyltransferase	0.386	0.0182
200647_x_at	*ZNF24*	eukaryotic translation initiation factor 3 subunit C-like///eukaryotic translation initiation factor 3 subunit C	0.306	0.0182
211341_at	*CHD7*	POU class 4 homeobox 1	1.362	0.0183
208033_s_at	*SLITRK5*	zinc finger homeobox 3	0.245	0.0183
204251_s_at	*PIGG*	centrosomal protein 164	0.408	0.0183
208988_at	*ATP13A2*	lysine demethylase 2A	0.369	0.0184
209820_s_at	*PPP1R11*	transducin beta like 3	0.564	0.0184
207439_s_at	*ABL1*	solute carrier family 35 member A2	0.449	0.0185
218287_s_at	*WLS*	argonaute 1, RISC catalytic component	0.411	0.0186
221048_x_at	*CYB561*	chromosome 17 open reading frame 80	0.225	0.0187
203652_at	*GPR135*	mitogen-activated protein kinase kinase kinase 11	0.321	0.0187
47553_at	*LAMA2*	whirlin	0.919	0.0188
209253_at	*SUB1*	sorbin and SH3 domain containing 3	0.349	0.0188
201474_s_at	*SERGEF*	integrin subunit alpha 3	0.388	0.0189
201232_s_at	*SNIP1*	proteasome 26S subunit, non-ATPase 13	0.369	0.0189
203861_s_at	*ERAP2*	actinin alpha 2	0.511	0.0190
214440_at	*LAMP5*	N-acetyltransferase 1	0.649	0.0191
216905_s_at	*CDH11*	suppression of tumorigenicity 14	1.237	0.0192
218180_s_at	*DNM1*	EPS8 like 2	0.922	0.0192
220136_s_at	*SAE1*	crystallin beta A2	1.334	0.0192
207847_s_at	*ADD3*	mucin 1, cell surface associated	1.934	0.0193
218070_s_at	*PLCL1*	GDP-mannose pyrophosphorylase A	0.498	0.0193
216230_x_at	*EIF6*	sphingomyelin phosphodiesterase 1	0.339	0.0194
202608_s_at	*METTL18*	N-deacetylase and N-sulfotransferase 1	0.178	0.0194

217626_at	*DHX8*	aldo-keto reductase family 1 member C1	0.252	0.0195
213748_at	*PHIP*	tripartite motif containing 66	0.604	0.0195
201570_at	*ZMIZ2*	SAMM50 sorting and assembly machinery component	0.325	0.0196
65630_at	*MAGEL2*	transmembrane protein 80	0.659	0.0196
211036_x_at	*ABTB2*	anaphase promoting complex subunit 5	0.330	0.0196
209868_s_at	*BAG1*	RNA binding motif single stranded interacting protein 1	0.439	0.0197
220592_at	*LY6G6C*	coiled-coil domain containing 40	0.437	0.0197
218314_s_at	*DTNA*	chromosome 11 open reading frame 57	0.375	0.0198
204981_at	*C7orf25///PSMA2*	solute carrier family 22 member 18	0.387	0.0198
214818_at	*DNAJA3*	coiled-coil domain containing 57	0.243	0.0198
204424_s_at	*LOC101927673///OTUB1*	LIM domain only 3	1.405	0.0199
220604_x_at	*TECR*	formimidoyltransferase cyclodeaminase	0.294	0.0200
214226_at	*IGHM*	protease, serine 53	0.455	0.0200
220440_at	*BMP8A*	galectin 13	0.184	0.0200
219462_at	*BATF3*	transmembrane protein 53	0.530	0.0201
201174_s_at	*SLC25A6*	TERF2 interacting protein	0.309	0.0201
219568_x_at	*TCF7L2*	SRY-box 18	0.467	0.0201
215205_x_at	*DIAPH2*	nuclear receptor corepressor 2	0.219	0.0202
217622_at	*RFPL2*	rhomboid domain containing 3	0.231	0.0202
221546_at	*KCNIP1*	pre-mRNA processing factor 18	0.199	0.0203
207556_s_at	*ZDHHC3*	diacylglycerol kinase zeta	0.574	0.0203
205295_at	*DYNLL1*	creatine kinase, mitochondrial 2	0.314	0.0204
201569_s_at	*SMARCE1*	SAMM50 sorting and assembly machinery component	0.365	0.0204
220319_s_at	*DMC1*	myosin regulatory light chain interacting protein	0.434	0.0204
213693_s_at	*MAPRE2*	mucin 1, cell surface associated	1.604	0.0205
221854_at	*FAM89B*	plakophilin 1	0.991	0.0206
212053_at	*TSSC4*	pyridoxal-dependent decarboxylase domain-containing protein 1///pyridoxal dependent decarboxylase domain containing 1	0.372	0.0206
212707_s_at	*PRC1*	RAS p21 protein activator 4B///RAS p21 protein activator 4C, pseudogene///RAS p21 protein activator 4	0.553	0.0206
215419_at	*ZNF174*	zinc finger RNA binding protein 2	0.383	0.0206
210252_s_at	*UBXN1*	MAP kinase activating death domain	0.456	0.0206
221005_s_at	*NQO2*	phosphatidylserine synthase 2	0.354	0.0206
203748_x_at	*ZRSR2*	RNA binding motif single stranded interacting protein 1	0.420	0.0207
221515_s_at	*PEX14*	leucine carboxyl methyltransferase 1	0.269	0.0207
34260_at	*PEAK1*	telomere maintenance 2	0.402	0.0208
213670_x_at	*TUBA4A*	NOP2/Sun RNA methyltransferase family member 5 pseudogene 1	0.435	0.0208
201591_s_at	*INPP5F*	nischarin	0.427	0.0209
221882_s_at	*PTAFR*	transmembrane protein 8A	0.421	0.0209
209237_s_at	*PICK1*	solute carrier family 23 member 2	0.256	0.0209

202740_at	*PRIM2B///PRIM2*	ABHD14A-ACY1 readthrough///aminoacylase 1	0.447	0.0210
201175_at	*DYSF*	thioredoxin related transmembrane protein 2	0.436	0.0210
207713_s_at	*CYP3A7-CYP3A51P*	RANBP2-type and C3HC4-type zinc finger containing 1	0.338	0.0210
219077_s_at	*PEX1*	WW domain containing oxidoreductase	0.311	0.0210
218786_at	*PIGQ*	5'-nucleotidase domain containing 3	0.660	0.0210
210949_s_at	*MST1R*	eukaryotic translation initiation factor 3 subunit C-like///eukaryotic translation initiation factor 3 subunit C	0.297	0.0211
219305_x_at	*KRT24*	F-box protein 2	0.600	0.0211
204740_at	*GOSR2*	connector enhancer of kinase suppressor of Ras 1	0.689	0.0211
215381_at	*MAN1B1*	mechanistic target of rapamycin	0.234	0.0212
220443_s_at	*PARP1*	ventral anterior homeobox 2	0.713	0.0212
212983_at	*PRDX6*	HRas proto-oncogene, GTPase	0.557	0.0212
201802_at	*IGHM///IGHG1*	solute carrier family 29 member 1 (Augustine blood group)	0.521	0.0212
35626_at	*SMAD2*	N-sulfoglucosamine sulfohydrolase	0.275	0.0212
219405_at	*SPATA2L*	tripartite motif containing 68	0.420	0.0214
218623_at	*OSBPL9*	HMP19 protein	1.217	0.0215
209326_at	*GFRA2*	solute carrier family 35 member A2	0.667	0.0215
203931_s_at	*ZBTB3*	mitochondrial ribosomal protein L12	0.371	0.0215
214235_at	*CHST8*	cytochrome P450 family 3 subfamily A member 5	1.021	0.0216
214476_at	*H1FX*	trefoil factor 2	0.176	0.0216
206196_s_at	*RAB22A*	RUN domain containing 3A	0.662	0.0216
213964_x_at	*SYMPK*	RPARP antisense RNA 1	0.407	0.0217
206231_at	*INPPL1*	potassium calcium-activated channel subfamily N member 1	0.380	0.0217
203115_at	*TMED5*	ferrochelatase	0.291	0.0217
219284_at	*CHSY1*	HSPB1 associated protein 1	0.343	0.0218
217730_at	*BCAT2*	microRNA 6513///transmembrane BAX inhibitor motif containing 1	0.572	0.0218
221090_s_at	*PIR*	2-oxoglutarate and iron dependent oxygenase domain containing 1	0.372	0.0219
40612_at	*SEC14L1P1*	dopey family member 1	0.495	0.0219
202866_at	*MXRA5*	DnaJ heat shock protein family (Hsp40) member B12	0.259	0.0219
214656_x_at	*STUB1*	myosin IC	0.236	0.0219
218597_s_at	*ENO1*	CDGSH iron sulfur domain 1	0.431	0.0221
204567_s_at	*CCNB2*	ATP binding cassette subfamily G member 1	0.826	0.0222
201284_s_at	*ZNF215*	acylaminoacyl-peptide hydrolase	0.441	0.0223
204717_s_at	*CA11*	solute carrier family 29 member 2	0.573	0.0223
213412_at	*CTPS2*	tight junction protein 3	0.440	0.0225
219868_s_at	*PCNX4*	ankyrin repeat and FYVE domain containing 1	0.274	0.0225
207229_at	*PPFIA1*	killer cell lectin like receptor A1, pseudogene	0.270	0.0226
200098_s_at	*ARNTL*	anaphase promoting complex subunit 5	0.317	0.0228

219543_at	*KNTC1*	phenazine biosynthesis like protein domain containing	0.606	0.0229
217969_at	*LIG1*	VPS51, GARP complex subunit	0.478	0.0230
53071_s_at	*HAMP*	2-oxoglutarate and iron dependent oxygenase domain containing 3	0.406	0.0231
218844_at	*GDAP1*	acyl-CoA synthetase family member 2	0.474	0.0231
216929_x_at	*LRRK1*	ABO blood group (transferase A, alpha 1-3-N-acetylgalactosaminyltransferase; transferase B, alpha 1-3-galactosyltransferase)	0.234	0.0231
217728_at	*CSPP1*	S100 calcium binding protein A6	0.751	0.0232
222143_s_at	*PSEN1*	myotubularin related protein 14	0.209	0.0233
221966_at	*SOX11*	G protein-coupled receptor 137	0.326	0.0233
217150_s_at	*MLXIPL*	neurofibromin 2	0.579	0.0233
205531_s_at	*GFRA1*	glutaminase 2	0.871	0.0233
205262_at	*CDK1*	potassium voltage-gated channel subfamily H member 2	0.444	0.0234
218652_s_at	*CACFD1*	phosphatidylinositol glycan anchor biosynthesis class G	0.328	0.0236
203683_s_at	*GALNT6*	vascular endothelial growth factor B	0.314	0.0237
217869_at	*ZNF45*	hydroxysteroid 17-beta dehydrogenase 12	0.379	0.0237
218608_at	*METTL4*	ATPase 13A2	0.427	0.0237
218120_s_at	*ESRRA*	heme oxygenase 2	0.280	0.0237
201500_s_at	*CAMSAP2*	protein phosphatase 1 regulatory inhibitor subunit 11	0.312	0.0237
213035_at	*MCF2L*	ankyrin repeat domain 28	0.644	0.0238
205666_at	*LOC101929777///WNT3*	flavin containing monooxygenase 1	0.521	0.0238
216161_at	*GALT*	strawberry notch homolog 1	0.228	0.0240
219046_s_at	*GLB1L2*	PBX/knotted 1 homeobox 2	0.261	0.0240
214100_x_at	*CDC25B*	NOP2/Sun RNA methyltransferase family member 5 pseudogene 1	0.402	0.0240
209164_s_at	*GABPB1*	cytochrome b561	0.538	0.0241
200824_at	*DNASE1L2*	glutathione S-transferase pi 1	0.783	0.0241
216840_s_at	*KBTBD2*	laminin subunit alpha 2	0.395	0.0241
214601_at	*DCBLD2*	tryptophan hydroxylase 1	0.616	0.0242
33494_at	*ATP6V0C*	electron transfer flavoprotein dehydrogenase	0.458	0.0242
205425_at	*ZNF227*	huntingtin interacting protein 1	0.904	0.0243
220482_s_at	*FLOT2*	secretion regulating guanine nucleotide exchange factor	0.360	0.0244
34206_at	*RTN3*	ArfGAP with RhoGAP domain, ankyrin repeat and PH domain 1	0.327	0.0244
205689_at	*NRBP1*	pecanex homolog 2 (Drosophila)	0.822	0.0246
203878_s_at	*HDAC7*	matrix metallopeptidase 11	0.637	0.0246
219692_at	*CBX6*	kringle containing transmembrane protein 2	0.439	0.0247
203397_s_at	*INPP5B*	polypeptide N-acetylgalactosaminyltransferase 3	0.897	0.0247
202488_s_at	*RIC3*	FXYD domain containing ion transport regulator 3	0.437	0.0247
35179_at	*KLF12*	beta-1,3-glucuronyltransferase 3	0.423	0.0247

207172_s_at	*TAF9B*	cadherin 11	0.941	0.0248
215116_s_at	*MARCKS*	dynamin 1	0.659	0.0249
210213_s_at	*DONSON*	eukaryotic translation initiation factor 6	0.286	0.0249
54970_at	*SLC16A5*	zinc finger MIZ-type containing 2	0.341	0.0251
219894_at	*CORIN*	MAGE family member L2	0.205	0.0251
210861_s_at	*SIRT3*	WNT1 inducible signaling pathway protein 3	1.055	0.0251
209221_s_at	*POSTN*	oxysterol binding protein like 2	0.268	0.0252
209702_at	*DCAF7*	fat mass and obesity associated	0.289	0.0252
207986_x_at	*SQLE*	cytochrome b561	0.272	0.0252
211475_s_at	*RGL3///EPOR*	BCL2 associated athanogene 1	0.343	0.0253
208817_at	*SETD1B*	catechol-O-methyltransferase	0.246	0.0253
207114_at	*RCOR3*	lymphocyte antigen 6 complex, locus G6C	0.198	0.0254
53202_at	*CACNA1E*	chromosome 7 open reading frame 25///proteasome subunit alpha 2	0.282	0.0254
205963_s_at	*GOLGA2*	DnaJ heat shock protein family (Hsp40) member A3	0.321	0.0254
214175_x_at	*MSH5-SAPCD1///SAPCD1*	PDZ and LIM domain 4	0.627	0.0255
214883_at	*STAG2*	thyroid hormone receptor, alpha	0.181	0.0255
219057_at	*PRKCB*	rabaptin, RAB GTPase binding effector protein 2	0.269	0.0256
38710_at	*SEC23A*	uncharacterized LOC101927673///OTU deubiquitinase, ubiquitin aldehyde binding 1	0.261	0.0257
220203_at	*CNIH3*	bone morphogenetic protein 8a	0.274	0.0258
208722_s_at	*RINT1*	anaphase promoting complex subunit 5	0.377	0.0258
212826_s_at	*PCSK7*	solute carrier family 25 member 6	0.373	0.0259
218169_at	*MDM1*	Vac14, PIKFYVE complex component	0.253	0.0260
207227_x_at	*TTC27*	ret finger protein like 2	0.300	0.0260
37117_at	*WTH3DI///RAB6A*	PRR5-ARHGAP8 readthrough///Rho GTPase activating protein 8	0.997	0.0260
214296_x_at	*SOX4*	IZUMO family member 4	0.247	0.0261
221307_at	*MAT2B*	potassium voltage-gated channel interacting protein 1	0.181	0.0262
218078_s_at	*HNRNPUL2-BSCL2///BSCL2*	zinc finger DHHC-type containing 3	0.340	0.0262
208386_x_at	*MYCT1*	DNA meiotic recombinase 1	0.356	0.0265
209222_s_at	*DERL1*	oxysterol binding protein like 2	0.343	0.0265
204370_at	*LETM1*	cleavage and polyadenylation factor I subunit 1	0.309	0.0265
213489_at	*ITPK1*	microtubule associated protein RP/EB family member 2	0.326	0.0266
212484_at	*RFNG*	family with sequence similarity 89 member B	0.328	0.0266
218612_s_at	*MACROD1*	tumor suppressing subtransferable candidate 4	0.302	0.0266
212735_at	*IGKC*	RUN and cysteine rich domain containing beclin 1 interacting protein	0.465	0.0266
210290_at	*IL6ST*	zinc finger protein 174	0.379	0.0267
210623_at	*NRP1*	UBX domain protein 1	0.310	0.0267

213542_at	*NPR3*	zinc finger protein 710	0.256	0.0267
207911_s_at	*BSPRY*	transglutaminase 5	0.269	0.0269
213876_x_at	*MRPL39*	zinc finger CCCH-type, RNA binding motif and serine/arginine rich 2	0.328	0.0269
202012_s_at	*PTTG1*	exostosin glycosyltransferase 2	0.293	0.0269
33760_at	*TNC*	peroxisomal biogenesis factor 14	0.283	0.0270
36612_at	*MIR6890///QARS*	family with sequence similarity 168 member A	0.360	0.0270
220008_at	*ZNF213-AS1*	pseudopodium enriched atypical kinase 1	0.197	0.0270
221818_at	*ATP6V0E1*	integrator complex subunit 5	0.320	0.0270
212242_at	*TIAM1*	tubulin alpha 4a	0.470	0.0272
206103_at	*NFATC1*	ras-related C3 botulinum toxin substrate 3 (rho family, small GTP binding protein Rac3)	0.325	0.0273
208382_s_at	*ARMC8*	DNA meiotic recombinase 1	0.647	0.0273
203607_at	*CD47*	inositol polyphosphate-5-phosphatase F	0.981	0.0273
219430_at	*CYP1B1*	G protein-coupled receptor 137	0.323	0.0273
211661_x_at	*DAZAP1*	platelet activating factor receptor	0.264	0.0274
207628_s_at	*AP1S1*	Williams-Beuren syndrome chromosome region 22	0.290	0.0274
209578_s_at	*TTLL7*	protein O-fucosyltransferase 2	0.563	0.0274
202335_s_at	*ATG9A*	ubiquitin conjugating enzyme E2 B	0.190	0.0274
65770_at	*MLX*	ras homolog family member T2	0.337	0.0275
204746_s_at	*CLDN7*	protein interacting with PRKCA 1	0.232	0.0275
218475_at	*FEV*	tRNA methyltransferase 2 homolog A	0.286	0.0275
214802_at	*SLC38A7*	exocyst complex component 7	0.411	0.0275
218660_at	*SPAG1*	dysferlin	0.472	0.0276
218189_s_at	*PCM1*	N-acetylneuraminate synthase	0.328	0.0276
211843_x_at	*SORBS1*	CYP3A7-CYP3A51P readthrough	0.279	0.0276
215280_s_at	*CBL*	PTPRF interacting protein alpha 3	0.440	0.0276
204873_at	*NENF*	peroxisomal biogenesis factor 1	0.336	0.0277
204255_s_at	*P4HTM*	vitamin D (1,25- dihydroxyvitamin D3) receptor	0.687	0.0277
204144_s_at	*PHF1*	phosphatidylinositol glycan anchor biosynthesis class Q	0.335	0.0277
206842_at	*S100A3*	potassium voltage-gated channel subfamily D member 1	0.298	0.0278
205455_at	*CCKBR*	macrophage stimulating 1 receptor	0.762	0.0279
213778_x_at	*IMPAD1*	zinc finger protein 276	0.415	0.0281
215411_s_at	*HNRNPH1*	TRAF3 interacting protein 2	0.328	0.0281
220267_at	*SLC25A37*	keratin 24	0.468	0.0282
203802_x_at	*ZFP37*	NOP2/Sun RNA methyltransferase family member 5	0.290	0.0283
213206_at	*SSX4B///SSX4*	golgi SNAP receptor complex member 2	0.281	0.0283
218636_s_at	*AKAP11*	mannosidase alpha class 1B member 1	0.316	0.0284
201391_at	*TDP1*	TNF receptor associated protein 1	0.429	0.0284
203075_at	*HSPB11*	SMAD family member 2	0.351	0.0284
214965_at	*NSUN5P2///NSUN5P1*	spermatogenesis associated 2 like	0.343	0.0285
204329_s_at	*TRAM1*	zinc finger protein 202	0.325	0.0286

212996_s_at	*IER3*	URB1 ribosome biogenesis 1 homolog (S. cerevisiae)	0.356	0.0286
218047_at	*ATP2B2*	oxysterol binding protein like 9	0.298	0.0286
205721_at	*HMGB3P1*	GDNF family receptor alpha 2	0.462	0.0286
220391_at	*CREM*	zinc finger and BTB domain containing 3	0.300	0.0286
1431_at	*KMT2A*	cytochrome P450 family 2 subfamily E member 1	0.496	0.0286
210406_s_at	*ATP10B*	RAB6C-like///RAB6C, member RAS oncogene family///RAB6A, member RAS oncogene family	0.277	0.0286
221065_s_at	*MADCAM1*	carbohydrate sulfotransferase 8	0.557	0.0286
218673_s_at	*CDCP1*	autophagy related 7	0.347	0.0286
204656_at	*COCH*	SH2 domain containing adaptor protein B	0.389	0.0287
202339_at	*WARS*	symplekin	0.351	0.0287
207595_s_at	*B3GALNT1*	bone morphogenetic protein 1	0.224	0.0287
201598_s_at	*MOXD1*	inositol polyphosphate phosphatase like 1	0.717	0.0288
211730_s_at	*RITA1*	RNA polymerase II subunit L	0.519	0.0289
203452_at	*ANKRD10*	beta-1,3-glucuronyltransferase 3	0.423	0.0289
39650_s_at	*RAB2A*	pecanex homolog 2 (Drosophila)	0.957	0.0290
215654_at	*CRY2*	branched chain amino acid transaminase 2	0.266	0.0290
217667_at	*POLD4*	SEC14 like 1 pseudogene 1	0.371	0.0290
52255_s_at	*FAM90A1*	collagen type V alpha 3 chain	0.585	0.0290
32209_at	*CCDC28B*	family with sequence similarity 89 member B	0.369	0.0291
218657_at	*DDX39A*	Rap guanine nucleotide exchange factor like 1	0.897	0.0292
209596_at	*NCAPG*	matrix remodeling associated 5	1.326	0.0292
217934_x_at	*RPS6KC1*	STIP1 homology and U-box containing protein 1	0.388	0.0293
208466_at	*MYCN*	RAB3D, member RAS oncogene family	0.193	0.0293
216554_s_at	*ALOX12B*	enolase 1	0.215	0.0293
210499_s_at	*ERC2-IT1*	polyglutamine binding protein 1	0.297	0.0294
219563_at	*CDKN2D*	spectrin repeat containing nuclear envelope family member 3///long intergenic non-protein coding RNA 341	0.787	0.0294
220214_at	*LOC101928625///MED21*	zinc finger protein 215	1.098	0.0295
209726_at	*SDC3*	carbonic anhydrase 11	0.743	0.0296
202066_at	*DEPDC1*	PTPRF interacting protein alpha 1	0.300	0.0297
219489_s_at	*HMGB2*	nucleoredoxin	0.578	0.0298
209936_at	*TOMM70*	RNA binding motif protein 5	0.471	0.0298
209824_s_at	*PLD3*	aryl hydrocarbon receptor nuclear translocator like	0.588	0.0298
209529_at	*ATG13*	phospholipid phosphatase 2	1.215	0.0299
220491_at	*KIF20B*	hepcidin antimicrobial peptide	0.325	0.0303
221163_s_at	*TRIM44*	MLX interacting protein like	0.614	0.0305
208534_s_at	*PLEC*	RAS p21 protein activator 4B///RAS p21 protein activator 4C, pseudogene///RAS p21 protein activator 4	0.519	0.0306
220002_at	*ANAPC15*	kinesin family member 26B	0.290	0.0308
220007_at	*AMBRA1*	methyltransferase like 8	0.292	0.0309
209622_at	*ZNF337*	serine/threonine kinase 16	0.275	0.0309
219136_s_at	*CBLN1*	lipase maturation factor 1	0.409	0.0309

61874_at	*IL2RA*	calcium channel flower domain containing 1	0.288	0.0310
209323_at	*FBLN2*	THAP domain containing 12	0.270	0.0310
219956_at	*PLAGL1*	polypeptide N-acetylgalactosaminyltransferase 6	0.232	0.0311
203193_at	*F8*	estrogen related receptor alpha	0.249	0.0313
220464_at	*CENPJ*	MCF.2 cell line derived transforming sequence like	0.264	0.0313
220923_s_at	*MRPL13*	paraneoplastic Ma antigen 3	0.184	0.0315
217589_at	*ARPC4-TTLL3///TTLL3///ARPC4*	RAB40A, member RAS oncogene family	0.208	0.0315
203179_at	*PPP2R5E*	galactose-1-phosphate uridylyltransferase	0.394	0.0315
200918_s_at	*MRPL17*	SRP receptor alpha subunit	0.386	0.0315
213713_s_at	*PCDH9*	galactosidase beta 1 like 2	0.609	0.0316
207192_at	*MOCOS*	deoxyribonuclease 1 like 2	0.274	0.0317
209528_s_at	*PINK1*	telomere maintenance 2	0.389	0.0318
219145_at	*CRYBB2P1///CRYBB2*	adhesion G protein-coupled receptor L1	0.347	0.0318
209492_x_at	*LTBR*	ATP synthase, H+ transporting, mitochondrial Fo complex subunit E	0.255	0.0318
200954_at	*TRMU///GTSE1*	ATPase H+ transporting V0 subunit c	0.373	0.0319
207266_x_at	*SPG20*	RNA binding motif single stranded interacting protein 1	0.380	0.0319
206325_at	*ZFR*	serpin family A member 6	0.193	0.0320
209236_at	*AMFR*	solute carrier family 23 member 2	0.525	0.0320
219549_s_at	*AFF1*	reticulon 3	0.272	0.0322
207386_at	*ENPP4*	cytochrome P450 family 7 subfamily B member 1	0.584	0.0322
202387_at	*FNBP1*	BCL2 associated athanogene 1	0.334	0.0322
217937_s_at	*LMAN2*	histone deacetylase 7	0.286	0.0323
218975_at	*HSPA6*	collagen type V alpha 3 chain	0.435	0.0324
208174_x_at	*ZDHHC24*	zinc finger CCCH-type, RNA binding motif and serine/arginine rich 2	0.299	0.0324
202867_s_at	*NPRL3*	DnaJ heat shock protein family (Hsp40) member B12	0.237	0.0325
202048_s_at	*THY1*	chromobox 6	0.366	0.0325
1487_at	*RPL27A*	estrogen related receptor alpha	0.388	0.0325
213804_at	*RFX2*	inositol polyphosphate-5-phosphatase B	0.405	0.0326
220282_at	*CDC5L*	RIC3 acetylcholine receptor chaperone	0.289	0.0326
204293_at	*DOLK*	N-sulfoglucosamine sulfohydrolase	0.297	0.0327
207335_x_at	*AASS*	ATP synthase, H+ transporting, mitochondrial Fo complex subunit E	0.282	0.0327
221616_s_at	*RAD51D*	TATA-box binding protein associated factor 9b	0.298	0.0328
215241_at	*ARIH2*	anoctamin 3	1.590	0.0329
219979_s_at	*RAB27B*	Hikeshi, heat shock protein nuclear import factor	0.350	0.0329
207173_x_at	*SLC39A8*	cadherin 11	1.098	0.0329

212908_at	*ACOX3*	DnaJ heat shock protein family (Hsp40) member C16	0.355	0.0330
207576_x_at	*PLCB1*	oxytocin/neurophysin I prepropeptide	0.632	0.0331
205067_at	*SYNJ2*	interleukin 1 beta	0.452	0.0332
205915_x_at	*GNAS*	glutamate ionotropic receptor NMDA type subunit 1	0.272	0.0333
207788_s_at	*MEIS3P1*	sorbin and SH3 domain containing 3	0.303	0.0333
200677_at	*POLR2K*	pituitary tumor-transforming 1 interacting protein	0.315	0.0334
221595_at	*KXD1*	RNA binding motif protein 48	0.217	0.0335
206600_s_at	*CCDC102B*	solute carrier family 16 member 5	0.231	0.0335
212034_s_at	*ZNHIT6*	exocyst complex component 7	0.237	0.0336
220356_at	*TRAPPC2L*	corin, serine peptidase	0.214	0.0336
203219_s_at	*TTR*	adenine phosphoribosyltransferase	0.330	0.0337
202586_at	*STAU2*	RNA polymerase II subunit L	0.373	0.0337
219135_s_at	*CUZD1*	lipase maturation factor 1	0.358	0.0337
211651_s_at	*CYP27A1*	laminin subunit beta 1	0.742	0.0337
221562_s_at	*RND3*	sirtuin 3	0.475	0.0337
210667_s_at	*CLIP2*	chromosome 21 open reading frame 33	0.291	0.0338
210809_s_at	*RPS28*	periostin	1.163	0.0338
200055_at	*SLCO2B1*	TATA-box binding protein associated factor 10	0.428	0.0339
215054_at	*TMC5*	ral guanine nucleotide dissociation stimulator like 3///erythropoietin receptor	0.303	0.0340
213153_at	*ACBD4*	SET domain containing 1B	0.259	0.0340
220204_s_at	*PDS5B*	bone morphogenetic protein 8a	0.312	0.0341
208432_s_at	*KLK10*	calcium voltage-gated channel subunit alpha1 E	0.251	0.0342
35436_at	*CFLAR*	golgin A2	0.250	0.0342
221406_s_at	*SNORA52///RPLP2*	MSH5-SAPCD1 readthrough (NMD candidate)///suppressor APC domain containing 1	0.458	0.0342
209685_s_at	*FGF2*	protein kinase C beta	1.205	0.0343
214719_at	*PGM3*	solute carrier family 46 member 3	0.633	0.0344
213558_at	*C1QTNF3-AMACR///AMACR*	piccolo presynaptic cytomatrix protein	0.410	0.0344
214841_at	*MMP2*	cornichon family AMPA receptor auxiliary protein 3	0.299	0.0344
218598_at	*GLT8D2*	RAD50 interactor 1	0.292	0.0345
221610_s_at	*MSANTD3-TMEFF1///TMEFF1*	signal transducing adaptor family member 2	0.320	0.0346
203118_at	*KIR2DS1*	proprotein convertase subtilisin/kexin type 7	0.348	0.0346
221027_s_at	*SAMD4A*	phospholipase A2 group XIIA	0.380	0.0347
219884_at	*SYNRG*	LIM homeobox 6	0.558	0.0347
204653_at	*MTMR6*	transcription factor AP-2 alpha	0.783	0.0348
210761_s_at	*CROCC*	growth factor receptor bound protein 7	0.664	0.0348
201047_x_at	*DAZAP2*	RAB6C-like///RAB6A, member RAS oncogene family	0.254	0.0350

205914_s_at	*PRPF19*	glutamate ionotropic receptor NMDA type subunit 1	0.345	0.0352
208906_at	*GATA3*	HNRNPUL2-BSCL2 readthrough (NMD candidate)///BSCL2, seipin lipid droplet biogenesis associated	0.543	0.0352
215135_at	*SOS2*	aspartyl aminopeptidase	0.193	0.0352
203188_at	*RPS25*	beta-1,4-glucuronyltransferase 1	0.480	0.0353
44617_at	*FUT6*	2-oxoglutarate and iron dependent oxygenase domain containing 2	0.199	0.0353
222006_at	*HSPA4*	leucine zipper and EF-hand containing transmembrane protein 1	0.269	0.0354
210740_s_at	*WHSC1*	inositol-tetrakisphosphate 1-kinase	0.300	0.0354
212968_at	*USP32P2*	RFNG O-fucosylpeptide 3-beta-N-acetylglucosaminyltransferase	0.271	0.0355
219188_s_at	*SHCBP1*	MACRO domain containing 1	0.465	0.0355
203916_at	*RRN3P1*	N-deacetylase and N-sulfotransferase 2	0.231	0.0356
212298_at	*FAM134B*	neuropilin 1	0.774	0.0356
222184_at	*MRPS34*	long intergenic non-protein coding RNA 965	0.682	0.0357
221856_s_at	*CRYZ*	family with sequence similarity 63 member A	0.241	0.0357
212706_at	*ARHGAP4*	uncharacterized LOC102724229///RAS p21 protein activator 4B///RAS p21 protein activator 4	0.461	0.0358
218792_s_at	*ARF6*	B-box and SPRY domain containing	0.933	0.0358
204577_s_at	*GRIN2C*	clusterin associated protein 1	0.438	0.0359
217846_at	*SCG5*	microRNA 6890///glutaminyl-tRNA synthetase	0.413	0.0360
222170_at	*CES3*	ZNF213 antisense RNA 1 (head to head)	0.293	0.0360
212075_s_at	*TMEM9B*	casein kinase 2 alpha 1	0.277	0.0360
214149_s_at	*TBC1D8B*	ATPase H+ transporting V0 subunit e1	0.264	0.0361
208196_x_at	*NEMF*	nuclear factor of activated T-cells 1	0.212	0.0361
209635_at	*MICAL1*	adaptor related protein complex 1 sigma 1 subunit	0.281	0.0362
202492_at	*ILF2*	autophagy related 9A	0.343	0.0362
210752_s_at	*PTK6*	MLX, MAX dimerization protein	0.380	0.0363
202790_at	*POU2AF1*	claudin 7	1.095	0.0364
219021_at	*CDKN1B*	ring finger protein 121	0.293	0.0364
207260_at	*ANKRD26*	FEV, ETS transcription factor	1.147	0.0365
205711_x_at	*IL1RL1*	ATP synthase, H+ transporting, mitochondrial F1 complex, gamma polypeptide 1	0.255	0.0365
222215_at	*SNX4*	solute carrier family 38 member 7	0.209	0.0365
207436_x_at	*HNRNPA3///HNRNPA3P1*	sorbin and SH3 domain containing 1	0.215	0.0366
206607_at	*FERMT2*	Cbl proto-oncogene	0.184	0.0366
222125_s_at	*C16orf58*	prolyl 4-hydroxylase, transmembrane	0.560	0.0367

Under-expressed genes in Metastases vs. Primary MCC tumors

221823_at	*C5orf30*	chromosome 5 open reading frame 30	-1.250	2.2E-05
201664_at	*SMC4*	structural maintenance of chromosomes 4	-0.801	3.3E-05
208119_s_at	*ZNF93*	zinc finger protein 93	-0.495	3.7E-05

205642_at	*BTBD18*	centriolin	-0.531	4.4E-05
207828_s_at	*CSH2///CSH1*	centromere protein F	-1.086	8.2E-05
202413_s_at	*SMC6*	ubiquitin specific peptidase 1	-0.394	0.0001
218781_at	*KIF14*	structural maintenance of chromosomes 6	-0.515	0.0002
206364_at	*RBM8A*	kinesin family member 14	-1.217	0.0002
201663_s_at	*AGTR1*	structural maintenance of chromosomes 4	-0.911	0.0002
217857_s_at	*TGIF1*	RNA binding motif protein 8A	-0.540	0.0002
203313_s_at	*GRAMD1B*	TGFB induced factor homeobox 1	-1.434	0.0002
210180_s_at	*SMG9*	transformer 2 beta homolog (Drosophila)	-0.473	0.0002
221335_x_at	*DUS1L*	SMG9, nonsense mediated mRNA decay factor	-0.456	0.0003
202293_at	*GH2*	stromal antigen 1	-0.691	0.0003
207071_s_at	*PDK2*	aconitase 1	-0.646	0.0004
200593_s_at	*PTGS1*	heterogeneous nuclear ribonucleoprotein U	-0.371	0.0004
209608_s_at	*GH2///GH1///CSHL1///CSH1*	acetyl-CoA acetyltransferase 2	-0.572	0.0005
208777_s_at	*CCNJL*	proteasome 26S subunit, non-ATPase 11	-0.328	0.0005
215509_s_at	*ELK1*	BUB1 mitotic checkpoint serine/threonine kinase	-0.549	0.0006
219227_at	*H2AFV*	cyclin J like	-0.656	0.0006
204162_at	*TMPO*	NDC80, kinetochore complex component	-1.023	0.0006
210376_x_at	*ROBO3*	ELK1, ETS transcription factor	-0.359	0.0006
202487_s_at	*NUP205*	H2A histone family member V	-0.644	0.0006
209754_s_at	*PEX16*	thymopoietin	-0.752	0.0007
209172_s_at	*ZNF302*	centromere protein F	-0.986	0.0007
213329_at	*ASPM*	SLIT-ROBO Rho GTPase activating protein 2C///SLIT-ROBO Rho GTPase activating protein 2B///SLIT-ROBO Rho GTPase activating protein 2	-0.686	0.0007
209457_at	*SIL1*	dual specificity phosphatase 5	-1.075	0.0008
212247_at	*PTPN3*	nucleoporin 205	-0.366	0.0008
218490_s_at	*OR7E12P*	zinc finger protein 302	-0.480	0.0008
219918_s_at	*KCNMB3*	abnormal spindle microtubule assembly	-0.953	0.0008
211080_s_at	*CERS4*	NIMA related kinase 2	-1.162	0.0009
209505_at	*HMGB3*	nuclear receptor subfamily 2 group F member 1	-1.626	0.0009
202647_s_at	*NUP62*	neuroblastoma RAS viral oncogene homolog	-0.966	0.0009
203997_at	*LOC101930388///LOC101928143///NUMB*	protein tyrosine phosphatase, non-receptor type 3	-0.960	0.0009
221125_s_at	*LPCAT4*	potassium calcium-activated channel subfamily M regulatory beta subunit 3	-0.475	0.0010
204641_at	*CTSF*	NIMA related kinase 2	-0.967	0.0010
203744_at	*GH1///CSHL1///CSH2///CSH1*	high mobility group box 3	-0.585	0.0011
202153_s_at	*TTC37*	nucleoporin 62	-0.519	0.0011

209073_s_at	*GH1///CSHL1///CSH1*	uncharacterized LOC101930388///uncharacterized LOC101928143///NUMB, endocytic adaptor protein	-0.363	0.0011
201514_s_at	*DIO2*	G3BP stress granule assembly factor 1	-0.416	0.0012
203574_at	*SLC35F6*	nuclear factor, interleukin 3 regulated	-1.101	0.0012
203048_s_at	*SETD3*	tetratricopeptide repeat domain 37	-0.433	0.0014
202946_s_at	*TRAK2*	BTB domain containing 3	-0.711	0.0015
202503_s_at	*COX4I1*	KIAA0101	-0.821	0.0015
205345_at	*CEP135*	BRCA1 associated RING domain 1	-0.672	0.0015
209753_s_at	*HIP1*	thymopoietin	-0.662	0.0016
219306_at	*CYBA*	kinesin family member 15	-1.010	0.0016
202651_at	*RABEPK*	lysophosphatidylglycerol acyltransferase 1	-1.154	0.0016
219765_at	*FAM173A*	zinc finger protein 329	-0.537	0.0016
208200_at	*VPS11*	interleukin 1 alpha	-0.621	0.0016
204531_s_at	*GTF2H1*	BRCA1, DNA repair associated	-0.406	0.0017
209927_s_at	*CD24*	chromatin target of PRMT1	-0.396	0.0017
204023_at	*PIK3CA*	replication factor C subunit 4	-0.413	0.0017
202124_s_at	*RHOBTB3*	trafficking kinesin protein 2	-0.467	0.0017
206003_at	*PMAIP1*	centrosomal protein 135	-0.836	0.0017
204835_at	*FUT2*	DNA polymerase alpha 1, catalytic subunit	-0.330	0.0018
209642_at	*HOXC10*	BUB1 mitotic checkpoint serine/threonine kinase	-1.065	0.0019
201635_s_at	*OR2H2*	FMR1 autosomal homolog 1	-0.342	0.0020
209772_s_at	*PHLPP2*	CD24 molecule	-0.858	0.0020
204369_at	*ATAD5*	phosphatidylinositol-4,5-bisphosphate 3-kinase catalytic subunit alpha	-0.528	0.0020
216048_s_at	*ZMYM1*	Rho related BTB domain containing 3	-0.816	0.0020
204285_s_at	*CENPI*	phorbol-12-myristate-13-acetate-induced protein 1	-1.085	0.0021
203358_s_at	*HNRNPC*	enhancer of zeste 2 polycomb repressive complex 2 subunit	-0.475	0.0021
201636_at	*RAB14*	FMR1 autosomal homolog 1	-0.353	0.0021
200664_s_at	*IGK///IGKC*	DnaJ heat shock protein family (Hsp40) member B1	-0.925	0.0021
214113_s_at	*ITCH*	RNA binding motif protein 8A	-0.435	0.0021
212371_at	*DNAJB12*	desumoylating isopeptidase 2	-0.615	0.0021
203653_s_at	*RAB15*	coilin	-0.369	0.0022
220223_at	*CEACAM4*	ATPase family, AAA domain containing 5	-0.369	0.0022
220206_at	*AHCTF1*	zinc finger MYM-type containing 1	-0.585	0.0022
214804_at	*EPHX2*	centromere protein I	-0.764	0.0023
212626_x_at	*MELK*	heterogeneous nuclear ribonucleoprotein C (C1/C2)	-0.232	0.0023
201292_at	*AMPD2*	topoisomerase (DNA) II alpha	-0.730	0.0023
221671_x_at	*CRLF3*	immunoglobulin kappa locus///immunoglobulin kappa constant	-3.104	0.0024
209743_s_at	*CDC25C*	itchy E3 ubiquitin protein ligase	-0.380	0.0024
205111_s_at	*PKP3*	phospholipase C epsilon 1	-1.021	0.0024

221651_x_at	*ITGB3BP*	immunoglobulin kappa locus///immunoglobulin kappa constant	-3.021	0.0024
204603_at	*CHD3*	exonuclease 1	-0.616	0.0024
204286_s_at	*MCF2L2*	phorbol-12-myristate-13-acetate-induced protein 1	-1.051	0.0025
214766_s_at	*CD44*	AT-hook containing transcription factor 1	-0.473	0.0025
204984_at	*ZNF512B*	glypican 4	-1.226	0.0026
207590_s_at	*TYMS*	centromere protein I	-0.475	0.0027
214697_s_at	*LMNB1*	polypyrimidine tract binding protein 3	-0.665	0.0028
202412_s_at	*CHAD*	ubiquitin specific peptidase 1	-0.442	0.0028
204825_at	*MIR8071-2///MIR8071-1///IGHV4-31///IGHM///IGHG2///IGHG1*	maternal embryonic leucine zipper kinase	-0.800	0.0028
218304_s_at	*PDLIM5*	oxysterol binding protein like 11	-0.503	0.0029
205474_at	*PRG2*	cytokine receptor like factor 3	-0.585	0.0029
205167_s_at	*MIR6516///SCARNA16///SNHG20*	cell division cycle 25C	-0.765	0.0030
218705_s_at	*TTK*	sorting nexin 24	-0.770	0.0030
212317_at	*STON1*	transportin 3	-0.277	0.0030
206412_at	*SLC12A8*	FER tyrosine kinase	-0.321	0.0030
205176_s_at	*PAEP*	integrin subunit beta 3 binding protein	-0.396	0.0030
220199_s_at	*COL7A1*	axin interactor, dorsalization associated	-0.585	0.0031
217239_x_at	*CEP152*	uncharacterized LOC101930405	-0.252	0.0032
210287_s_at	*SPDEF*	fms related tyrosine kinase 1	-0.537	0.0032
214812_s_at	*ZSCAN16*	MOB kinase activator 1A	-0.541	0.0032
222158_s_at	*FBXW11*	desumoylating isopeptidase 2	-0.643	0.0032
214677_x_at	*ZNF146*	immunoglobulin lambda constant 1	-2.948	0.0033
206414_s_at	*PPFIBP1*	ArfGAP with SH3 domain, ankyrin repeat and PH domain 2	-0.430	0.0033
206023_at	*EI24*	neuromedin U	-1.767	0.0033
216952_s_at	*TNK1*	lamin B2	-0.595	0.0033
202589_at	*CD58*	thymidylate synthetase	-0.516	0.0033
218782_s_at	*ALMS1*	ATPase family, AAA domain containing 2	-0.941	0.0034
220840_s_at	*CASP10*	chromosome 1 open reading frame 112	-0.717	0.0034
203276_at	*OSGEP*	lamin B1	-0.570	0.0034
203654_s_at	*NXPH3*	coilin	-0.410	0.0034
211430_s_at	*SEC14L1*	microRNA 8071-2///microRNA 8071-1///immunoglobulin heavy variable 4-31///immunoglobulin heavy constant mu///immunoglobulin heavy constant gamma 2 (G2m marker)///immunoglobulin heavy constant gamma 1 (G1m marker)	-3.112	0.0034
204321_at	*TCEB2*	neogenin 1	-1.050	0.0035
213684_s_at	*CDK2*	PDZ and LIM domain 5	-0.546	0.0035
209138_x_at	*UBE2C*	immunoglobulin lambda constant 1	-2.675	0.0035

204822_at	*SPAG5*	TTK protein kinase	-0.963	0.0035
207746_at	*CYAT1///IGLV1-44///IGLC1*	DNA polymerase theta	-0.902	0.0036
213413_at	*WDYHV1*	stonin 1	-0.848	0.0036
203714_s_at	*PLK2*	tubulin folding cofactor E	-0.404	0.0036
210188_at	*SMYD2*	GA binding protein transcription factor alpha subunit	-0.347	0.0037
212287_at	*ENOX1*	SUZ12 polycomb repressive complex 2 subunit	-0.338	0.0037
217010_s_at	*SEMA6A*	cell division cycle 25C	-0.631	0.0038
209422_at	*PLK4*	PHD finger protein 20	-0.399	0.0038
215758_x_at	*HACD2*	zinc finger protein 93	-0.402	0.0038
201179_s_at	*ORAI3*	G protein subunit alpha i3	-0.439	0.0039
215170_s_at	*SLC11A2*	centrosomal protein 152	-0.887	0.0039
219676_at	*KIF18B*	zinc finger and SCAN domain containing 16	-0.491	0.0040
213906_at	*PPP6R3*	MYB proto-oncogene like 1	-1.221	0.0040
200050_at	*RNF39*	zinc finger protein 146	-0.261	0.0040
204444_at	*CHAF1A*	kinesin family member 11	-0.672	0.0041
205953_at	*MIR7110///PDIA5*	leucine rich repeats and immunoglobulin like domains 2	-0.348	0.0041
222061_at	*ARL4C*	CD58 molecule	-0.308	0.0042
208836_at	*FUT4*	ATPase Na+/K+ transporting subunit beta 3	-0.381	0.0043
214220_s_at	*TRAF3IP2*	ALMS1, centrosome and basal body associated protein	-0.358	0.0043
209450_at	*SCAMP1*	O-sialoglycoprotein endopeptidase	-0.432	0.0043
204252_at	*FBXO24*	cyclin dependent kinase 2	-0.445	0.0044
202954_at	*COMMD9*	ubiquitin conjugating enzyme E2 C	-0.868	0.0044
203211_s_at	*CDKN2C*	myotubularin related protein 2	-1.633	0.0044
203145_at	*SGCB*	sperm associated antigen 5	-0.724	0.0044
215121_x_at	*COPA*	immunoglobulin lambda light chain-like///immunoglobulin lambda variable 1-44///immunoglobulin lambda constant 1	-2.521	0.0045
219060_at	*STIL*	WDYHV motif containing 1	-0.572	0.0045
201939_at	*RBM12B*	polo like kinase 2	-1.072	0.0046
204709_s_at	*WDR62*	kinesin family member 23	-0.977	0.0046
201374_x_at	*RAD54L*	protein phosphatase 2 catalytic subunit beta	-0.212	0.0046
212922_s_at	*CPB1*	SET and MYND domain containing 2	-0.440	0.0046
219501_at	*PURA*	ecto-NOX disulfide-thiol exchanger 1	-0.775	0.0047
215028_at	*ABCD1*	semaphorin 6A	-0.778	0.0047
203764_at	*MEX3D*	DLG associated protein 5	-0.934	0.0047
204887_s_at	*MPP5*	polo like kinase 4	-0.708	0.0048
212640_at	*NR3C1*	3-hydroxyacyl-CoA dehydratase 2	-0.249	0.0048
214836_x_at	*TPX2*	immunoglobulin kappa locus///immunoglobulin kappa constant	-2.467	0.0048
204011_at	*RGCC*	sprouty RTK signaling antagonist 2	-0.953	0.0048
222039_at	*ICK*	kinesin family member 18B	-0.630	0.0048
215379_x_at	*COA3*	immunoglobulin lambda variable 1-44	-2.611	0.0049
209773_s_at	*CHGA*	ribonucleotide reductase regulatory subunit M2	-0.881	0.0049
207164_s_at	*CSH1*	zinc finger and BTB domain containing 18	-0.477	0.0049

203976_s_at	*CDCA3*	chromatin assembly factor 1 subunit A	-0.494	0.0050
200751_s_at	*KIF2C*	heterogeneous nuclear ribonucleoprotein C (C1/C2)	-0.296	0.0050
201896_s_at	*DESI1*	proline and serine rich coiled-coil 1	-0.779	0.0050
202107_s_at	*GRK4*	minichromosome maintenance complex component 2	-0.362	0.0051
202208_s_at	*BTN2A1*	ADP ribosylation factor like GTPase 4C	-0.668	0.0051
209893_s_at	*ARFGAP2*	fucosyltransferase 4	-0.596	0.0051
218542_at	*NAALADL1*	centrosomal protein 55	-0.850	0.0052
217043_s_at	*MAP2*	mitofusin 1	-0.399	0.0052
206973_at	*DLC1*	PTPRF interacting protein alpha 2	-0.822	0.0052
201054_at	*ITFG2*	heterogeneous nuclear ribonucleoprotein A0	-0.357	0.0053
211792_s_at	*DLAT*	cyclin dependent kinase inhibitor 2C	-0.760	0.0054
214737_x_at	*WNT6*	heterogeneous nuclear ribonucleoprotein C (C1/C2)	-0.214	0.0054
205120_s_at	*RELN*	sarcoglycan beta	-0.580	0.0054
208775_at	*ILF3*	exportin 1	-0.224	0.0054
214336_s_at	*FANCI*	coatomer protein complex subunit alpha	-0.381	0.0054
201748_s_at	*PRKD3*	scaffold attachment factor B	-0.252	0.0054
205339_at	*MIR4745///PTBP1*	SCL/TAL1 interrupting locus	-0.715	0.0054
201299_s_at	*NEUROD2*	MOB kinase activator 1A	-0.588	0.0054
51228_at	*STUM*	RNA binding motif protein 12B	-0.514	0.0055
215218_s_at	*KERA*	WD repeat domain 62	-0.394	0.0055
204558_at	*PAK2*	RAD54-like (S. cerevisiae)	-0.583	0.0055
91816_f_at	*STC1*	mex-3 RNA binding family member D	-0.538	0.0057
219321_at	*OPTN*	membrane palmitoylated protein 5	-0.497	0.0057
201866_s_at	*RLF*	nuclear receptor subfamily 3 group C member 1	-0.601	0.0057
210052_s_at	*NUAK1*	TPX2, microtubule nucleation factor	-0.762	0.0057
206472_s_at	*HOXC8*	transducin like enhancer of split 3	-0.434	0.0057
219650_at	*ETFB*	ERCC excision repair 6 like, spindle assembly checkpoint helicase	-0.558	0.0058
218723_s_at	*HOXC4*	regulator of cell cycle	-0.869	0.0058
204569_at	*LPCAT3*	intestinal cell (MAK-like) kinase	-0.630	0.0058
222036_s_at	*HLA-F-AS1*	minichromosome maintenance complex component 4	-0.487	0.0059
205656_at	*HYAL1*	protocadherin 17	-1.054	0.0059
212816_s_at	*TRAPPC6A*	cystathionine-beta-synthase	-1.887	0.0060
221436_s_at	*PITRM1*	cell division cycle associated 3	-0.770	0.0060
212445_s_at	*TCTA*	neural precursor cell expressed, developmentally down-regulated 4-like, E3 ubiquitin protein ligase	-0.896	0.0061
209408_at	*DLG5*	kinesin family member 2C	-0.775	0.0061
201298_s_at	*GDF11*	MOB kinase activator 1A	-0.573	0.0061
203944_x_at	*FOXJ1*	butyrophilin subfamily 2 member A1	-0.402	0.0062
201585_s_at	*F2RL1*	splicing factor proline and glutamine rich	-0.311	0.0063
214162_at	*AURKA*	additional sex combs like 3, transcriptional regulator	-0.861	0.0063

218319_at	*PTGIS*	pellino E3 ubiquitin protein ligase 1	-0.893	0.0063
219510_at	*IGFBP3*	DNA polymerase theta	-0.487	0.0063
210015_s_at	*KCNJ2*	microtubule associated protein 2	-0.899	0.0064
210762_s_at	*NRGN*	DLC1 Rho GTPase activating protein	-0.944	0.0064
202975_s_at	*QKI*	Rho related BTB domain containing 3	-0.576	0.0064
212132_at	*PSMB2*	LSM14A, mRNA processing body assembly factor	-0.298	0.0065
217805_at	*PLPPR2*	interleukin enhancer binding factor 3	-0.330	0.0066
213008_at	*UROS*	Fanconi anemia complementation group I	-0.383	0.0066
211084_x_at	*ANKRD6*	protein kinase D3	-0.462	0.0066
202189_x_at	*ATP2B3*	microRNA 4745///polypyrimidine tract binding protein 1	-0.229	0.0066
212774_at	*RAB28*	zinc finger and BTB domain containing 18	-0.644	0.0067
208878_s_at	*RAD21*	p21 (RAC1) activated kinase 2	-0.340	0.0067
218247_s_at	*ASF1B*	mex-3 RNA binding family member C	-0.514	0.0067
211375_s_at	*SETSIP///SETP4///SET*	interleukin enhancer binding factor 3	-0.310	0.0068
204596_s_at	*OR7E47P*	stanniocalcin 1	-0.705	0.0068
204886_at	*TSPAN4*	polo like kinase 4	-0.664	0.0069
204243_at	*KLK11*	rearranged L-myc fusion	-0.319	0.0069
206403_at	*JCHAIN*	zinc finger protein 536	-1.732	0.0069
215176_x_at	*S100A5*	immunoglobulin kappa locus///immunoglobulin kappa constant	-3.201	0.0071
210292_s_at	*TM7SF2*	protocadherin 11 Y-linked///protocadherin 11 X-linked	-1.843	0.0073
218039_at	*NCAPH*	nucleolar and spindle associated protein 1	-0.526	0.0073
201223_s_at	*CEP170P1///CEP170*	RAD23 homolog B, nucleotide excision repair protein	-0.337	0.0074
202627_s_at	*PHC3*	serpin family E member 1	-0.841	0.0074
213506_at	*PDLIM2*	F2R like trypsin receptor 1	-0.607	0.0075
204092_s_at	*C7*	aurora kinase A	-0.679	0.0076
212143_s_at	*ECT2*	insulin like growth factor binding protein 3	-1.162	0.0076
201890_at	*HIRA*	ribonucleotide reductase regulatory subunit M2	-0.578	0.0076
206765_at	*SLIT2*	potassium voltage-gated channel subfamily J member 2	-0.941	0.0076
208650_s_at	*OGFOD2*	CD24 molecule	-0.879	0.0077
221030_s_at	*CCDC51*	Rho GTPase activating protein 24	-0.193	0.0077
212263_at	*TXN*	QKI, KH domain containing RNA binding	-0.913	0.0078
211519_s_at	*LAMB1*	kinesin family member 2C	-0.744	0.0079
221505_at	*ITPR1*	acidic nuclear phosphoprotein 32 family member E	-0.448	0.0079
214426_x_at	*MIR1178///CIT*	chromatin assembly factor 1 subunit A	-0.442	0.0079
204671_s_at	*MUM1*	ankyrin repeat domain 6	-1.853	0.0081
218355_at	*KATNA1*	kinesin family member 4A	-0.719	0.0082
200958_s_at	*LMF1*	syndecan binding protein	-0.930	0.0083
207495_at	*FXYD3*	RAB28, member RAS oncogene family	-0.346	0.0083
206567_s_at	*MSRB1*	PHD finger protein 20	-0.415	0.0083
206500_s_at	*SLC25A12*	MIS18 binding protein 1	-0.344	0.0084

200989_at	*ING1*	hypoxia inducible factor 1 alpha subunit	-0.313	0.0084
200607_s_at	*NDRG3*	RAD21 cohesin complex component	-0.562	0.0084
218115_at	*VAMP2*	anti-silencing function 1B histone chaperone	-0.772	0.0084
218336_at	*LRPPRC*	prefoldin subunit 2	-0.329	0.0085
209201_x_at	*NCOR2*	C-X-C motif chemokine receptor 4	-1.078	0.0085
215780_s_at	*VAC14*	SET-like protein///SET pseudogene 4///SET nuclear proto-oncogene	-0.263	0.0086
203098_at	*MIR6773///ESRP2*	chromodomain Y-like	-0.527	0.0086
212592_at	*CCDC86*	joining chain of multimeric IgA and IgM	-2.718	0.0087
205437_at	*USP32///USP6*	zinc finger protein 211	-0.508	0.0087
211270_x_at	*NPY1R*	polypyrimidine tract binding protein 1	-0.213	0.0089
211671_s_at	*GPRC5B*	nuclear receptor subfamily 3 group C member 1	-0.916	0.0089
211527_x_at	*CDKN3*	vascular endothelial growth factor A	-1.318	0.0089
212919_at	*COPB1*	decapping mRNA 2	-0.431	0.0089
212949_at	*TAOK2*	non-SMC condensin I complex subunit H	-0.690	0.0090
204207_s_at	*PTCH1*	RNA guanylyltransferase and 5'-phosphatase	-0.408	0.0090
201297_s_at	*SRSF3*	MOB kinase activator 1A	-0.573	0.0090
210537_s_at	*ABCG5*	transcriptional adaptor 2A	-0.294	0.0090
204672_s_at	*MIR1204///PVT1*	ankyrin repeat domain 6	-1.667	0.0091
211087_x_at	*SMUG1*	mitogen-activated protein kinase 14	-0.254	0.0092
212746_s_at	*FMO3*	centrosomal protein 170 pseudogene 1///centrosomal protein 170	-0.481	0.0092
220328_at	*UXT*	polyhomeotic homolog 3	-0.207	0.0092
217022_s_at	*KIAA0355*	immunoglobulin heavy constant alpha 2 (A2m marker)///immunoglobulin heavy constant alpha 1///immunoglobulin heavy locus	-2.823	0.0092
203881_s_at	*SIAH2*	dystrophin	-1.144	0.0092
206075_s_at	*TMEM256-PLSCR3///PLSCR3*	casein kinase 2 alpha 1	-0.288	0.0092
218539_at	*TMSB15B///TMSB15A*	F-box protein 34	-0.338	0.0092
202992_at	*GORASP1*	complement component 7	-1.759	0.0093
219787_s_at	*POU4F1*	epithelial cell transforming 2	-0.775	0.0095
208931_s_at	*MANBA*	interleukin enhancer binding factor 3	-0.476	0.0096
201741_x_at	*PDIA2*	serine and arginine rich splicing factor 1	-0.384	0.0096
214159_at	*STRA6*	phospholipase C epsilon 1	-0.236	0.0096
209897_s_at	*CHKA*	slit guidance ligand 2	-0.680	0.0096
219000_s_at	*VILL*	DNA replication and sister chromatid cohesion 1	-0.586	0.0097
203617_x_at	*KIF20A*	ELK1, ETS transcription factor	-0.268	0.0097
214550_s_at	*BBOF1*	transportin 3	-0.300	0.0097
208708_x_at	*LOC101929368///FKBP1A*	eukaryotic translation initiation factor 5	-0.437	0.0098
211200_s_at	*CAPN1*	EF-hand calcium binding domain 2	-0.860	0.0098

203418_at	*CXCL1*	cyclin A2	-0.938	0.0099
208864_s_at	*HSPA1L///HSPA1B///HSPA1A*	thioredoxin	-0.379	0.0099
205112_at	*NCBP2*	phospholipase C epsilon 1	-0.663	0.0099
823_at	*MTSS1*	C-X3-C motif chemokine ligand 1	-0.312	0.0099
216942_s_at	*LGALS3BP*	CD58 molecule	-0.808	0.0099
218875_s_at	*TRAF5*	F-box protein 5	-0.615	0.0100
212320_at	*CDKN2B*	tubulin beta class I	-0.326	0.0100
221685_s_at	*CLIC4*	spindle apparatus coiled-coil protein 1	-0.401	0.0100
212801_at	*GPX2*	microRNA 1178///citron rho-interacting serine/threonine kinase	-0.564	0.0100
218614_at	*PRKCI*	KIAA1551	-0.707	0.0101
210543_s_at	*LOC100506558///MATN2*	protein kinase, DNA-activated, catalytic polypeptide	-0.528	0.0101
203100_s_at	*ZNF318*	chromodomain Y-like	-0.635	0.0102
221290_s_at	*ZNF234*	melanoma associated antigen (mutated) 1	-0.440	0.0102
205526_s_at	*NDUFA10*	katanin catalytic subunit A1	-0.312	0.0103
201291_s_at	*OBSL1*	topoisomerase (DNA) II alpha	-0.584	0.0103
209808_x_at	*ZWILCH*	inhibitor of growth family member 1	-0.268	0.0104
221082_s_at	*BPHL*	NDRG family member 3	-0.331	0.0104
212016_s_at	*ITGB4*	microRNA 4745///polypyrimidine tract binding protein 1	-0.393	0.0109
214543_x_at	*CRYBB1*	QKI, KH domain containing RNA binding	-0.708	0.0109
221522_at	*BAD*	ankyrin repeat domain 27	-0.274	0.0109
205440_s_at	*ZNF250*	neuropeptide Y receptor Y1	-0.641	0.0110
210350_x_at	*MED1*	inhibitor of growth family member 1	-0.244	0.0111
221520_s_at	*ARHGEF28*	cell division cycle associated 8	-0.703	0.0111
204962_s_at	*GLT8D1*	solute carrier family 35 member F6///centromere protein A	-0.747	0.0112
209714_s_at	*ZNF137P*	cyclin dependent kinase inhibitor 3	-0.794	0.0112
202899_s_at	*MDM2*	serine and arginine rich splicing factor 3	-0.338	0.0113
222087_at	*PRKCD*	microRNA 1204///Pvt1 oncogene (non-protein coding)	-0.422	0.0113
218495_at	*ABHD3*	ubiquitously expressed prefoldin like chaperone	-0.206	0.0114
203288_at	*GGA2*	KIAA0355	-0.358	0.0114
209339_at	*USP19*	siah E3 ubiquitin protein ligase 2	-0.280	0.0114
209084_s_at	*MAPK6*	RAB28, member RAS oncogene family	-0.399	0.0115
205347_s_at	*C11orf98///LBHD1*	thymosin beta 15B///thymosin beta 15a	-0.804	0.0115
208290_s_at	*DST*	eukaryotic translation initiation factor 5	-0.512	0.0115
204937_s_at	*LIAS*	zinc finger protein 274	-0.472	0.0115
211745_x_at	*IL11RA*	hemoglobin subunit alpha 2///hemoglobin subunit alpha 1	-1.293	0.0115
214295_at	*GGT2///GGTLC1///GGTLC2///GGT1*	uncharacterized LOC57235	-0.770	0.0115
218755_at	*DDX25*	kinesin family member 20A	-0.704	0.0117

220890_s_at	*PNPLA2*	apolipoprotein L domain containing 1///DEAD-box helicase 47	-0.357	0.0117
202157_s_at	*FZD5*	CUGBP, Elav-like family member 2	-0.598	0.0117
200959_at	*RBM48*	FUS RNA binding protein	-0.285	0.0117
210187_at	*SNORA21///RPL23*	uncharacterized LOC101929368///FK506 binding protein 1A	-0.553	0.0118
205967_at	*CARD8*	histone cluster 2, H4b///histone cluster 4, H4///histone cluster 2, H4a///histone cluster 1, H4l///histone cluster 1, H4e///histone cluster 1, H4b///histone cluster 1, H4h///histone cluster 1, H4c///histone cluster 1, H4j///histone cluster 1, H4k///histone cluster 1, H4f///histone cluster 1, H4d///histone cluster 1, H4a///histone cluster 1, H4i	-0.298	0.0118
219978_s_at	*GABRB3*	nucleolar and spindle associated protein 1	-0.556	0.0119
201865_x_at	*PAPD7*	nuclear receptor subfamily 3 group C member 1	-0.829	0.0119
212921_at	*CEP70*	SET and MYND domain containing 2	-0.543	0.0119
200800_s_at	*CXCL11*	heat shock protein family A (Hsp70) member 1 like///heat shock protein family A (Hsp70) member 1B///heat shock protein family A (Hsp70) member 1A	-1.087	0.0120
201521_s_at	*SEBOX///VTN*	nuclear cap binding protein subunit 2	-0.311	0.0120
203037_s_at	*TUBGCP2*	metastasis suppressor 1	-1.146	0.0121
204352_at	*DPY19L4*	TNF receptor associated factor 5	-1.064	0.0121
211919_s_at	*LOC101930112///SPG7*	C-X-C motif chemokine receptor 4	-1.004	0.0121
212206_s_at	*GTSE1*	H2A histone family member V	-0.476	0.0122
207530_s_at	*RNF128*	cyclin dependent kinase inhibitor 2B	-0.420	0.0122
220046_s_at	*N4BP1*	cyclin L1	-0.271	0.0122
208103_s_at	*PRPSAP1*	acidic nuclear phosphoprotein 32 family member E	-0.666	0.0123
221881_s_at	*CEBPA-AS1*	chloride intracellular channel 4	-0.396	0.0124
216468_s_at	*MAPRE1*	zinc finger protein 682	-0.589	0.0125
209677_at	*ACYP1*	protein kinase C iota	-0.321	0.0126
202350_s_at	*PSME4*	uncharacterized LOC100506558///matrilin 2	-0.701	0.0127
203520_s_at	*LINC00963*	zinc finger protein 318	-0.309	0.0127
217663_at	*REM1*	zinc finger protein 234	-0.238	0.0128
206099_at	*YWHAQ*	protein kinase C eta	-0.454	0.0128
210029_at	*MAP3K7*	indoleamine 2,3-dioxygenase 1	-0.749	0.0129
201897_s_at	*HIPK1*	CDC28 protein kinase regulatory subunit 1B	-0.541	0.0129
211762_s_at	*EDNRB*	karyopherin subunit alpha 2	-0.503	0.0129
218349_s_at	*ECM1*	zwilch kinetochore protein	-0.492	0.0130
208705_s_at	*PHB2*	eukaryotic translation initiation factor 5	-0.320	0.0131
212467_at	*HNRNPA2B1*	DnaJ heat shock protein family (Hsp40) member C13	-0.348	0.0132
212742_at	*SET*	ring finger protein 115	-0.257	0.0133
220135_s_at	*PHTF1*	solute carrier family 7 member 9	-0.567	0.0134

207098_s_at	*SUZ12P1///SUZ12*	mitofusin 1	-0.418	0.0136
211256_x_at	*YBX1*	butyrophilin subfamily 2 member A1	-0.319	0.0136
209091_s_at	*TMED3*	SH3 domain containing GRB2 like endophilin B1	-0.544	0.0136
213858_at	*LOC100289518*	zinc finger protein 250	-0.370	0.0138
203497_at	*MYD88*	mediator complex subunit 1	-0.249	0.0140
207394_at	*ZCCHC11*	zinc finger protein 137, pseudogene	-0.559	0.0142
211558_s_at	*ADGRE1*	deoxyhypusine synthase	-0.318	0.0143
202043_s_at	*UPP1*	spermine synthase	-0.357	0.0144
208651_x_at	*ITGB1BP1*	CD24 molecule	-0.606	0.0145
213654_at	*CDIPT*	TATA-box binding protein associated factor 5 like	-0.211	0.0145
217373_x_at	*EIF3CL///EIF3C*	MDM2 proto-oncogene	-0.338	0.0145
215903_s_at	*NDST3*	microtubule associated serine/threonine kinase 2	-0.576	0.0145
214414_x_at	*ZFHX3*	hemoglobin subunit alpha 2///hemoglobin subunit alpha 1	-1.707	0.0145
201979_s_at	*BTG1*	protein phosphatase 5 catalytic subunit	-0.224	0.0146
205046_at	*KDM2A*	centromere protein E	-0.722	0.0146
213017_at	*TBL3*	abhydrolase domain containing 3	-0.448	0.0147
214190_x_at	*BIRC5*	golgi associated, gamma adaptin ear containing, ARF binding protein 2	-0.198	0.0147
207331_at	*AGO1*	centromere protein F	-0.514	0.0147
218528_s_at	*DHX9*	ring finger protein 38	-0.280	0.0148
207121_s_at	*C17orf80*	mitogen-activated protein kinase 6	-0.295	0.0149
220154_at	*SORBS3*	dystonin	-0.343	0.0149
220941_s_at	*ELK4*	chromosome 21 open reading frame 91	-0.620	0.0149
212934_at	*ITGA3*	UBX domain protein 2B	-0.437	0.0150
211744_s_at	*MRPS14*	CD58 molecule	-0.753	0.0150
218764_at	*ACTN2*	protein kinase C eta	-1.107	0.0150
218696_at	*NAT1*	eukaryotic translation initiation factor 2 alpha kinase 3	-0.344	0.0151
218793_s_at	*CRYBA2*	sex comb on midleg-like 1 (Drosophila)	-1.239	0.0151
211271_x_at	*SAMM50*	microRNA 4745///polypyrimidine tract binding protein 1	-0.257	0.0152
221245_s_at	*ANAPC5*	frizzled class receptor 5	-0.692	0.0153
204950_at	*C11orf57*	caspase recruitment domain family member 8	-0.612	0.0153
205850_s_at	*SLC22A18*	gamma-aminobutyric acid type A receptor beta3 subunit	-0.660	0.0154
202466_at	*CCDC57*	poly(A) RNA polymerase D7, non-canonical	-0.471	0.0154
214649_s_at	*PPP1R3D*	myotubularin related protein 2	-0.540	0.0154
219036_at	*LMO3*	centrosomal protein 70	-0.532	0.0154
219931_s_at	*FTCD*	kelch like family member 12	-0.349	0.0154
211122_s_at	*LGALS13*	C-X-C motif chemokine ligand 11	-1.016	0.0154
213391_at	*ALG6*	dpy-19 like 4 (C. elegans)	-0.401	0.0155
204315_s_at	*CXCL9*	G2 and S-phase expressed 1	-0.692	0.0156

208079_s_at	*PRPF18*	aurora kinase A	-0.641	0.0156
215773_x_at	*CKMT2*	poly(ADP-ribose) polymerase 2	-0.333	0.0156
212377_s_at	*MYLIP*	notch 2	-1.263	0.0156
202529_at	*IGLL3P*	phosphoribosyl pyrophosphate synthetase associated protein 1	-0.288	0.0157
202579_x_at	*LOC102724985///PDXDC1*	high mobility group nucleosomal binding domain 4	-0.293	0.0157
209147_s_at	*MADD*	phospholipid phosphatase 1	-0.490	0.0159
215739_s_at	*CDK12*	tubulin gamma complex associated protein 3	-0.467	0.0160
200712_s_at	*TULP3*	microtubule associated protein RP/EB family member 1	-0.463	0.0160
201910_at	*TELO2*	FERM, ARH/RhoGEF and pleckstrin domain protein 1	-0.761	0.0160
205260_s_at	*NSUN5P1*	acylphosphatase 1	-0.345	0.0160
212220_at	*KIFC1*	proteasome activator subunit 4	-0.432	0.0160
200693_at	*ZNF273*	tyrosine 3-monooxygenase/tryptophan 5-monooxygenase activation protein theta	-0.273	0.0162
222162_s_at	*JUND*	ADAM metallopeptidase with thrombospondin type 1 motif 1	-0.884	0.0162
202802_at	*ABHD14A-ACY1///ACY1*	deoxyhypusine synthase	-0.354	0.0163
211537_x_at	*GNAZ*	mitogen-activated protein kinase kinase kinase 7	-0.388	0.0163
211593_s_at	*SUPT16H*	microtubule associated serine/threonine kinase 2	-0.537	0.0163
200594_x_at	*TMX2*	heterogeneous nuclear ribonucleoprotein U	-0.180	0.0163
212291_at	*PSMD8*	homeodomain interacting protein kinase 1	-0.295	0.0164
214710_s_at	*RBCK1*	cyclin B1	-0.658	0.0164
211801_x_at	*NT5DC3*	mitofusin 1	-0.307	0.0164
206701_x_at	*POLR3C*	endothelin receptor type B	-0.551	0.0165
203099_s_at	*MTOR*	chromodomain Y-like	-0.418	0.0166
202240_at	*VAX2*	polo like kinase 1	-0.361	0.0166
205292_s_at	*SGSH*	heterogeneous nuclear ribonucleoprotein A2/B1	-0.207	0.0168
211793_s_at	*TRIM68*	abl-interactor 2	-0.234	0.0168
216379_x_at	*PATZ1*	CD24 molecule	-0.570	0.0168
204684_at	*HMP19*	neuronal pentraxin 1	-1.891	0.0168
212840_at	*MRPL12*	UBX domain protein 7	-0.406	0.0169
203212_s_at	*CDC20*	myotubularin related protein 2	-0.959	0.0169
210231_x_at	*RHOQ*	SET nuclear proto-oncogene	-0.179	0.0169
209653_at	*CYP3A5*	karyopherin subunit alpha 4	-0.366	0.0171
215285_s_at	*RUNDC3A*	putative homeodomain transcription factor 1	-0.680	0.0172
76897_s_at	*NCOA2*	FK506 binding protein 15	-0.338	0.0172
202583_s_at	*RPARP-AS1*	RAN binding protein 9	-0.393	0.0173
213971_s_at	*TMCC1*	SUZ12 polycomb repressive complex 2 subunit pseudogene 1///SUZ12 polycomb repressive complex 2 subunit	-0.223	0.0174
203521_s_at	*PHF8*	zinc finger protein 318	-0.290	0.0174
202156_s_at	*KCNN1*	CUGBP, Elav-like family member 2	-0.655	0.0174
208627_s_at	*FECH*	Y-box binding protein 1	-0.289	0.0175

213175_s_at	*MIR6513///TMBIM1*	small nuclear ribonucleoprotein polypeptides B and B1	-0.239	0.0175
209771_x_at	*OGFOD1*	CD24 molecule	-0.550	0.0177
202449_s_at	*TNPO1*	retinoid X receptor alpha	-0.730	0.0177
206102_at	*SMPDL3A*	GINS complex subunit 1	-0.402	0.0178
202391_at	*ABCG1*	brain abundant membrane attached signal protein 1	-1.892	0.0178
219531_at	*SNRK*	centrosomal protein 72	-0.436	0.0180
215524_x_at	*MAPK8IP2*	uncharacterized protein MGC40069///T-cell receptor alpha constant///T cell receptor alpha joining 17///T cell receptor alpha variable 20///T cell receptor delta variable 2///YME1 like 1 ATPase	-0.205	0.0180
209153_s_at	*PBLD*	transcription factor 3	-0.453	0.0181
212262_at	*ADNP2*	QKI, KH domain containing RNA binding	-0.721	0.0181
217208_s_at	*VPS51*	discs large MAGUK scaffold protein 1	-0.595	0.0181
212704_at	*CDKN2A*	zinc finger CCHC-type containing 11	-0.405	0.0181
215882_at	*S100A6*	centrosomal protein 152	-0.692	0.0182
220429_at	*C3orf52*	N-deacetylase and N-sulfotransferase 3	-1.058	0.0182
208739_x_at	*VEGFB*	small ubiquitin-related modifier 2 pseudogene///small ubiquitin-like modifier 2///small ubiquitin-like modifier 3	-0.309	0.0184
200920_s_at	*HSD17B12*	BTG anti-proliferation factor 1	-0.602	0.0184
200898_s_at	*HMOX2*	meningioma expressed antigen 5 (hyaluronidase)	-0.426	0.0184
202094_at	*ANKRD28*	baculoviral IAP repeat containing 5	-0.680	0.0184
204018_x_at	*FMO1*	hemoglobin subunit alpha 2///hemoglobin subunit alpha 1	-1.117	0.0185
211227_s_at	*RFX3*	protocadherin 11 Y-linked///protocadherin 11 X-linked	-0.508	0.0186
217414_x_at	*SBNO1*	hemoglobin subunit alpha 2///hemoglobin subunit alpha 1	-1.205	0.0186
206315_at	*IFT57*	cytokine receptor like factor 1	-1.211	0.0186
212105_s_at	*PKNOX2*	DEAH-box helicase 9	-0.450	0.0186
200608_s_at	*GSTP1*	RAD21 cohesin complex component	-0.384	0.0187
266_s_at	*TPH1*	CD24 molecule	-0.615	0.0188
214422_at	*ETFDH*	RAD23 homolog B, nucleotide excision repair protein	-0.237	0.0188
214831_at	*FBXL7*	ELK4, ETS transcription factor	-0.267	0.0189
213963_s_at	*FGFR1OP*	Sin3A associated protein 30	-0.249	0.0189
203800_s_at	*ARAP1*	mitochondrial ribosomal protein S14	-0.324	0.0189
208959_s_at	*PCNX2*	endoplasmic reticulum protein 44	-0.331	0.0190
201911_s_at	*MMP11*	FERM, ARH/RhoGEF and pleckstrin domain protein 1	-0.660	0.0191
209506_s_at	*KREMEN2*	nuclear receptor subfamily 2 group F member 2///nuclear receptor subfamily 2 group F member 1	-0.721	0.0191
212265_at	*GALNT3*	QKI, KH domain containing RNA binding	-0.833	0.0191
207740_s_at	*B3GAT3*	nucleoporin 62	-0.454	0.0191

210121_at	*HSDL2*	beta-1,3-galactosyltransferase 2	-0.828	0.0192
212015_x_at	*NUP188*	microRNA 4745///polypyrimidine tract binding protein 1	-0.218	0.0192
220350_at	*MAN2A1*	zinc finger protein 235	-0.271	0.0194
201697_s_at	*E2F8*	DNA (cytosine-5-)-methyltransferase 1	-0.337	0.0195
209892_at	*WISP3*	fucosyltransferase 4	-0.792	0.0196
209679_s_at	*OSBPL2*	small cell adhesion glycoprotein	-0.338	0.0196
202294_at	*COMMD10*	stromal antigen 1	-0.316	0.0196
212514_x_at	*FTO*	DEAD-box helicase 3, X-linked	-0.353	0.0197
204554_at	*PDLIM4*	protein phosphatase 1 regulatory subunit 3D	-0.681	0.0199
209026_x_at	*THRA*	tubulin beta class I	-0.276	0.0199
222077_s_at	*RABEP2*	Rac GTPase activating protein 1	-0.502	0.0199
205036_at	*PSIP1*	LSM6 homolog, U6 small nuclear RNA and mRNA degradation associated	-0.303	0.0201
219649_at	*TAF4*	ALG6, alpha-1,3-glucosyltransferase	-0.279	0.0202
213554_s_at	*PRR5-ARHGAP8///ARHGAP8*	CDV3 homolog	-0.294	0.0202
203915_at	*KATNBL1*	C-X-C motif chemokine ligand 9	-1.448	0.0202
209938_at	*IZUMO4*	transcriptional adaptor 2A	-0.260	0.0203
201594_s_at	*MTF2*	protein phosphatase 4 regulatory subunit 1	-0.351	0.0203
215508_at	*CENPA*	BUB1 mitotic checkpoint serine/threonine kinase	-0.337	0.0204
215946_x_at	*CLP1*	immunoglobulin lambda like polypeptide 3, pseudogene	-1.347	0.0204
209656_s_at	*RUBCN*	transmembrane protein 47	-1.071	0.0206
219226_at	*TGM5*	cyclin dependent kinase 12	-0.177	0.0206
217028_at	*MTFR1*	C-X-C motif chemokine receptor 4	-0.904	0.0207
205854_at	*FAM168A*	tubby like protein 3	-0.251	0.0208
209680_s_at	*RAC3*	kinesin family member C1	-0.553	0.0208
200799_at	*WBSCR22*	heat shock protein family A (Hsp70) member 1B///heat shock protein family A (Hsp70) member 1A	-1.102	0.0209
215239_x_at	*UBE2B*	zinc finger protein 273	-0.386	0.0209
203751_x_at	*RHOT2*	JunD proto-oncogene, AP-1 transcription factor subunit	-0.335	0.0209
204993_at	*TRMT2A*	G protein subunit alpha z	-0.491	0.0210
217815_at	*EXOC7*	SPT16 homolog, facilitates chromatin remodeling subunit	-0.247	0.0210
200820_at	*NANS*	proteasome 26S subunit, non-ATPase 8	-0.215	0.0210
210573_s_at	*KCND1*	RNA polymerase III subunit C	-0.304	0.0210
214061_at	*FBXL12*	TBC1 domain family member 31	-0.565	0.0211
202207_at	*ZNF276*	ADP ribosylation factor like GTPase 4C	-0.849	0.0211
210163_at	*CFDP1*	C-X-C motif chemokine ligand 11	-0.815	0.0211
216652_s_at	*CABYR*	down-regulator of transcription 1	-0.479	0.0212
200072_s_at	*TRAP1*	heterogeneous nuclear ribonucleoprotein M	-0.232	0.0212
204597_x_at	*TIMELESS*	stanniocalcin 1	-1.006	0.0213
211391_s_at	*ZNF202*	POZ/BTB and AT hook containing zinc finger 1	-0.432	0.0215
217452_s_at	*URB1*	beta-1,3-galactosyltransferase 2	-0.813	0.0215

202870_s_at	*CYP2E1*	cell division cycle 20	-0.808	0.0216
214449_s_at	*WTH3DI///RAB6C///RAB6A*	ras homolog family member Q	-0.546	0.0216
200943_at	*KPNA1*	high mobility group nucleosome binding domain 1	-0.206	0.0216
212412_at	*ATG7*	PDZ and LIM domain 5	-0.684	0.0216
205731_s_at	*SHB*	nuclear receptor coactivator 2	-0.382	0.0217
213349_at	*BMP1*	transmembrane and coiled-coil domain family 1	-0.398	0.0217
212916_at	*RTCA*	PHD finger protein 8	-0.315	0.0217
210839_s_at	*POLR2L*	ectonucleotide pyrophosphatase/phosphodiesterase 2	-0.876	0.0218
205173_x_at	*COL5A3*	CD58 molecule	-0.815	0.0219
208096_s_at	*RAPGEFL1*	collagen type XXI alpha 1 chain	-1.387	0.0219
212635_at	*FAM46A*	transportin 1	-0.339	0.0220
201023_at	*RAB3D*	TATA-box binding protein associated factor 7	-0.449	0.0221
213624_at	*PQBP1*	sphingomyelin phosphodiesterase acid like 3A	-0.816	0.0221
209458_x_at	*SCYL2*	hemoglobin subunit alpha 2///hemoglobin subunit alpha 1	-1.095	0.0222
200666_s_at	*SYNE3///LINC00341*	DnaJ heat shock protein family (Hsp40) member B1	-0.562	0.0222
218655_s_at	*PSMB9*	CWC25 spliceosome associated protein homolog	-0.176	0.0226
207620_s_at	*NXN*	calcium/calmodulin dependent serine protein kinase	-0.373	0.0227
209710_at	*RBM5*	GATA binding protein 2	-0.609	0.0228
202516_s_at	*PLPP2*	discs large MAGUK scaffold protein 1	-0.497	0.0228
209481_at	*BICD1*	SNF related kinase	-0.444	0.0228
208603_s_at	*CLOCK*	mitogen-activated protein kinase 8 interacting protein 2	-0.256	0.0228
209786_at	*ARNTL2*	high mobility group nucleosomal binding domain 4	-0.326	0.0229
203321_s_at	*IQGAP1*	ADNP homeobox 2	-0.225	0.0229
213341_at	*MSL2*	fem-1 homolog C	-0.318	0.0229
209644_x_at	*RASA2*	cyclin dependent kinase inhibitor 2A	-0.527	0.0230
210416_s_at	*DTL*	checkpoint kinase 2	-0.443	0.0231
202514_at	*PTENP1*	discs large MAGUK scaffold protein 1	-0.485	0.0234
203248_at	*UBE2V2*	zinc finger protein 24	-0.243	0.0234
219474_at	*KIF26B*	chromosome 3 open reading frame 52	-0.209	0.0235
218829_s_at	*METTL8*	chromodomain helicase DNA binding protein 7	-0.661	0.0235
215469_at	*ZSCAN9*	SLIT and NTRK like family member 5	-1.061	0.0235
209678_s_at	*STK16*	protein kinase C iota	-0.362	0.0236
218236_s_at	*NBN*	protein kinase D3	-0.858	0.0236
209744_x_at	*THAP12*	itchy E3 ubiquitin protein ligase	-0.270	0.0237
212072_s_at	*OIP5*	casein kinase 2 alpha 1	-0.217	0.0238
202123_s_at	*TINF2*	ABL proto-oncogene 1, non-receptor tyrosine kinase	-0.253	0.0238
221958_s_at	*PNMA3*	wntless Wnt ligand secretion mediator	-0.629	0.0238
212770_at	*RAB40A*	transducin like enhancer of split 3	-0.366	0.0239
207234_at	*SRP9*	regulatory factor X3	-0.181	0.0240

218100_s_at	*SRPRA*	intraflagellar transport 57	-1.105	0.0240
217641_at	*ATP5I*	G protein-coupled receptor 135	-0.419	0.0241
202559_x_at	*PDK3*	chromatin target of PRMT1	-0.260	0.0242
214512_s_at	*SERPINA6*	SUB1 homolog, transcriptional regulator	-0.383	0.0243
213249_at	*GUSB*	F-box and leucine rich repeat protein 7	-0.990	0.0243
205588_s_at	*PKIA*	FGFR1 oncogene partner	-0.377	0.0243
219409_at	*CYP7B1*	Smad nuclear interacting protein 1	-0.202	0.0244
219759_at	*MCC*	endoplasmic reticulum aminopeptidase 2	-1.131	0.0245
204273_at	*PAX6*	endothelin receptor type B	-1.012	0.0247
219463_at	*SMARCA2*	lysosomal associated membrane protein family member 5	-0.366	0.0248
202443_x_at	*PBK*	notch 2	-1.129	0.0248
215436_at	*ANO3*	hydroxysteroid dehydrogenase like 2	-0.281	0.0249
212691_at	*HIKESHI*	nucleoporin 188	-0.223	0.0249
217946_s_at	*DNAJC16*	SUMO1 activating enzyme subunit 1	-0.334	0.0249
205882_x_at	*CYFIP1*	adducin 3	-0.711	0.0249
205934_at	*OXT*	phospholipase C like 1	-0.650	0.0249
213528_at	*IL1B*	methyltransferase like 18	-0.408	0.0249
205105_at	*PRDX1*	mannosidase alpha class 2A member 1	-0.403	0.0250
203334_at	*GRIN1*	DEAH-box helicase 8	-0.178	0.0250
213074_at	*PTTG1IP*	pleckstrin homology domain interacting protein	-0.297	0.0251
219990_at	*ADAMTS5*	E2F transcription factor 8	-0.692	0.0251
218439_s_at	*LYPLA1*	COMM domain containing 10	-0.476	0.0252
213497_at	*TAF10*	ankyrin repeat and BTB domain containing 2	-0.364	0.0252
207719_x_at	*ASAP1*	centrosomal protein 170 pseudogene 1///centrosomal protein 170	-0.315	0.0253
205741_s_at	*SPRY1*	dystrobrevin alpha	-0.606	0.0254
208336_s_at	*SLC46A3*	trans-2,3-enoyl-CoA reductase	-0.278	0.0258
209374_s_at	*PCLO*	immunoglobulin heavy constant mu	-2.066	0.0258
220358_at	*STAP2*	basic leucine zipper ATF-like transcription factor 3	-0.264	0.0259
216511_s_at	*PLA2G12A*	transcription factor 7 like 2	-0.459	0.0259
205961_s_at	*LHX6*	PC4 and SFRS1 interacting protein 1	-0.414	0.0260
205603_s_at	*GRB7*	diaphanous related formin 2	-0.830	0.0260
213090_s_at	*GTF2E1*	TATA-box binding protein associated factor 4	-0.235	0.0260
218791_s_at	*HNRNPDL*	katanin regulatory subunit B1 like 1	-0.330	0.0261
203347_s_at	*SDHC*	metal response element binding transcription factor 2	-0.311	0.0262
214221_at	*DNPEP*	ALMS1, centrosome and basal body associated protein	-0.321	0.0262
200703_at	*B4GAT1*	dynein light chain LC8-type 1	-0.344	0.0264
211989_at	*DEK*	SWI/SNF related, matrix associated, actin dependent regulator of chromatin, subfamily e, member 1	-0.285	0.0264
210821_x_at	*GZMB*	centromere protein A	-0.463	0.0264
212141_at	*BUB1B*	minichromosome maintenance complex component 4	-0.459	0.0266
218009_s_at	*NDST2*	protein regulator of cytokinesis 1	-0.580	0.0266
204595_s_at	*CENPU*	stanniocalcin 1	-0.910	0.0266

212142_at	*LINC00965*	minichromosome maintenance complex component 4	-0.481	0.0268
203814_s_at	*FAM63A*	NAD(P)H quinone dehydrogenase 2	-0.621	0.0268
210095_s_at	*LOC102724229///RASA4B///RASA4*	insulin like growth factor binding protein 3	-1.077	0.0268
203208_s_at	*CLUAP1*	mitochondrial fission regulator 1	-0.647	0.0269
213518_at	*UBD///GABBR1*	protein kinase C iota	-0.267	0.0274
215708_s_at	*RNF121*	primase (DNA) subunit 2 pseudogene///primase (DNA) subunit 2	-0.540	0.0275
204752_x_at	*C1D*	poly(ADP-ribose) polymerase 2	-0.299	0.0275
222037_at	*TPRKB*	minichromosome maintenance complex component 4	-0.455	0.0276
202581_at	*ATF6B*	heat shock protein family A (Hsp70) member 1B///heat shock protein family A (Hsp70) member 1A	-0.758	0.0278
217370_x_at	*ZNF267*	FUS RNA binding protein	-0.283	0.0278
209787_s_at	*ARHGAP11A*	high mobility group nucleosomal binding domain 4	-0.309	0.0279
211804_s_at	*C1RL*	cyclin dependent kinase 2	-0.456	0.0279
203207_s_at	*SNX3*	mitochondrial fission regulator 1	-0.626	0.0280
220127_s_at	*UBE2S*	F-box and leucine rich repeat protein 12	-0.267	0.0280
203715_at	*PCMTD2*	tubulin folding cofactor E	-0.353	0.0281
203166_at	*UAP1*	craniofacial development protein 1	-0.232	0.0282
219928_s_at	*ERAL1*	calcium binding tyrosine phosphorylation regulated	-0.264	0.0283
211699_x_at	*EPHB6*	hemoglobin subunit alpha 2///hemoglobin subunit alpha 1	-1.045	0.0284
208644_at	*RNF40*	poly(ADP-ribose) polymerase 1	-0.220	0.0284
200844_s_at	*RWDD3*	peroxiredoxin 6	-0.384	0.0284
211647_x_at	*ZMYM6*	immunoglobulin heavy constant mu///immunoglobulin heavy constant gamma 1 (G1m marker)	-0.305	0.0284
203046_s_at	*HNRNPH3*	timeless circadian clock	-0.259	0.0284
202056_at	*PRKRA*	karyopherin subunit alpha 1	-0.282	0.0286
204805_s_at	*TUSC2*	H1 histone family member X	-0.314	0.0286
213405_at	*DDOST*	RAB22A, member RAS oncogene family	-0.279	0.0286
215351_at	*ZNF230*	RNA 3'-terminal phosphate cyclase	-0.340	0.0288
202194_at	*AGRN*	transmembrane p24 trafficking protein 5	-0.318	0.0288
203044_at	*KIF13A*	chondroitin sulfate synthase 1	-0.372	0.0288
207469_s_at	*ROCK2*	pirin	-1.230	0.0290
201752_s_at	*RBM23*	adducin 3	-0.606	0.0290
202206_at	*AGFG2*	ADP ribosylation factor like GTPase 4C	-0.837	0.0290
221766_s_at	*SMIM7*	family with sequence similarity 46 member A	-0.850	0.0293
209392_at	*RFXANK*	ectonucleotide pyrophosphatase/phosphodiesterase 2	-0.971	0.0294
221220_s_at	*TUBB4A*	SCY1 like pseudokinase 2	-0.338	0.0294
202705_at	*HMCES*	cyclin B2	-0.584	0.0294

207039_at	*SLC26A2*	cyclin dependent kinase inhibitor 2A	-0.465	0.0295
219080_s_at	*NDN*	CTP synthase 2	-0.260	0.0296
219972_s_at	*THEG*	pecanex homolog 4 (Drosophila)	-0.345	0.0296
204279_at	*PIK3R3*	proteasome subunit beta 9	-1.101	0.0297
214806_at	*RCN3*	BICD cargo adaptor 1	-0.609	0.0299
204980_at	*ZNF155*	clock circadian regulator	-0.328	0.0299
220658_s_at	*ATP2C2*	aryl hydrocarbon receptor nuclear translocator like 2	-0.499	0.0299
206316_s_at	*VIPR1*	kinetochore associated 1	-0.215	0.0299
210840_s_at	*TOPBP1*	IQ motif containing GTPase activating protein 1	-0.498	0.0299
212542_s_at	*COMP*	pleckstrin homology domain interacting protein	-0.308	0.0301
207831_x_at	*TUBD1*	deoxyhypusine synthase	-0.279	0.0301
218733_at	*ZNF468*	male-specific lethal 2 homolog (Drosophila)	-0.296	0.0301
202726_at	*VASP*	DNA ligase 1	-0.274	0.0301
206636_at	*MRPL9*	RAS p21 protein activator 2	-0.363	0.0302
200921_s_at	*PAM16*	BTG anti-proliferation factor 1	-0.514	0.0302
221279_at	*CDC42BPA*	ganglioside induced differentiation associated protein 1	-0.353	0.0303
218585_s_at	*SLC1A1*	denticleless E3 ubiquitin protein ligase homolog	-0.368	0.0303
219441_s_at	*F12*	leucine rich repeat kinase 1	-0.514	0.0303
221925_s_at	*BDH2*	centrosome and spindle pole associated protein 1	-0.412	0.0304
207782_s_at	*COL6A1*	presenilin 1	-0.359	0.0304
204913_s_at	*MINOS1-NBL1///NBL1*	SRY-box 11	-0.696	0.0304
205696_s_at	*ADGRL2*	GDNF family receptor alpha 1	-0.377	0.0306
209226_s_at	*SLC17A4*	transportin 1	-0.297	0.0306
215493_x_at	*VSNL1*	butyrophilin subfamily 2 member A1	-0.379	0.0307
217494_s_at	*PARD3*	phosphatase and tensin homolog pseudogene 1	-0.317	0.0307
209096_at	*NOS3*	ubiquitin conjugating enzyme E2 V2	-0.386	0.0307
203214_x_at	*LOC100506718///FLRT2*	cyclin dependent kinase 1	-0.626	0.0307
216321_s_at	*SETSIP///SET*	nuclear receptor subfamily 3 group C member 1	-0.708	0.0307
205181_at	*NR2F2*	zinc finger and SCAN domain containing 9	-0.385	0.0309
202907_s_at	*ANGEL2*	nibrin	-0.377	0.0310
207304_at	*LTBP1*	zinc finger protein 45	-0.453	0.0312
210191_s_at	*CHD1*	putative homeodomain transcription factor 1	-0.786	0.0312
219698_s_at	*IGSF3*	methyltransferase like 4	-0.324	0.0313
217196_s_at	*WDR47*	calmodulin regulated spectrin associated protein family member 2	-0.725	0.0313
213599_at	*EMX2*	Opa interacting protein 5	-0.672	0.0313
221455_s_at	*AP1M2*	uncharacterized LOC101929777///Wnt family member 3	-0.269	0.0314
220052_s_at	*SNORD54///RPS20*	TERF1 interacting nuclear factor 2	-0.220	0.0314
213226_at	*USP7*	cyclin A2	-0.529	0.0314
201273_s_at	*AZGP1*	signal recognition particle 9	-0.241	0.0315

201853_s_at	*SIK1*	cell division cycle 25B	-0.483	0.0316
206173_x_at	*ZNF37BP*	GA binding protein transcription factor beta subunit 1	-0.348	0.0316
212447_at	*TP53BP2*	kelch repeat and BTB domain containing 2	-0.272	0.0318
213865_at	*APOLD1*	discoidin, CUB and LCCL domain containing 2	-0.277	0.0318
206347_at	*SEL1L*	pyruvate dehydrogenase kinase 3	-0.237	0.0319
201637_s_at	*ORC6*	FMR1 autosomal homolog 1	-0.250	0.0319
217403_s_at	*NAT9*	zinc finger protein 227	-0.297	0.0320
202605_at	*CSE1L*	glucuronidase beta	-0.271	0.0320
204612_at	*TSKU*	protein kinase (cAMP-dependent, catalytic) inhibitor alpha	-0.778	0.0320
211299_s_at	*NGDN*	flotillin 2	-0.260	0.0321
206132_at	*CSNK1D*	mutated in colorectal cancers	-0.462	0.0322
217765_at	*SLC43A1*	nuclear receptor binding protein 1	-0.224	0.0323
205646_s_at	*NIPAL3*	paired box 6	-1.267	0.0324
210101_x_at	*ZFP69B*	SH3 domain containing GRB2 like endophilin B1	-0.408	0.0325
201517_at	*SLC7A4*	nuclear cap binding protein subunit 2	-0.230	0.0325
206966_s_at	*ADNP*	Kruppel like factor 12	-0.308	0.0326
206544_x_at	*BTG3*	SWI/SNF related, matrix associated, actin dependent regulator of chromatin, subfamily a, member 2	-0.628	0.0328
219148_at	*GDE1*	PDZ binding kinase	-0.818	0.0328
210611_s_at	*TGOLN2*	dystrobrevin alpha	-0.531	0.0329
201668_x_at	*EFCAB11*	myristoylated alanine rich protein kinase C substrate	-0.306	0.0329
208923_at	*BAZ1B*	cytoplasmic FMR1 interacting protein 1	-0.538	0.0330
221677_s_at	*MKI67*	downstream neighbor of SON	-0.289	0.0332
208680_at	*TSC2*	peroxiredoxin 1	-0.266	0.0332
216306_x_at	*PDHB*	microRNA 4745///polypyrimidine tract binding protein 1	-0.228	0.0333
212448_at	*ELAVL3*	neural precursor cell expressed, developmentally down-regulated 4-like, E3 ubiquitin protein ligase	-0.901	0.0334
219935_at	*BAP1*	ADAM metallopeptidase with thrombospondin type 1 motif 5	-1.148	0.0334
203007_x_at	*CENPN*	lysophospholipase I	-0.507	0.0339
209592_s_at	*GADD45A*	DDB1 and CUL4 associated factor 7	-0.402	0.0339
209218_at	*LRRC15*	squalene epoxidase	-0.750	0.0339
221039_s_at	*AZIN1*	ArfGAP with SH3 domain, ankyrin repeat and PH domain 1	-0.686	0.0340
218344_s_at	*MYL5*	REST corepressor 3	-0.431	0.0341
212558_at	*TALDO1*	sprouty RTK signaling antagonist 1	-0.469	0.0342
208876_s_at	*TCF4*	p21 (RAC1) activated kinase 2	-0.204	0.0342
207983_s_at	*S1PR1*	stromal antigen 2	-0.275	0.0343
212887_at	*PFKFB2*	Sec23 homolog A, coat complex II component	-0.597	0.0344

213761_at	*LOC102724197///LOC100132705///GGT2///GGTLC1///GGTLC2///GGT3P///GGT1*	Mdm1 nuclear protein	-0.769	0.0347
218710_at	*KCNC4*	tetratricopeptide repeat domain 27	-0.330	0.0349
205930_at	*CYB5R1*	general transcription factor IIE subunit 1	-0.219	0.0349
210559_s_at	*PYCR1*	cyclin dependent kinase 1	-0.590	0.0350
209067_s_at	*SYBU*	heterogeneous nuclear ribonucleoprotein D like	-0.269	0.0351
201417_at	*PRKAR1A*	SRY-box 4	-0.399	0.0351
217993_s_at	*COX20*	methionine adenosyltransferase 2B	-0.371	0.0351
212867_at	*SPRED2*	nuclear receptor coactivator 2	-0.467	0.0351
202004_x_at	*NMT1*	succinate dehydrogenase complex subunit C	-0.301	0.0351
212107_s_at	*TGM2*	DEAH-box helicase 9	-0.290	0.0352
211714_x_at	*TSGA10*	tubulin beta class I	-0.262	0.0352
200934_at	*SLC35C1*	DEK proto-oncogene	-0.356	0.0353
210164_at	*PNPLA4*	granzyme B	-1.079	0.0353
220471_s_at	*ATG5*	myc target 1	-0.500	0.0353
218172_s_at	*RAB3GAP2*	derlin 1	-0.446	0.0353
203755_at	*SYNE2*	BUB1 mitotic checkpoint serine/threonine kinase B	-0.509	0.0354
218883_s_at	*TCF25*	centromere protein U	-0.385	0.0356
214669_x_at	*NAMPT*	immunoglobulin kappa constant	-2.276	0.0356
212196_at	*MAGEA9B///MAGEA9*	interleukin 6 signal transducer	-0.416	0.0356
212117_at	*PADI2*	ras homolog family member Q	-0.358	0.0356
219054_at	*PSMC2*	natriuretic peptide receptor 3	-0.860	0.0357
217286_s_at	*PXDC1*	NDRG family member 3	-0.283	0.0357
218558_s_at	*CTR9*	mitochondrial ribosomal protein L39	-0.265	0.0358
203554_x_at	*COX11*	pituitary tumor-transforming 1	-0.501	0.0360
201645_at	*CTTN*	tenascin C	-1.497	0.0360
212257_s_at	*ELMO3*	SWI/SNF related, matrix associated, actin dependent regulator of chromatin, subfamily a, member 2	-0.535	0.0360
206409_at	*SDC4*	T-cell lymphoma invasion and metastasis 1	-0.293	0.0361
219094_at	*FGF18*	armadillo repeat containing 8	-0.569	0.0361
211075_s_at	*FOSL1*	CD47 molecule	-1.054	0.0361
202435_s_at	*TUG1*	cytochrome P450 family 1 subfamily B member 1	-0.803	0.0361
205890_s_at	*KHDRBS3*	ubiquitin D///gamma-aminobutyric acid type B receptor subunit 1	-1.213	0.0361
218443_s_at	*CHPF2*	DAZ associated protein 1	-0.205	0.0362
219882_at	*RBM19*	tubulin tyrosine ligase like 7	-0.507	0.0362
202158_s_at	*KLHL7*	CUGBP, Elav-like family member 2	-0.538	0.0362
200056_s_at	*SH3GL3*	C1D nuclear receptor corepressor	-0.210	0.0365
210117_at	*PRAME*	sperm associated antigen 1	-0.774	0.0366
219030_at	*NOP14-AS1*	TP53RK binding protein	-0.320	0.0366
209997_x_at	*GPATCH1*	pericentriolar material 1	-0.297	0.0366

214075_at	*CSNK1G1*	neudesin neurotrophic factor	-0.271	0.0367

Supplementary Table S7. Differentially expressed genes between MCC primay tumors and metastases in the MCC GEO cohort GSE39612 (cut off p<0.05 & fold change >=1.5)

ID	Gene symbol	Gene title	logFC	*P-v* alue
Over-expressed genes in Metastases vs. Primary MCC tumors				
222020_s_at	*LOC102725271 ///NTM*	neurotrimin-like///neurotrimin	0.917	4.8E-05
230147_at	*F2RL2*	coagulation factor II thrombin receptor like 2	2.255	6.8E-05
211824_x_at	*NLRP1*	NLR family pyrin domain containing 1	0.902	0.0001
203417_at	*MFAP2*	microfibrillar associated protein 2	1.555	0.0001
201792_at	*AEBP1*	AE binding protein 1	1.562	0.0001
202450_s_at	*CTSK*	cathepsin K	1.932	0.0002
227383_at	*LOC727820*	uncharacterized LOC727820	1.317	0.0002
217763_s_at	*RAB31*	RAB31, member RAS oncogene family	1.396	0.0002
201960_s_at	*MYCBP2*	MYC binding protein 2, E3 ubiquitin protein ligase	0.518	0.0002
225016_at	*APCDD1*	APC down-regulated 1	2.239	0.0003
223121_s_at	*SFRP2*	secreted frizzled related protein 2	2.347	0.0003
235944_at	*HMCN1*	hemicentin 1	1.430	0.0003
229404_at	*TWIST2*	twist family bHLH transcription factor 2	1.482	0.0003
227384_s_at	*MMP28*	uncharacterized LOC727820	1.405	0.0003
239273_s_at	*COL8A2*	matrix metallopeptidase 28	0.897	0.0004
211062_s_at	*MFSD4A*	G protein-coupled receptor 78///carboxypeptidase Z	0.759	0.0004
221900_at	*CAPG*	collagen type VIII alpha 2 chain	1.217	0.0004
201850_at	*RNA45S5*	capping actin protein, gelsolin like	1.262	0.0005
201373_at	*IQSEC1*	plectin	0.679	0.0006
203907_s_at	*LAMA2*	IQ motif and Sec7 domain 1	0.512	0.0007
219764_at	*LOC441179*	frizzled class receptor 10	1.292	0.0007
216840_s_at	*ST7L*	laminin subunit alpha 2	1.177	0.0007
217764_s_at	*LOC153811///RNF130*	RAB31, member RAS oncogene family	1.206	0.0007
213909_at	*KIF26B*	leucine rich repeat containing 15	2.020	0.0007
227140_at	*HOXA1*	inhibin beta A subunit	1.428	0.0008
238617_at	*ZNF451*	kinesin family member 26B	1.085	0.0008
223122_s_at	*HTRA1*	secreted frizzled related protein 2	2.765	0.0008
214454_at	*SNORD3D///SNORD3C///SNORD3B-2///SNORD3A///SNORD3B-1*	ADAM metallopeptidase with thrombospondin type 1 motif 2	0.624	0.0008
214639_s_at	*RFTN1*	homeobox A1	0.563	0.0009
201185_at	*IDS*	HtrA serine peptidase 1	1.050	0.0010
212646_at	*MAF*	raftlin, lipid raft linker 1	1.000	0.0011
212190_at	*EGFLAM*	serpin family E member 2	1.157	0.0012
212221_x_at	*EFEMP2*	iduronate 2-sulfatase	0.655	0.0012
214020_x_at	*CD109*	integrin subunit beta 5	0.911	0.0012
210113_s_at	*COL3A1*	NLR family pyrin domain containing 1	0.790	0.0012

206363_at	*MAGIX*	MAF bZIP transcription factor	1.278	0.0013
226911_at	*RBMS3*	EGF like, fibronectin type III and laminin G domains	0.705	0.0013
206580_s_at	*TMEM204*	EGF containing fibulin like extracellular matrix protein 2	1.046	0.0014
226545_at	*HELZ2*	CD109 molecule	1.532	0.0014
232458_at	*CDH11*	collagen type III alpha 1 chain	2.050	0.0014
241789_at	*MIR6834///PFDN6*	RNA binding motif single stranded interacting protein 3	0.769	0.0015
219315_s_at	*LFNG*	transmembrane protein 204	0.737	0.0015
228230_at	*NUMBL*	helicase with zinc finger 2	0.574	0.0015
207172_s_at	*ABCB4*	cadherin 11	1.005	0.0016
212091_s_at	*HDAC2*	collagen type VI alpha 1 chain	1.136	0.0017
226695_at	*COL6A2*	paired related homeobox 1	1.648	0.0018
215270_at	*DIO2*	LFNG O-fucosylpeptide 3-beta-N-acetylglucosaminyltransferase	0.426	0.0019
207819_s_at	*ZBTB45*	ATP binding cassette subfamily B member 4	0.610	0.0020
213519_s_at	*ACER3*	laminin subunit alpha 2	0.909	0.0021
209156_s_at	*ADGRA2*	collagen type VI alpha 2 chain	1.332	0.0021
229554_at	*GLI3*	lumican	1.394	0.0021
203700_s_at	*PDE4A*	deiodinase, iodothyronine, type II	1.464	0.0021
222687_s_at	*MAST3*	alkaline ceramidase 3	0.688	0.0025
219463_at	*FZD2*	lysosomal associated membrane protein family member 5	1.046	0.0025
221814_at	*SUGCT*	adhesion G protein-coupled receptor A2	0.676	0.0026
227376_at	*THBS2*	GLI family zinc finger 3	1.138	0.0026
211447_s_at	*AHR*	phosphodiesterase 4A	0.288	0.0027
202465_at	*RMDN1*	procollagen C-endopeptidase enhancer	1.204	0.0027
213045_at	*MXRA8*	microtubule associated serine/threonine kinase 3	0.459	0.0028
213428_s_at	*CHPF2*	collagen type VI alpha 1 chain	1.193	0.0028
210220_at	*SLC6A6*	frizzled class receptor 2	0.842	0.0028
219655_at	*MXRA5*	succinyl-CoA:glutarate-CoA transferase	0.647	0.0029
203083_at	*GSX2*	thrombospondin 2	1.292	0.0029
202820_at	*KRTAP4-11*	aryl hydrocarbon receptor	0.861	0.0030
222665_at	*ZCCHC24*	regulator of microtubule dynamics 1	0.358	0.0031
201069_at	*C19orf66*	matrix metallopeptidase 2	1.592	0.0031
213422_s_at	*SPRED1*	matrix remodeling associated 8	0.861	0.0032
221799_at	*NEK3*	chondroitin polymerizing factor 2	0.451	0.0032
228754_at	*SCARF2*	solute carrier family 6 member 6	0.909	0.0033
201959_s_at	*SSPN*	MYC binding protein 2, E3 ubiquitin protein ligase	0.483	0.0033
209596_at	*LEP*	matrix remodeling associated 5	1.628	0.0033
212419_at	*ZNF692*	zinc finger CCHC-type containing 24	0.971	0.0033
1555491_a_at	*FAM134A*	chromosome 19 open reading frame 66	0.430	0.0034
235074_at	*ITGBL1*	sprouty related EVH1 domain containing 1	0.566	0.0034
213116_at	*ASPN*	NIMA related kinase 3	0.733	0.0034
201744_s_at	*MIR6787///SLC16A3*	lumican	1.929	0.0035

227557_at	*UQCC2*	scavenger receptor class F member 2	0.590	0.0035
204964_s_at	*SGO1*	sarcospan	0.710	0.0036
217762_s_at	*LAT*	RAB31, member RAS oncogene family	1.077	0.0036
225320_at	*CLCNKB*	mitochondrial calcium uniporter	0.325	0.0037
222129_at	*PKP2*	family with sequence similarity 134 member A	0.534	0.0038
214927_at	*CCDC88A*	integrin subunit beta like 1	0.864	0.0038
219087_at	*LGALS8*	asporin	1.587	0.0038
202855_s_at	*TPTEP1*	microRNA 6787///solute carrier family 16 member 3	0.472	0.0038
211005_at	*APOBEC3C*	linker for activation of T-cells	0.841	0.0039
229947_at	*ABCB9*	peptidase inhibitor 15	1.298	0.0039
232297_at	*LINC00619*	kelch like family member 5	0.663	0.0040
55093_at	*CAP1*	chondroitin polymerizing factor 2	0.395	0.0041
221078_s_at	*FMO5*	coiled-coil domain containing 88A	0.390	0.0041
210732_s_at	*RNF175*	galectin 8	0.415	0.0042
230085_at	*RASA3*	pyruvate dehydrogenase kinase 3	0.752	0.0044
204976_s_at	*VPS33A*	Alport syndrome, mental retardation, midface hypoplasia and elliptocytosis chromosomal region gene 1	0.698	0.0044
209584_x_at	*PET117*	apolipoprotein B mRNA editing enzyme catalytic subunit 3C	0.668	0.0044
213800_at	*OLFML3*	complement factor H	1.505	0.0045
208022_s_at	*SLC38A10*	cell division cycle 14B	0.689	0.0045
213798_s_at	*CCDC80*	adenylate cyclase associated protein 1	0.494	0.0045
230389_at	*PDXK*	formin binding protein 1	0.802	0.0045
236465_at	*SRPRA*	ring finger protein 175	0.488	0.0045
213940_s_at	*ZNF346*	formin binding protein 1	0.742	0.0045
222484_s_at	*DDR2*	C-X-C motif chemokine ligand 14	2.315	0.0045
225562_at	*TGFBI*	RAS p21 protein activator 3	0.707	0.0046
205941_s_at	*TGFB1*	collagen type X alpha 1 chain	1.333	0.0047
218162_at	*SLC29A4*	olfactomedin like 3	1.351	0.0047
225241_at	*NAP1L4*	coiled-coil domain containing 80	1.083	0.0050
218019_s_at	*S100A4*	pyridoxal (pyridoxine, vitamin B6) kinase	0.378	0.0051
225381_at	*ANTXR1*	mir-100-let-7a-2 cluster host gene	0.998	0.0051
217428_s_at	*CAMKV*	collagen type X alpha 1 chain	1.258	0.0052
203699_s_at	*LOC105376944*	deiodinase, iodothyronine, type II	1.723	0.0052
200917_s_at	*PLEKHM2*	SRP receptor alpha subunit	0.356	0.0052
227561_at	*FBLN2*	discoidin domain receptor tyrosine kinase 2	0.650	0.0054
201506_at	*ASAP1-IT2*	transforming growth factor beta induced	1.058	0.0054
225464_at	*KCTD12*	FERM domain containing 6	0.949	0.0054
203085_s_at	*C1R*	transforming growth factor beta 1	0.962	0.0054
201438_at	*PIK3CA*	collagen type VI alpha 3 chain	1.207	0.0054
203939_at	*ZNF546*	5'-nucleotidase ecto	0.958	0.0054
231240_at	*GXYLT2*	deiodinase, iodothyronine, type II	0.812	0.0055
203186_s_at	*B2M*	S100 calcium binding protein A4	1.223	0.0056
220092_s_at	*BTN3A2*	anthrax toxin receptor 1	0.361	0.0057
208935_s_at	*MCTS1*	galectin 8	0.505	0.0057

212146_at	*IRF5*	pleckstrin homology and RUN domain containing M2	0.501	0.0057
219165_at	*TRANK1*	PDZ and LIM domain 2	0.969	0.0057
1557080_s_at	*KYAT1*	integrin subunit beta like 1	0.761	0.0058
225481_at	*HMGCLL1*	FERM domain containing 6	0.914	0.0058
203886_s_at	*CCND1*	fibulin 2	1.075	0.0058
1557685_at	*SPATS2L*	ASAP1 intronic transcript 2	0.416	0.0058
212188_at	*FAS*	potassium channel tetramerization domain containing 12	1.043	0.0059
212067_s_at	*SFXN3*	complement C1r subcomponent	1.451	0.0059
235371_at	*TBRG1*	glucoside xylosyltransferase 2	0.946	0.0060
221558_s_at	*RASSF9*	lymphoid enhancer binding factor 1	0.872	0.0060
232311_at	*CD47*	beta-2-microglobulin	1.048	0.0061
212613_at	*CCDC81*	butyrophilin subfamily 3 member A2	0.822	0.0062
203231_s_at	*SLC1A1*	ataxin 1	0.958	0.0062
205469_s_at	*H2AFJ*	interferon regulatory factor 5	0.674	0.0063
213261_at	*MBP*	tetratricopeptide repeat and ankyrin repeat containing 1	0.663	0.0063
218002_s_at	*MEIS1*	C-X-C motif chemokine ligand 14	2.322	0.0065
208711_s_at	*SZRD1*	cyclin D1	0.822	0.0066
215617_at	*TMEM200A*	spermatogenesis associated serine rich 2 like	0.781	0.0067
203232_s_at	*IL13RA1*	ataxin 1	1.052	0.0068
204781_s_at	*TMEM117*	Fas cell surface death receptor	0.682	0.0069
220974_x_at	*DICER1*	sideroflexin 3	0.690	0.0069
209365_s_at	*LOC101927929*	extracellular matrix protein 1	1.140	0.0071
226318_at	*FCHSD2*	transforming growth factor beta regulator 1	0.459	0.0071
226016_at	*WWC1*	CD47 molecule	1.071	0.0072
220936_s_at	*THY1*	H2A histone family member J	0.660	0.0073
210136_at	*AHNAK*	myelin basic protein	0.944	0.0074
225407_at	*PDSS2*	myelin basic protein	0.871	0.0074
212423_at	*GNAI2*	zinc finger CCHC-type containing 24	0.739	0.0074
219594_at	*ZNF646*	ninjurin 2	0.655	0.0074
238447_at	*VPS50*	RNA binding motif single stranded interacting protein 3	1.258	0.0075
212002_at	*SH3RF3*	SUZ RNA binding domain containing 1	0.554	0.0076
234994_at	*API5*	transmembrane protein 200A	0.879	0.0076
211612_s_at	*HEG1*	interleukin 13 receptor subunit alpha 1	0.889	0.0077
220134_x_at	*TNFRSF21*	eva-1 homolog B	0.442	0.0077
223594_at	*GAS7*	transmembrane protein 117	0.764	0.0077
213229_at	*CALD1*	dicer 1, ribonuclease III	0.756	0.0077
224252_s_at	*SH3D19*	FXYD domain containing ion transport regulator 5	0.912	0.0078
219563_at	*ZFP82*	spectrin repeat containing nuclear envelope family member 3///long intergenic non-protein coding RNA 341	0.574	0.0078
224909_s_at	*TTPAL*	phosphatidylinositol-3,4,5-trisphosphate dependent Rac exchange factor 1	0.979	0.0079

213103_at	*C21orf91*	StAR related lipid transfer domain containing 13	0.595	0.0080
224733_at	*NFKBID*	CKLF like MARVEL transmembrane domain containing 3	0.684	0.0080
208850_s_at	*TXNDC9*	Thy-1 cell surface antigen	0.901	0.0080
211986_at	*AGR2*	AHNAK nucleoprotein	0.902	0.0081
201040_at	*EXOSC3*	G protein subunit alpha i2	0.501	0.0082
228461_at	*GRP*	SH3 domain containing ring finger 3	0.785	0.0083
205116_at	*HIST1H2BK*	laminin subunit alpha 2	0.656	0.0084
212822_at	*CDKL3*	heart development protein with EGF like domains 1	0.589	0.0084
214581_x_at	*NFYC*	TNF receptor superfamily member 21	0.771	0.0084
207704_s_at	*LRSAM1*	growth arrest specific 7	0.447	0.0084
201616_s_at	*SYK*	caldesmon 1	1.046	0.0084
225162_at	*SEPT7P2*	SH3 domain containing 19	0.833	0.0085
200661_at	*FAM174B*	cathepsin A	0.606	0.0086
226368_at	*FAP*	carbohydrate (chondroitin 4) sulfotransferase 11	0.643	0.0086
219633_at	*FRZB*	alpha tocopherol transfer protein like	0.326	0.0088
220941_s_at	*LINC00304*	chromosome 21 open reading frame 91	0.462	0.0089
1553042_a_at	*LOC728485*	NFKB inhibitor delta	0.255	0.0090
215076_s_at	*ADAM21*	collagen type III alpha 1 chain	1.220	0.0090
205168_at	*MEGF6*	discoidin domain receptor tyrosine kinase 2	0.671	0.0090
209356_x_at	*TSPOAP1-AS1*	EGF containing fibulin like extracellular matrix protein 2	0.757	0.0091
229691_at	*NOTCH2NL*	zinc finger and BTB domain containing 42	0.591	0.0091
208945_s_at	*UBXN2B*	beclin 1	0.379	0.0091
202208_s_at	*CFLAR*	ADP ribosylation factor like GTPase 4C	0.685	0.0091
206326_at	*GSAP*	gastrin releasing peptide	1.189	0.0093
214752_x_at	*NFATC2*	filamin A	0.503	0.0093
209806_at	*ABCB1*	histone cluster 1, H2bk	1.382	0.0093
227675_at	*TMF1*	leucine rich repeat and sterile alpha motif containing 1	0.400	0.0094
226068_at	*POSTN*	spleen associated tyrosine kinase	0.961	0.0094
212937_s_at	*SETMAR*	collagen type VI alpha 1 chain	0.570	0.0094
209955_s_at	*BLK*	fibroblast activation protein alpha	1.004	0.0096
209999_x_at	*ZKSCAN3*	suppressor of cytokine signaling 1	0.476	0.0096
244023_at	*PRICKLE1*	spleen associated tyrosine kinase	0.722	0.0097
203698_s_at	*XG*	frizzled-related protein	0.793	0.0097
1553449_at	*CNTN2*	long intergenic non-protein coding RNA 304	0.335	0.0097
209348_s_at	*TRABD2A*	MAF bZIP transcription factor	0.843	0.0097
229584_at	*LOC101927792*	leucine rich repeat kinase 2	0.990	0.0098
213942_at	*AKAP13*	multiple EGF like domains 6	0.611	0.0098
235821_at	*RARRES2*	WNT1 inducible signaling pathway protein 1	0.848	0.0099
221729_at	*CERCAM*	collagen type V alpha 2 chain	1.264	0.0099
218856_at	*KHNYN*	TNF receptor superfamily member 21	0.774	0.0099

228826_at	*TRIM56*	TSPOAP1 antisense RNA 1	0.658	0.0099
214722_at	*NECTIN2*	notch 2 N-terminal like	0.726	0.0100
239629_at	*CYP26B1*	CASP8 and FADD like apoptosis regulator	0.770	0.0102
226210_s_at	*TMEM151B*	maternally expressed 3 (non-protein coding)	1.283	0.0102
213142_x_at	*IL1B*	gamma-secretase activating protein	1.051	0.0103
228442_at	*EPHA1-AS1*	nuclear factor of activated T-cells 2	0.922	0.0103
212288_at	*GTPBP8*	formin binding protein 1	1.000	0.0103
209993_at	*OR1E1*	ATP binding cassette subfamily B member 1	0.336	0.0103
203185_at	*BTN3A3*	Ras association domain family member 2	1.042	0.0104
228394_at	*SNX29*	serine/threonine kinase 10	0.433	0.0104
215855_s_at	*HIC1*	TATA element modulatory factor 1	0.391	0.0104
1555778_a_at	*LOC101928269///LOC100506403///RUNX1*	periostin	1.643	0.0105
214440_at	*CHAC1*	N-acetyltransferase 1	0.775	0.0106
207788_s_at	*IFNGR2*	sorbin and SH3 domain containing 3	0.451	0.0106
225604_s_at	*NBL1*	GLI pathogenesis related 2	0.790	0.0107
206255_at	*ZNF582-AS1*	BLK proto-oncogene, Src family tyrosine kinase	0.389	0.0107
1568957_x_at	*KIAA1257*	SLIT-ROBO Rho GTPase activating protein 2C///SLIT-ROBO Rho GTPase activating protein 2B///SLIT-ROBO Rho GTPase activating protein 2	0.323	0.0109
226065_at	*FGF5*	prickle planar cell polarity protein 1	0.798	0.0109
1554062_at	*SULF2*	Xg blood group	0.908	0.0109
227867_at	*PDPN*	TraB domain containing 2A	0.494	0.0110
200859_x_at	*UPB1*	filamin A	0.492	0.0110
206980_s_at	*C16orf45*	fms related tyrosine kinase 3 ligand	0.392	0.0110
221718_s_at	*COMP*	A-kinase anchoring protein 13	0.819	0.0111
235570_at	*MED15*	RNA binding motif single stranded interacting protein 3	1.086	0.0111
209496_at	*TSPAN12*	retinoic acid receptor responder 2	1.173	0.0111
224794_s_at	*YWHAZ*	cerebral endothelial cell adhesion molecule	0.605	0.0111
212356_at	*RUNX3*	KH and NYN domain containing	0.388	0.0112
226040_at	*TRAC///TRAJ17///TRAV20///TRDV2*	tripartite motif containing 56	0.651	0.0112
232078_at	*NMNAT3*	nectin cell adhesion molecule 2	0.366	0.0112
213932_x_at	*PCBD2*	major histocompatibility complex, class I, A	0.503	0.0113
225790_at	*BTRC*	methionine sulfoxide reductase B3	0.448	0.0113
219825_at	*C8orf48*	cytochrome P450 family 26 subfamily B member 1	1.084	0.0113
210511_s_at	*CEP89*	inhibin beta A subunit	1.117	0.0114
222689_at	*TPSB2*	alkaline ceramidase 3	0.500	0.0114
204780_s_at	*IL1R2*	Fas cell surface death receptor	0.951	0.0115
38241_at	*COL1A2*	butyrophilin subfamily 3 member A3	0.776	0.0116
225624_at	*FMNL3*	sorting nexin 29	0.513	0.0116
230218_at	*HEPH*	hypermethylated in cancer 1	0.452	0.0116

209360_s_at	*KCNC4*	uncharacterized LOC101928269///uncharacterized LOC100506403///runt related transcription factor 1	0.909	0.0117
201642_at	*LINGO1-AS1*	interferon gamma receptor 2 (interferon gamma transducer 1)	0.385	0.0119
201621_at	*STXBP5L*	neuroblastoma 1, DAN family BMP antagonist	0.746	0.0119
205582_s_at	*ISG15*	gamma-glutamyltransferase 5	0.640	0.0120
1555705_a_at	*OR2L2*	CKLF like MARVEL transmembrane domain containing 3	0.597	0.0120
224724_at	*ITGA8*	sulfatase 2	0.984	0.0121
213071_at	*RMND5B*	dermatopontin	0.932	0.0122
221898_at	*COL11A1*	podoplanin	0.958	0.0122
213746_s_at	*FLJ38576*	filamin A	0.576	0.0122
224043_s_at	*CHRNA4*	beta-ureidopropionase 1	0.208	0.0122
221730_at	*WLS*	collagen type V alpha 2 chain	1.446	0.0123
212736_at	*C7*	chromosome 16 open reading frame 45	0.806	0.0123
205713_s_at	*FARP1*	cartilage oligomeric matrix protein	1.235	0.0124
205422_s_at	*EAF2*	integrin subunit beta like 1	0.654	0.0125
222175_s_at	*LOC100996412*	mediator complex subunit 15	0.292	0.0125
218084_x_at	*BTN2A1*	FXYD domain containing ion transport regulator 5	0.895	0.0126
214848_at	*DZIP1*	tyrosine 3-monooxygenase/tryptophan 5-monooxygenase activation protein zeta	0.319	0.0130
204198_s_at	*CADPS*	runt related transcription factor 3	1.190	0.0130
210972_x_at	*CEBPA*	T-cell receptor alpha constant///T cell receptor alpha joining 17///T cell receptor alpha variable 20///T cell receptor delta variable 2	0.941	0.0130
204821_at	*COTL1*	butyrophilin subfamily 3 member A3	0.732	0.0130
207134_x_at	*HIPK2*	tryptase beta 2 (gene/pseudogene)	1.143	0.0132
228585_at	*RAB3GAP1*	ectonucleoside triphosphate diphosphohydrolase 1	0.416	0.0132
227995_at	*SIKE1*	plexin domain containing 2	0.524	0.0132
209869_at	*NFATC1*	adrenoceptor alpha 2A	0.803	0.0133
205403_at	*AIFM2*	interleukin 1 receptor type 2	0.939	0.0133
242676_at	*VASN*	NDUFV2 antisense RNA 1	0.260	0.0134
203697_at	*ASB13*	frizzled-related protein	0.803	0.0134
202403_s_at	*TUBE1*	collagen type I alpha 2 chain	1.017	0.0135
230640_at	*NOTCH2*	formin like 3	0.710	0.0135
210904_s_at	*MIR612///NEAT1*	interleukin 13 receptor subunit alpha 1	0.579	0.0135
203903_s_at	*TTC33*	hephaestin	0.976	0.0135
210190_at	*ZNF790-AS1*	syntaxin 11	0.331	0.0136
227566_at	*F2R*	neurotrimin-like///neurotrimin	1.149	0.0136
205381_at	*ZNF404*	leucine rich repeat containing 17	0.813	0.0138
205483_s_at	*RNASEH2B*	ISG15 ubiquitin-like modifier	1.051	0.0138
222688_at	*TMC5*	alkaline ceramidase 3	0.788	0.0138
208324_at	*CRISP3*	A-kinase anchoring protein 13	0.211	0.0138

206114_at	*SMAD4*	EPH receptor A4	1.081	0.0139
214265_at	*TSPAN14*	integrin subunit alpha 8	0.511	0.0139
204320_at	*STAG3L2///STAG3L3///TRIM73*	collagen type XI alpha 1 chain	1.420	0.0140
221942_s_at	*MGC4859*	guanylate cyclase 1 soluble subunit alpha	1.006	0.0140
221958_s_at	*ZFHX3*	wntless Wnt ligand secretion mediator	0.828	0.0141
203906_at	*KIAA1841*	IQ motif and Sec7 domain 1	0.393	0.0142
210510_s_at	*TFAP2A-AS1*	neuropilin 1	0.582	0.0142
201910_at	*GRIK1*	FERM, ARH/RhoGEF and pleckstrin domain protein 1	0.612	0.0142
230292_at	*LOC101928461*	uncharacterized LOC100996412	0.580	0.0143
202192_s_at	*XAF1*	growth arrest specific 7	0.845	0.0143
203944_x_at	*RUSC1-AS1*	butyrophilin subfamily 2 member A1	0.471	0.0143
204557_s_at	*NUP210L*	DAZ interacting zinc finger protein 1	0.950	0.0144
239884_at	*PTPN11*	calcium dependent secretion activator	1.003	0.0145
204039_at	*TAS2R39*	CCAAT/enhancer binding protein alpha	0.829	0.0145
221059_s_at	*LINC00664*	coactosin like F-actin binding protein 1	0.899	0.0145
211161_s_at	*CD7*	collagen type III alpha 1 chain	1.495	0.0146
37892_at	*MCOLN2*	collagen type XI alpha 1 chain	1.580	0.0147
202311_s_at	*CEP290*	collagen type I alpha 1 chain	1.651	0.0147
1554795_a_at	*APOE*	filamin binding LIM protein 1	0.558	0.0148
212796_s_at	*NLRC5*	TBC1 domain family member 2B	0.439	0.0148
213531_s_at	*MOXD1*	RAB3 GTPase activating protein catalytic subunit 1	0.233	0.0148
211105_s_at	*SIRT5*	nuclear factor of activated T-cells 1	0.856	0.0149
230031_at	*ZNF562*	heat shock protein family A (Hsp70) member 5	0.401	0.0149
206999_at	*RIN3*	interleukin 12 receptor subunit beta 2	0.397	0.0149
224461_s_at	*LTBP2*	apoptosis inducing factor, mitochondria associated 2	0.418	0.0150
225867_at	*ADAM28*	vasorin	0.577	0.0150
218862_at	*KIF6*	ankyrin repeat and SOCS box containing 13	0.653	0.0150
202310_s_at	*AP1S2*	collagen type I alpha 1 chain	1.276	0.0151
212377_s_at	*BOD1*	notch 2	0.714	0.0152
220983_s_at	*ZBTB38*	microRNA 612///nuclear paraspeckle assembly transcript 1 (non-protein coding)	0.223	0.0152
230867_at	*DNAJC19*	collagen type VI alpha 6 chain	1.018	0.0153
203989_x_at	*LINC01279*	coagulation factor II thrombin receptor	1.108	0.0153
229210_at	*ZNF358*	ribonuclease H2 subunit B	0.327	0.0155
201852_x_at	*PRR15*	collagen type III alpha 1 chain	1.394	0.0155
221002_s_at	*LIMK1*	tetraspanin 14	0.364	0.0156
233555_s_at	*SOX15*	sulfatase 2	0.734	0.0157
204036_at	*FHOD1*	lysophosphatidic acid receptor 1	1.106	0.0157
1554250_s_at	*KIAA1551*	stromal antigen 3-like 2 (pseudogene)///stromal antigen 3-like 3 (pseudogene)///tripartite motif containing 73	0.466	0.0158

204556_s_at	*LRP1*	DAZ interacting zinc finger protein 1	0.559	0.0159
242738_s_at	*ATG101*	zinc finger homeobox 3	0.583	0.0160
221815_at	*NOX4*	abhydrolase domain containing 2	0.459	0.0160
208886_at	*GPR68*	H1 histone family member 0	0.548	0.0162
206133_at	*ANXA2*	XIAP associated factor 1	0.844	0.0162
214551_s_at	*ZNF233*	CD7 molecule	0.690	0.0167
211822_s_at	*DNAH6*	NLR family pyrin domain containing 1	0.517	0.0167
207540_s_at	*ATP1A1-AS1*	spleen associated tyrosine kinase	0.839	0.0168
213728_at	*LINC00623*	lysosomal associated membrane protein 1	0.347	0.0168
230110_at	*KDSR*	mucolipin 2	0.962	0.0170
203382_s_at	*ANGPT2*	apolipoprotein E	1.002	0.0170
225602_at	*GOLGA3*	GLI pathogenesis related 2	0.763	0.0170
226474_at	*NUCB1*	NLR family CARD domain containing 5	1.112	0.0171
205574_x_at	*ACTN3*	bone morphogenetic protein 1	0.500	0.0171
1554474_a_at	*SULF1*	monooxygenase DBH like 1	0.727	0.0172
217718_s_at	*FAM181B*	tyrosine 3-monooxygenase/tryptophan 5-monooxygenase activation protein beta	0.248	0.0172
63825_at	*SNRPC*	abhydrolase domain containing 2	0.445	0.0174
204682_at	*ZNF383*	latent transforming growth factor beta binding protein 2	0.749	0.0174
208268_at	*XCL1*	ADAM metallopeptidase domain 28	0.270	0.0174
224583_at	*MAP3K15*	coactosin like F-actin binding protein 1	1.077	0.0175
204527_at	*LOC100289058*	myosin VA	0.608	0.0176
227610_at	*FAM27B///FAM27C*	tetraspanin 11	0.615	0.0176
201124_at	*TRAF3*	integrin subunit beta 5	0.844	0.0176
227061_at	*LOC101928812*	long intergenic non-protein coding RNA 1279	1.245	0.0178
204184_s_at	*MRGPRF*	G protein-coupled receptor kinase 3	0.435	0.0179
229802_at	*COA1*	WNT1 inducible signaling pathway protein 1	1.312	0.0179
211566_x_at	*FBXL4*	brain and reproductive organ-expressed (TNFRSF1A modulator)	0.291	0.0180
224925_at	*ABCC1*	phosphatidylinositol-3,4,5-trisphosphate dependent Rac exchange factor 1	0.919	0.0180
204357_s_at	*WDR6*	LIM domain kinase 1	0.373	0.0182
1554544_a_at	*ANXA2P2*	myelin basic protein	0.570	0.0182
218530_at	*TMCC1-AS1*	formin homology 2 domain containing 1	0.509	0.0183
227152_at	*USP13*	KIAA1551	1.209	0.0183
212998_x_at	*ABHD13*	HLA class II histocompatibility antigen, DQ beta 1 chain-like///major histocompatibility complex, class II, DQ beta 1	1.482	0.0183
36920_at	*FCER1A*	myotubularin 1	0.262	0.0184
200784_s_at	*FMOD*	LDL receptor related protein 1	0.450	0.0184
229530_at	*SPON1*	guanylate cyclase 1 soluble subunit alpha	0.836	0.0184
221541_at	*ADCY7*	cysteine rich secretory protein LCCL domain containing 2	0.854	0.0185
209664_x_at	*ITGA4*	nuclear factor of activated T-cells 1	0.281	0.0185

219773_at	*RASGRP2*	NADPH oxidase 4	0.731	0.0185
225442_at	*ALKBH1*	discoidin domain receptor tyrosine kinase 2	0.820	0.0186
200625_s_at	*MYCBPAP*	adenylate cyclase associated protein 1	0.424	0.0187
221276_s_at	*KIAA0930*	syncoilin, intermediate filament protein	0.531	0.0189
229055_at	*LINC00461///MIR9-2*	G protein-coupled receptor 68	0.612	0.0189
226421_at	*ELF1*	Alport syndrome, mental retardation, midface hypoplasia and elliptocytosis chromosomal region gene 1	0.708	0.0189
201590_x_at	*LGALS3*	annexin A2	0.857	0.0189
1555033_a_at	*C1S*	regulator of G-protein signaling 12	0.225	0.0190
201141_at	*ZNF253*	glycoprotein nmb	1.180	0.0190
206682_at	*PARP14*	C-type lectin domain family 10 member A	0.841	0.0190
235643_at	*LOC101929549*	sterile alpha motif domain containing 9 like	0.659	0.0191
204735_at	*ST3GAL1*	phosphodiesterase 4A	0.729	0.0192
226372_at	*CYB5D1*	carbohydrate (chondroitin 4) sulfotransferase 11	0.634	0.0193
229429_x_at	*EIF4G2*	long intergenic non-protein coding RNA 623	0.592	0.0194
203047_at	*PARVB*	serine/threonine kinase 10	0.561	0.0194
201125_s_at	*KRTDAP*	integrin subunit beta 5	0.816	0.0195
227607_at	*LAMB2*	STAM binding protein like 1	0.978	0.0195
202419_at	*HAS2*	3-ketodihydrosphingosine reductase	0.369	0.0196
228152_s_at	*FANCI*	DEAD-box helicase 60-like	0.695	0.0196
205572_at	*WWC3*	angiopoietin 2	0.570	0.0196
202106_at	*ZNF844*	golgin A3	0.230	0.0197
200649_at	*RBMS2*	nucleobindin 1	0.365	0.0197
212353_at	*WFDC1*	sulfatase 1	0.900	0.0198
206932_at	*ZNF230*	cholesterol 25-hydroxylase	0.959	0.0199
201888_s_at	*CDC34*	interleukin 13 receptor subunit alpha 1	0.732	0.0199
225558_at	*TMEM139*	GIT ArfGAP 2	0.496	0.0200
206366_x_at	*MIRLET7D*	X-C motif chemokine ligand 1	1.177	0.0201
1563077_at	*NFAT5*	uncharacterized LOC100289058	0.374	0.0204
207043_s_at	*KIAA1210*	solute carrier family 6 member 9	0.302	0.0204
221571_at	*GIGYF1*	TNF receptor associated factor 3	0.494	0.0205
232187_at	*SMPD3*	palmdelphin	0.324	0.0205
227727_at	*CAPZB*	MAS related GPR family member F	0.768	0.0207
232330_at	*KIAA1147*	cytochrome c oxidase assembly factor 1 homolog	0.388	0.0208
202804_at	*LOC105372404*	ATP binding cassette subfamily C member 1	0.662	0.0208
209221_s_at	*C5orf63*	oxysterol binding protein like 2	0.421	0.0208
241398_at	*LOC100508408///SNORD14B///RPS13*	metallophosphoesterase domain containing 1	0.621	0.0208
233110_s_at	*PLA2R1*	BCL2 like 12	0.286	0.0209
231365_at	*FAM20C*	HOXA10 antisense RNA///microRNA 196b	0.374	0.0209
233573_s_at	*POT1-AS1*	WD repeat domain 6	0.272	0.0209

208816_x_at	*SLFN5*	annexin A2 pseudogene 2	0.769	0.0209
217992_s_at	*UVSSA*	EF-hand domain family member D2	0.589	0.0210
234993_at	*MGEA5*	abhydrolase domain containing 13	0.385	0.0211
211734_s_at	*NBPF10///NBPF8///NBPF11*	Fc fragment of IgE receptor Ia	0.832	0.0212
202709_at	*PTGDR*	fibromodulin	0.824	0.0212
210427_x_at	*HID1*	annexin A2	0.845	0.0212
209436_at	*RAB7B*	spondin 1	0.990	0.0212
203741_s_at	*IL1RAPL2*	adenylate cyclase 7	0.859	0.0212
213416_at	*ATP2A3*	integrin subunit alpha 4	0.892	0.0213
208206_s_at	*TMEM183B///TMEM183A*	RAS guanyl releasing protein 2	0.587	0.0213
213010_at	*RUBCN*	protein kinase C delta binding protein	0.767	0.0213
215313_x_at	*DKK2*	major histocompatibility complex, class I, A	0.589	0.0213
230252_at	*DOK4*	lysophosphatidic acid receptor 5	0.598	0.0214
227144_at	*LCA5*	KIAA0930	0.547	0.0214
209534_x_at	*UQCRQ*	A-kinase anchoring protein 13	0.587	0.0215
202273_at	*LOC149684///BPI*	platelet derived growth factor receptor beta	0.862	0.0215
212420_at	*COPZ2*	E74 like ETS transcription factor 1	0.852	0.0215
238455_at	*GRB10*	plexin domain containing 2	0.443	0.0215
208949_s_at	*PAPLN*	lectin, galactoside binding soluble 3	0.939	0.0216
208747_s_at	*ZDHHC20*	complement component 1, s subcomponent	1.432	0.0216
224701_at	*CLEC2B*	poly(ADP-ribose) polymerase family member 14	0.884	0.0217
226632_at	*ANGEL2*	cytoglobin	0.667	0.0218
203476_at	*C1QTNF6*	trophoblast glycoprotein	0.948	0.0218
208322_s_at	*CHM*	ST3 beta-galactoside alpha-2,3-sialyltransferase 1	0.458	0.0218
1555358_a_at	*LARS*	ectonucleoside triphosphate diphosphohydrolase 4	0.296	0.0218
227039_at	*IFIT5*	A-kinase anchoring protein 13	0.676	0.0219
211864_s_at	*AQP1*	myoferlin	0.981	0.0219
204253_s_at	*TDRG1*	vitamin D (1,25- dihydroxyvitamin D3) receptor	0.294	0.0219
229822_at	*ECM2*	parvin beta	0.486	0.0220
230835_at	*GRID1*	keratinocyte differentiation associated protein	2.675	0.0220
235834_at	*PPFIA1*	caldesmon 1	0.540	0.0220
216264_s_at	*UNC13D*	laminin subunit beta 2	0.756	0.0221
230372_at	*BTN3A2///BTN3A3*	hyaluronan synthase 2	0.942	0.0221
205991_s_at	*LOC339666*	paired related homeobox 1	0.709	0.0221
225273_at	*LOC101929181*	WWC family member 3	0.720	0.0222
225776_at	*TAP2*	RNA binding motif single stranded interacting protein 2	0.563	0.0223
219478_at	*NLRP2*	WAP four-disulfide core domain 1	0.744	0.0225
52837_at	*NDUFA3*	KIAA1644	0.494	0.0225
226837_at	*MTHFR*	sprouty related EVH1 domain containing 1	0.963	0.0226

212940_at	*LINC00471*	collagen type VI alpha 1 chain	0.430	0.0226
202278_s_at	*MYOD1*	serine palmitoyltransferase long chain base subunit 1	0.360	0.0227
205842_s_at	*PARP12*	Janus kinase 2	0.492	0.0227
203381_s_at	*SERPINB13*	apolipoprotein E	0.959	0.0227
1552264_a_at	*BNC2*	mitogen-activated protein kinase 1	0.331	0.0229
217404_s_at	*FMO3*	collagen type II alpha 1 chain	0.265	0.0229
232090_at	*PCDH18*	DNM3 opposite strand/antisense RNA	0.886	0.0229
217525_at	*PPP1R9B*	olfactomedin like 1	0.739	0.0229
214753_at	*LOC101928865*	NEDD4 binding protein 2 like 2	0.665	0.0229
215092_s_at	*ADIPOQ*	nuclear factor of activated T-cells 5	0.694	0.0229
220002_at	*NLN*	kinesin family member 26B	0.298	0.0230
233634_at	*CCDC168*	MARVEL domain containing 3	0.408	0.0230
228755_at	*EEF1AKMT1*	GRB10 interacting GYF protein 1	0.449	0.0230
212999_x_at	*MTDH*	HLA class II histocompatibility antigen, DQ beta 1 chain-like///major histocompatibility complex, class II, DQ beta 1	0.864	0.0231
218076_s_at	*LOC100505710*	Rho GTPase activating protein 17	0.330	0.0231
238699_s_at	*ITPRIPL2*	calcium/calmodulin dependent serine protein kinase	0.212	0.0231
232517_s_at	*ASAH1*	helicase with zinc finger 2	0.299	0.0232
200787_s_at	*MSL3*	phosphoprotein enriched in astrocytes 15	0.567	0.0232
231732_at	*COL5A1*	sphingomyelin phosphodiesterase 3	0.306	0.0232
201949_x_at	*N4BP1*	capping actin protein of muscle Z-line beta subunit	0.292	0.0233
227502_at	*NPL*	KIAA1147	0.336	0.0233
213069_at	*DCAF8*	heart development protein with EGF like domains 1	0.687	0.0235
229327_s_at	*CHD7*	MAF bZIP transcription factor	0.662	0.0235
217118_s_at	*SMAD6*	KIAA0930	0.659	0.0235
238761_at	*FOXN3*	ELK4, ETS transcription factor	0.576	0.0236
226380_at	*PPP2R5C*	protein tyrosine phosphatase, non-receptor type 21	0.590	0.0239
230383_x_at	*ZNF93*	schlafen family member 5	0.659	0.0239
233893_s_at	*TTLL11*	UV stimulated scaffold protein A	0.563	0.0240
238852_at	*IHH*	paired related homeobox 1	0.778	0.0240
1556607_at	*TNKS2*	EH domain containing 4	0.204	0.0241
217226_s_at	*TRPC6*	sideroflexin 3	0.596	0.0241
223494_at	*RFX2*	meningioma expressed antigen 5 (hyaluronidase)	0.523	0.0242
229447_x_at	*POLDIP3*	neuroblastoma breakpoint family member 10///neuroblastoma breakpoint family member 8///neuroblastoma breakpoint family member 11	0.520	0.0242
215894_at	*ZNF226*	prostaglandin D2 receptor	0.499	0.0243
230266_at	*RAB2A*	RAB7B, member RAS oncogene family	0.765	0.0244
207522_s_at	*RUNX2*	ATPase sarcoplasmic/endoplasmic reticulum Ca2+ transporting 3	0.859	0.0245

219908_at	*ZNF654*	dickkopf WNT signaling pathway inhibitor 2	0.460	0.0246
235449_at	*EFNB2*	leucine rich repeat and sterile alpha motif containing 1	0.225	0.0247
228213_at	*TEX2*	H2A histone family member J	0.483	0.0247
209691_s_at	*CYB5B*	docking protein 4	0.674	0.0247
227810_at	*SEPT4*	zinc finger protein 558	0.598	0.0247
1558028_x_at	*DNM1*	non-coding RNA activated by DNA damage	0.525	0.0248
219561_at	*NUTM2B-AS1*	coatomer protein complex subunit zeta 2	0.688	0.0250
215248_at	*HOXB6*	growth factor receptor bound protein 10	0.528	0.0250
226435_at	*LOC400043*	papilin, proteoglycan like sulfated glycoprotein	0.863	0.0251
225365_at	*GATA2*	zinc finger DHHC-type containing 20	0.386	0.0251
209335_at	*SERPINF1*	decorin	1.201	0.0252
209732_at	*PRKCA*	C-type lectin domain family 2 member B	1.163	0.0252
206547_s_at	*POMC*	protein phosphatase with EF-hand domain 1	0.388	0.0252
200872_at	*MZT2A///MZT2B///PHGDH*	S100 calcium binding protein A10	1.019	0.0252
223571_at	*RAD51D*	C1q and tumor necrosis factor related protein 6	0.626	0.0252
207099_s_at	*LOC101929977*	CHM, Rab escort protein 1	0.229	0.0252
212285_s_at	*HOOK3*	agrin	0.467	0.0252
203596_s_at	*CLDND2*	interferon induced protein with tetratricopeptide repeats 5	0.444	0.0253
53720_at	*CLCN1*	chromosome 19 open reading frame 66	0.613	0.0253
207542_s_at	*MICALL2*	aquaporin 1 (Colton blood group)	0.572	0.0253
209846_s_at	*NIFK-AS1*	butyrophilin subfamily 3 member A2	0.981	0.0254
206101_at	*LMO3*	extracellular matrix protein 2	0.830	0.0255
210235_s_at	*CBX6*	PTPRF interacting protein alpha 1	0.383	0.0255
228352_at	*P2RX7*	unc-13 homolog D	0.265	0.0255
204820_s_at	*NACA*	butyrophilin subfamily 3 member A2///butyrophilin subfamily 3 member A3	1.023	0.0255
208790_s_at	*TRPM3*	polymerase I and transcript release factor	0.698	0.0257
1568955_at	*LPP*	SLIT-ROBO Rho GTPase activating protein 2C///SLIT-ROBO Rho GTPase activating protein 2B///SLIT-ROBO Rho GTPase activating protein 2	0.293	0.0257
226944_at	*OR5V1*	HtrA serine peptidase 3	0.466	0.0258
225973_at	*SEC61A2*	transporter 2, ATP binding cassette subfamily B member	0.979	0.0258
235321_at	*URM1*	NADH:ubiquinone oxidoreductase core subunit S1	0.553	0.0258
221690_s_at	*FNBP1L*	NLR family pyrin domain containing 2	0.916	0.0259
231876_at	*ARRDC2*	tripartite motif containing 56	0.473	0.0260
226929_at	*NLRP7*	methylenetetrahydrofolate reductase (NAD(P)H)	0.398	0.0261
204014_at	*EIF1AD*	dual specificity phosphatase 4	0.927	0.0262
211084_x_at	*PPARA*	protein kinase D3	0.387	0.0262

214022_s_at	*PIN4*	interferon induced transmembrane protein 1	1.012	0.0263
218543_s_at	*MTF1*	poly(ADP-ribose) polymerase family member 12	0.766	0.0263
222986_s_at	*EPSTI1*	shisa family member 5	0.454	0.0264
1559249_at	*CYP2E1*	ataxin 1	0.499	0.0265
238478_at	*NT5DC2*	basonuclin 2	0.650	0.0265
40665_at	*MDFIC*	flavin containing monooxygenase 3	0.913	0.0265
225977_at	*LOC100289090*	protocadherin 18	0.208	0.0265
225124_at	*MTBP*	protein phosphatase 1 regulatory subunit 9B	0.481	0.0266
201615_x_at	*MIR3610///RAD21-AS1*	caldesmon 1	0.940	0.0266
206649_s_at	*HMGN3*	transcription factor binding to IGHM enhancer 3	0.351	0.0267
227054_at	*ARID3A*	eukaryotic translation elongation factor 1 alpha lysine methyltransferase 1	0.499	0.0268
227277_at	*LOC100505715*	metadherin	0.370	0.0268
227514_at	*KLF8*	inositol 1,4,5-trisphosphate receptor interacting protein like 2	0.726	0.0268
213902_at	*CLUL1*	N-acylsphingosine amidohydrolase 1	0.381	0.0269
238905_at	*CELF3*	ras homolog family member J	0.414	0.0269
207551_s_at	*MCM3AP*	male-specific lethal 3 homolog (Drosophila)	0.369	0.0269
212488_at	*PSMD6-AS2*	collagen type V alpha 1 chain	1.249	0.0270
221867_at	*CIITA*	NEDD4 binding protein 1	0.403	0.0272
240440_at	*PCIF1*	N-acetylneuraminate pyruvate lyase	0.389	0.0272
202944_at	*GJD3*	alpha-N-acetylgalactosaminidase	0.481	0.0273
221447_s_at	*PCGF5*	glycosyltransferase 8 domain containing 2	0.662	0.0273
207069_s_at	*DYNC1H1*	SMAD family member 6	0.425	0.0274
200646_s_at	*BTBD9*	nucleobindin 1	0.411	0.0275
218031_s_at	*TNFAIP6*	forkhead box N3	0.603	0.0275
224611_s_at	*ATP9B*	DnaJ heat shock protein family (Hsp40) member C5	0.351	0.0275
221024_s_at	*HHEX*	solute carrier family 2 member 10	0.828	0.0276
204163_at	*RELL1*	elastin microfibril interfacer 1	0.673	0.0277
1557718_at	*UMPS*	protein phosphatase 2 regulatory subunit B'gamma	0.490	0.0277
204037_at	*DCAF13*	lysophosphatidic acid receptor 1	0.576	0.0278
215420_at	*LOC101927210*	indian hedgehog	0.238	0.0278
222562_s_at	*OPRL1*	tankyrase 2	0.663	0.0278
217287_s_at	*YME1L1*	transient receptor potential cation channel subfamily C member 6	0.233	0.0278
235240_at	*CLMP*	ataxin 3	0.460	0.0279
205201_at	*ISLR*	GLI family zinc finger 3	0.506	0.0279
214021_x_at	*CST7*	integrin subunit beta 5	0.837	0.0280
201553_s_at	*FXYD2*	lysosomal associated membrane protein 1	0.335	0.0280
226872_at	*SLC19A1*	regulatory factor X2	0.614	0.0280

208733_at	*RDH11*	RAB2A, member RAS oncogene family	0.378	0.0282
232231_at	*DKK3*	runt related transcription factor 2	1.207	0.0283
204502_at	*RAB22A*	SAM and HD domain containing deoxynucleoside triphosphate triphosphohydrolase 1	0.688	0.0283
1556744_a_at	*RCN3*	zinc finger protein 654	0.538	0.0283
227552_at	*ACRBP*	septin 1	0.578	0.0284
202669_s_at	*ZBTB1*	ephrin B2	0.840	0.0284
227382_at	*LIN28B*	cytochrome b5 type B	0.319	0.0285
222150_s_at	*IFI44*	gamma-secretase activating protein	0.827	0.0286
226865_at	*SRGN*	plexin domain containing 2	0.801	0.0286
210657_s_at	*HNRNPM*	septin 4	0.507	0.0286
202728_s_at	*LINC00301*	latent transforming growth factor beta binding protein 1	0.645	0.0286
215116_s_at	*LOC102724200 ///TRAPPC10*	dynamin 1	0.696	0.0286
206624_at	*TULP4*	ubiquitin specific peptidase 9, Y-linked	0.969	0.0286
235730_at	*LRRK1*	NUTM2B antisense RNA 1	0.531	0.0286
205366_s_at	*LARP1*	homeobox B6	0.202	0.0286
226582_at	*RNF166*	uncharacterized LOC400043	0.547	0.0287
202283_at	*LOC440117*	serpin family F member 1	1.221	0.0288
217312_s_at	*TMEM173*	collagen type VII alpha 1 chain	0.777	0.0288
205720_at	*HIVEP2*	proopiomelanocortin	0.344	0.0288
228617_at	*CSTA*	XIAP associated factor 1	0.976	0.0289
201617_x_at	*KIAA1468*	caldesmon 1	0.962	0.0289
1556209_at	*DARS2*	C-type lectin domain family 2 member B	0.522	0.0291
202856_s_at	*VWA5A*	microRNA 6787///solute carrier family 16 member 3	0.738	0.0291
211748_x_at	*SH3KBP1*	prostaglandin D2 synthase	1.232	0.0292
224359_s_at	*PRR5L*	hook microtubule tethering protein 3	0.334	0.0292
231162_at	*PLIN2*	claudin domain containing 2	0.231	0.0293
203512_at	*CYP2R1*	trafficking protein particle complex 3	0.362	0.0294
204879_at	*BFSP1*	podoplanin	0.434	0.0294
221653_x_at	*UBA5*	apolipoprotein L2	0.559	0.0294
202048_s_at	*HERC6*	chromobox 6	0.354	0.0295
207091_at	*ZNF775*	purinergic receptor P2X 7	0.367	0.0295
202822_at	*VIM*	LIM domain containing preferred translocation partner in lipoma	0.587	0.0296
238430_x_at	*IGF1*	schlafen family member 5	0.707	0.0296
226055_at	*FAM187A///CC DC103*	arrestin domain containing 2	0.440	0.0298
225242_s_at	*HCG11*	coiled-coil domain containing 80	1.147	0.0298
229178_at	*RHOF*	protogenin	0.209	0.0299
244689_at	*PRADC1*	peroxisome proliferator activated receptor alpha	0.398	0.0299
232034_at	*GINS2*	long intergenic non-protein coding RNA 537	0.550	0.0300
1555469_a_at	*TEDDM1*	cytoplasmic linker associated protein 2	0.361	0.0300

231075_x_at	*GABPB1-AS1*	abl-interactor 2	0.357	0.0301
205323_s_at	*ANK3*	metal regulatory transcription factor 1	0.280	0.0301
235276_at	*CNTNAP2*	epithelial stromal interaction 1 (breast)	1.129	0.0302
209975_at	*HMGN3-AS1*	cytochrome P450 family 2 subfamily E member 1	0.504	0.0302
202439_s_at	*ARAP3*	iduronate 2-sulfatase	0.601	0.0303
1559942_at	*LOC100653005*	MyoD family inhibitor domain containing	0.352	0.0304
201893_x_at	*REC114*	decorin	1.058	0.0304
203878_s_at	*DOCK5*	matrix metallopeptidase 11	0.950	0.0305
213241_at	*EPDR1*	plexin C1	1.182	0.0305
220560_at	*UBE2F*	chromosome 11 open reading frame 21	0.440	0.0305
210809_s_at	*MAMSTR*	periostin	1.334	0.0306
228008_at	*WIPF2*	AT-rich interaction domain 3A	0.496	0.0306
230986_at	*HES4*	Kruppel like factor 8	0.482	0.0307
208372_s_at	*LINC00957*	LIM domain kinase 1	0.306	0.0310
212269_s_at	*ZNF548*	minichromosome maintenance complex component 3 associated protein	0.351	0.0310
232527_at	*TOB1-AS1*	PSMD6 antisense RNA 2	0.473	0.0310
204197_s_at	*ZBTB4*	runt related transcription factor 3	0.858	0.0310
208325_s_at	*TMEM25*	A-kinase anchoring protein 13	0.712	0.0310
217875_s_at	*IFITM3*	prostate transmembrane protein, androgen induced 1	0.761	0.0311
226997_at	*PYCARD*	ADAM metallopeptidase with thrombospondin type 1 motif 12	0.732	0.0311
203876_s_at	*TTLL13P*	matrix metallopeptidase 11	0.866	0.0311
228948_at	*LOC101930595 ///PGGHG*	EPH receptor A4	0.876	0.0311
205101_at	*LOC100130872 ///SPON2*	class II major histocompatibility complex transactivator	0.602	0.0311
212187_x_at	*FAM219B*	prostaglandin D2 synthase	1.267	0.0312
230025_at	*SYTL2*	gap junction protein delta 3	0.336	0.0313
207808_s_at	*KLF15*	protein S (alpha)	0.769	0.0313
226326_at	*PREX2*	polycomb group ring finger 5	0.452	0.0313
204463_s_at	*CST1*	endothelin receptor type A	0.646	0.0314
202047_s_at	*TRAPPC2L*	chromobox 6	0.317	0.0314
241084_x_at	*MINOS1-NBL1///NBL1*	dynein cytoplasmic 1 heavy chain 1	0.303	0.0314
203149_at	*ASMT*	nectin cell adhesion molecule 2	0.336	0.0315
209285_s_at	*PPP1R21*	family with sequence similarity 208 member A	0.570	0.0315
206025_s_at	*RIBC1*	TNF alpha induced protein 6	0.938	0.0316
237766_at	*LOC101928401*	ATPase phospholipid transporting 9B (putative)	0.299	0.0317
200904_at	*PANK2*	major histocompatibility complex, class I, E	0.668	0.0317
215933_s_at	*SLC25A35*	hematopoietically expressed homeobox	0.527	0.0318
211656_x_at	*DEF6*	HLA class II histocompatibility antigen, DQ beta 1 chain-like///major histocompatibility complex, class II, DQ beta 1	1.102	0.0318

227654_at	*TRBC1*	family with sequence similarity 65 member C	0.847	0.0318
220843_s_at	*WNT10B*	DDB1 and CUL4 associated factor 13	0.451	0.0319
211372_s_at	*MIR194-2*	interleukin 1 receptor type 2	0.437	0.0320
219011_at	*SIPA1*	pleckstrin homology domain containing A4	0.526	0.0320
211902_x_at	*KCNAB1*	YME1 like 1 ATPase	0.695	0.0321
226834_at	*TTN*	CXADR like membrane protein	0.897	0.0322
207191_s_at	*SLC39A13*	immunoglobulin superfamily containing leucine rich repeat	1.004	0.0322
226796_at	*RBMS1*	abhydrolase domain containing 15	0.422	0.0323
210140_at	*FHAD1*	cystatin F	1.005	0.0323
213068_at	*FAM222B*	dermatopontin	1.300	0.0324
201029_s_at	*IDUA*	CD99 molecule	0.679	0.0324
215336_at	*DGKA*	A-kinase anchoring protein 11	0.269	0.0327
221127_s_at	*ERICH1*	dickkopf WNT signaling pathway inhibitor 3	0.566	0.0327
213405_at	*VSTM2A*	RAB22A, member RAS oncogene family	0.385	0.0328
61734_at	*LOC101928045*	reticulocalbin 3	0.511	0.0329
223717_s_at	*HRH4*	acrosin binding protein	0.307	0.0329
1557036_at	*EDEM1*	zinc finger and BTB domain containing 1	0.417	0.0329
213546_at	*FUT6*	uncharacterized protein DKFZp586I1420	0.432	0.0330
214059_at	*RPUSD1*	interferon induced protein 44	0.776	0.0330
216971_s_at	*MYO7B*	plectin	0.436	0.0331
211067_s_at	*TAP1*	growth arrest specific 7	0.668	0.0332
229218_at	*RORA*	collagen type I alpha 2 chain	1.158	0.0333
223123_s_at	*AQP9*	PITH domain containing 1	0.338	0.0334
208184_s_at	*SNTG2*	trafficking protein particle complex subunit 10-like///trafficking protein particle complex 10	0.245	0.0335
224170_s_at	*ABCB4///ABCB1*	tubby like protein 4	0.336	0.0335
201887_at	*AEBP2*	interleukin 13 receptor subunit alpha 1	0.774	0.0335
202404_s_at	*MINA*	collagen type I alpha 2 chain	1.075	0.0335
219441_s_at	*ANGPTL1*	leucine rich repeat kinase 1	0.564	0.0336
227726_at	*OR10H3*	ring finger protein 166	0.437	0.0336
226069_at	*RNF213*	prickle planar cell polarity protein 1	0.724	0.0337
212566_at	*TBC1D16*	microtubule associated protein 4	0.444	0.0337
224929_at	*HLA-G*	transmembrane protein 173	0.627	0.0337
212641_at	*VSX1*	human immunodeficiency virus type I enhancer binding protein 2	0.559	0.0338
204971_at	*PARD3B*	cystatin A	1.892	0.0338
225508_at	*ART4*	KIAA1468	0.539	0.0338
244738_at	*EML4*	bromodomain and WD repeat domain containing 3	0.272	0.0338
227235_at	*CTSO*	guanylate cyclase 1 soluble subunit alpha	0.807	0.0339

204222_s_at	*PCDHA1///PCDHA2///PCDHA3///PCDHA4///PCDHA5///PCDHA6///PCDHA7///PCDHA8///PCDHA10///PCDHA11///PCDHA12///PCDHA13///PCDHAC1///PCDHAC2///PCDHA9*	GLI pathogenesis related 1	0.664	0.0340
205011_at	*SLC25A10*	von Willebrand factor A domain containing 5A	0.669	0.0340
204136_at	*OTP*	collagen type VII alpha 1 chain	0.876	0.0340
1554168_a_at	*CDKN2AIPNL*	SH3 domain containing kinase binding protein 1	0.657	0.0340
233119_at	*NACC2*	proline rich 5 like	0.293	0.0340
204606_at	*LOC102724984///KIAA2013*	C-C motif chemokine ligand 21	0.786	0.0342
206796_at	*AGAP9*	WNT1 inducible signaling pathway protein 1	0.480	0.0343
202686_s_at	*LRP10*	AXL receptor tyrosine kinase	0.866	0.0343
206746_at	*ABAT*	beaded filament structural protein 1	0.274	0.0343
219352_at	*DEF8*	HECT and RLD domain containing E3 ubiquitin protein ligase family member 6	0.968	0.0343
229931_at	*CALY*	zinc finger protein 775	0.216	0.0344
204854_at	*GMNC*	prolyl 3-hydroxylase 3	0.422	0.0344
204352_at	*APBB1*	TNF receptor associated factor 5	0.687	0.0344
201050_at	*FAM13A-AS1*	phospholipase D family member 3	0.420	0.0345
201426_s_at	*CPSF2*	vimentin	0.757	0.0345
228258_at	*R3HDM4*	TBC1 domain family member 10C	0.902	0.0346
216250_s_at	*WNT5A*	leupaxin	0.739	0.0346
226137_at	*NBPF26///NBPF10///NBPF14*	zinc finger homeobox 3	0.610	0.0347
201809_s_at	*PRDM2*	endoglin	0.634	0.0347
211663_x_at	*CCR7*	prostaglandin D2 synthase	0.918	0.0348
1557169_x_at	*ANPEP*	HLA complex group 11 (non-protein coding)	0.280	0.0348
203900_at	*CDH8*	seizure threshold 2 homolog (mouse)	0.265	0.0349
213503_x_at	*WDR11*	annexin A2	0.770	0.0349
222812_s_at	*TRIB2*	ras homolog family member F, filopodia associated	0.343	0.0349
204891_s_at	*CPVL*	LCK proto-oncogene, Src family tyrosine kinase	1.053	0.0351
235172_at	*LOC101928173*	GABPB1 antisense RNA 1	0.702	0.0352
209442_x_at	*PLAC1*	ankyrin 3, node of Ranvier (ankyrin G)	0.912	0.0352
222546_s_at	*NLRC3*	EPS8 like 2	0.240	0.0352
215145_s_at	*EFR3A*	contactin associated protein-like 2	0.463	0.0352

208851_s_at	*NDUFV3*	Thy-1 cell surface antigen	0.773	0.0353
1556145_a_at	*FNDC1*	uncharacterized LOC100131170	0.201	0.0353
208544_at	*RP9P*	adrenoceptor alpha 2B	0.264	0.0353
231766_s_at	*ZMIZ2*	collagen type XII alpha 1 chain	1.006	0.0354
221875_x_at	*ZMAT2*	major histocompatibility complex, class I, F	0.647	0.0354
242137_at	*SH3BP5-AS1*	RNA binding motif single stranded interacting protein 3	0.233	0.0355
1558827_a_at	*TK2*	zinc finger protein 831	0.266	0.0355
230263_s_at	*TUG1*	dedicator of cytokinesis 5	0.789	0.0356
223253_at	*SLC25A40*	ependymin related 1	0.739	0.0356
225791_at	*KLC1*	ubiquitin conjugating enzyme E2 F (putative)	0.370	0.0357
204575_s_at	*HTT*	matrix metallopeptidase 19	0.744	0.0358
231251_at	*SNAI2*	WAS/WASL interacting protein family member 2	0.269	0.0358
219102_at	*CROCC*	reticulocalbin 3	0.440	0.0359
228367_at	*FAM198B*	alpha kinase 2	0.481	0.0360
224916_at	*DDHD1*	transmembrane protein 173	0.616	0.0360
236179_at	*LINC01468*	cadherin 11	0.917	0.0361
227347_x_at	*MAN2C1*	hes family bHLH transcription factor 4	0.854	0.0361
230214_at	*OPRK1*	murine retrovirus integration site 1 homolog	0.592	0.0361
225629_s_at	*LINC01180*	zinc finger and BTB domain containing 4	0.289	0.0364
221830_at	*LRG1*	RAP2A, member of RAS oncogene family	0.549	0.0364
212203_x_at	*RAB18*	interferon induced transmembrane protein 3	0.713	0.0365
227276_at	*PSG9*	plexin domain containing 2	0.632	0.0365
221666_s_at	*MTG2*	PYD and CARD domain containing	0.860	0.0365
239779_at	*DIRAS3*	uncharacterized LOC101930595///protein-glucosylgalactosylhydroxylysine glucosidase	0.318	0.0366
218638_s_at	*NOL4L*	uncharacterized LOC100130872///spondin 2	0.778	0.0367
204806_x_at	*SNED1*	major histocompatibility complex, class I, F	0.617	0.0367
222449_at	*TMCO6*	prostate transmembrane protein, androgen induced 1	0.680	0.0368
207760_s_at	*MAOA*	nuclear receptor corepressor 2	0.334	0.0368
218775_s_at	*IL2RB*	WW and C2 domain containing 2///claudin 22	0.270	0.0369
222450_at	*ZNF334*	prostate transmembrane protein, androgen induced 1	0.882	0.0369
233408_at	*LINC00665*	phosphatidylinositol-3,4,5-trisphosphate dependent Rac exchange factor 2	0.191	0.0370
211896_s_at	*ARHGAP44*	decorin	1.225	0.0371
206224_at	*XPNPEP1*	cystatin SN	0.539	0.0371
37005_at	*ENPEP*	MINOS1-NBL1 readthrough///neuroblastoma 1, DAN family BMP antagonist	0.650	0.0372
206779_s_at	*NOV*	acetylserotonin O-methyltransferase	0.180	0.0373

232843_s_at	*CACNA1E*	dedicator of cytokinesis 8	0.688	0.0373
205619_s_at	*ZNRF2P1*	mesenchyme homeobox 1	0.730	0.0374
214369_s_at	*RND2*	RAS guanyl releasing protein 2	0.415	0.0375
226659_at	*TSHZ2*	DEF6, guanine nucleotide exchange factor	0.726	0.0375
211796_s_at	*ZNF174*	T cell receptor beta constant 1	1.412	0.0375
204164_at	*DOK3*	signal-induced proliferation-associated 1	0.474	0.0376
1554018_at	*ATP11A*	glycoprotein nmb	0.786	0.0376
239915_at	*PDGFB*	HOXA cluster antisense RNA 3	0.274	0.0376
1552295_a_at	*SH3GL1P2*	solute carrier family 39 member 13	0.349	0.0379
207118_s_at	*SENP5*	matrix metallopeptidase 23A (pseudogene)///matrix metallopeptidase 23B	0.477	0.0379
225269_s_at	*SUPT3H*	RNA binding motif single stranded interacting protein 1	0.322	0.0379
1564635_a_at	*CDH6*	forkhead associated phosphopeptide binding domain 1	0.193	0.0379
236297_at	*RAMP2-AS1*	plexin domain containing 2	0.553	0.0380
228642_at	*UBE2U*	HOXA transcript antisense RNA, myeloid-specific 1	1.002	0.0380
212884_x_at	*EML3*	apolipoprotein E	0.493	0.0380
201508_at	*GPR52*	insulin like growth factor binding protein 4	0.695	0.0381
205059_s_at	*LOC101926921 ///DAB2*	iduronidase, alpha-L-	0.419	0.0382
1552318_at	*STAU2-AS1*	GTPase, IMAP family member 1	0.534	0.0383
205900_at	*IBA57*	keratin 1	1.981	0.0383
213417_at	*HLA-DQB1*	T-box 2	0.537	0.0383
203813_s_at	*KCNN1*	slit guidance ligand 3	0.554	0.0385
203385_at	*GPR182*	diacylglycerol kinase alpha	0.678	0.0385
1558371_a_at	*ARHGAP33*	glutamate rich 1	0.453	0.0385
220016_at	*MEIOC*	AHNAK nucleoprotein	0.808	0.0386
203279_at	*LOC401261*	ER degradation enhancing alpha-mannosidase like protein 1	0.521	0.0387
208466_at	*ANKRD10-IT1*	RAB3D, member RAS oncogene family	0.225	0.0388
204929_s_at	*CXCL13*	vesicle associated membrane protein 5	0.722	0.0390
221555_x_at	*GPATCH2L*	cell division cycle 14B	0.495	0.0391
234989_at	*CKAP4*	microRNA 612///nuclear paraspeckle assembly transcript 1 (non-protein coding)	0.896	0.0391
207166_at	*LOC101927228*	G protein subunit gamma transducin 1	0.447	0.0391
219407_s_at	*GPR157*	laminin subunit gamma 3	0.424	0.0391
202307_s_at	*FBRS*	transporter 1, ATP binding cassette subfamily B member	0.880	0.0391
235567_at	*CCDC50*	RAR related orphan receptor A	0.745	0.0391
209994_s_at	*IQCB1*	ATP binding cassette subfamily B member 4///ATP binding cassette subfamily B member 1	0.371	0.0392
204344_s_at	*LOC100287290*	Sec23 homolog A, coat complex II component	0.277	0.0394
230000_at	*PRMT5-AS1*	ring finger protein 213	0.788	0.0395

228488_at	*FBXL14*	TBC1 domain family member 16	0.575	0.0395
211530_x_at	*KRT6B*	major histocompatibility complex, class I, G	0.454	0.0396
1566324_a_at	*SRL*	MAF bZIP transcription factor	0.351	0.0397
224075_s_at	*COL6A5*	visual system homeobox 1	0.267	0.0397
228411_at	*SNORA17B///SNORA17A///SNHG7*	par-3 family cell polarity regulator beta	0.549	0.0398
223718_at	*FAM163A*	acrosin binding protein	0.263	0.0399
228673_s_at	*ELFN2*	echinoderm microtubule associated protein like 4	0.499	0.0400
203758_at	*USP4*	cathepsin O	0.815	0.0401
1555970_at	*WARS*	F-box protein 28	0.324	0.0401
208933_s_at	*FAAH*	galectin 8	0.678	0.0401
211675_s_at	*SHE*	MyoD family inhibitor domain containing	0.837	0.0402
212993_at	*PLCH1*	NACC family member 2	0.682	0.0402
213290_at	*DUSP7*	collagen type VI alpha 2 chain	0.581	0.0402
232201_at	*CORIN*	naked cuticle homolog 2	0.504	0.0402
227776_at	*IKZF3*	alkaline ceramidase 3	0.707	0.0403
221601_s_at	*SLC15A2*	Fc fragment of IgM receptor	0.641	0.0403
204049_s_at	*POU6F1*	phosphatase and actin regulator 2	0.704	0.0404
205997_at	*S100PBP*	ADAM metallopeptidase domain 28	0.515	0.0404
207977_s_at	*ME3*	dermatopontin	0.870	0.0404
224706_at	*INPP4B*	uncharacterized protein KIAA2013///KIAA2013	0.304	0.0404
204048_s_at	*EPHA6*	phosphatase and actin regulator 2	0.742	0.0404
202196_s_at	*RALGPS1*	dickkopf WNT signaling pathway inhibitor 3	0.781	0.0404
208364_at	*LAMC2*	inositol polyphosphate-4-phosphatase type I A	0.238	0.0405
223725_at	*C21orf91-OT1*	long intergenic non-protein coding RNA 852	0.496	0.0406
240230_s_at	*FBXO9*	ArfGAP with GTPase domain, ankyrin repeat and PH domain 9	0.549	0.0406
227252_at	*WIPF1*	LDL receptor related protein 10	0.482	0.0406
209460_at	*CER1*	4-aminobutyrate aminotransferase	0.365	0.0407
210786_s_at	*RNF217*	Fli-1 proto-oncogene, ETS transcription factor	0.313	0.0407
225637_at	*FUT11*	differentially expressed in FDCP 8 homolog	0.358	0.0407
219896_at	*ZBTB20*	calcyon neuron specific vesicular protein	0.253	0.0408
212489_at	*FLJ46875*	collagen type V alpha 1 chain	0.935	0.0408
209536_s_at	*CCL28*	EH domain containing 4	0.509	0.0409
1558711_at	*ZNF506*	FAM13A antisense RNA 1	0.329	0.0410
233208_x_at	*LOC101929368///FKBP1A*	cleavage and polyadenylation specific factor 2	0.366	0.0410
235836_at	*KATNBL1*	matrix remodeling associated 7	0.286	0.0410
221764_at	*KLF13*	R3H domain containing 4	0.274	0.0411
205990_s_at	*LOC100996694*	Wnt family member 5A	1.137	0.0412

226740_x_at	*SMAD7*	neuroblastoma breakpoint family member 26///neuroblastoma breakpoint family member 10///neuroblastoma breakpoint family member 14	0.470	0.0412
217430_x_at	*LOC101927533 ///LOC440867*	collagen type I alpha 1 chain	0.333	0.0412
203056_s_at	*ACACA*	PR/SET domain 2	0.534	0.0412
206337_at	*GCG*	C-C motif chemokine receptor 7	0.888	0.0413
40489_at	*FOXO1*	atrophin 1	0.273	0.0414
202888_s_at	*GPR155*	alanyl aminopeptidase, membrane	0.695	0.0414
222184_at	*RFC3*	long intergenic non-protein coding RNA 965	0.471	0.0414
205792_at	*XRCC4*	WNT1 inducible signaling pathway protein 2	1.048	0.0415
222024_s_at	*NT5M*	A-kinase anchoring protein 13	0.633	0.0415
229694_at	*PLOD2*	WD repeat domain 11	0.549	0.0416
202479_s_at	*HLA-J*	tribbles pseudokinase 2	0.525	0.0416
208146_s_at	*HDLBP*	carboxypeptidase, vitellogenic like	0.826	0.0416
220731_s_at	*TFPI*	NECAP endocytosis associated 2	0.390	0.0418
235733_at	*COL28A1*	glucoside xylosyltransferase 2	0.508	0.0418
202943_s_at	*VRK3*	alpha-N-acetylgalactosaminidase	0.327	0.0418
236787_at	*C21orf58*	uncharacterized LOC101928173	0.545	0.0420
236295_s_at	*CLN5*	NLR family CARD domain containing 3	0.848	0.0420
212150_at	*RASL11A*	EFR3 homolog A	0.602	0.0421
226930_at	*DIRC2*	fibronectin type III domain containing 1	0.749	0.0422
239027_at	*ZNF135*	dedicator of cytokinesis 8	0.581	0.0423
215388_s_at	*SLC25A51*	complement factor H related 1///complement factor H	1.108	0.0424
213943_at	*LMO4*	twist family bHLH transcription factor 1	1.022	0.0424
221924_at	*AAMDC*	zinc finger MIZ-type containing 2	0.315	0.0424

Under-expressed genes in Metastases vs. Primary MCC tumors

1555095_at	*LINC01558*	long intergenic non-protein coding RNA 1558	-1.161	0.0001
207698_at	*GPR78///CPZ*	long intergenic non-protein coding RNA 1558	-0.841	0.0004
238862_at	*PLEC*	major facilitator superfamily domain containing 4A	-0.329	0.0005
225762_x_at	*FZD10*	RNA, 45S pre-ribosomal 5	-1.100	0.0007
239665_at	*LRRC15*	uncharacterized LOC441179	-1.816	0.0007
1552738_a_at	*INHBA*	suppression of tumorigenicity 7 like	-0.539	0.0007
232192_at	*ADAMTS2*	uncharacterized LOC153811///ring finger protein 130	-0.670	0.0008
231885_at	*SERPINE2*	zinc finger protein 451	-0.259	0.0010

235102_x_at	*ITGB5*	small nucleolar RNA, C/D box 3D///small nucleolar RNA, C/D box 3C///small nucleolar RNA, C/D box 3B-2///small nucleolar RNA, C/D box 3A///small nucleolar RNA, C/D box 3B-1	-1.215	0.0011
225767_at	*MRPL12*	RNA, 45S pre-ribosomal 5	-1.274	0.0011
229165_at	*ZNF70*	mitochondrial ribosomal protein L12	-0.398	0.0013
1554578_at	*COL6A1*	zinc finger protein 70	-0.447	0.0015
222944_s_at	*PRRX1*	MAGI family member, X-linked	-0.329	0.0015
242048_at	*LUM*	microRNA 6834///prefoldin subunit 6	-0.354	0.0019
224059_s_at	*NEBL*	NUMB like, endocytic adaptor protein	-0.427	0.0019
242141_at	*LAMP5*	histone deacetylase 2	-0.542	0.0021
207279_s_at	*PCOLCE*	nebulette	-0.626	0.0023
229862_x_at	*SPANXA2-OT1*	zinc finger and BTB domain containing 45	-0.411	0.0024
1564491_at	*MMP2*	SPANXA2 overlapping transcript 1	-0.271	0.0027
230338_x_at	*CYP1B1-AS1*	GS homeobox 2	-0.306	0.0033
234633_at	*MCU*	keratin associated protein 4-11	-0.261	0.0033
207092_at	*PI15*	leptin	-0.445	0.0036
233410_at	*LGR4*	CYP1B1 antisense RNA 1	-0.256	0.0037
220661_s_at	*KLHL5*	zinc finger protein 692	-0.526	0.0038
229443_at	*PDK3*	ubiquinol-cytochrome c reductase complex assembly factor 2	-0.383	0.0038
1553690_at	*AMMECR1*	shugoshin 1	-0.388	0.0038
230674_at	*CFH*	leucine rich repeat containing G protein-coupled receptor 4	-0.223	0.0039
205985_x_at	*CDC14B*	chloride voltage-gated channel Kb	-0.422	0.0040
214154_s_at	*FNBP1*	plakophilin 2	-0.300	0.0041
243952_at	*CXCL14*	transmembrane phosphatase with tensin homology pseudogene 1	-0.783	0.0042
207321_s_at	*COL10A1*	ATP binding cassette subfamily B member 9	-0.415	0.0044
1560851_at	*MARCH3*	long intergenic non-protein coding RNA 619	-0.277	0.0045
215300_s_at	*MIR100HG*	flavin containing monooxygenase 5	-0.267	0.0045
204590_x_at	*RFX5*	VPS33A, CORVET/HOPS core subunit	-0.296	0.0047
227316_at	*FRMD6*	PET117 homolog	-0.312	0.0047
230448_at	*COL6A3*	solute carrier family 38 member 10	-0.324	0.0048
230385_at	*NT5E*	membrane associated ring-CH-type finger 3	-0.365	0.0049
232753_at	*PDLIM2*	zinc finger protein 346	-0.351	0.0053
202964_s_at	*EXOC3L4*	regulatory factor X5	-0.313	0.0054
227281_at	*RGS5*	solute carrier family 29 member 4	-0.340	0.0054
244001_at	*LEF1*	nucleosome assembly protein 1 like 4	-0.205	0.0055
219365_s_at	*ATXN1*	CaM kinase like vesicle associated	-0.763	0.0057
1567179_at	*ZNF221*	uncharacterized LOC105376944	-0.233	0.0057
230677_at	*IGHV1-69*	exocyst complex component 3 like 4	-0.443	0.0058
1558785_a_at	*PHACTR2-AS1*	regulator of G-protein signaling 5	-0.260	0.0059
231854_at	*ZNF781*	phosphatidylinositol-4,5-bisphosphate 3-kinase catalytic subunit alpha	-0.342	0.0059

240429_at	*ECM1*	zinc finger protein 546	-0.282	0.0060
218163_at	*MSI2*	MCTS1, re-initiation and release factor	-0.549	0.0062
233848_x_at	*TLN1*	zinc finger protein 221	-0.272	0.0063
206037_at	*NINJ2*	kynurenine aminotransferase 1	-0.533	0.0065
233576_at	*C7orf50*	3-hydroxymethyl-3-methylglutaryl-CoA lyase like 1	-0.293	0.0066
240915_at	*EVA1B*	immunoglobulin heavy variable 1-69	-0.215	0.0069
1556859_a_at	*FXYD5*	PHACTR2 antisense RNA 1	-0.278	0.0070
1552785_at	*LOC145783///ZNF280D*	zinc finger protein 781	-0.180	0.0071
241470_x_at	*SYNE3///LINC00341*	Ras association domain family member 9	-0.401	0.0071
220389_at	*PREX1*	coiled-coil domain containing 81	-0.288	0.0072
243579_at	*STARD13*	musashi RNA binding protein 2	-0.605	0.0072
213664_at	*LOC100128840*	solute carrier family 1 member 1	-0.658	0.0072
232763_at	*CMTM3*	talin 1	-0.367	0.0072
204069_at	*ARHGEF39*	Meis homeobox 1	-0.735	0.0074
240147_at	*LOC101928491*	chromosome 7 open reading frame 50	-0.318	0.0075
229013_at	*ZKSCAN8*	uncharacterized LOC145783///zinc finger protein 280D	-0.469	0.0078
1568853_at	*CTSA*	uncharacterized LOC101927929	-0.313	0.0078
1554360_at	*CHST11*	FCH and double SH3 domains 2	-0.407	0.0078
213085_s_at	*LOC645188*	WW and C2 domain containing 1	-0.920	0.0079
1562783_at	*KLHL20*	uncharacterized LOC100128840	-0.693	0.0080
231892_at	*ZBTB42*	Rho guanine nucleotide exchange factor 39	-0.471	0.0081
216669_at	*BECN1*	prenyl (decaprenyl) diphosphate synthase, subunit 2	-0.268	0.0082
204876_at	*ARL4C*	zinc finger protein 646	-0.268	0.0082
1561567_at	*ARL17B///ARL17A*	uncharacterized LOC101928491	-0.266	0.0083
1554216_at	*GTDC1*	VPS50, EARP/GARPII complex subunit	-0.183	0.0083
201686_x_at	*FLNA*	apoptosis inhibitor 5	-0.474	0.0083
206579_at	*EDA*	zinc finger with KRAB and SCAN domains 8	-0.253	0.0085
235176_at	*SOCS1*	ZFP82 zinc finger protein	-0.373	0.0087
1560895_at	*ZNF780B*	uncharacterized LOC645188	-0.283	0.0087
210634_at	*LRRK2*	kelch like family member 20	-0.238	0.0090
1554047_at	*WISP1*	thioredoxin domain containing 9	-0.285	0.0091
228969_at	*COL5A2*	anterior gradient 2, protein disulphide isomerase family member	-0.234	0.0091
227913_at	*GABARAPL3*	exosome component 3	-0.281	0.0091
1554245_x_at	*MEG3*	ADP ribosylation factor like GTPase 17B///ADP ribosylation factor like GTPase 17A	-0.545	0.0092
236457_at	*ELK4*	glycosyltransferase like domain containing 1	-0.301	0.0092
241911_at	*RASSF2*	cyclin dependent kinase like 3	-0.450	0.0094

1559218_s_at	*STK10*	nuclear transcription factor Y subunit gamma	-0.235	0.0094
1569973_at	*NAT1*	septin 7 pseudogene 2	-0.427	0.0094
206217_at	*SORBS3*	ectodysplasin A	-0.407	0.0094
51158_at	*GLIPR2*	family with sequence similarity 174 member B	-0.659	0.0095
243294_at	*SRGAP2C///SRGAP2B///SRGAP2*	zinc finger protein 780B	-0.386	0.0097
242070_at	*FLT3LG*	uncharacterized LOC728485	-0.685	0.0098
207665_at	*GPR21*	ADAM metallopeptidase domain 21	-0.276	0.0098
238903_at	*HLA-A*	UBX domain protein 2B	-0.396	0.0101
211457_at	*MSRB3*	GABA type A receptor associated protein like 3 pseudogene	-0.317	0.0101
206919_at	*FAHD2A*	ELK4, ETS transcription factor	-0.273	0.0103
1554059_at	*ZSWIM4*	SET domain and mariner transposase fusion gene	-0.580	0.0107
211773_s_at	*GGT5*	zinc finger with KRAB and SCAN domains 3	-0.310	0.0107
206970_at	*DPT*	contactin 2	-0.473	0.0110
217443_at	*TMEM167A*	uncharacterized LOC101927792	-0.224	0.0110
221294_at	*SMG9*	G protein-coupled receptor 21	-0.295	0.0111
1564066_at	*ENTPD1*	transmembrane protein 151B	-0.343	0.0114
235621_at	*PLXDC2*	fumarylacetoacetate hydrolase domain containing 2A	-0.334	0.0114
205067_at	*ADRA2A*	interleukin 1 beta	-0.710	0.0115
238263_at	*ZNF530*	EPHA1 antisense RNA 1	-0.243	0.0115
223486_at	*MYOG*	GTP binding protein 8 (putative)	-0.569	0.0115
214515_at	*NDUFV2-AS1*	olfactory receptor family 1 subfamily E member 1	-0.248	0.0115
219270_at	*STX11*	ChaC glutathione specific gamma-glutamylcyclotransferase 1	-0.460	0.0118
231915_at	*ELAVL3*	zinc finger SWIM-type containing 4	-0.252	0.0119
238708_at	*LRRC17*	ZNF582 antisense RNA 1 (head to head)	-0.343	0.0120
1554852_a_at	*LOC100505666*	KIAA1257	-0.425	0.0121
208378_x_at	*EPHA4*	fibroblast growth factor 5	-0.417	0.0121
226276_at	*GUCY1A3*	transmembrane protein 167A	-0.605	0.0122
233990_at	*NRP1*	SMG9, nonsense mediated mRNA decay factor	-0.280	0.0124
219274_at	*RPS15A*	tetraspanin 12	-1.263	0.0126
228090_at	*IPO5P1*	nicotinamide nucleotide adenylyltransferase 3	-0.551	0.0131
1554894_a_at	*LRRC41*	pterin-4 alpha-carbinolamine dehydratase 2	-0.347	0.0131
222374_at	*COL1A1*	beta-transducin repeat containing E3 ubiquitin protein ligase	-0.338	0.0131
236634_at	*FBLIM1*	chromosome 8 open reading frame 48	-0.498	0.0131
232106_s_at	*TBC1D2B*	centrosomal protein 89	-0.388	0.0131
244664_at	*HSPA5*	zinc finger protein 530	-0.577	0.0133
207282_s_at	*IL12RB2*	myogenin (myogenic factor 4)	-0.855	0.0133

228436_at	*COL6A6*	potassium voltage-gated channel subfamily C member 4	-0.688	0.0136
1562748_at	*LOC101928747///RBMX///SNORD61*	LINGO1 antisense RNA 1	-0.302	0.0136
206338_at	*NUCB2*	ELAV like neuron-specific RNA binding protein 3	-0.369	0.0137
215518_at	*NCL*	syntaxin binding protein 5 like	-0.551	0.0138
1567240_x_at	*LOC102725438*	olfactory receptor family 2 subfamily L member 2	-0.241	0.0138
235886_at	*LNP1*	uncharacterized LOC100505666	-0.344	0.0138
213297_at	*LPAR1*	required for meiotic nuclear division 5 homolog B	-0.297	0.0139
1559441_s_at	*ZC3H10*	uncharacterized LOC651430	-0.179	0.0140
206736_x_at	*LMAN2*	cholinergic receptor nicotinic alpha 4 subunit	-0.220	0.0141
235979_at	*ABHD2*	complement component 7	-0.264	0.0141
219551_at	*H1F0*	ELL associated factor 2	-0.639	0.0143
229882_at	*KIF24*	ribosomal protein S15a	-0.408	0.0144
230626_at	*FAM171B*	tetraspanin 12	-0.969	0.0144
1565748_at	*LAMP1*	importin 5 pseudogene 1	-0.306	0.0146
215765_at	*HGD*	leucine rich repeat containing 41	-0.349	0.0147
240294_at	*BMP1*	homeodomain interacting protein kinase 2	-0.309	0.0148
204665_at	*YWHAB*	suppressor of IKBKE 1	-0.303	0.0148
230891_at	*FNDC5*	tubulin epsilon 1	-0.239	0.0151
231479_at	*MYO5A*	tetratricopeptide repeat domain 33	-0.238	0.0152
1558809_s_at	*TSPAN11*	ZNF790 antisense RNA 1	-0.426	0.0153
229999_at	*KCNMB3*	uncharacterized LOC101928747///RNA binding motif protein, X-linked///small nucleolar RNA, C/D box 61	-0.297	0.0153
229838_at	*ZNF461*	nucleobindin 2	-0.425	0.0153
241892_at	*ADAM11*	nucleolin	-0.333	0.0153
239043_at	*TMEM221*	zinc finger protein 404	-1.056	0.0154
219580_s_at	*GRK3*	transmembrane channel like 5	-0.470	0.0155
1570237_at	*BRE*	uncharacterized LOC102725438	-0.221	0.0155
207802_at	*SLITRK5*	cysteine rich secretory protein 3	-0.461	0.0155
227181_at	*MOBP*	leukemia NUP98 fusion partner 1	-0.408	0.0155
202526_at	*ZNF702P*	SMAD family member 4	-0.220	0.0156
227430_at	*LOC101060835///HLA-DQB1*	zinc finger CCCH-type containing 10	-0.434	0.0159
207775_at	*MTM1*	uncharacterized LOC79150	-0.193	0.0159
200805_at	*CRISPLD2*	lectin, mannose binding 2	-0.345	0.0159
244673_at	*RCOR3*	KIAA1841	-0.178	0.0160
1557070_at	*SLC12A4*	TFAP2A antisense RNA 1	-0.449	0.0161
1559923_at	*SYNC*	glutamate ionotropic receptor kainate type subunit 1	-0.208	0.0161
236785_at	*RRP7BP///RRP7A*	uncharacterized LOC101928461	-0.359	0.0162
1552862_at	*RGS12*	RUSC1 antisense RNA 1	-0.312	0.0162

220652_at	*GPNMB*	kinesin family member 24	-0.219	0.0163
232598_at	*CLEC10A*	nucleoporin 210 like	-0.293	0.0163
227370_at	*FANCC*	family with sequence similarity 171 member B	-0.947	0.0164
1552637_at	*SAMD9L*	protein tyrosine phosphatase, non-receptor type 11	-0.227	0.0164
1553553_at	*ZBTB26*	taste 2 receptor member 39	-0.289	0.0165
231581_at	*APOA2*	long intergenic non-protein coding RNA 664	-0.300	0.0166
214308_s_at	*STAMBPL1*	homogentisate 1,2-dioxygenase	-0.338	0.0170
1556850_at	*DDX60L*	centrosomal protein 290	-0.279	0.0170
219185_at	*CH25H*	sirtuin 5	-0.476	0.0173
236725_at	*HBB*	WW and C2 domain containing 1	-0.762	0.0173
241089_at	*FABP4*	zinc finger protein 562	-0.209	0.0173
1562005_at	*GIT2*	Ras and Rab interactor 3	-0.254	0.0174
1556232_at	*CCDC116*	kinesin family member 6	-0.431	0.0174
228415_at	*GADD45GIP1*	adaptor related protein complex 1 sigma 2 subunit	-0.678	0.0174
226097_at	*AFF3*	fibronectin type III domain containing 5	-0.482	0.0175
225030_at	*SLC25A37*	biorientation of chromosomes in cell division 1	-0.508	0.0175
225512_at	*BRINP3*	zinc finger and BTB domain containing 38	-0.583	0.0176
225358_at	*SLC6A9*	DnaJ heat shock protein family (Hsp40) member C19	-0.551	0.0176
221125_s_at	*GPR107*	potassium calcium-activated channel subfamily M regulatory beta subunit 3	-0.406	0.0176
1560446_at	*ENTPD6*	importin 5 pseudogene 1	-0.354	0.0178
232391_at	*PALMD*	zinc finger protein 461	-0.200	0.0178
207880_at	*FAAP20*	ADAM metallopeptidase domain 11	-0.682	0.0178
239128_at	*ZNF333*	transmembrane protein 221	-0.319	0.0178
226260_x_at	*HECTD4*	zinc finger protein 358	-0.275	0.0179
214930_at	*OSBPL2*	SLIT and NTRK like family member 5	-0.899	0.0181
210193_at	*MPPED1*	myelin-associated oligodendrocyte basic protein	-0.219	0.0181
226961_at	*BCL2L12*	proline rich 15	-0.345	0.0181
206557_at	*HOXA10-AS///MIR196B*	zinc finger protein 702, pseudogene	-0.341	0.0181
217040_x_at	*EFHD2*	SRY-box 15	-0.441	0.0183
218214_at	*TKT*	autophagy related 101	-0.439	0.0185
241433_at	*PRKCDBP*	REST corepressor 3	-0.567	0.0186
209401_s_at	*LPAR5*	solute carrier family 12 member 4	-0.223	0.0187
202938_x_at	*PDGFRB*	ribosomal RNA processing 7 homolog B, pseudogene///ribosomal RNA processing 7 homolog A	-0.319	0.0189
1559513_a_at	*CYGB*	Fanconi anemia complementation group C	-0.209	0.0191
1554973_a_at	*ZNF17*	zinc finger and BTB domain containing 26	-0.290	0.0192
219466_s_at	*TPBG*	apolipoprotein A2	-0.208	0.0192
230919_at	*ENTPD4*	zinc finger protein 233	-0.463	0.0192
215341_at	*MYOF*	dynein axonemal heavy chain 6	-0.317	0.0193

236623_at	*VDR*	ATP1A1 antisense RNA 1	-0.247	0.0194
206891_at	*ING4*	actinin alpha 3 (gene/pseudogene)	-0.295	0.0197
231430_at	*KIAA1644*	family with sequence similarity 181 member B	-0.353	0.0198
211696_x_at	*UBE3D*	hemoglobin subunit beta	-1.309	0.0200
235978_at	*LOC101927460*	fatty acid binding protein 4	-0.498	0.0200
201342_at	*SPTLC1*	small nuclear ribonucleoprotein polypeptide C	-0.295	0.0200
235414_at	*JAK2*	zinc finger protein 383	-0.323	0.0201
1555792_a_at	*MAPK1*	coiled-coil domain containing 116	-0.260	0.0201
212889_x_at	*MRPS22*	GADD45G interacting protein 1	-0.213	0.0202
243967_at	*COL2A1*	AF4/FMR2 family member 3	-0.263	0.0202
231078_at	*DNM3OS*	solute carrier family 25 member 37	-0.335	0.0203
242581_at	*OLFML1*	mitogen-activated protein kinase kinase kinase 15	-0.292	0.0203
217562_at	*N4BP2L2*	BMP/retinoic acid inducible neural specific 3	-0.907	0.0204
223791_at	*MARVELD3*	family with sequence similarity 27 member B///family with sequence similarity 27 member C	-0.576	0.0205
220264_s_at	*ARHGAP17*	G protein-coupled receptor 107	-0.344	0.0205
242199_at	*CASK*	uncharacterized LOC101928812	-0.207	0.0205
201704_at	*PEA15*	ectonucleoside triphosphate diphosphohydrolase 6 (putative)	-0.286	0.0205
236584_at	*JADE1*	Fanconi anemia core complex associated protein 20	-0.203	0.0206
1569251_a_at	*WDR78*	zinc finger protein 333	-0.222	0.0206
244378_at	*TIAM2*	HECT domain E3 ubiquitin protein ligase 4	-0.261	0.0207
209943_at	*CRB1*	F-box and leucine rich repeat protein 4	-0.399	0.0208
216074_x_at	*PTPN21*	WW and C2 domain containing 1	-0.875	0.0209
1558481_s_at	*EHD4*	TMCC1 antisense RNA 1 (head to head)	-0.287	0.0210
227788_at	*ARSK*	ubiquitin specific peptidase 13 (isopeptidase T-3)	-0.360	0.0211
228205_at	*KALRN*	transketolase	-0.253	0.0213
236721_at	*PFDN4*	alkB homolog 1, histone H2A dioxygenase	-0.269	0.0213
223731_at	*ZNF558*	MYCBP associated protein	-0.281	0.0214
238850_at	*NORAD*	long intergenic non-protein coding RNA 461///microRNA 9-2	-1.364	0.0215
206900_x_at	*DCN*	zinc finger protein 253	-0.317	0.0217
1559538_at	*PPEF1*	uncharacterized LOC101929549	-0.252	0.0218
1558183_at	*S100A10*	zinc finger protein 17	-0.185	0.0218
1552711_a_at	*AGRN*	cytochrome b5 domain containing 1	-0.416	0.0219
217607_x_at	*LOC100126784*	eukaryotic translation initiation factor 4 gamma 2	-0.212	0.0220
223785_at	*PTRF*	Fanconi anemia complementation group I	-0.442	0.0222
228346_at	*HTRA3*	zinc finger protein 844	-0.720	0.0223
218234_at	*NDUFS1*	inhibitor of growth family member 4	-0.263	0.0225

1557322_at	*TRIM55*	zinc finger protein 230	-0.299	0.0225
241927_x_at	*SELM*	cell division cycle 34	-0.300	0.0225
233327_at	*DUSP4*	ubiquitin protein ligase E3D	-0.244	0.0226
1562934_at	*PRKD3*	uncharacterized LOC101927460	-0.293	0.0226
227753_at	*IFITM1*	transmembrane protein 139	-0.469	0.0227
227793_at	*SHISA5*	microRNA let-7d	-0.523	0.0228
223448_x_at	*B4GALNT3*	mitochondrial ribosomal protein S22	-0.357	0.0229
232332_at	*TFE3*	KIAA1210	-0.214	0.0230
1552739_s_at	*RHOJ*	suppression of tumorigenicity 7 like	-0.475	0.0232
218517_at	*EIF4E*	jade family PHD finger 1	-0.440	0.0232
1554141_s_at	*LOC101928324*	WD repeat domain 78	-0.270	0.0232
219950_s_at	*CHPT1*	T-cell lymphoma invasion and metastasis 2	-0.317	0.0233
217232_x_at	*NAGA*	hemoglobin subunit beta	-1.106	0.0234
244403_at	*C2CD4B*	crumbs 1, cell polarity complex component	-1.003	0.0234
1562825_at	*GLT8D2*	uncharacterized LOC105372404	-0.191	0.0234
239623_at	*HNRNPA3P1*	chromosome 5 open reading frame 63	-0.248	0.0236
200018_at	*DNAJC5*	uncharacterized LOC100508408///small nucleolar RNA, C/D box 14B///ribosomal protein S13	-0.221	0.0236
210194_at	*SUN1*	phospholipase A2 receptor 1	-0.249	0.0237
229438_at	*SLC2A10*	family with sequence similarity 20 member C	-0.325	0.0238
244705_at	*EMILIN1*	POT1 antisense RNA 1	-0.203	0.0239
209116_x_at	*ATXN3*	hemoglobin subunit beta	-1.349	0.0241
1569132_s_at	*PDLIM4*	arylsulfatase family member K	-0.204	0.0242
225981_at	*ARMC6*	HID1 domain containing	-0.447	0.0244
221112_at	*CENPI*	interleukin 1 receptor accessory protein like 2	-0.228	0.0244
205635_at	*NPHP4*	kalirin, RhoGEF kinase	-0.387	0.0245
212165_at	*SAMHD1*	transmembrane protein 183B///transmembrane protein 183A	-0.560	0.0245
1554612_at	*SLC45A2*	RUN and cysteine rich domain containing beclin 1 interacting protein	-0.237	0.0245
205362_s_at	*SEPT1*	prefoldin subunit 4	-0.491	0.0246
229953_x_at	*LTBP1*	LCA5, lebercilin	-0.249	0.0248
201568_at	*ZNF613*	ubiquinol-cytochrome c reductase complex III subunit VII	-0.350	0.0248
1563753_at	*USP9Y*	uncharacterized LOC149684///bactericidal/permeability-increasing protein	-0.286	0.0249
217630_at	*COL7A1*	angel homolog 2	-0.307	0.0252
222428_s_at	*PTGDS*	leucyl-tRNA synthetase	-0.531	0.0252
230236_at	*COBL*	testis development related 1 (non-protein coding)	-0.391	0.0253
240407_at	*TRAPPC3*	uncharacterized LOC100126784	-0.284	0.0254
1555267_at	*APOL2*	glutamate ionotropic receptor delta type subunit 1	-0.345	0.0255

1562841_at	*LINC01149*	uncharacterized LOC339666	-0.283	0.0256
1561732_at	*SDCCAG8*	uncharacterized LOC101929181	-0.202	0.0257
218563_at	*PRDX2*	NADH:ubiquinone oxidoreductase subunit A3	-0.327	0.0261
232721_at	*DNAH14*	tripartite motif containing 55	-0.320	0.0261
226051_at	*PRTG*	selenoprotein M	-0.982	0.0261
1552979_at	*LINC00537*	long intergenic non-protein coding RNA 471	-0.222	0.0262
206657_s_at	*CLASP2*	myogenic differentiation 1	-0.736	0.0263
1559674_at	*ABI2*	zinc finger protein 333	-0.309	0.0263
216257_at	*C9orf131*	serpin family B member 13	-0.213	0.0264
1553727_at	*AOC3*	beta-1,4-N-acetyl-galactosaminyltransferase 3	-0.317	0.0265
1561469_at	*MMP11*	uncharacterized LOC101928865	-0.233	0.0266
207175_at	*PLXNC1*	adiponectin, C1Q and collagen domain containing	-1.132	0.0266
225942_at	*C11orf21*	neurolysin	-0.340	0.0266
1566665_at	*HRG*	coiled-coil domain containing 168	-0.226	0.0267
237270_at	*HBA2///HBA1*	uncharacterized LOC100505710	-0.347	0.0268
242674_at	*GSK3B*	eukaryotic translation initiation factor 4E	-0.285	0.0270
242510_at	*PMEPA1*	uncharacterized LOC101928324	-0.229	0.0271
230364_at	*ADAMTS12*	choline phosphotransferase 1	-0.478	0.0271
229306_at	*PROS1*	C2 calcium dependent domain containing 4B	-0.599	0.0273
202249_s_at	*CACNB4*	DDB1 and CUL4 associated factor 8	-0.235	0.0273
220619_at	*EDNRA*	chromodomain helicase DNA binding protein 7	-0.223	0.0273
206808_at	*FAM208A*	heterogeneous nuclear ribonucleoprotein A3 pseudogene 1	-0.189	0.0274
214169_at	*HLA-E*	Sad1 and UNC84 domain containing 1	-0.491	0.0276
1569240_at	*FAM65C*	zinc finger protein 93	-0.279	0.0277
242545_at	*PLEKHA4*	tubulin tyrosine ligase like 11	-0.243	0.0278
218690_at	*ABHD15*	PDZ and LIM domain 4	-0.342	0.0279
215357_s_at	*CD99*	DNA polymerase delta interacting protein 3	-0.199	0.0280
224004_at	*ASB4*	zinc finger protein 226	-0.239	0.0280
221758_at	*PIK3IP1*	armadillo repeat containing 6	-0.315	0.0281
207590_s_at	*PMS1*	centromere protein I	-0.360	0.0282
216344_at	*ZNF576*	nephronophthisis 4	-0.259	0.0282
207717_s_at	*AKAP11*	plakophilin 2	-1.197	0.0283
221644_s_at	*FANCB*	solute carrier family 45 member 2	-0.181	0.0283
218099_at	*DKFZP586I1420*	testis expressed 2	-0.393	0.0285
219851_at	*PITHD1*	zinc finger protein 613	-0.203	0.0286
207954_at	*ZNF473*	GATA binding protein 2	-0.316	0.0287
215195_at	*MAP4*	protein kinase C alpha	-0.595	0.0288
212995_x_at	*ZNF496*	mitotic spindle organizing protein 2A///mitotic spindle organizing protein 2B///phosphoglycerate dehydrogenase	-0.402	0.0289
37793_r_at	*BRWD3*	RAD51 paralog D	-0.543	0.0289
229446_at	*GLIPR1*	uncharacterized LOC101929977	-0.437	0.0291

208437_at	*LOC101927768*	chloride voltage-gated channel 1	-0.211	0.0293
241478_at	*MAGI3*	MICAL like 2	-0.374	0.0293
213050_at	*CCL21*	cordon-bleu WH2 repeat protein	-0.908	0.0293
1557059_at	*FOXO3*	NIFK antisense RNA 1	-0.339	0.0293
1565936_a_at	*AXL*	LIM domain only 3	-0.376	0.0294
222018_at	*P3H3*	nascent polypeptide-associated complex alpha subunit	-0.515	0.0295
1569779_at	*MALAT1*	long intergenic non-protein coding RNA 1149	-0.273	0.0295
239684_at	*TRAF5*	transient receptor potential cation channel subfamily M member 3	-0.234	0.0296
243772_at	*PLD3*	serologically defined colon cancer antigen 8	-0.431	0.0296
234840_s_at	*TBC1D10C*	olfactory receptor family 5 subfamily V member 1	-0.186	0.0296
228747_at	*LPXN*	Sec61 translocon alpha 2 subunit	-0.489	0.0297
228626_at	*ENG*	ubiquitin related modifier 1	-0.292	0.0298
215017_s_at	*TRHDE*	formin binding protein 1 like	-0.620	0.0298
211658_at	*LINC00032*	peroxiredoxin 2	-0.340	0.0298
242283_at	*CCDC77*	dynein axonemal heavy chain 14	-0.684	0.0298
222427_s_at	*SZT2*	leucyl-tRNA synthetase	-0.457	0.0299
237461_at	*MAK*	NLR family pyrin domain containing 7	-0.277	0.0299
223682_s_at	*LCK*	eukaryotic translation initiation factor 1A domain containing	-0.358	0.0299
214225_at	*ZNF806///ZNF285*	peptidylprolyl cis/trans isomerase, NIMA-interacting 4	-0.368	0.0300
230985_at	*EPS8L2*	chromosome 9 open reading frame 131	-0.243	0.0302
1556894_at	*LOC100131170*	5'-nucleotidase domain containing 2	-0.194	0.0303
204894_s_at	*ADRA2B*	amine oxidase, copper containing 3	-0.712	0.0303
1556839_s_at	*COL12A1*	uncharacterized LOC100289090	-0.535	0.0304
1563614_at	*HLA-F*	MDM2 binding protein	-0.279	0.0304
1560207_at	*ZNF831*	microRNA 3610///RAD21 antisense RNA 1	-0.273	0.0304
209377_s_at	*PGLS*	high mobility group nucleosomal binding domain 3	-0.355	0.0305
206226_at	*MMP19*	histidine rich glycoprotein	-0.198	0.0306
238944_at	*ALPK2*	uncharacterized LOC100505715	-0.807	0.0306
237238_at	*ZNF99*	WW and C2 domain containing 1	-0.493	0.0306
214414_x_at	*WFIKKN1*	hemoglobin subunit alpha 2///hemoglobin subunit alpha 1	-1.561	0.0307
206556_at	*MRVI1*	clusterin like 1	-0.643	0.0308
206817_x_at	*GRIK5*	CUGBP, Elav-like family member 3	-0.308	0.0310
240562_at	*RAP2A*	glycogen synthase kinase 3 beta	-0.248	0.0310
89948_at	*NCOR2*	PDX1 C-terminal inhibiting factor 1	-0.184	0.0312
224065_at	*WWC2///CLDN22*	homeodomain interacting protein kinase 2	-0.200	0.0312
243244_at	*PDE10A*	calcium voltage-gated channel auxiliary subunit beta 4	-0.180	0.0313

234682_at	*DOCK8*	BTB domain containing 9	-0.246	0.0315
1554714_at	*MEOX1*	RELT like 1	-0.292	0.0318
239800_at	*HAUS5*	uridine monophosphate synthetase	-0.278	0.0318
1562950_at	*NCAM1*	uncharacterized LOC101927210	-0.426	0.0320
206564_at	*HOXA-AS3*	opioid related nociceptin receptor 1	-0.194	0.0321
217585_at	*MMP23A///MMP23B*	nebulette	-0.722	0.0323
1556294_at	*RNF186*	FXYD domain containing ion transport regulator 2	-0.216	0.0323
243290_at	*HOTAIRM1*	WW and C2 domain containing 1	-0.311	0.0323
211576_s_at	*IGFBP4*	solute carrier family 19 member 1	-0.549	0.0324
208481_at	*GIMAP1*	ankyrin repeat and SOCS box containing 4	-0.198	0.0324
1569974_x_at	*KRT1*	septin 7 pseudogene 2	-0.254	0.0325
219220_x_at	*TBX2*	mitochondrial ribosomal protein S22	-0.404	0.0325
1555632_at	*GMPS*	phosphoinositide-3-kinase interacting protein 1	-0.265	0.0325
217042_at	*CLDN2*	retinol dehydrogenase 11 (all-trans/9-cis/11-cis)	-0.256	0.0326
1554742_at	*SYTL5*	PMS1 homolog 1, mismatch repair system component	-0.244	0.0327
219089_s_at	*SLIT3*	zinc finger protein 576	-0.285	0.0327
222056_s_at	*A2MP1*	fumarylacetoacetate hydrolase domain containing 2A	-0.401	0.0327
243597_at	*THAP8*	Fanconi anemia complementation group B	-0.300	0.0328
229349_at	*RAB3D*	lin-28 homolog B	-1.300	0.0330
1554676_at	*VAMP5*	serglycin	-0.184	0.0331
233605_x_at	*GNGT1*	heterogeneous nuclear ribonucleoprotein M	-0.227	0.0332
1563145_at	*LAMC3*	long intergenic non-protein coding RNA 301	-0.182	0.0332
239663_x_at	*QSER1*	La ribonucleoprotein domain family member 1	-0.273	0.0336
213130_at	*SEC23A*	zinc finger protein 473	-0.329	0.0337
240268_at	*CEP57L1*	uncharacterized LOC440117	-0.365	0.0337
1557616_at	*PPFIA2*	zinc finger protein 496	-0.293	0.0338
218365_s_at	*FBXO28*	aspartyl-tRNA synthetase 2, mitochondrial	-0.296	0.0339
209122_at	*NKD2*	perilipin 2	-0.455	0.0340
1559620_at	*FCMR*	uncharacterized LOC101927768	-0.249	0.0340
230409_at	*PHACTR2*	membrane associated guanylate kinase, WW and PDZ domain containing 3	-0.322	0.0341
231548_at	*INPP4A*	forkhead box O3	-0.398	0.0342
239181_at	*LINC00852*	cytochrome P450 family 2 subfamily R member 1	-0.208	0.0343
222579_at	*FLI1*	ubiquitin like modifier activating enzyme 5	-0.482	0.0343
228582_x_at	*IPO9-AS1*	metastasis associated lung adenocarcinoma transcript 1 (non-protein coding)	-0.762	0.0344
241072_s_at	*MXRA7*	insulin like growth factor 1	-0.280	0.0345
219937_at	*PLEKHH1*	thyrotropin releasing hormone degrading enzyme	-0.348	0.0348

228300_at	*ATN1*	family with sequence similarity 187 member A///coiled-coil domain containing 103	-0.209	0.0348
1559292_s_at	*LINC00965*	long intergenic non-protein coding RNA 32	-0.252	0.0348
224521_s_at	*WISP2*	coiled-coil domain containing 77	-0.452	0.0349
1555315_a_at	*LOC100653049 ///KRT34*	male germ cell associated kinase	-0.261	0.0350
223312_at	*NECAP2*	protease associated domain containing 1	-0.381	0.0351
243136_at	*C18orf61*	GINS complex subunit 2	-0.239	0.0351
1552940_at	*AKAP14*	transmembrane epididymal protein 1	-0.224	0.0352
236328_at	*HIST1H4L*	zinc finger protein 806///zinc finger protein 285	-0.609	0.0352
1559403_at	*TECTA*	HMGN3 antisense RNA 1	-0.236	0.0353
235806_at	*RIMBP2*	ArfGAP with RhoGAP domain, ankyrin repeat and PH domain 3	-0.228	0.0353
234272_at	*CFHR1///CFH*	uncharacterized LOC100653005	-0.218	0.0354
222085_at	*TWIST1*	family with sequence similarity 174 member B	-0.411	0.0354
240689_at	*TAF8*	REC114 meiotic recombination protein	-0.199	0.0355
235467_s_at	*IL16*	potassium voltage-gated channel subfamily C member 4	-0.672	0.0356
1554316_at	*WIPF3*	6-phosphogluconolactonase	-0.252	0.0358
1557091_at	*SNX20*	MEF2 activating motif and SAP domain containing transcriptional regulator	-0.558	0.0358
1553883_at	*NDRG3*	zinc finger protein 99	-0.207	0.0360
1552811_at	*ELP4*	WAP, follistatin/kazal, immunoglobulin, kunitz and netrin domain containing 1	-0.197	0.0360
211699_x_at	*TMEM209*	hemoglobin subunit alpha 2///hemoglobin subunit alpha 1	-1.144	0.0361
1569090_x_at	*SLC7A6*	long intergenic non-protein coding RNA 957	-0.258	0.0363
1553168_at	*TRPV2*	glutamate ionotropic receptor kainate type subunit 5	-0.242	0.0363
1558622_a_at	*IFITM2///IFITM1*	zinc finger protein 548	-0.202	0.0363
241405_at	*ZNF469*	TOB1 antisense RNA 1	-0.238	0.0363
226647_at	*MEX3C*	transmembrane protein 25	-0.383	0.0364
1568843_at	*SEC62*	tubulin tyrosine ligase like 13, pseudogene	-0.205	0.0366
224805_s_at	*TRAF3IP3*	family with sequence similarity 219 member B	-0.184	0.0367
220613_s_at	*TSGA10IP*	synaptotagmin like 2	-0.215	0.0369
231015_at	*FGFR1*	Kruppel like factor 15	-0.695	0.0370
211170_s_at	*TRIM38*	phosphodiesterase 10A	-0.265	0.0371
228059_x_at	*TPCN2*	mitochondrial ribosomal protein S22	-0.310	0.0371
238628_s_at	*PIK3C2A*	trafficking protein particle complex 2 like	-0.274	0.0371
1554145_a_at	*LOC728805*	protein phosphatase 1 regulatory subunit 21	-0.253	0.0373
211745_x_at	*CXorf65*	hemoglobin subunit alpha 2///hemoglobin subunit alpha 1	-1.187	0.0373
235946_at	*DGCR2*	RIB43A domain with coiled-coils 1	-0.368	0.0374
1569486_at	*PHF11*	uncharacterized LOC101928401	-0.274	0.0374

228966_at	*FEM1A*	pantothenate kinase 2	-0.237	0.0374
228836_at	*LOC100507560*	solute carrier family 25 member 35	-0.287	0.0374
213053_at	*COX7A2*	HAUS augmin like complex subunit 5	-0.332	0.0375
206213_at	*SNX13*	Wnt family member 10B	-0.344	0.0376
1556717_at	*TLCD2*	microRNA 194-2	-0.288	0.0376
214952_at	*LOC101928668*	neural cell adhesion molecule 1	-0.205	0.0376
231524_at	*C2orf88*	potassium voltage-gated channel subfamily A member regulatory beta subunit 1	-0.259	0.0378
242771_at	*UQCR11*	titin	-0.237	0.0379
219739_at	*VWA1*	ring finger protein 186	-0.322	0.0380
229418_at	*HIST2H2BE*	family with sequence similarity 222 member B	-0.232	0.0381
236648_at	*LINC01545*	guanine monophosphate synthase	-0.364	0.0383
223509_at	*FBN1*	claudin 2	-0.215	0.0384
1553033_at	*TFAP2A*	synaptotagmin like 5	-0.177	0.0384
236394_at	*MGC32805*	alpha-2-macroglobulin pseudogene 1	-0.304	0.0385
230117_at	*MX2*	V-set and transmembrane domain containing 2A	-0.290	0.0385
228500_at	*TMSB4Y*	THAP domain containing 8	-0.355	0.0386
231095_at	*SLC38A7*	uncharacterized LOC101928045	-0.510	0.0386
221169_s_at	*EMP1*	histamine receptor H4	-0.275	0.0387
1565659_at	*CLN8*	fucosyltransferase 6	-0.246	0.0388
226078_at	*FOXI1*	RNA pseudouridylate synthase domain containing 1	-0.283	0.0389
216660_at	*FOXF2*	myosin VIIB	-0.183	0.0390
209458_x_at	*SCAF1*	hemoglobin subunit alpha 2///hemoglobin subunit alpha 1	-1.241	0.0390
204018_x_at	*FAM230C*	hemoglobin subunit alpha 2///hemoglobin subunit alpha 1	-1.191	0.0390
205568_at	*JAM2*	aquaporin 9	-0.511	0.0392
1570422_at	*KLHL36*	syntrophin gamma 2	-0.226	0.0392
235184_at	*C2orf27A*	AE binding protein 2	-0.237	0.0392
229675_at	*ATG12*	MYC induced nuclear antigen	-0.233	0.0392
239183_at	*CDKL1*	angiopoietin like 1	-0.327	0.0394
219705_at	*ANXA2P1*	glutamine and serine rich 1	-0.273	0.0394
208520_at	*ATXN7*	olfactory receptor family 10 subfamily H member 3	-0.279	0.0394
243118_at	*PSMB8*	centrosomal protein 57 like 1	-0.426	0.0395
207220_at	*ETS1*	ADP-ribosyltransferase 4 (Dombrock blood group)	-0.239	0.0399
206973_at	*MYL4*	PTPRF interacting protein alpha 2	-0.443	0.0399

224212_s_at	*MOB1A*	protocadherin alpha 1///protocadherin alpha 2///protocadherin alpha 3///protocadherin alpha 4///protocadherin alpha 5///protocadherin alpha 6///protocadherin alpha 7///protocadherin alpha 8///protocadherin alpha 10///protocadherin alpha 11///protocadherin alpha 12///protocadherin alpha 13///protocadherin alpha subfamily C, 1///protocadherin alpha subfamily C, 2///protocadherin alpha 9	-0.774	0.0402
218275_at	*SPTBN1*	solute carrier family 25 member 10	-0.481	0.0402
231488_at	*COL18A1*	orthopedia homeobox	-0.257	0.0402
235006_at	*METTL7A*	CDKN2A interacting protein N-terminal like	-0.392	0.0402
1569616_at	*PKHD1*	geminin coiled-coil domain containing	-0.239	0.0408
233567_at	*LRRFIP1*	IPO9 antisense RNA 1	-0.261	0.0409
202652_at	*RNF207*	amyloid beta precursor protein binding family B member 1	-0.351	0.0410
225726_s_at	*TMEM174*	pleckstrin homology, MyTH4 and FERM domain containing H1	-0.852	0.0413
210518_at	*TBX15*	cadherin 8	-0.206	0.0415
206969_at	*PTGER4*	keratin, type I cuticular Ha4///keratin 34	-1.019	0.0416
243721_at	*SERPING1*	uncharacterized LOC497259	-0.201	0.0419
237281_at	*CHKB*	A-kinase anchoring protein 14	-0.198	0.0419
214562_at	*CC2D2A*	histone cluster 1, H4l	-0.202	0.0419
219702_at	*SLAMF8*	placenta specific 1	-0.280	0.0420
236904_x_at	*PGPEP1*	tectorin alpha	-0.237	0.0421
226616_s_at	*MVP*	NADH:ubiquinone oxidoreductase subunit V3	-0.557	0.0421
223945_x_at	*IL2RG*	retinitis pigmentosa 9 pseudogene	-0.266	0.0423
241771_at	*LOC100130950*	RIMS binding protein 2	-0.311	0.0423
224782_at	*REST*	zinc finger matrin-type 2	-0.312	0.0424
1562348_at	*SMIM14*	long intergenic non-protein coding RNA 664	-0.253	0.0425

Supplementary Table S8. Comparison of the 7 IGF2BP3 candidate targets with IGF2BP3 targets identified in published studies

	Overlap with IGF2P3 targets identified in previous studies*							
**Data Source**	**Hanniford et al.**	**Ennajdaoui et al.**				**Conway et al.**	**Hafner et al.**	**Count**
Condition	Endogenous IGF2BP3	Endogenous IGF2BP3		iCLIP IGF2BP3		IGF2BP3	FLAG IGF2BP3	**(no. of experiments)**
Cell Line	WM278	PL45	PANC1	PL45	PANC1	H9 hESC	293T	
ZMIZ2	yes	yes	yes	no	no	no	yes	4
ABHD2	no	no	no	no	no	no	no	0
FMNL3	yes	no	no	no	no	no	no	1
TUG1	yes	yes	yes	no	no	no	yes	4
UBXN2B	yes	yes	yes	no	no	yes	no	4
MUC1	yes	yes	no	no	no	no	no	2
LPCAT3	yes	yes	yes	no	no	no	yes	4

* Yes and no refers to present or absent in the respective study

Hanniford et al. Cancer Cell, 37: 55-70 (2020)

Ennajdaoui et al. Cell Rep. 15(9): 1876-1883 (2016)

Conway et al. Cell Rep. 15(3):666-679 (2016)

Hafner et al. Cell 141: 129-141 (2010)

Supplementary Table S9. Primary and second antibodies used for immuoblotting

Antibody	Company	Catalog no.	Dilution
BRD2 (BL-167-2A2)	Thermo Fisher Scientific	A700-008	1:1000
BRD3	Thermo Fisher Scientific	A302-368A	1:1000
BRD4 (E2A7X)	Cell Signaling Technology	13440S	1:1000
IGF2BP3/IMP3 (D6U2N)	Cell Signaling Technology	57145S	1:1000
ZMIZ1 (E2X3X)	Cell Signaling Technology	89500S	1:1000
HMGA1 (D6A4)	Cell Signaling Technology	7777S	1:1000
GAPDH (14C10)	Cell Signaling Technology	2118S	1:1000
alpha Tubulin (B-7)	Santa Cruz Biotechnology	sc-5286	1:1000
MCPyV large T-antigen Antibody (CM2B4)	Santa Cruz Biotechnology	sc-136172	1:1000
Cleaved PARP1 antibody (E51)	abcam	ab32064	1:1000
IRDye® 680RD Goat anti-Rabbit IgG Secondary Antibody	LI-COR	D30228-15	1:5000
IRDye® 800CW Goat anti-Mouse IgG Secondary Antibody	LI-COR	D20802-25	1:5000
Goat anti-Rabbit IgG (H+L) Secondary Antibody, HRP	Thermo Fisher Scientific	65-6120	1:2000
Goat anti-Mouse IgG (H+L) Secondary Antibody, HRP	Thermo Fisher Scientific	62-6520	1:2000
