# Supplementary material for: IGF2BP3 As a Prognostic Biomarker and Regulator of Metastasis in Merkel Cell Carcinoma
Source: JID Innov. 2025 Feb 12;5(3):100355. doi: 10.1016/j.xjidi.2025.100355 (PMC11951866; doi:10.1016/j.xjidi.2025.100355)
Supplement: Supplementary Figures 1-15 [file mmc2.pdf]

Supplementary Figures

IGF2BP3 as a Prognostic Biomarker and Regulator of Metastasis in Merkel Cell Carcinoma

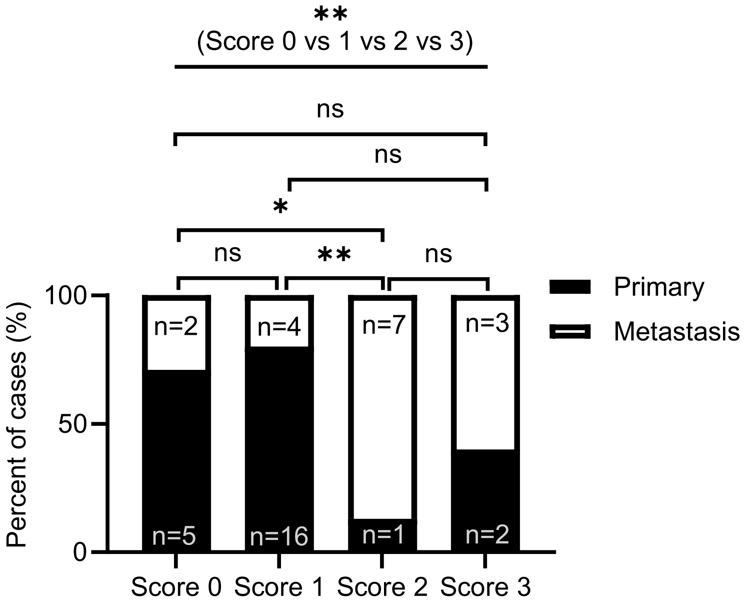

Supplementary Figure S1. Association of IGF2BP3 expression with MCC metastasis. Comparison of IGF2BP3 IHC intensity score (0, negative; 1, weak; 2, intermediate; 3, strong) between primary tumors and metastases in the Swedish MCC cohort. **P*<0.05, ***P*<0.01 and ns=not significant by Fisher's exact test.

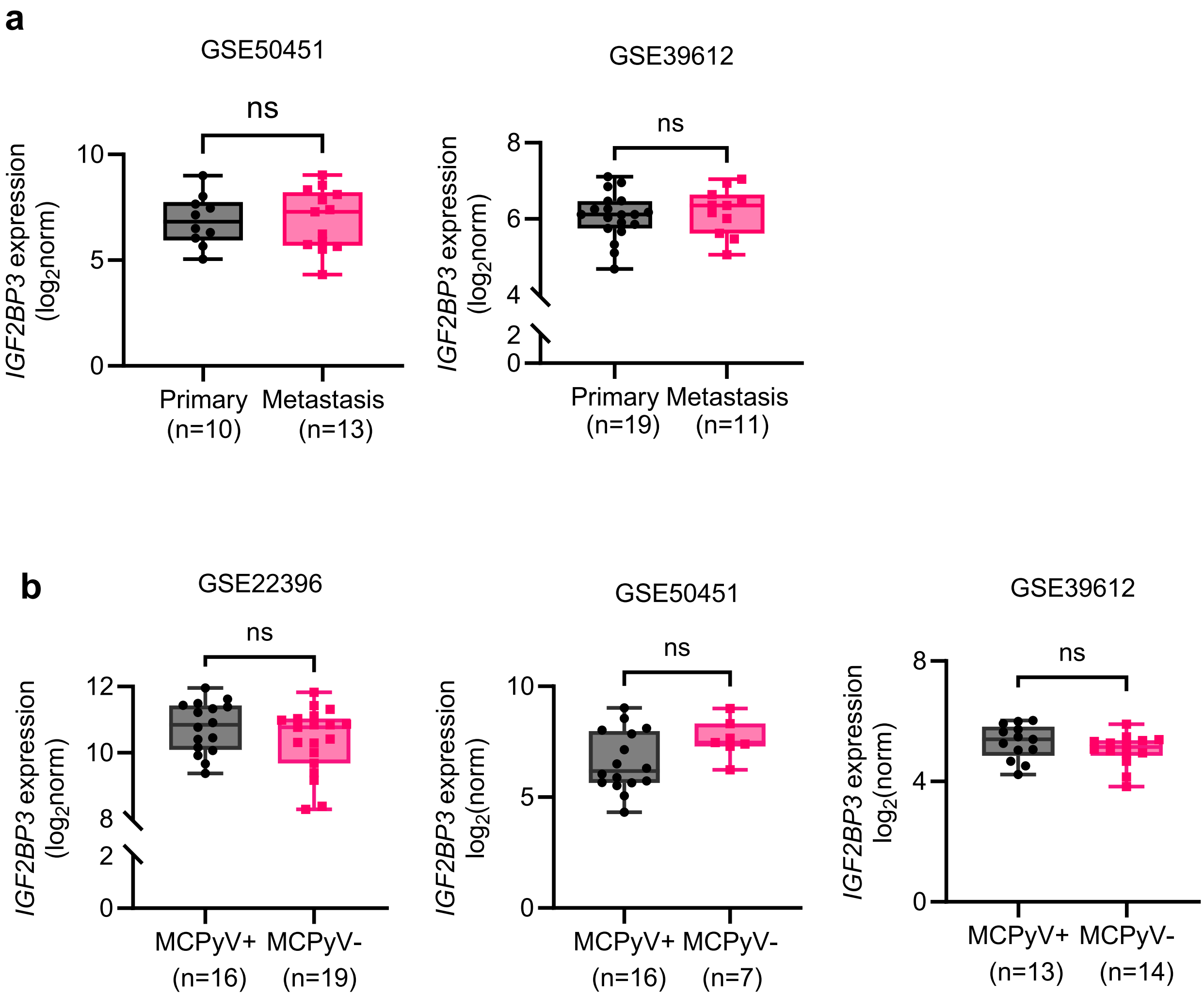

Supplementary Figure S2. Comparison of *IGF2BP3* expression in MCC GEO datasets. **(a)** *IGF2BP3* mRNA expression levels were analyzed from two microarray expression profiles of MCC primary tumors and metastases using data from two Gene Expression Omnibus datasets (GEO accession no. GSE50451 and GSE39612). **(b)** Comparison of *IGF2BP3* mRNA expression levels between virus-positive (MCPyV+) and virus-negative (MCPyV-) MCC tumors across the three GEO datasets. (**a** and **b**) Log_2norm, normalized expression in log_2 scale. The box represents the median and interquartile range (25-75%). Differences between the two groups were analyzed by Mann-Whitney U-test. ns, not significant.

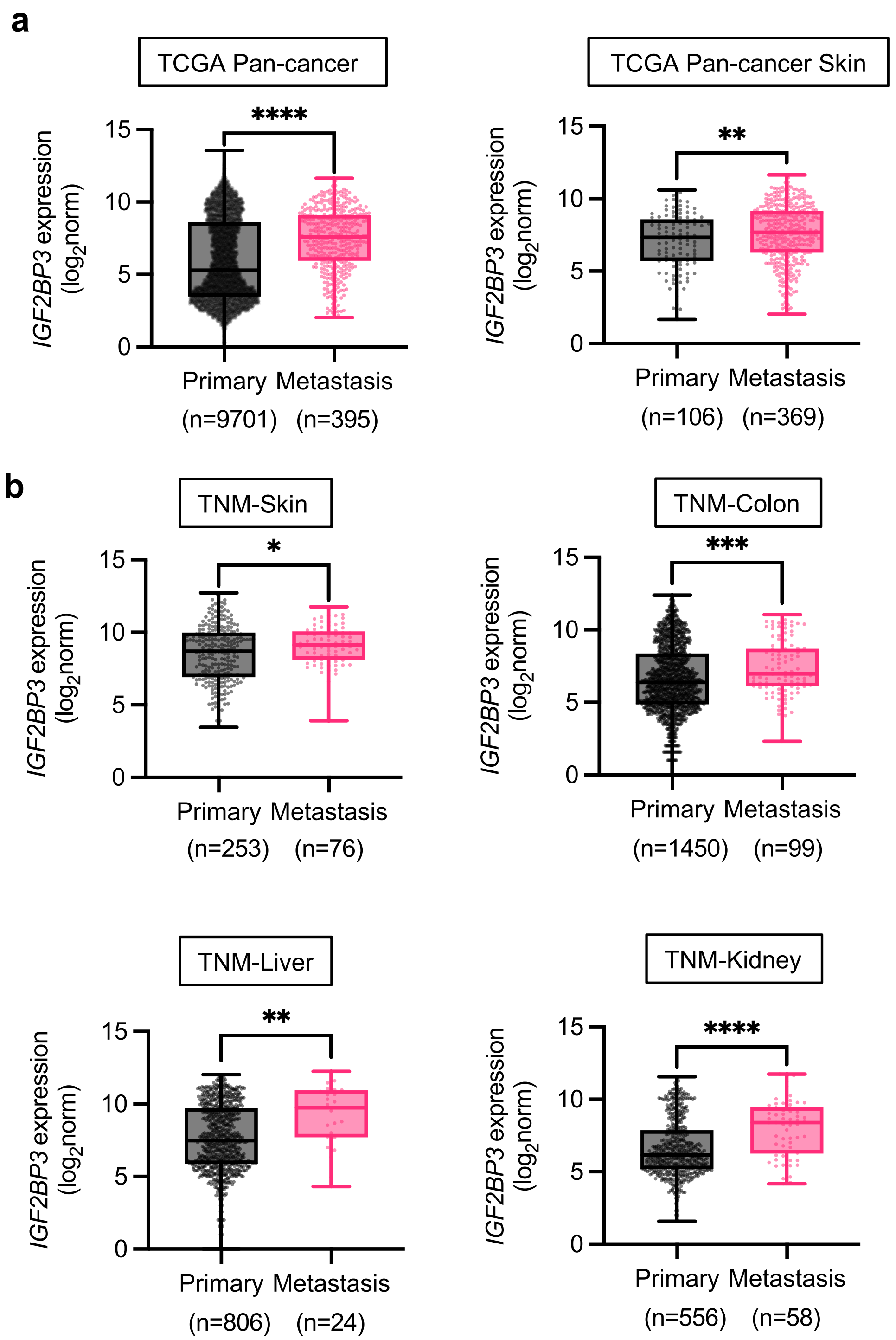

Supplementary Figure S3. Elevated *IGF2BP3* expression level in tumor metastases is common in a variety of cancer types. (**a**) Comparison of *IGF2BP3* mRNA expression between primary tumors and metastases using TCGA Pan-cancer dataset and its skin cancer cohort from UCSC Xena (https://xenabrowser.net/). (**b**) Comparison of *IGF2BP3* expression in metastases and primary tumors in skin, colon, liver and kidney cancers using gene chip data from TNMplot database (https://tnmplot.com/analysis/). (**a** and **b**) Log_2norm, normalized expression in log_2 scale. The box represents the median and interquartile range (25-75%). Statistical analyses were performed using Mann-Whitney U-test. *P<0.05, **P<0.01, ***P<0.001 ****P<0.0001.

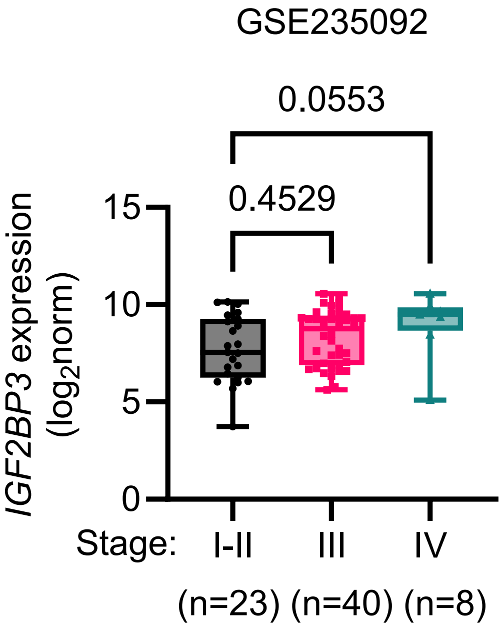

Supplementary Figure S4. Analysis of *IGF2BP3* expression among stages I-II, III and IV in GSE235092. *IGF2BP3* mRNA across stages I-II, III and IV obtained from the GSE235092 dataset. Log_2norm, normalized expression in log_2 scale. The box represents the median and interquartile range (25-75%). **P*<0.05 by Mann-Whitney U-test.

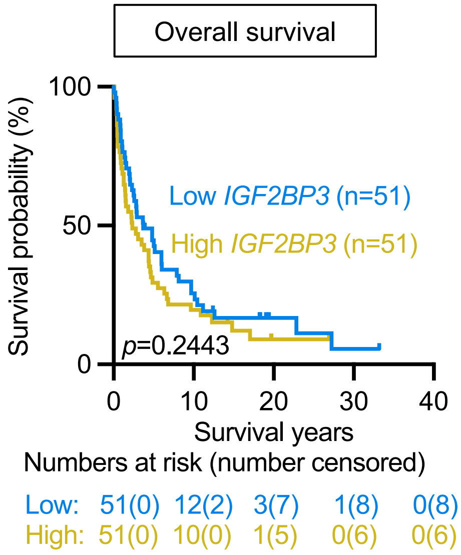

Supplementary Figure S5. Analysis of overall survival in the Finnish MCC cohort for cases with low or high *IGF2BP3* expression. Kaplan-Meier plot comparing overall survival time for cases in the Finnish MCC cohort with low or high IGF2BP3 mRNA levels in their primary tumors. The survival analysis was assessed using log rank test.

a MCC xenograft mouse 1

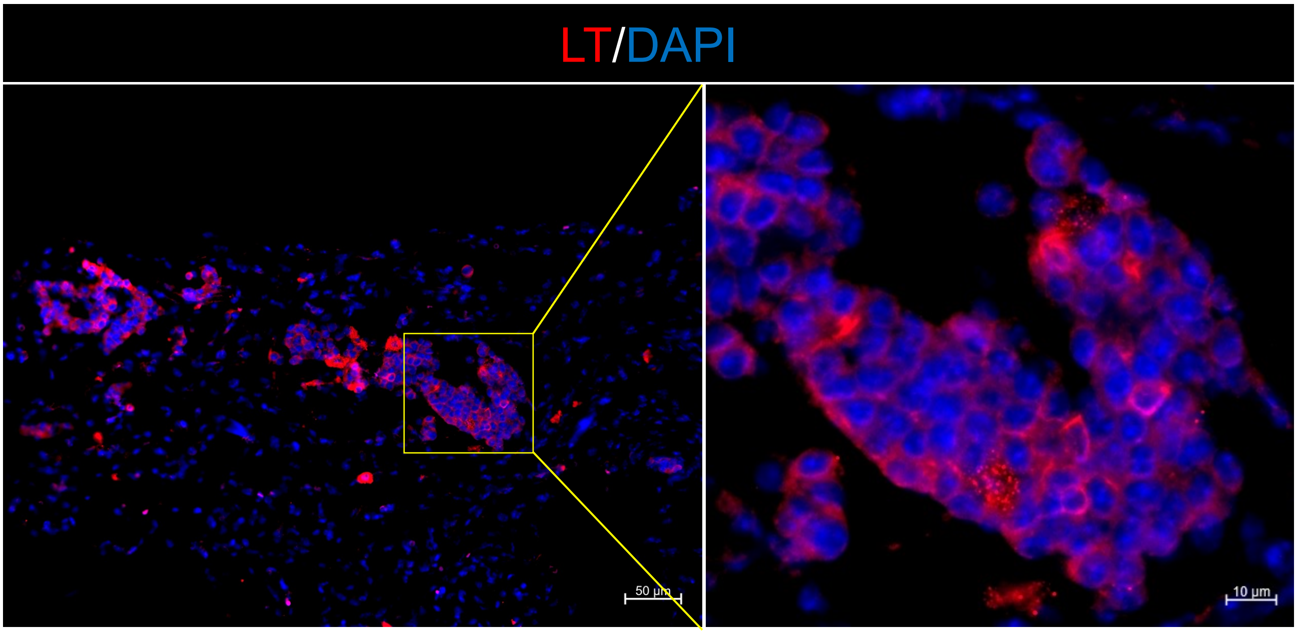

b MCC xenograft mouse 3

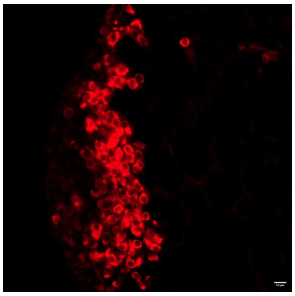

Supplementary Figure S6. Immunofluorescence staining of MCPyV LT in lung metastasis of MCC xenograft model. (**a**) Immunofluorescence of MCPyV large T antigen (LT) (red) in lung metastasis of mouse 1. The left panel shows low magnification (scale bar = 50 µm), highlighting the overall distribution of LT-positive cells. The right panel is a high magnification of the area outlined in yellow, providing detailed visualization of LT-positive cells at a higher resolution (scale bar = 10 µm). Nuclei are counterstained with DAPI (blue). (**b**) Immunofluorescence of MCPyV LT (red) in lung metastasis of mouse 3. The scale bar represents 10 µm.

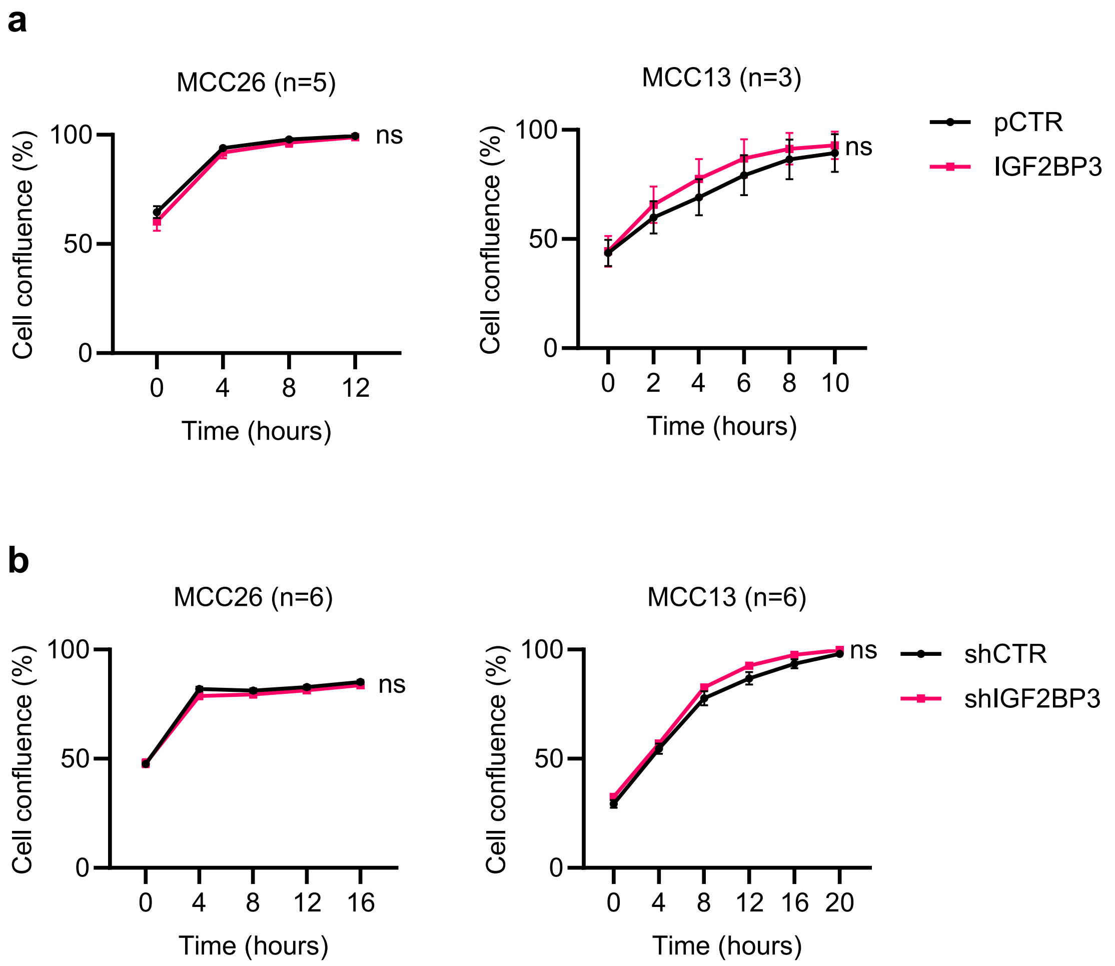

Supplementary Figure S7. Analysis of cell growth upon modulation of IGF2BP3 expression in MCC cell lines. MCC cell lines were transfected with (**a**) empty vector (pCTR) or plasmid expressing IGF2BP3 (IGF2BP3), or (**b**) short hairpin RNA targeting IGF2BP3 (shIGF2B3) or shRNA control (shCTR) for 48 h. Cell growth was monitored via IncuCyte over various time points. Error bars indicate mean ± SEM, statistical analysis was assessed by two-way ANOVA. n = number of biological replicates; ns = not significant.

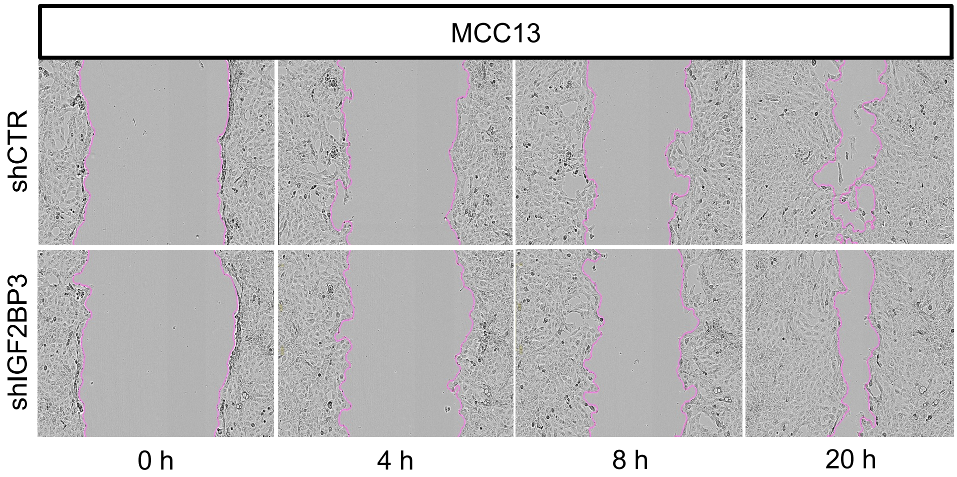

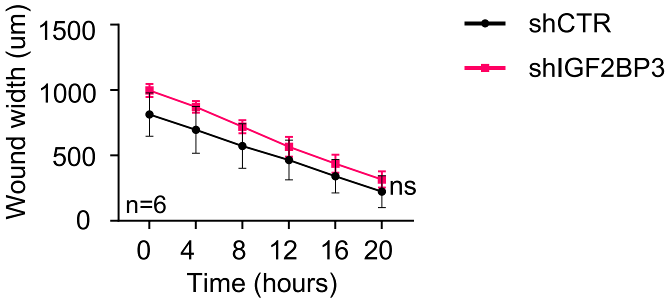

Supplementary Figure S8. Analysis of cell migration upon silencing of IGF2BP3 in MCC13 cell line. MCC13 cells were transfected with short hairpin RNA targeting IGF2BP3 (shIGF2BP3) or shRNA control (shCTR) for 48 h, prior to a scratch was made in the confluent monolayer cells. The gap of the wound was monitored over time using the IncuCyte. Representative images of 4 time points are shown, and the quantification of the wound width is shown below the images. Error bars indicate mean ± SEM from 6 independent replicates. Statistical analysis was performed using two-way ANOVA. ns = not significant .

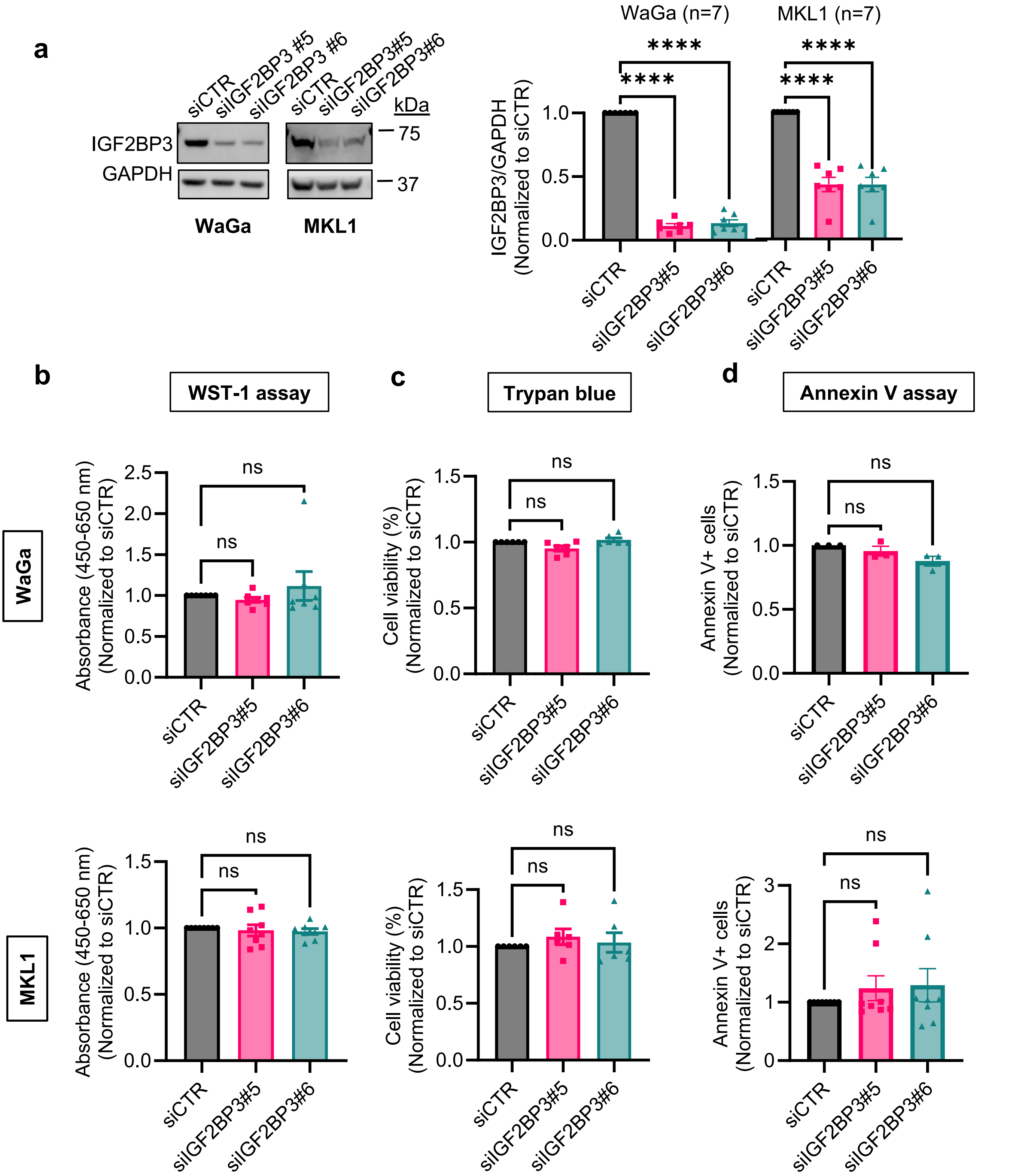

Supplementary Figure S9. Analysis of cell viability upon silencing of IGF2BP3 in MCC cell lines. WaGa and MKL1 cells were transfected with siRNAs targeting IGF2BP3 (siIGF2BP3#5 and siIGF2BP3#6) or siRNA control (siCTR). (**a**) The silencing efficacy was assessed by immunoblotting. (**b**) Cell viability was evaluated using WST-1 assay (WaGa, n=7; MKL1, n=8) and (**c**) trypan blue exclusion assay (WaGa, n=6; MKL1, n=6). (**d**) Annexin V assay (WaGa, n=3; MKL1, n=8) was applied to assess the silencing effect on apoptosis (Annexin V+). Error bars represent mean ± SEM. ns = not significant by one-way ANOVA with post-hoc Dunnett's test.

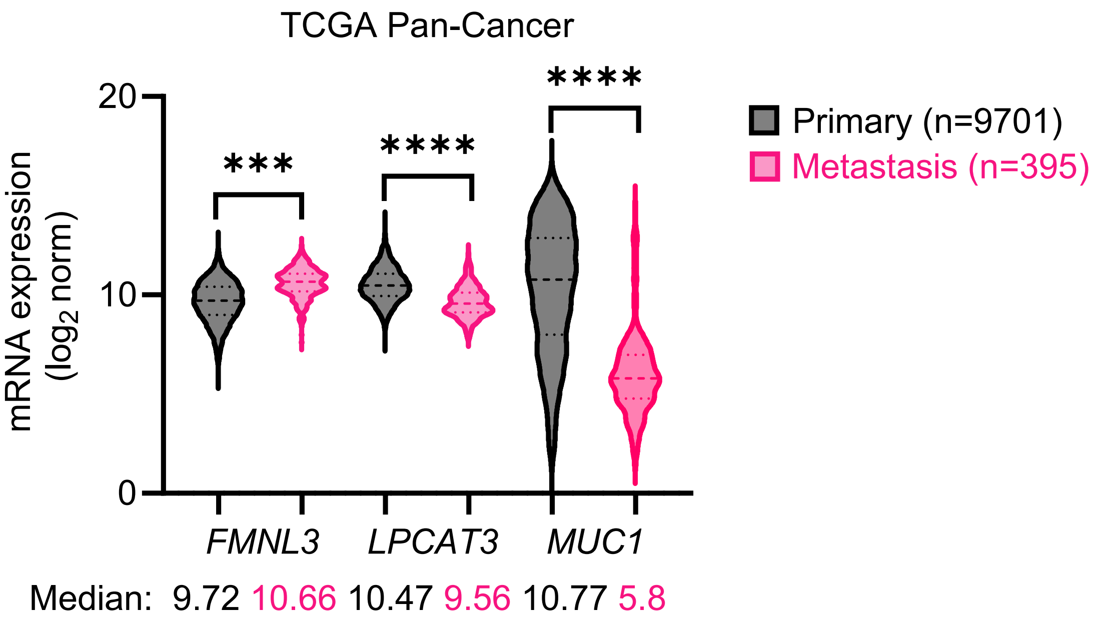

Supplementary Figure S10. Differential expression of IGF2BP3 target genes between primary tumors and metastases in the TCGA pan-cancer cohort. The violin plots illustrate the distribution of expression levels, where the solid line represents the median value, and the dashed lines represent the 25th and 75th percentiles (interquartile range). The width of the violin at each level indicates the density of the data at that expression level. The individual violins are shown for both primary tumors (grey) and metastases (pink). The difference between the two group was evaluated using Mann-Whitney U-test. **P*<0.05, ****P*<0.001, *****P*<0.0001, ns = not significant.

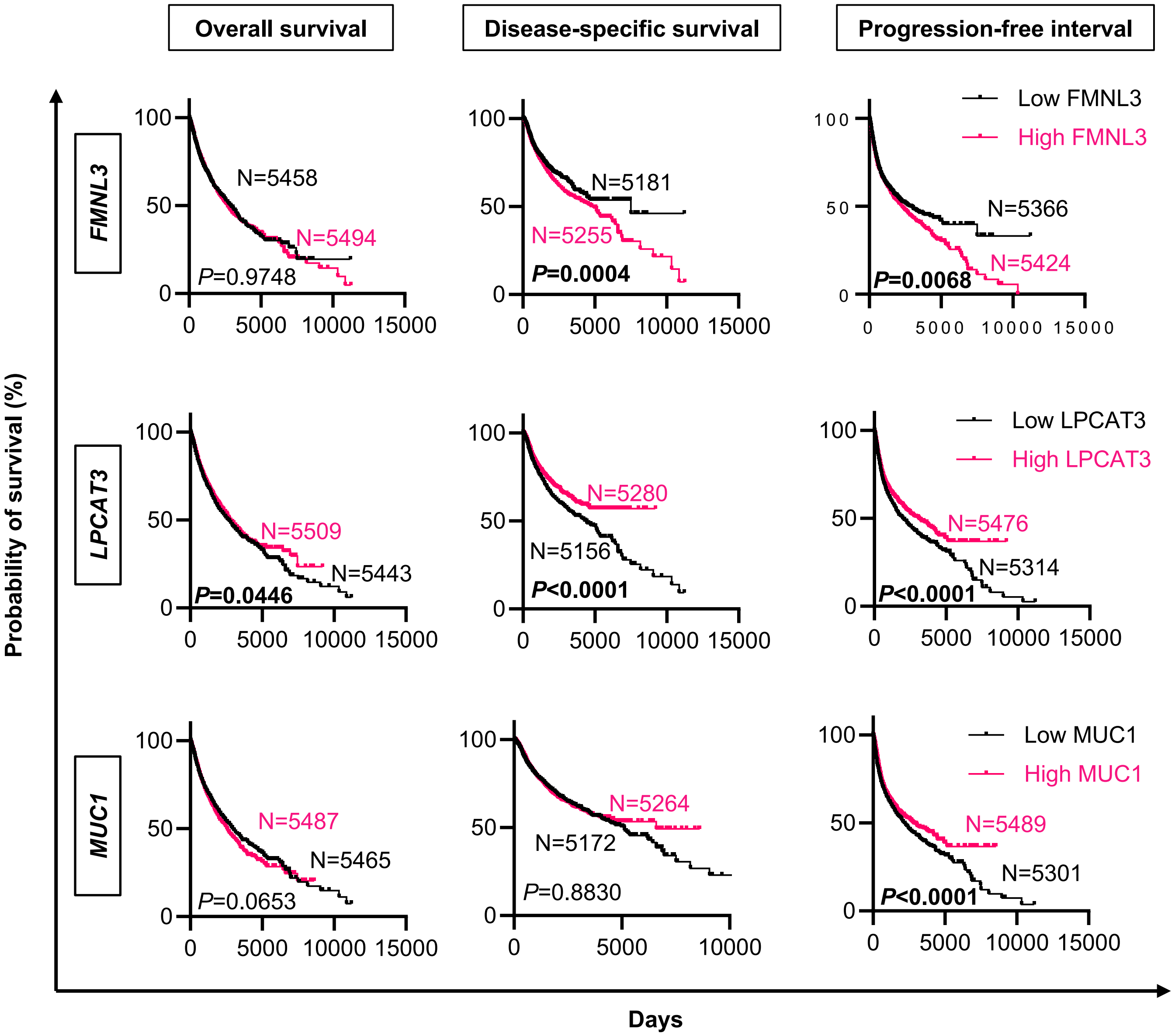

Supplementary Figure S11. Associations between survival and expression levels of candidate IGF2BP3 target genes in the TCGA pan-cancer cohort. Kaplan-Meir plots and log-rank test were used to assess the differences between high/low expression and overall, disease-specific and progression-free survival.

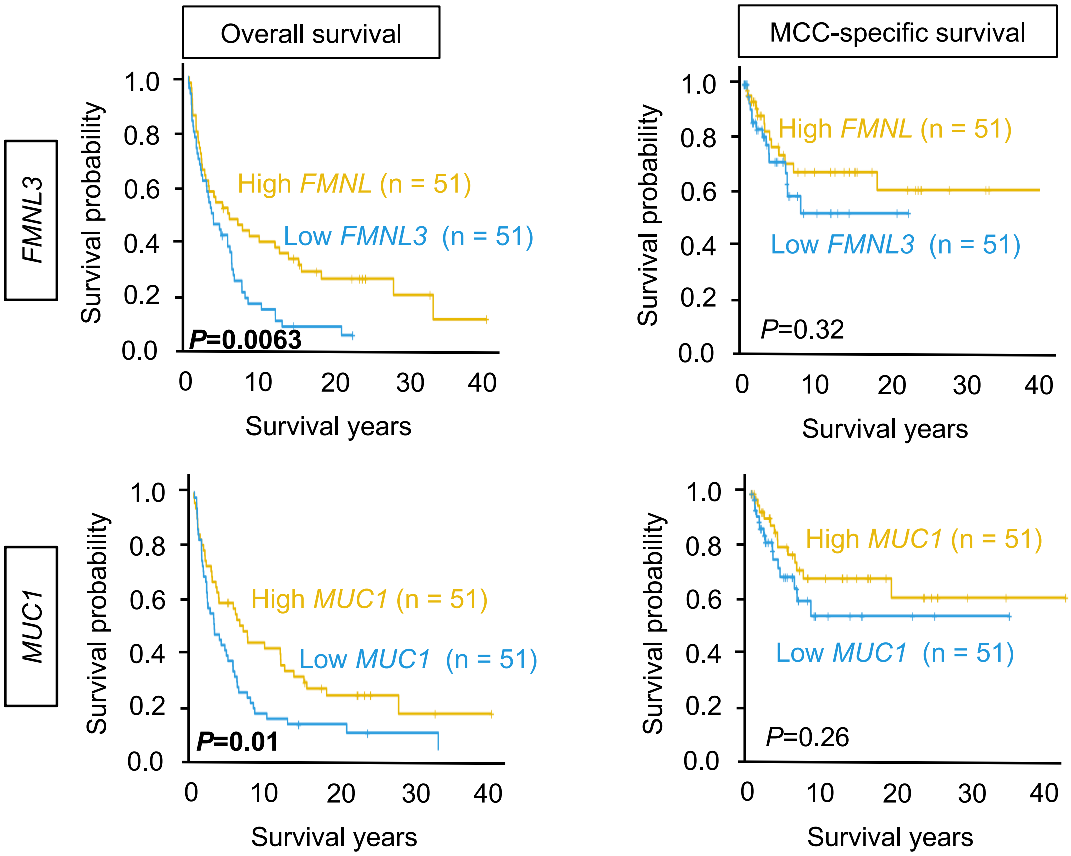

Supplementary Figure S12. Analysis of expression levels of IGF2BP3 targets and survival in the Finnish MCC cohort. Kaplan-Meier plots illustrating associations between overall/MCC-specific survival and high or low expression levels (based on the median expression value) of the target genes. Differences in survival were calculated using log-rank test. Associations with significant *P*-values are highlighted in bold.

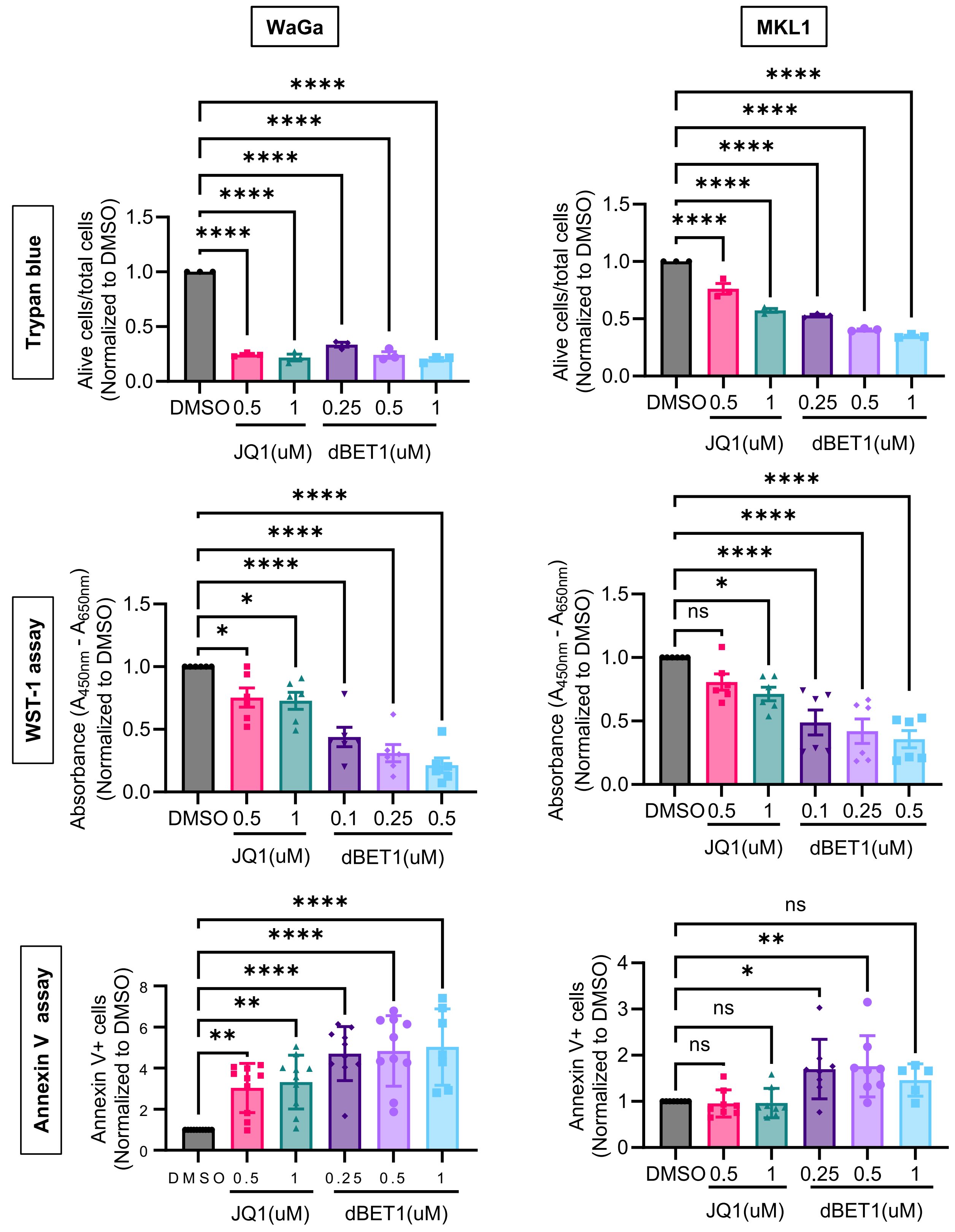

Supplementary Figure S13. Effect of BET inhibitor or degrader treatment on cell viability and apoptosis. WaGa and MKL1 cells were treated with various concentrations of BET inhibitor JQ1 or degrader dBET1. DMSO was used as a vehicle control. Cell viability was assessed by trypan blue (*Top;* WaGa, n=3; MKL1, n=3) and WST-1 (*Middle,* WaGa, n=6; MKL1, n=6) assays, while apoptosis was evaluated by Annexin V assay (*Bottom,* WaGa, n=10; MKL1, n=8). Error bars represent mean ± SEM. *P<0.05, **P<0.01, ****P<0.0001 and ns=not significant by one-way ANOVA with post-hoc Dunnett's test.

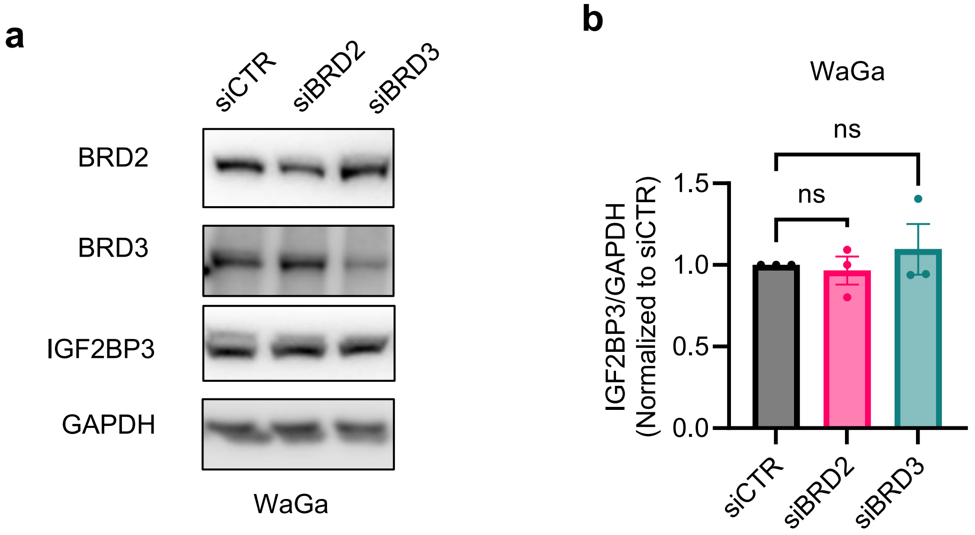

Supplementary Figure S14. Impact of silencing BRD2 or BRD13 on IGF2BP3. (**a**) Immunoblots evaluating the IGF2BP3 expression after silencing BRD2 or BRD3 in WaGa cells. siCTR was used as control. GAPDH, loading control. (**b**) Quantification of IGF2BP3 expression from (**a**). Error bars represent mean ± SEM (n=3). *P*-values were assessed by one-way ANOVA with post-hoc Dunnett's test. ns=not significant.

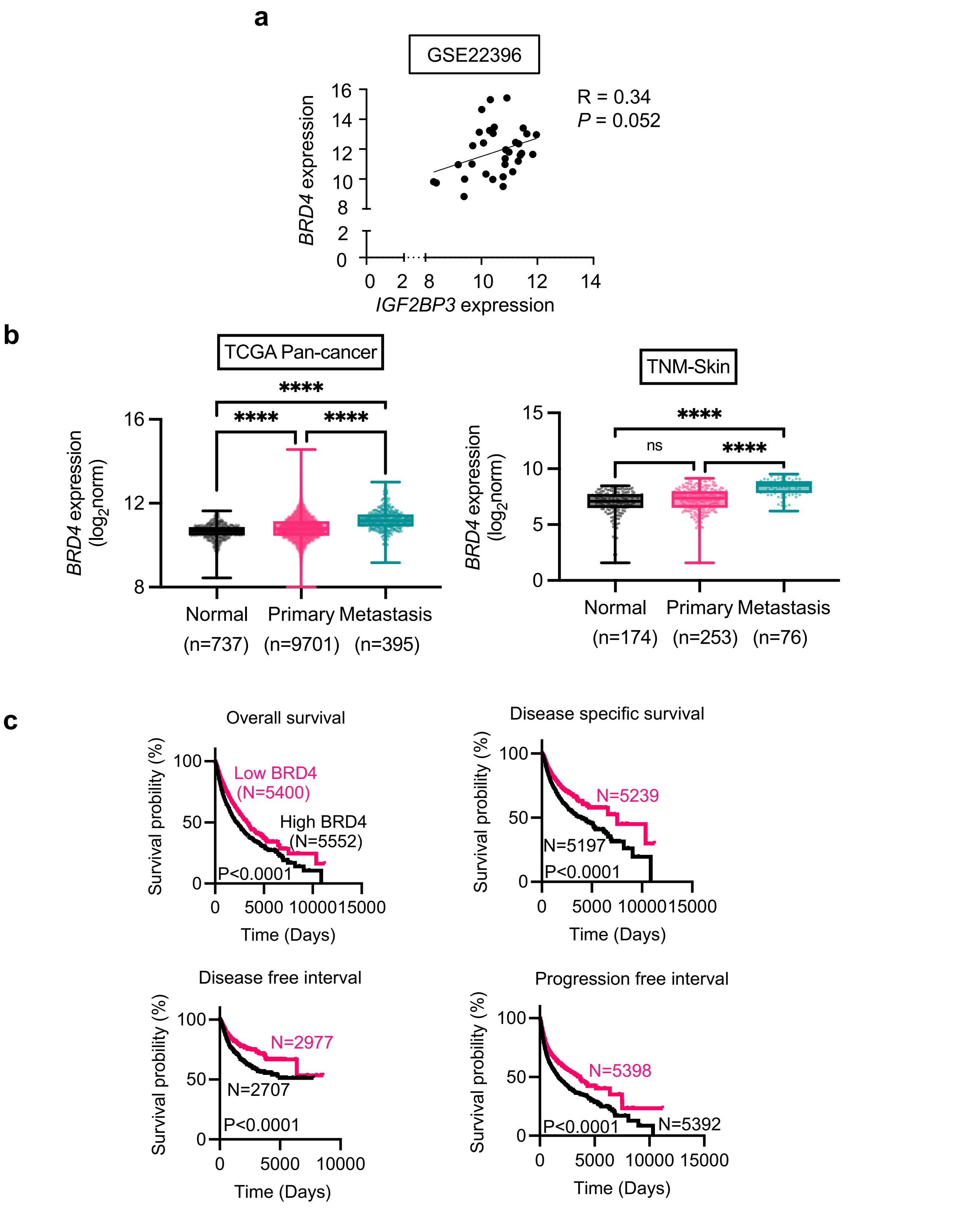

Supplementary Figure S15. Associations between BRD4 expression and IGF2BP3, metastasis and survival. (**a**) Correlation between *BRD4* expression and *IGF2BP3* expression in the GEO MCC cohort GSE22396. R=Pearson's correlation coefficient. (**b**) Increased BRD4 expression in metastases compared to primary tumors and normal tissues in the TCGA pan-cancer cohort overall as well as in the TNM skin cancer cohort. ****P<0.0001, ns=not significant by Kruskal-Wallis with post-hoc Dunn's-test.(**c**) Kaplan-Meier analysis illustrating shorter survival of patients with high as compared to low expression of BRD4 in the TCGA pan-cancer cohort. Median was used as the cut-off for low (pink) and high (black) *BRD4* expression.
